# A Longitudinal, Practical Curriculum for Faculty Development as New Coaches in Graduate Medical Education

**DOI:** 10.21980/J88M08

**Published:** 2025-07-31

**Authors:** Simanjit K Mand, Chariti Gent, Sharon Barbour, Benjamin H Schnapp

**Affiliations:** * University of Wisconsin School of Medicine and Public Health, BerbeeWalsh Department of Emergency Medicine, Madison, WI; ^ University of Wisconsin Madison, Division of Continuing Studies, Madison, WI

## Abstract

**Audience and Type of Curriculum:**

This coach development curriculum is designed for faculty physicians who have little to no prior experience with coaching in medical education.

**Length of Curriculum:**

The curriculum was developed for use over an entire academic year (12 months), but can be scaled to as few as 6 months due to the degree of schedule flexibility allowed by the structure of the educational approaches.

**Introduction:**

As coaching becomes more common in medical education, faculty educators are in a prime position to integrate a coaching approach in interactions with trainees. Because coaching is a unique skill set that requires targeted training and deliberate practice, there is a need for a detailed curriculum for faculty development as coaches. Adequate training will ensure faculty understand the coaching principles and have built a level of comfortability with the coaching approach prior to implementation with learners.

**Educational Goals:**

The aim of this curriculum is to provide a longitudinal, practical, and interactive coach training curriculum for faculty with no prior coaching experience.

**Educational Methods:**

The educational strategies used in this curriculum include: asynchronous learning with assigned reading material to ensure a basic understanding of core coaching principles; high-yield synchronous sessions involving a mix of didactics, small-group discussion and reflection, and simulated case-based scenarios to provide opportunity for faculty to practice coaching skills. Each phase of the curriculum (three in total) uses a different educational strategy to address a specific goal and associated objectives

**Research Methods:**

The educational content was evaluated with anonymous pre- and post-surveys that consisted of both Likert-style and open-ended questions. Surveys were designed based on the Kirkpatrick Evaluation Model. Three post-surveys were used to evaluate each phase of the curriculum.

**Results:**

Seven faculty participated in this curriculum. Kirkpatrick Levels 1 and 3 were used to evaluate faculty reactions and behaviors, respectively, in response to the curriculum. Participants reported a high satisfaction with the overall curriculum, increased understanding of coaching approach, increased comfort with incorporating coaching relational and communication skills, and increased comfort in approaching select resident scenarios. Unexpected results include faculty reporting use of coaching skills in interpersonal interactions outside of the professional space and an improved bond with other faculty participants.

**Discussion:**

This curriculum is an effective method for increasing faculty knowledge and comfort with coaching skills. This curriculum and its materials can be adapted for other audiences who have no prior coaching experience and seek to learn the fundamentals of coaching and its use in medical education.

**Topics:**

Faculty development, coaching in medical education.

## USER GUIDE

**Table:** 

List of Resources:	
Abstract	1
User Guide	3
Curriculum Chart	11
Appendix A: Curriculum Phase 1 Overview – Foundations of Coaching	19
Appendix B: Curriculum Phase 2 Overview – Faculty Development for the Coaching Approach, Core Content and Skills of Coaching	21
Appendix C: Curriculum Phase 3 Overview - Experience of Coaching	24
Appendix D1: Session 1: Foundations of the Coach Approach – Facilitator Guide	25
Appendix D2: Session 1: Foundations of the Coach Approach – Handout	33
Appendix D3: Session 1: Foundations of the Coach Approach – Slide Deck	35
Appendix E1: Session 2: Introduction to Core Coaching Skills – Facilitator Guide	36
Appendix E2: Session 2: The Coaching Framework – Handout	43
Appendix E3: Session 2: Introduction to Core Coaching Skills – Slide Deck	45
Appendix F1: Session 3: Deep Dive into Co-Creating Relationships – Facilitator Guide	46
Appendix F2: Session 3: Deep Dive into Co-Creating Relationships – Slide Deck	56
Appendix G1: Session 4: Deep Dive into the Skills of Effective Communication – Facilitator Guide	57
Appendix G2: Session 4: Deep Dive into the Skills of Effective Communication – Slide Deck	65
Appendix H1: Session 5: Deep Dive into Evoking Awareness – Facilitator Guide	66
Appendix H2: Session 5: Your Inner Critic – Handout	73
Appendix H3: Session 5: Individual Core Values List	74
Appendix H4: Session 5: Deep Dive into Evoking Awareness – Slide Deck	75
Appendix I1: Session 6: Deep Dive into Facilitating Learning and Growth – Facilitator Guide	76
Appendix I2: Session 6: Facilitating Learning and Growth – Handout	84
Appendix I3: Session 6: Deep Dive into Facilitating Learning and Growth – Slide Deck	85
Appendix J: Faculty Pre-Survey	86
Appendix K: Asynchronous / Phase 1 Survey	87
Appendix L: Didactic Sessions / Phase 2 Survey	88
Appendix M: Faculty Coaching / Phase 3 Survey	91

**Learner Audience:**

Faculty

**Length of Curriculum:**

The curriculum was developed for use over an entire academic year (~12 months), but can be scaled to as few as 6 months due to the degree of schedule flexibility allowed by the structure of the educational approaches.

**Topics:**

Faculty development, coaching in medical education.

**Objectives:**

After this longitudinal development program for faculty coaches, faculty learners will be able to:

1. Define the critical differences between coaching and mentoring
2. Understand how coaching can be, and is currently being, used in medical education
3. Understand the foundational principles of the coaching mindset and how to use this to build and maintain a successful coaching relationship
4. Learn and practice conversational and relational coaching skills in a small-group setting
5. Gain confidence in applying learned coaching skills in an environment simulating common faculty-resident interactions
6. Form a shared community that can continue to revise roles and responsibilities of a faculty coach in our program
7. Experience and reinforce the learned coaching principles and mindset in an optional coaching experience as the coachee
8. Demonstrate the logistical process of successful communication, agenda-setting for meetings, effective meeting frequency, and coachee-driven focus

### Brief introduction

Coaching in medical education is a practice distinct from mentoring and advising; coaching is a partnership between a coach and learner that focuses on performance improvement by facilitating self-reflection, informed learner self-evaluation, and creation of feasible goals that maximize a learner’s personal and professional potential.^1^ Coaching focuses on learners as experts in their experiences and motivations, centers on questioning and evoking awareness rather than telling, and creates a non-judgmental space to help learners align their core values with necessary training competencies and goals beyond training.^1^ The coach is present to facilitate conversations, deepen learner understanding, and help hold learners accountable to their goals.

Coaching in medical education has already proven to provide several individual benefits for learners.^2^ In addition to technical and non-technical skill improvement, coaching has also been noted to help in several well-being areas, particularly among resident physicians, including reducing imposter syndrome,^3^ building social support and increasing resilience,^4^ decreasing emotional exhaustion,^5^ and improving self-care.^2^ Furthermore, the Accreditation Council of Graduate Medical Education (ACGME) requires residency programs to have mechanisms in place to promote learner reflective practice and commitment to personal growth and well-being,^6^ and coaching has the potential to support these areas within residency education.

A resident coaching program was conceptualized in our department to address two overarching concerns that were extrapolated from our annual ACGME and institutional surveys in the years following the onset of the COVID pandemic. The first area for improvement was in resident satisfaction with faculty members’ feedback (72% compared to specialty compliance of 78%). The second was in resident well-being (20% reporting professional fulfillment and 74% reporting burn out).

After identifying academic and well-being coaching as target applications of our coaching program, the next immediate step became creating a faculty development curriculum for coaching. Faculty educators are well-positioned and well-equipped to become coaches for trainees in graduate medical education (GME) with their heavy investment in resident education and knowledge of programmatic assessment processes. However, coaching is still a unique skill set that requires targeted education and sufficient opportunity for deliberate faculty practice prior to use with learners.^7^–^10^

### Problem identification, general and targeted needs assessment

While national coaching certification programs exist,^11^ these are time-intensive, expensive, and suboptimal for highlighting the nuances of coaching use in medical education. Several resources provide general recommendations on necessary topics to learn and skills to practice for faculty development as coaches but lack specific practical or interactive components.^9^,^12^,^13^ Many programs have trained faculty with brief (commonly 1–2 sessions) one-on-one or group educational sessions that provide an overview of coaching principles,^5^,^10^,^14^–^19^ with some offering an additional printout reference sheet to help guide coach-coachee sessions.^5^,^14^ At the time of our curriculum development, no detailed curriculum existed outlining a clear and practical longitudinal approach to faculty development as coaches. Thus, we describe a multiphase curriculum that provides a practical approach and materials to train faculty as coaches in a graduate medical education program.

Through a comprehensive review of the medical education and faculty development literature, we identified a lack of a detailed faculty development curriculum for coaching in graduate medical education.

For the local needs assessment, one of the authors created a list of potential topics derived from the literature review. Since our participating faculty had no prior coach training, the content selected were the most foundational, yet practical, topics needed to get all faculty trained on core coaching principles and skills. Necessary curricular content included the core competencies of coaching, how coaching differs from other academic roles, and the nuances of coaching application in medical education. In consultation with the institution’s Director of Professional Coach Education (CG), a pre-existing didactic-based interactive curriculum was modified to highlight topics pertinent to our faculty group. Other curricular phases were developed to ensure foundational understanding of coaching principles and provision of an optional experience to be coached.

#### Participants

Our three-year EM residency program trains 13 residents per year. We sought to create a resident coaching program to pair each individual resident with a faculty coach.

Seven faculty participated in the curriculum after a department-wide solicitation for voluntary participation in a novel resident coaching program. All participants were core faculty with varying roles in medical education (including Assistant Residency Program Director, Assistant Director of Medical Student Clerkship, Director of Medical Student Clerkship, Director of Simulation, Director of Quality Improvement Curriculum for residents, Medical Education Fellow). The faculty group consisted of one fellow, five Assistant Professors, and one Associate Professor. Six of the participants were already involved in the Clinical Competency Committee (CCC) for the residency program and were familiar with the processes of formative and summative evaluations of residents. One participant joined CCC after becoming a faculty coach.

### Goals of the curriculum

A primary goal for this curriculum was to develop a longitudinal, practical, and interactive coach training program for faculty with no prior experience. We iteratively reviewed the needs assessment findings and developed three additional overarching curricular goals with targeted learning objectives for each. Curricular design was greatly influenced by the desire to have a multimodal approach to incorporate high-yield topics while maintaining a degree of schedule flexibility for faculty. Curricular goals were: 1) to introduce faculty to basic coaching concepts and principles, 2) to allow faculty to learn and practice coaching skills in a low-pressure, supportive environment, and 3) to provide faculty with the opportunity to participate in a coaching relationship as a coachee.

### Objectives of the curriculum

After this longitudinal development program for faculty coaches, faculty learners will be able to:

1. Define the critical differences between coaching and mentoring
2. Understand how coaching can be, and is currently being, used in medical education
3. Understand the foundational principles of the coaching mindset and how to use this to build and maintain a successful coaching relationship
4. Learn and practice conversational and relational coaching skills in a small-group setting
5. Gain confidence in applying learned coaching skills in an environment simulating common faculty-resident interactions
6. Form a shared community that can continue to revise roles and responsibilities of a faculty coach in our program
7. Experience and reinforce the learned coaching principles and mindset in an optional coaching experience as the coachee
8. Demonstrate the logistical process of successful communication, agenda-setting for meetings, effective meeting frequency, and coachee-driven focus

### Educational strategies

We used a variety of educational strategies, with each of the three phases of the curriculum targeting a separate goal and related objectives. Please see the curriculum chart below.

### Results and tips for successful implementation

#### Implementation

Implementation occurred over the course of one academic year (August 2023 to June 2024) with seven participating faculty. Phase 1 was completed from August 2023 to October 2023 and consisted of a suggested timeline to individually complete asynchronous reading and review of supplemental materials. Phase 2 was completed from January 2024 to March 2024 and consisted of six in-person sessions that were held every 1–2 weeks. Faculty attendance for each session was 86%, 100%, 86%, 100%, 86%, and 71%, respectively. An optional Phase 3 was completed from November 2023 to June 2024 and consisted of six 1:1 coaching sessions for each faculty participant who elected to partake. Five out of seven faculty voluntarily participated while the remaining two faculty independently sought out a coach external to this opportunity.

#### Results

The response rate for the pre-survey was 100% (6/6), and post-surveys were 83% (5/6), 100% (6/6), and 83% (5/6) for Phases 1–3, respectively. Consent was implied with survey return.

##### Phase 1: Foundations of Coaching

###### Kirkpatrick Level 1: Reaction

All participants reported reading 50% or more of the curated articles for supplemental reading, with all reporting that it was “somewhat effective” (80%) to “extremely effective” (20%) for learning foundation coaching principles. Three out of five participants reported reading 50% or more of the assigned reading from *Coaching in Medical Education*, with four out of five participants reporting this primary resource was “somewhat effective” (50%) to “extremely effective” (50%) for learning foundational coaching principles.

Many participants noted that these were useful resources that gave a framework for understanding coaching principles before the in-person sessions. However, many reported a desire for more accountability in the future to motivate them to read assigned materials. Some suggested linking asynchronous material to in-person sessions for spaced repetition and deeper understanding of the reading material provided.

##### Phase 2: Core Content and Skills of Coaching

###### Kirkpatrick Level 1: Reaction

All participants reported this training was “really worth my time.” Participants unanimously agreed that they would recommend this course to a colleague/provide positive reviews.

The majority of participants noted they preferred the in-person, small group format and would recommend the same frequency, every one-two weeks, for initial training. They all valued the practice and feedback during the synchronous sessions. Continued training was preferred on a monthly or quarterly basis.

Faculty participants reported the most useful things learned from the course were a script/algorithm to approach coaching with residents, how coaching differs from mentoring, and how to create a supportive space and maintain a growth mindset.

###### Kirkpatrick Level 3: Behavior

Five out of six faculty reported that they feel “confident” (60%) or “very confident” (40%) with facilitating coaching conversations with learners. Five out of six participants reported that being a resident coach is “increasing” their professional satisfaction. Table 2 provides additional qualitative feedback from faculty participants.

Table 3 compares pre-survey results to post-survey results assessing faculty comfort level when approaching select resident scenarios. While there was a positive trend in increasing comfort levels with each resident scenario after the training sessions, all faculty participants noted that they continue to feel challenged by residents who may be disengaged or who do not have as much buy-in to the process as their peers.

Unexpected results include how coaching allowed for not only great self-reflection and growth, but that participants also found use for integrating coaching skills in other interpersonal interactions. There was also an improved bond with other faculty participants. In addition, all participants reported that resident coaching is taking “more time” than anticipated, with one faculty noting “to do coaching well requires a significant time investment on our part.”

##### Phase 3: Experience of Coaching

###### Kirkpatrick Level 1: Reaction

All participants reported that their experience with an assigned physician coach “exceeded expectations.”

###### Kirkpatrick Level 3: Behavior

All faculty noted that this experience was “very helpful” in improving confidence in ability to coach and support residents, finding new ways to enhance relationships with residents, and improving their ability to help residents establish, and work towards, key goals. All faculty noted that this experience was “very helpful” (80%) or “helpful” (20%) in providing new insights on how to understand residents’ behaviors and motivations. All faculty noted that this experience was “very helpful” (60%) and “helpful” (40%) in developing their ability to discuss uncomfortable topics constructively with residents.

One faculty noted a future area for improvement was incorporating more intentional debriefing as a group, suggesting “debriefing our own coaching sessions, our resident coaching sessions, and training sessions.” Table 4 provides additional qualitative feedback from faculty participants.

### Evaluation and Feedback

Faculty reactions to this curriculum have been overwhelmingly positive. Faculty reported an increase in both their understanding of and comfort with using coaching skills at the conclusion of the curriculum. One main strength of the curriculum is the multimodal approach, with each curricular phase providing a different educational strategy and critical content. There was also a great degree of schedule flexibility throughout the curriculum, with the didactic-based phase being the only scheduled, in-person sessions. Additional unanticipated positives reported by faculty were the development of a shared community and connection among other faculty coaches, and the general applicability of coaching skills to other aspects of participants’ lives.

Areas for curricular improvement include the need to better integrate each of the phases as a cohesive curriculum. While the intent was to develop a curriculum with schedule flexibility, participants report limited use of the asynchronous material with a future desire to pair it with the synchronous portions of the curriculum for deeper understanding and more accountability. Formal group debriefing was also suggested by some participants, which may deepen understanding and a sense of shared community. Lastly, even with the attempt to limit faculty time commitment, participants still note that resident coaching was overall exceeding the amount of time they were expecting to commit to the opportunity. This is likely multifactorial, including the individual time spent with each resident coachee; however, it may in part be due to the several curricular phases. Since all participants viewed each phase positively, the time burden may be best managed with future time or salary compensation for coaches. More streamlined iterations of the curriculum could also be examined in the future, such as removing the optional experience of being coached.

Future iterations of our curriculum will work to more seamlessly integrate different aspects of the curriculum (assigning asynchronous content to synchronous group sessions), and provide more opportunities for coaches to debrief all of their experiences as a group.[Table t1-jetem-10-3-c1]

### Associated content

#### Appendices

A. Curriculum Phase 1 Overview – Foundations of Coaching
B. Curriculum Phase 2 Overview – Faculty Development for the Coaching Approach, Core Content and Skills of Coaching
C. Curriculum Phase 3 Overview - Experience of Coaching
D1. Session 1: Foundations of the Coach Approach – Facilitator Guide
D2. Session 1: Foundations of the Coach Approach – Handout
D3. Session 1: Foundations of the Coach Approach – Slide Deck
E1. Session 2: Introduction to Core Coaching Skills – Facilitator Guide
E2. Session 2: The Coaching Framework – Handout
E3. Session 2: Introduction to Core Coaching Skills – Slide Deck
F1. Session 3: Deep Dive into Co-Creating Relationships – Facilitator Guide
F2. Session 3: Deep Dive into Co-Creating Relationships – Slide Deck
G1. Session 4: Deep Dive into the Skills of Effective Communication – Facilitator Guide
G2. Session 4: Deep Dive into the Skills of Effective Communication – Slide Deck
H1. Session 5: Deep Dive into Evoking Awareness – Facilitator Guide
H2. Session 5: Your Inner Critic – Handout.docx
H3. Session 5: Individual Core Values List.
H4. Session 5: Deep Dive into Evoking Awareness – Slide Deck
I1. Session 6: Deep Dive into Facilitating Learning and Growth – Facilitator Guide
I2. Session 6: Facilitating Learning and Growth – Handout
I3. Session 6: Deep Dive into Facilitating Learning and Growth – Slide Deck
J. Faculty Pre-Survey
K. Asynchronous / Phase 1 Survey
L. Didactic Sessions / Phase 2 Survey
M. Faculty Coaching / Phase 3 Survey

### Evaluation and feedback

Faculty reactions to this curriculum have been overwhelmingly positive. Faculty reported an increase in both their understanding of and comfort with using coaching skills at the conclusion of the curriculum. One main strength of the curriculum is the multimodal approach, with each curricular phase providing a different educational strategy and critical content. There was also a great degree of schedule flexibility throughout the curriculum, with the didactic-based phase being the only scheduled, in-person sessions. Additional unanticipated positives reported by faculty were the development of a shared community and connection among other faculty coaches, and the general applicability of coaching skills to other aspects of participants’ lives.

Areas for curricular improvement include the need to better integrate each of the phases as a cohesive curriculum. While the intent was to develop a curriculum with schedule flexibility, participants report limited use of the asynchronous material with a future desire to pair it with the synchronous portions of the curriculum for deeper understanding and more accountability. Formal group debriefing was also suggested by some participants, which may deepen understanding and a sense of shared community. Lastly, even with the attempt to limit faculty time commitment, participants still note that resident coaching was overall exceeding the amount of time they were expecting to commit to the opportunity. This is likely multifactorial, including the individual time spent with each resident coachee; however, it may in part be due to the several curricular phases. Since all participants viewed each phase positively, the time burden may be best managed with future time or salary compensation for coaches. More streamlined iterations of the curriculum could also be examined in the future, such as removing the optional experience of being coached.

Future iterations of our curriculum will work to more seamlessly integrate different aspects of the curriculum (assigning asynchronous content to synchronous group sessions), and provide more opportunities for coaches to debrief all of their experiences as a group.

## Supplementary Information













## Figures and Tables

**Table:** 

Topic	Recommended Educational Strategy	Educational Content	Objectives	Learners	Timing, Resources Needed	Recommended Assessment, Milestones Addressed
Phase 1: Foundations of Coaching and Coaching Use in Medical Education.	Assigned asynchronous reading material and video resources.	***Coaching in Medical Education*** book chapters describing coaching in the academic environment, coaching theories, competencies for academic coaches, and applications of coaching: Chapter 1: Coaching in the Academic Environment Chapter 2: Coaching and the Master Adaptive Learner Model Chapter 3: Coaching Theories: A Scientific Foundation for Coaching Competencies in Medical Education Chapter 4: Competencies for Academic Coaches Chapter 5: Overview of Individual, Team, and Peer Coaching Chapter 6: Applications of Coaching *While not used in our curriculum, the following free resource can be used in place of the handbook referenced above: https://www.amaassn.org/system/files/2019-09/coaching-medical-education-faculty-handbook.pdf **Assigned supplemental articles:** Wolff M, Deiorio NM, Miller Juve A, et al. Beyond advising and mentoring: Competencies for coaching in medical education. *Med Teach*. 2021;43(10):1210–1213. doi:10.1080/0142159X.2021.1947479 https://www.tandfonline.com/doi/full/10.1080/0142159X.2021.1947479 Sargeant J, Lockyer JM, Mann K, et al. The R2C2 model in residency education: How does it foster coaching and promote feedback use? *Acad Med*. 2018;93(7). doi:10.1097/ACM.0000000000002131 https://journals.lww.com/00001888-201807000-00030 Lovell B. What do we know about coaching in medical education? A literature review. *Med Educ*. 2018;52(4):376–390. doi:10.1111/medu.13482 https://onlinelibrary.wiley.com/doi/10.1111/medu.13482 MacKenzie C, Chan TM, Mondoux S. Clinical improvement interventions for residents and practicing physicians: A scoping review of coaching and mentoring for practice improvement. *AEM Educ Train*. 2019;3(4). doi:10.1002/aet2.10345 https://onlinelibrary.wiley.com/doi/10.1002/aet2.10345 Sargeant J, Mann K, Manos S, et al. R2C2 in action: Testing an evidence-based model to facilitate feedback and coaching in residency. *J Grad Med Educ*. 2017;9(2). doi:10.4300/JGME-D-16-00398.1 https://meridian.allenpress.com/jgme/article/9/2/165/34733/R2C2-in-Action-Testing-an-EvidenceBased-Model-to Sargeant J, Lockyer J, Mann K, et al. Facilitated reflective performance feedback: Developing an evidence-and theory-based model that builds relationship, explores reactions and content, and coaches for performance change (R2C2). *Acad Med*. 2015;90(12). doi:10.1097/ACM.0000000000000809 http://journals.lww.com/00001888-201512000-00034 **Videos:** American Medical Association Video Series on Medical Academic Coaching: https://edhub.amaassn.org/pages/develop-academiccoaching-skillsmedical-education Both supplemental articles and videos help to demonstrate real use of coaching in different medical education environments	To understand the critical differences between coaching, mentoring, and advising. To understand how coaching can be, and is currently being, used in medical education. To understand the foundational principles of the coaching mindset and how to use this to build and maintain a successful coaching relationship.	Faculty physicians	Timing: - ~10 hours for reading content - ~80 minutes video review Resources: - *Coaching in Medical Education* book - Supplemental articles and reference video link (Appendix A) Post-survey	Assessment: Post-survey (Appendix K)
Session 1: Foundations of the Coach Approach *Suggested pre-reading:* *Chapters 1–2* *Lovell article* *MacKenzie article*	Lecture defining coaching and coaching principles. Demonstration of mentoring vs. coaching approach to conversation. Paired-practice of active listening. Paired and group debriefing of active listening.	Introduce disciplines that inform coaching, coaching core beliefs and mindset, and coaching literature from medical education. Demonstrate the coaching approach to a conversation. Practice active listening.	To understand the role of a coach (vs. mentor or advisor) and qualities of a successful coach. To understand the ethics and competencies of professional coaching as set by the International Coach Federation. To introduce and practice active listening.	Faculty physicians	Timing: 60 minutes for lecture 15 minutes for demonstration 15 minutes for practice and debrief Resources: - Instructors: 2 - Equipment: project/screen Material: lecture (Appendix D3), facilitator guide (Appendix D1), pre-session handouts (Appendix D2)	Assessment: Pre-and post-surveys (Appendices J,L)
Session 2: Introduction to Core Coaching Skills *Suggested pre-reading:* *Chapters 3–4* *Wolff article*	Lecture defining core skills of a professionally trained coach. Demonstration of asking powerful questions. Paired-practice of asking powerful questions and beginning a coaching conversation. Paired and group debriefing of asking powerful questions.	Describe coaching curiosity, what makes a powerful question. Describe the general structure of a coaching session: the beginning, middle, and end. Demonstrate core skills of a professionally trained coach. Practice asking powerful questions and beginning a coaching conversation.	To define professional coaching curiosity. To describe what makes a question powerful. To practice using powerful questions in a coaching context. To identify the 8 core competencies of professional coaching. To understand the basic structure of a coaching session and how to apply it to a coaching relationship.	Faculty physicians	Timing: 45 minutes for lecture 10 minutes for demonstration 35 minutes for practice and debrief Resources: - Instructors: 2 - Equipment: project/screen Material: lecture (Appendix E3), facilitator guide (Appendix E1), pre-session handout (Appendix E2)	Assessment: Pre-and post-surveys (Appendices J,L)
Session 3: Deep Dive into Co-Creating Relationships *Suggested pre-work:* *AMA videos*	Lecture defining how to effectively establish and maintain coaching agreements. Demonstration of creating the designed alliance. Paired-practice on reflecting on a relationship of trust and safety, and designing your own alliance for being with your learner. Paired and group debriefing of reflecting on a relationship of trust and safety, and designing your own alliance for being with your learner.	Describe how to cultivate trust and safety with a coachee. Demonstrate how to structure a coaching relationship. Understanding how to define primary focus areas. Practice reflecting on a relationship that has brought you a sense of trust and safety. Practice designing your own alliance with your learners.	To describe how to design an alliance with your coachee. To learn how to build trust and safety in a coaching relationship. To demonstrate how to co-create the structure of a relationship and define primary focus areas.	Faculty physicians	Timing: 20 minutes for lecture 30 minutes for demonstration 40 minutes for practice and debrief Resources: - Instructors: 2 - Equipment: project/screen Material: lecture (Appendix F2), facilitator guide (Appendix F1)	Assessment: Pre- and post-surveys (Appendices J,L)
Session 4: Deep Dive into the Skills of Effective Communication *Suggested pre-reading:* *Sargeant articles x3*	Lecture defining active listening, questioning powerfully, and talking succinctly and directly. Demonstration of different levels of active listening Paired-practice with asking powerful questions, active listening, holding silence. Paired and group debriefing of asking powerful questions, active listening, holding silence.	Describe the three levels of listening: internal, focused on the other, and global. Describe characteristics and importance of direct communication. Practice asking powerful questions, listening actively, and holding silence.	To define effective communication in coaching. To practice active listening, questioning powerfully, and talking succinctly and directly. To describe the importance of direct communication.	Faculty physicians	Timing: 10 minutes for lecture 20 minutes for demonstration 60 minutes for practice and debrief Resources: - Instructors: 2 - Equipment: project/screen Material: lecture (Appendix G2), facilitator guide (Appendix G1)	Assessment: Pre- and post-surveys (Appendices J,L)
Session 5: Deep Dive into Evoking Awareness	Lecture defining values and how to evoke awareness around values and the internal critic. Demonstration of addressing the inner critic. Paired-practice with discussing values and addressing your inner critic. Paired and group debriefing of discussing values and addressing your inner critic.	Describe values and learn how to identify them. Identify the inner critic. Practice exploring one’s core values, how the inner critic may limit aligning goals with values, and how to gain deeper insight into emotions and thoughts.	To discover how to shed light on one’s values and beliefs. To understand the relationship between experience, thoughts, emotions, and actions.	Faculty physicians	Timing: 45 minutes for lecture 15 minutes for demonstration 30 minutes for practice and debrief Resources: - Instructors: 2 - Equipment: project/screen Material: lecture (Appendix H4), facilitator guide (Appendix H1), pre-session handouts (Appendices H2-H3)	Assessment: Pre- and post-surveys (Appendix J,L)
Session 6: Deep Dive into Facilitating Learning and Growth *Suggested pre-reading:* *Chapters 5–6*	Lecture providing tools on how to integrate new awareness into action. Demonstration of how to move insight into action. Paired-practice with moving insight into action. Paired and group debriefing of moving insight into action.	Describe how to facilitate client growth: bottom line, bridge to action, barriers, bolsters. Practice moving insight into action.	To integrate insight into action. To highlight the importance of presence.	Faculty physicians	Timing: 45 minutes for lecture 10 minutes for demonstration 35 minutes for practice and debrief Resources: - Instructors: 2 - Equipment: project/screen Material: lecture (Appendix I3), facilitator guide (Appendix I1), pre-session handout (Appendix I2)	Assessment: Pre- and post-surveys (Appendices J,L)
Phase 3: Experience of Coaching	1. Providing an optional, real-life example of coaching from the coachee perspective.	Variable depending on coach : coachee agenda.	To experience and reinforce the learned coaching principles and mindset in an optional coaching experience as the coachee. To demonstrate the logistical process of successful communication, agenda-setting for meetings, effective meeting frequency, and coachee-driven focus.	Faculty physicians	Timing: ~60 minutes for each session Resources: - Formally-trained faculty coach: 1 Equipment: computer/laptop for virtual meeting platform	Post-survey (Appendix M)

**Table 1 t1-jetem-10-3-c1:** Core Content and Skills of Coaching (Curriculum Phase 2 Overview)

Goal 2: To allow faculty to learn and practice coaching skills in a low-pressure, supportive environment As previous studies denote, the importance of the simulated practice of coaching skills prior to implementation (see manuscript for citations), as an in-person, didactic-based, interactive series for education and practice was deemed necessary for faculty training and coach development. Author SKM partnered with the institution’s Director of Professional Coach Education, CG, to include a modified pre-existing didactic-based interactive curriculum used to train faculty in other departments within the institution. Objectives: To learn and practice conversational and relational coaching skills in a small-group setting To gain confidence in applying learned coaching skills in an environment simulating common faculty-resident interactions To form a shared community that can continue to revise roles and responsibilities of a faculty coach in our program			
Workshop Title	Objectives	Teaching Strategies	Length
Foundations of the Coach Approach	To define the role of a coach To define the ethics and competencies of professional coaching as set by the International Coach Federation	Didactics, Small group discussion, Simulated case-based scenarios	90 min
Introduction to Core Coaching Skills	To introduce the basic structure of a coaching session and relationship	Didactics, Small group discussion, Simulated case-based scenarios	90 min
Deep Dive into Co-Creating Relationships	To describe how to effectively establish and maintain coaching agreements	Didactics, Small group discussion, Simulated case-based scenarios	90 min
Deep Dive into the Skills of Effective Communication	To explore skills of listening actively, questioning powerfully, and talking succinctly and directly	Didactics, Small group discussion, Simulated case-based scenarios	90 min
Deep Dive into Evoking Awareness	To discover how to shed light on one’s values and beliefs To understand the relationship between experience, thoughts, emotions, and actions	Didactics, Small group discussion, Simulated case-based scenarios	90 min
Deep Dive into Facilitating Learning and Growth	To provide tools on how to integrate new awareness into action	Didactics, Small group discussion, Simulated case-based scenarios	90 min

**Table 2:** Select Qualitative Faculty Feedback from Phase 2

Core Content and Skills of Coaching: Didactic-Based Curriculum
**What is the most useful thing that you’ve learned during this course?** “The coaching mindset can be applied to all interpersonal interactions.” “Learning a script/algorithm for coaching residents” “Creating space, maintaining a growth mindset” “I learned about how coaching differs from mentoring and the importance of structuring a coaching meeting” **What are your suggestions for improving our training?** “Practice coaching with simulated coachees who are less enthusiastic/engaged.” “Sessions where [we] can acknowledge the difference in our program from traditional coaching relationships & defining our role.” “Would love something more sustainable & more long term to maintain skills.” **What unexpected results, if any, did you get from this program?** “Some great reflection on myself as an educator, physician, person. Introspection and changes in mindset.” “Improved bond with other coach faculty.” “How much I would use these skills for non-coaching related interpersonal interactions.” “I learned to view every person (including mentees, patients, and also myself) as naturally creative, resourceful, and whole.” **What would you tell a colleague about this program?** “Definitely worthwhile to undertake if you want to learn how to approach mentees differently.” “It is a way to learn concrete tools that allow you to become a better listener, builds empathy and helps guide changing perspectives and approaches to problem solving.” “Great program - really solid start for a small group to learn coaching techniques.”

**Table 3:** Self-Reported Faculty Comfort Levels When Approaching Select Resident Scenarios

Helping individual residents…	Mean Baseline Score (Pre)^a^	Mean Post-Training Score (Post)^a^
Through personal struggles	3.83	4.33
Who are struggling with clinical skills	4.33	4.50
Who are struggling with professional concerns	3.50	4.00
Who are disengaged	3.50	4.00
Who are already excelling in residency	3.83	4.33

a Rated on a 5-point Likert-type scale (1=very uncomfortable, 5=very comfortable)

**Table 4:** Select Qualitative Faculty Feedback from Phase 3

Experience of Coaching: Faculty Participants as Coachees
**What did you gain from this coaching experience (personally or professionally)?** “I gained a lot of insight about coaching the residents but…learned a lot about myself.” “A better understanding of how coaching differs from mentoring, the value of having a coach, accomplishment of some of my personal and professional goals that I wouldn’t have been able to achieve otherwise.” “It helped me become better at self-reflection. Provided an excellent role-model for how I can do my own coaching more effectively.” “It helped me professionally to see a coach ‘do their thing’ and gain helpful tips by going through the experience.” **How did this experience influence your understanding of what a successful coaching relationship looks/feels like?** “It really allowed me to experience what professional coaching was which allowed me to learn from seeing…” “It really showed me how coaching was different from mentorship and highlighted how coachee-driven the process is.” **How did this experience affect your own approach to coaching with residents (and other trainees)?** “Provided me with additional techniques to help trainees open up and ask questions in a way that allows them to direct much of the discussion and its outcome.” “I don’t think I could approach coaching and how different it is from mentorship without being able to fully experience it firsthand myself.” “Before the training, I naturally operated in a mentorship role. Now, I feel much more comfortable assuming a true coaching role where I can aid the residents in developing their own goals, trajectory instead of being prescriptive.” **What are some suggestions to optimize this experience in the future?** “Allow for the coaches to continue this experience longitudinally. It adds so much insight and clarity.” “Since coaching is a new concept to many (if not all) of us, it would be great to have some sort of protected time or buy down in order to devote more time to develop our coaching skills.” “I think debriefing as a group would be more helpful - debriefing our own coaching sessions, our resident coaching sessions, and training sessions.”

**Figure:**
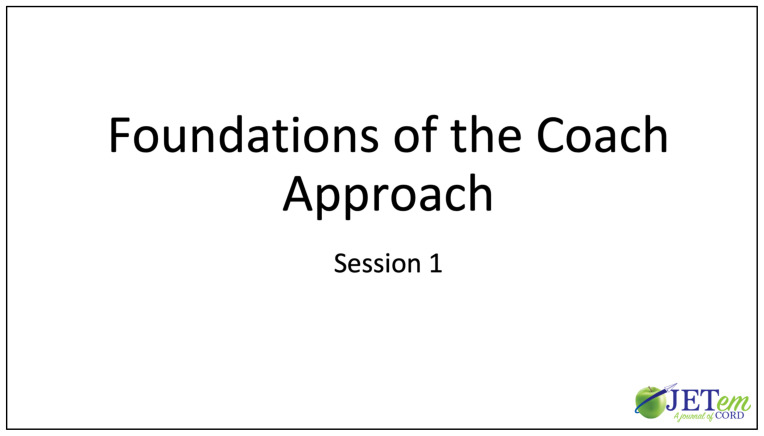
Please see associated Power Point

**Figure:**
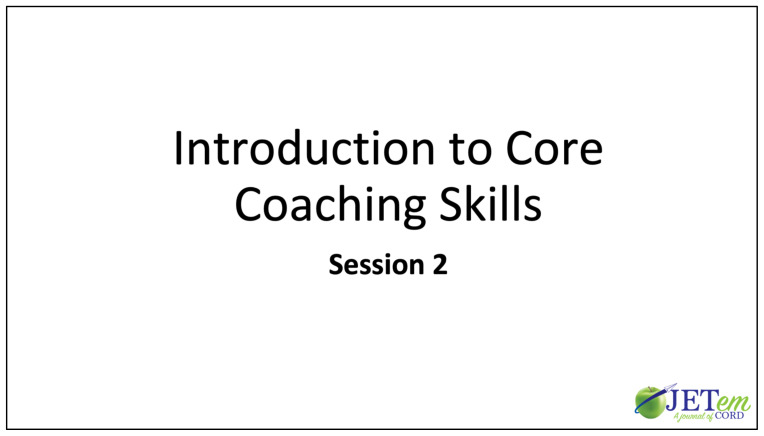
Please see associated Power Point

**Figure:**
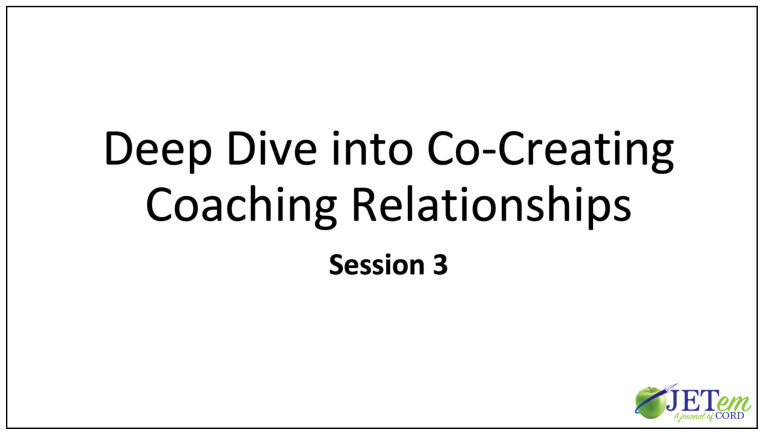
Please see associated Power Point

**Figure:**
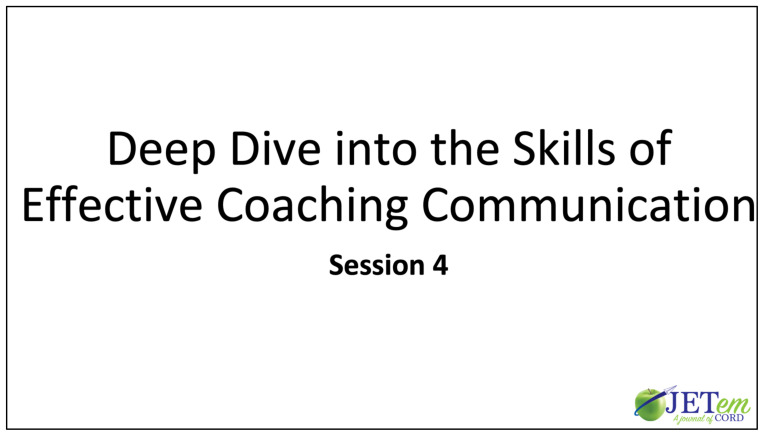
Please see associated Power Point

**Figure:**
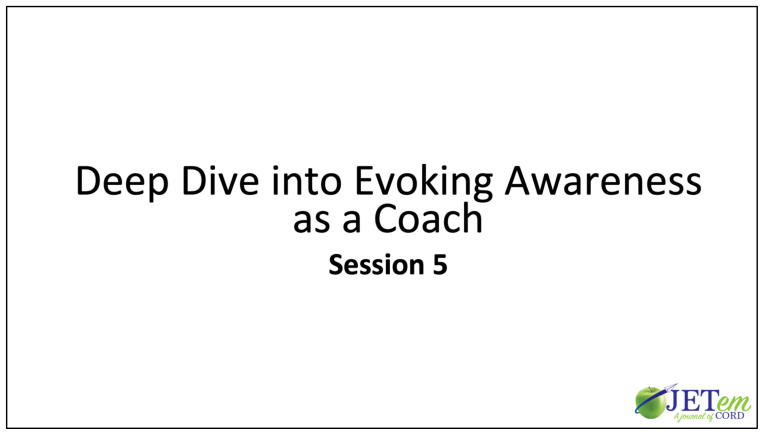
Please see associated Power Point

**Figure:**
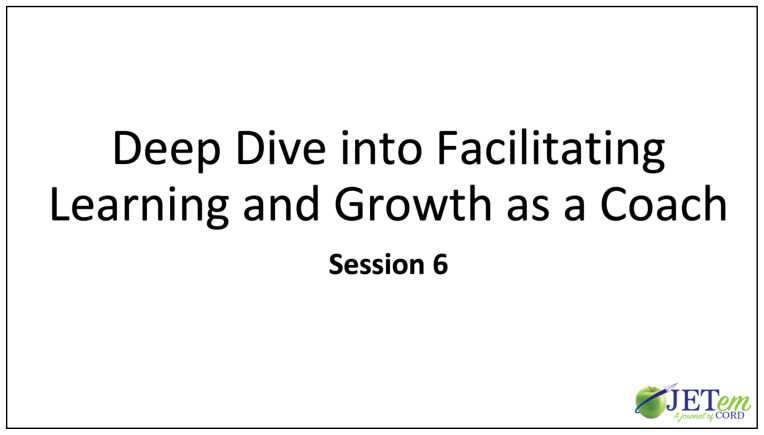
Please see associated Power Point

## Appendix A Curriculum Phase 1 Overview – Foundations of Coaching

**Table:**

Goal 1: To introduce faculty to basic coaching concepts and principles In order to minimize burden on existing clinical and academic responsibilities, an asynchronous list of reading and video material was curated to provide a foundation of coaching concepts and principles. Objectives: To define the critical differences between coaching, mentoring, and advising To understand how coaching can be, and is currently being, used in medical education To understand the foundational principles of the coaching mindset and how to use this to build and maintain a successful coaching relationship		

Reference book	*Coaching in Medical Education*	
	Aug:	**Chapter 1**: Coaching in the Academic Environment
		**Chapter 2**: Coaching and the Master Adaptive Learner Model
		**Chapter 3**: Coaching Theories: A Scientific Foundation for Coaching Competencies in Medical Education
	Sept:	**Chapter 4**: Competencies for Academic Coaches
		**Chapter 5**: Overview of Individual, Team, and Peer Coaching
		**Chapter 6**: Applications of Coaching
	*Can substitute this resource with the following free faculty handbook: https://www.ama-assn.org/system/files/2019-09/coaching-medical-education-facultyhandbook.pdf	

Curated list of recommended articles	Wolff M, Deiorio NM, Miller Juve A, et al. Beyond advising and mentoring: Competencies for coaching in medical education. *Med Teach*. 2021;43(10):1210–1213. doi:10.1080/0142159X.2021.1947479 https://www.tandfonline.com/doi/full/10.1080/0142159X.2021.1947479 Sargeant J, Lockyer JM, Mann K, et al. The R2C2 model in residency education: How does it foster coaching and promote feedback use? *Acad Med*. 2018;93(7). doi:10.1097/ACM.0000000000002131 https://journals.lww.com/00001888-201807000-00030 Lovell B. What do we know about coaching in medical education? A literature review. *Med Educ*. 2018;52(4):376–390. doi:10.1111/medu.13482 https://onlinelibrary.wiley.com/doi/10.1111/medu.13482 MacKenzie C, Chan TM, Mondoux S. Clinical improvement interventions for residents and practicing physicians: A scoping review of coaching and mentoring for practice improvement. *AEM Educ Train*. 2019;3(4). doi:10.1002/aet2.10345 https://onlinelibrary.wiley.com/doi/10.1002/aet2.10345 Sargeant J, Mann K, Manos S, et al. R2C2 in action: Testing an evidence-based model to facilitate feedback and coaching in residency. *J Grad Med Educ*. 2017;9(2). doi:10.4300/JGME-D-16-00398.1 https://meridian.allenpress.com/jgme/article/9/2/165/34733/R2C2-in-Action-Testingan-EvidenceBased-Model-to Sargeant J, Lockyer J, Mann K, et al. Facilitated reflective performance feedback: Developing an evidence-and theory-based model that builds relationship, explores reactions and content, and coaches for performance change (R2C2). *Acad Med*. 2015;90(12). doi:10.1097/ACM.0000000000000809 http://journals.lww.com/00001888-201512000-00034	

Recommended demonstrational videos	American Medical Association Video Series on Medical Academic Coaching: https://edhub.ama-assn.org/pages/develop-academic-coaching-skills-medical-education	

## Appendix B Curriculum Phase 2 Overview – Faculty Development for the Coaching Approach, Core Content and Skills of Coaching

### Session 1: Foundations of the Coach Approach (90 Minutes)

This session describes the nature and distinctive characteristics of a coach approach based on the ethics and competencies of professional coaching as promulgated by the International Coach Federation (ICF). It provides an overview of the qualities of a successful coach, including what it means to be a coach (vs. mentor, teacher, or advisor), as well as how to build effective and trusting coaching-like relationships within the UW Department of Emergency Medicine.

Key topics include:

- What is coaching?
- Qualities of a successful coach
- Trust and safety in the coaching context
  ○ The Designed Alliance
  ○ The role of cultural context
  ○ Empathy and vulnerability as tools
  ○ Partnering for empowerment
  ○ Being fully present as coach

### Session 2: Introduction to Core Coaching Skills (90 Minutes)

This session introduces the basic structure of a coaching session and relationship, and it provides direct instruction in the core skills of a professionally trained coach.

Key topics include:

- Co-creating the coaching relationship
- Effective communication
- Evoking awareness
- Facilitating learning and growth

### Session 3: Deep Dive into Co-Creating Relationships (90 Minutes)

This session provides participants an opportunity to practice how to effectively establish and maintain coaching agreements within the coaching session and relationship. Participants are encouraged to bring questions and be ready to discuss not only the scenarios in the assigned case studies but also their own real-world experiences in this area, too.

Key topics include:

- Goal setting
- The Designed Alliance in practice
- Trust and safety-building tips and ideas

### Session 4: Deep Dive into the Skills of Effective Communication (90 Minutes)

This module focuses on the skills of effective communication. You’ll explore examples of listening actively, questioning powerfully, and talking succinctly and directly—all in a way to benefit the person being coached. Participants are encouraged to bring questions and be ready to discuss not only the scenarios in the assigned case studies but also their own real-world experiences in this area, too.

Key topics include:

- The three levels of listening
- Leveraging the power of silence
- How to design and ask powerful questions
- Principles of direct communication

### Session 5: Deep Dive into Evoking Awareness (90 Minutes)

This session presents strategies for partnering to evoke new learning and awareness around goals. You’ll also be introduced to the importance of recognizing new insights not just around a situation but also around the growth one is experiencing as a person. Participants are encouraged to bring questions and be ready to discuss not only the scenarios in the assigned case studies but also their own real-world experiences in this area, too.

Key topics include:

- Discovering helpful new solutions, values, and beliefs
- Understanding the relationship between experience, thoughts, emotions, and actions
- Uncovering habitual behaviors that serve (or don’t)

### Session 6: Deep Dive into Facilitating Learning and Growth (90 Minutes)

This session gives participants an opportunity to learn about the necessary tools and steps for integrating new awareness into action. You will also learn how to hold the coachee accountable in a way that is supportive and sets up the coachee for long-term success. Participants are encouraged to bring questions and be ready to discuss not only the scenarios in the assigned case studies but also their own real-world experiences in this area, too.

Key topics include:

- Sourcing sustainable supports
- Managing obstacles
- Acknowledging and championing
- Celebrating successes

## Appendix C Curriculum Phase 3 Overview - Experience of Coaching

**Table:**

Goal 3: To provide faculty with the opportunity to participate in a coaching relationship as a coachee Faculty were offered the opportunity to receive coaching from a formally-trained faculty coach from a variety of academic departments at our institution. This was an opportunity for the faculty participants to experience what it is like to be coached from the other side of the table, experience a successful coaching relationship, and to inform future interactions with resident coachees. Objectives: To experience and reinforce the learned coaching principles and mindset in an optional coaching experience as the coachee To demonstrate the logistical process of successful communication, agenda-setting for meetings, effective meeting frequency, and coachee-driven focus	
Faculty coaches	Four formally-trained faculty coaches from a variety of departments (emergency medicine, pediatrics, dermatology, and internal medicine) served as the coaches for Phase 3
Meeting Logistics	Program consisted of six individual sessions (1 hour/session) Scheduled independently by coach and coachee
Meeting Content	Per the discretion of the coach and coachee* *No details requested or discussed from these individual meetings

## Appendix D1 Foundations of the Coach Approach – Facilitator Guide

**Table:**

Time	Slide	Notes/Presenter Talking Points

8:00–8:08	Slide 1 Welcome	**Welcome and Introduction** *Welcome everyone! We are so excited to be together for this series of workshops. As always, our intention is that our days together will provide connection, learning from one another, practice, and building on the skills you already have*.
	Introductions	Introduce facilitators Introduce participants (all and in person) Optional - ice breaker question: *What food, when you think about it, immediately takes you back in time (and why)?*
	Slide 2 Invitations	*Let’s begin our day by being present to this Coach Approach program, to each other, and to ourselves*. *Let’s start by being present in this room and learning space*. ***Our Invitations for Learning & Growth*** *Finally, let’s be present with ourselves.* ***Grounding activity - holistic and present***

8:08–8:10	Slide 3	**Start With Why**
	Slide 4	**Learning Objectives & Agenda**. Big picture: *Today’s session, as well as the next one, are going to provide a broad and general picture of professional coaching, as well as how it relates to interacting with learners. As we move into our final four meetings together, we’ll be revisiting the concepts introduced in the first two sessions and do a much deeper dive into particular skills that we believe will be most valuable to you as faculty coaches*. *By the end of today’s meeting, you will be able to*: **Refer** to the research on professional coaching and its effectiveness **Distinguish** between mentoring, coaching, advising, teaching **Apply** two foundational coaching competencies between now and our next meeting

8:10–8:25	No slide	**ACTIVITY: DEMO - Mentoring vs Coaching** Topic: How to build capacity for taking in DEIB (diversity, equity, inclusion, and belonging) perspectives in our coaching training? *Watch us mentor; then watch us coach. Jot down notes about what is different and what is similar*. Mentoring Demo (3 min) Coaching Demo (5 mins) - be sure to do trust and safety up front Debrief: what did you notice? (5–7 min) Instructors to highlight comments around: trust and safety partnering asking, not telling/emphasize curiosity and not knowing/pq’s (powerful questions) less talking on the part of the coach (than mentor) more listening/nonverbals reflective offers/holding up the mirror coachee directs the conversation *We’ve just seen the difference between coaching and mentoring*. *We saw how it worked in this short demo*.

8:25–8:35	Slide 5	**How Coaching is Different** Definitions of Coaching *Let’s talk about how the ICF defines coaching*. ICF: ICF defines coaching as partnering with clients in a thought-provoking and creative process that inspires them to maximize their personal and professional potential. The process of coaching often unlocks previously untapped sources of imagination, productivity, and leadership. *We all have goals we want to reach, challenges we’re striving to overcome, and times when we feel stuck. Partnering with a coach can change your life, setting you on a path to greater personal and professional fulfillment*.
	Slide 6	*Given that definition, let’s look again at how coaching is different from mentoring, counseling, and consulting* Session 1 handout discussion

8:35–8:38	Slide 7	**Disciplines That Inform Coaching** *We’ve explored what distinguishes coaching from other related professions. And now - Where does coaching come from?* *It’s a pretty new profession - it has been around for about 30 years*. Coaching draws from multiple disciplines, including: positive psychology behavioral psychology/CBT (cognitive behavioral therapy) motivational Interviewing learning theory adult development theory mindfulness neuroscience
	Slide 8	**Coaching Core Beliefs - (2 min)** *All coaching is about establishing a trusting, collaborative relationship that leads to coachees’ self-reflection and generative steps forward that serve them AND are chosen by them. Coaching core beliefs supports this trust and safety and development of a safe and courageous space to grow*. **Creative Resourceful and Whole (CRW)**: we look for strengths and give space for coachees’ own insights to emerge - they have tremendous inner resources! **Coachees are expert** in their own life -- they know themselves. **Coach the person, not the problem:** We hold that coaching is transformational not transactional. We focus on development and inner change over solving one problem at a time. **Unconditional Positive Regard (UPR):** Each person is worthy of UPR. We are all human and all have issues and brilliance. **Curiosity reigns/not knowing:** Create space for new awareness and insight, new possibilities. **Power in the relationship (partnering and presence):** The power isn’t about the coach’s power - it’s in the relationship, the partnering, the presence. **Trust and safety are foundational**. **Awareness is necessary for transformation/change:** New awareness put into action leads to lasting change.

8:38–8:40	Slide 9	**Why Do People Come to Coaching? Sample Issues in Coaching** **Ask folks to raise their hands or shout it out:** *what do you think people come to a coach for?* Transitions - work and life Habit change, behavior change Enhance leadership/management skills Increase confidence Goal attainment Define values Envision the future Increase awareness and resilience Team building Relationship building Burnout, stress Bridge: *Some of the research on coaching and burnout and stress has been done in the field of medicine. Let’s talk about that*.

8:40–8:50	Slide 10–15	**The Research and Data** Present data from several studies that show coaching is a successful intervention for medical professionals in particular. Highlight Relevant Studies: *JAMA* *Mayo* *Journal of Occupational Psych* 2020

8:50–9:00	Slide 16	**BREAK 10 min**

9:00–9:02	Slide 17	**Bottomline for the Coach Approach** Coaching works! You are seen as an ally by the coachee. You provide safe space. They are heard and seen by you (genuinely). You are working to foster awareness and growth.

9:02–9:06	Slide 18 CC’s	***In this last half hour****, we’re going to do a very basic introduction to two foundational competencies of a professional coach:* - **Embodies a Coaching Mindset and** - **Listens Actively** Slide 19 **Quick review of the 8 CC’s (1 min)** Slide 19 **Coaching Mindset (2 min)**
	Slide 19 Mindset	Briefly review what this is: open, curious, flexible, other-centered, and empathetic! **Open** to what shows up, to being surprised, even amazed. **Curious** - we step into NOT knowing - we don’t know what is best for this person, but they do! **Flexible** - We are agile, we move with the client/learner rather than leading where we think they should go. **Other-Centered** - Our focus is on that person, what’s truly important to them and their fulfillment. **Empathetic** - We bring our hearts as well as body and mind to session. Our wholeness. Empathy can spring from the humility of not knowing and being with. *This is a mindset that asks us to broaden our range. Where most of us have become great problem-solvers in life, this asks us to not default to problem-solving but to be at choice - is this a good moment to problem-solve or to get curious and draw out this person’s own answers?* *So, we ask you the question (rhetorically*): What do you need to let go of to embrace this mindset? *Thought experiment or inquiry for you to take with you until we meet again*.

9:06–9:08	Slide 20 Just Listen (in pairs) and debrief	**Active Listening** *The coaching mindset builds trust and safety pretty quickly and allows clients or learners to be more vulnerable which leads to more impactful and on-target change. HOW we listen also creates trust and safety*. *Active listening is a rich and nuanced competency in which we practice different levels of listening: “listening for” and reflecting the essence of what was said and what was NOT said, including the energy and/or emotion*. *We will do a deeper dive in this competency in future sessions*. *For now, we are going to put a toe in the active listening waters, by practicing JUST listening*. **Why do we practice listening?** BECAUSE active listening is harder than we think! *We all get distracted. Lunch, formulating a response, judging something they said, comparing, etc. I want to say what I want to say… and often we’ve been trained to listen in order to problem solve or to offer solutions / our own insights vs. evoking someone else’s*.

9:08–9:10	Slide 20	**Activity: Just Listen**. *In this activity, we want you to practice listening differently–to JUST LISTEN. You will not be looking for an answer or solution or even to connect with a similar story. You are simply sitting with another person and JUST LISTENING. Just listen and hold this person as creative, resourceful, and whole. There is nothing to compare or fix or judge. You get to JUST LISTEN*. **Set up/Instructions:** Pairs will go into a corner to chat. Speaker will have two minutes to share a stand-out moment in their life. It could be simple or more exotic, recent or long ago; eg, walking to the bus stop with your child and holding hands yesterday, or kayaking in a fjord years ago. Listener will just listen. Switch and repeat -Two minutes. Be sure to time yourselves! We’ll also shout out. **Pair debrief** (2 min) Afterwards, you have two minutes to share with each other what it was like to just listen and to just be listened to. **One minute each**.

9:10–9:17		**JUST Listen activity including pair debrief**

9:17–9:22		**Large Group Debrief** (3+ min) *What is it like to let go, to be present, and to JUST LISTEN?* *What did you notice? No wrong answers here!* *What was easy? Hard?* **Bottomline:** *Just listening takes practice and can be very liberating for both the listener and speaker. It builds trust and safety, allows for more vulnerability, provides the opportunity to go deeper, finds new awareness, and can make more impactful change*.

9:22–9:30	Slide 21	**Application:** *Practice embodying a coaching mindset (holding unconditional positive regard, being curious) and just listening. Notice what you notice, and notice what comes up. Looking at the core beliefs can help*. **IF TIME** (5 min) **Discuss as a big group:** How, where, with whom will you do this? **Summary:** What’s your biggest **take away today**? Write it down and then ask a few to share.

## Appendix D2 Session 1: Foundations of the Coach Approach – Handout

### What are the core principles/assumptions of coaching?

- Naturally Creative Resourceful and Whole (NCRW)
- Coachees are expert in their own lives
- Address the coachee’s whole life
- Unconditional Positive Regard (UPR)
- Beginner’s Mind/curiosity reigns
- The power is granted to the relationship
- Deepening the learning, forwarding the action (evoking transformation and change)
- Trust and safety/confidentiality are the bedrocks
- Trust and safety are the bedrock of all successful coaching relationships - confidentiality must be established and honored
- The relationship is co-created and voluntary
- While most coaching engagements take place consistently over time, a single “coachlike” conversation can be life-changing

### What are the core competencies of a professional coach?

- Demonstrates ethical practice
- Embodies a coaching mindset
- Establishes and maintains agreements
- Cultivates trust and safety
- Maintains presence
- Listens actively
- Evokes awareness
- Facilitates client growth

### Why do people typically seek out the services of a coach?

- Transitions - work and life
- Burnout, stress
- Habit change, behavior change
- Enhance leadership/management skills
- Increase confidence
- Goal attainment
- Define values
- Envision the future
- Increase awareness and resilience
- Team building
- Relationship building

### Coaching is based on the framework of many domains

- Positive psychology
- Behavioral psychology
- Motivational interviewing
- Adult development
- Mindfulness

### Coach vs. Other Roles

**Table:**

Role	Purpose	Approach	Who Directs the Process
**Coach**	To support personal or professional discovery and development	Nonjudgemental conversation Provocative and powerful questioning Facilitation of conversation	Coachee
**Mentor**	To share mentor’s own experience and knowledge to benefit the mentee	Guidance Sharing of mentor’s experiences and opinions	Mentor or mentee
**Advisor**	To provide expert insight and recommendations	Provision of information and expert knowledge Problem solving	Advisor or advisee Targeted issue or question
**Counselor**	To facilitate healing or forward movement from past experiences	Therapeutic and behavioral interventions	Counselor

Adapted from Joe Donaldson, 2013

## Appendix E1 Session 2: Introduction to Core Coaching Skills – Facilitator Guide

**Learning Objectives:**

By the end of the session, you will be able to:

1. Define professional coaching curiosity
2. Describe what makes a question powerful and use powerful questions in a coaching context
3. Identify the 8 core competencies of professional coaching
4. Understand the basic structure of a coaching session and how to apply it to a coaching relationship

**Table:**

Time	Slide	Notes/Presenter Talking Points
10:00–10:08	Slide 1 & 2	Today’s agenda/learning objectives (3 min)
10:08–10:13	Slide 3	(5 mins) *In Class #1 we gave a high-level overview of coaching and the research related to the effectiveness of coaching, particularly in the field of medicine*. *We also dipped our toes into two coaching Core Competencies: the coaching mindset and active listening*. *Let’s get grounded in content by giving a brief review of what we covered:* Mindset - 2 core aspects: Hold as creative, resourceful and whole with unconditional positive regard. Being curious. Step into the ‘not knowing’ and be okay with it. *The mindset is also about being open, flexible and client/learner-centered. What’s important to them and ultimately, their sense of fulfillment*. *Last time, we gave you two homework assignments:* Practice embodying a coaching mindset. Practice just listening (focus of homework assignment was here). *Share what you learned, what was hard, what came up, what you noticed, etc*. If needed: Just listening (a component of active listening, one of the CC’s) is a powerful form of effective communication. A coach approach will include that. Mindset is about expanding range and being at choice…being flexible, open, comfortable in not knowing, AND CURIOUS! A choice = what is most needed in this situation? Telling or being curious? Pull out a thread from their comments related to curiosity. *Your curiosity will open your mind to questions that will help your learner see new possibilities and awaken to new awareness, actions, and impact!* Curiosity is foundational in coaching. Let’s turn our attention there now.
10:13–10:15	No slide	**Distinctions about Curiosity** *In coaching, we have a very distinct flavor/essence of curiosity:* *Curiosity in service to the person in front of you*. *It’s NOT your curiosity about their story and getting more details about the story; it’s a curiosity that goes deeper into what’s truly important here, what values are here, what do they really want, what’s getting in the way, etc*. Example: If I’m listening to someone, and they are talking about a recent trip to Hawaii and what an amazing experience it was, from a coaching perspective, I ask questions like, “What was amazing about it? What was the experience like?” and not things like, “Where in Hawaii did you go? What did you do? Did you do the road to Hana?”
10:15–10:20	Slide 4	**Activity: Coaching Curiosity** Pairs 2 minutes each - track time! Take a picture of the slide. Practice asking questions from this coaching lens of curiosity. Talk about a favorite vacation. Ask whatever questions you want that come up during the two minutes–just be certain that you are asking questions about their experience and not your relating to it in the common way we use to connect. Notice when relational curiosity comes up (it will! we’re human!) and return to coaching curiosity and pull out their experience and what’s important to them.
10:20–10:25	No slide	**Debrief the Feel for Coaching Curiosity Activity** (1 min) *Write down a few observations or things you noticed (both when you shared and when you asked questions). What’s it like to listen with a coaching curiosity (vs. a common way we listen when we’re trying to connect)?* (4 min) *Who wants to share what they wrote? One or two people?* *Now that you’ve experienced coaching curiosity, let’s turn to what makes that curiosity particularly powerful?*
10:25–10:27	Slide 5	(2 mins) **Overview of slide of What Makes a Powerful Question** *Holding in mind what you just talked about in pairs, we’re now going to offer what we believe makes a question powerful*. What makes a question powerful? -short -open ended -how or what (not why) Examples are on the slide. (Will refer to Handout later.)
10:27–10:35		**Powerful Questions DEMO and DEBRIEF** (3 min) DEMO - one minute doing weak questions and then switch and do one minute doing powerful questions - consider a topic about being frustrated or stuck. (3 min) - DEBRIEF - what did you notice? *Note that powerful questions are curious, generative, thought-provoking and NOT leading them to what you think the answer is. Emphasize that they are exploratory!*
10:35–10:50	Slide 6	(15 min total) **Activity - Powerful Questions Practice in Pairs** Just practice asking powerful questions (4 min each) Set up: One person is the coach One person is the coachee/learner Coachee/learner will talk about a place where they are frustrated or feeling stuck (personal or professional scenario). Coach will practice asking powerful questions in the pauses. *Don’t worry about doing this perfectly! You have permission to experiment and ask terrible questions!* *Try to start them with WHAT or HOW*. *The only rule is that you ask only one question at a time–don’t stack. Ask a question and then duct-tape your mouth shut*. Set your timer for 4 minutes. Once 4 minutes is up, coachee will let coach know what one question was most helpful or impactful and why (about one minute each for this part). (3 min) Debrief pairs activity as a big group.
10:50–10:55	Slide 7	**Taking Stock: Where We’re At** Core Competencies - put up slide. We’ve touched on: Coaching mindset Listening actively and the power of silence Curiosity and asking powerful questions, which is a core part of evoking awareness What’s left → *Ethics*: just a light touch; we’re not going to do a deep dive into this; we just want you to be aware that the ICF has a code and you can go to the website or ask us questions outside of class if you have them. Main thing: confidentiality reigns! *Agreements*: what we’ll **do** and how we’ll **be** together both throughout the entire relationship and each individual session; we will cover in depth next session. *Presence*: how we fully “be with” the client; the dance; coach the who not just the what; we will continue our learning around this as we progress through the program together *Facilitating Learning and Growth*: There is usually a new perspective or insight in a session and that will disappear if it’s not taken into the world. We “forward the action” in coaching. “The shelf life of a new insight is extremely short” - Doug Silsbee. Therefore, we need to partner with our clients to integrate what we’re learning and put it into action for continued growth and development/transformation–more to come on how to do this, too! All of these core competencies flow together within a session. And we’re going to deep dive on all of these as we move through the program. For the balance of our time today, we’re going to talk about what a typical coaching session looks like.
10:55–11:02	Slide 8	Structure of a Coaching Session Review structure of a coaching session: Beginning: identify what learner wants to accomplish. Middle: explore and uncover new insight and awareness about learner (both about self and situation). End: take insights into action. Roughly, 10%-80%-10% in terms of time commitment. No matter how long the session is (5 minutes or 50 minutes–doesn’t matter!)--this is the basic structure. Let’s look at key example questions for the structure. Note that these are powerful questions!
	Slide 9	Beginning: what do you want to talk about? what’s on your mind? and what else? what’s important to you about that? what do you want to be different (by the time we’re done talking today)? what’s getting in the way?
	Slide 10	Middle: What do you want? How can I help? If you’re saying yes to this, what are you saying no to? What do you want instead? What’s the real challenge here for you? What do you want it to be like six months from now? What’s this experience like for you? What are you feeling? What do you need to let go of? What are you embracing?
	Slide 11	Ending: take insights into action What are you learning about yourself? *Situation? New awareness? Takeaway? - - all of these are golden! LAND the awareness and then take into life*. How do you want to integrate this moving forward? What are your next steps?
11:02–11:07		**Activity: Experience the Beginning of the Structure -- get a taste** *We’ll do more with the middle and end parts in another session. For now, we want you to experience the power of the questions we typically ask at the beginning of a coaching session to help clarify where we are headed and why. I’m going to ask you to think of something you’d want to be coached on, and then I’ll ask you a series of questions and you’ll write down your answers and notice what you notice*. *Let’s begin. Think of something you’d like coaching on (give some examples for them, like how to get along with a new boss, how to manage all of the workload as of late, how to have hard conversations with the team, etc.). You can also use the same topic from before. I’ll ask you questions, one at a time, and give you time to reflect and answer in between*. ○ What do you want to talk about today? ○ What’s important to you about this topic? ○ What else? ○ What else? ○ What do you want to be different at the end of our conversation? ○ What do you need to consider or resolve to get you there?
11:07–11:10		**Debrief:** What did you notice? Any questions that were particularly helpful for you? And if time, are there any questions about anything from today before we go into integration time?
11:10–11:20	Slide 12	**Pairs Integration Activity: What are you learning so far?** **Processing in groups of 2:** What stands out to you from what we’ve covered in our first two foundational sessions? How will you apply what you’re learning to the coaching relationship?
11:20–11:25	No slide	Debrief in a big group
11:25–11:30	Slide 13	**Application** *Practice making your questions more powerful wherever and whenever you can*. *Keep a list of your five favorite powerful questions on a notecard or sticky note next to your desk or in your pocket*.

## Appendix E2 Session 2: The Coaching Framework – Handout

### 1. The Coaching Mindset

○ We hold our client as creative, resourceful & whole.
  *We recognize that our client has inner resources, insights, and wisdom*.
○ We know that we don’t have to solve the problem for the client.
  *We step into curiosity and not knowing*.

### 2. The Coaching Session Structure

**Table:**

**Beginning**	*What’s on your mind? What else?*
	*What do you want to talk about today?*
	*What’s important to you about that?*
	*What do you want to walk away with today?*
	*What’s getting in the way?*
**Middle**	*What do you want?*
	*What’s getting in the way?*
	*What’s this experience like for you?*
	*If you’re saying yes to this, what are you saying no to?*
	*What do you want instead?*
	*What’s the real challenge here for you?*
	*Where else does this show up?*
	*What are you feeling as you say that?*
	*What do you want it to be like six months from now?*
	*What do you need to let go of?*
	*What are you embracing?*
**End**	*What are you learning about yourself?*
	*How do you want to integrate this moving forward?*
	*What are your next steps?*

### 3. The Coaching Skills

○ Presence
  *the ability to be in the here and now*
○ Active Listening
  *the ability to focus completely on what the client is saying and not saying*
○ Powerful Questions
  *the ability to explore beyond the client’s current way of thinking and feeling to new and expanded ways*

## Appendix F1 Session 3: Deep Dive into Co-Creating Relationships – Facilitator Guide

**Learning Objectives:**

By the end of the session, you will be able to:

1. **Design an Alliance** (DA) with your coachee: Co-create space that is intentional and enhances trust for a coach/coachee relationship.
2. You will learn how to:
  - Build trust and safety
  - DA: Co-create the structure of the relationship
  - DA: Define Primary Focus Areas/Goals

**Table:**

Time	Slide	Notes/Presenter Talking Points
10:00–10:05	Slide 1	(3 mins) Be present exercise.
10:05–10:08	Slide 2	**Foundations Review** Quick Recap of Where We’ve Been: The last two session were about foundational, overarching principles and ideas of coaching, including: An overview of the research on professional coaching and its effectiveness Distinguish between mentoring, coaching, advising, teaching Apply foundational coaching competencies to coaching mindset and listening actively Distinguish “essence of coaching curiosity” from more common relational curiosity. It’s open, in service to the client or learner Define what makes a question powerful and use powerful questions in a coaching context Identify the 8 core competencies of professional coaching Understand the basic structure of a coaching session and how to apply it to a coaching relationship In today’s session, as well as in those remaining after today, we are going to be doing a deeper dive into some of these areas. Today, we are going to do a deeper dive into THE foundational piece of any coaching relationship: creating structure and trust and safety to support a helpful and satisfying relationship. Before we go there, we’re going to a small group activity to warm up our brains and reground us in where we’re at in our learning.
10:08 – 10:18	Slide 3	**GROUND YOUR KNOWLEDGE: RECAP ACTIVITY** In pairs and in a big group (10 min) PAIRS (4–6 min total): Turn to the person next to you: 2–3 mins each to discuss/share where and/or how you’ve used any of the foundational skills/competencies that you’ve learned about so far, the results you achieved, and any lingering questions you have. BIG GROUP (5 mins) What have you experimented with? What did you notice?
10:18 – 10:20	Slide 4	**Today’s Agenda/What You Will Learn: Deep Dive on Co-Creating Relationships** Co-creating a relationship means that you and the learner come **together** in a space that is *intentional* and *enhances trust*. It includes three parts: How to cultivate trust and safety How to structure the relationship How to define Primary Focus Areas and Goals We’re going to begin with cultivating trust and safety–and how to do it–by leaning into our own prior experiences.
10:20 – 10:35	Slide 5 Slide 6	**Cultivating Trust and Safety** *Your Experience of Trust and Safety* Activity: Your Trust Story **Part 1** - Quiet thought and notetaking (2 min) *Take two minutes to jot down some notes about an experience where you felt a high degree of trust and safety in a relationship. It could be past or present, professional or personal*. *What did you notice, what did you observe, and/or what did it feel like when you experienced this high degree of trust and safety?* **Part 2** - Sharing in pairs (3 min each / 6 min total) *Now, in pairs, you will share your story. It doesn’t have to be fully told or perfect; just share what you noticed or observed or felt like when you experienced a relationship of a high degree of trust and safety*. *Share the story*. **Big group debrief 1**. *Thanks for sharing in your groups. Based on your own experience or on what you just heard, what cultivates trust and safety in a relationship?* *All of these are important in a coaching relationship as well. Trust and safety are foundational for impactful effective coaching*. **Big group debrief 2:** *What is more likely to happen for your learner when there is a high degree of trust and safety?* **Bottom Line:** When there is trust and safety, clients can go deeper, be more vulnerable, and have bigger insights leading to new possibilities, new actions and new results for them. Trust and safety set the stage for impactful, even transformational, coaching. It is KEY.
10:35–10:40	Slide 7 Slide 8 Slide 9 Slide 10	**Defining Trust and Safety** *In the coaching approach, we believe that you must build trust and safety from the get-go, from the first moment you meet and begin opening your mouth to speak*. The ICF defines this skill as: Partnering (with the client) to create a safe, supportive environment that allows the client to share freely. Maintaining a relationship of mutual respect and trust. *Let’s break this down. We’ll start by looking at the last sentence of this definition: maintaining a relationship of mutual respect and trust. What do we mean by TRUST in our context?* Trust comes in two forms,^24^ and you need both in a relationship in order for the relationship to be effective over the long-term: Predictive trust (eg, do what you say you will do, show up when you say you will, etc.) Vulnerability-based trust (eg, being able to comfortably and quickly acknowledge, without provocation, mistakes, weaknesses, failures, and need for help, etc.) Let’s get even more specific for the realm of coaching. The ICF delineates six elements as critical to cultivating trust and safety (CTS): To seek to understand the client within their context which may include their identity, environment, experiences, values, and beliefs. To demonstrate respect for the client’s identity, perceptions, style and language and to adapt one’s coaching to the client. To acknowledge and respect the client’s unique talents, insights, and work in the coaching process. To show support, empathy, and concern for the client. To acknowledge and support the client’s expression of feelings, perceptions, concerns, beliefs and suggestions. To demonstrate openness and transparency as a way to display vulnerability and build trust with the client. All of the six are great – but how to put them into practice? We do it from the get-go with: holding a particular **mindset**, **(unconditional positive regard, creative/resourceful/whole)** engaging specific coaching **skills (active listening, curiosity, presence)** co-creating **structure (the Designed Alliance!)** *Let’s start with structure. In coaching, we call the structure The Designed Alliance (DA)*. The power of the Designed Alliance: When done well, effective relationship alliances with others are formed, minimizing miscommunications and aligning expectations. We use the Designed Alliance in coaching, and the DA is generalizable and can be used for building teams, clarifying meeting agendas and building relationships in general, including mentor/coach relationships.
10:40 – 10:55	Slide 11	**The Structure/The Designed Alliance** There are three parts: How we want to BE together so that this is a useful (and enjoyable) relationship. What we are going to DO together: Primary Focus Areas (PFAs)/Goals. The logistics. I think we all get logistics - discussing how often, how long, in person, on zoom, etc. For coaching, typical is two times a month for 30–60 minutes, usually no less than three months. **Demo of DA for a coaching relationship - THE BEING** (5 mins) *Welcome and my Intentions (what I practice to help create a safe and courageous space.)* Naturally resourceful, creative, and whole All emotions are welcome How I’m holding you and this relationship All of you is welcome here – and it’s up to you regarding what you want to share here This is as close as we can get to a judgment-free zone I come at this with deep curiosity and respect Confidentiality Personal pronouns Identity/context that you want to share Communication style Ask Questions: Do you have any questions about anything I’ve said so far? Is there anything I haven’t said that is important for you to have a good experience with me? What do I need to know about you and/or what’s important to you about this experience? **DEMO OVER and then say:** You can do exactly this kind of thing with your learner, or make it even simpler, make it your own, with simply: Make your intentions known. And then ask them what they want/need. This is a quick side note: the Designed Alliance Is useful for so many things! You can use this same process for setting up for a meeting, e.g. This is what’s important for me to cover in this 20-minute meeting. How about you? Spotlight context and identity: Maybe add that we each bring our own identity and lived experience, and our clients bring their own identities and lived experience. Sometimes naming those differences and their impact on the relationship can be HUGE for building trust and safety. You may not say it eloquently, and you may mess up, and that’s okay! PAY ATTENTION TO THE POWER DYNAMIC that might exist. Male/Female White/Color Old/Young US Citizen/Non-Citizen *Sometimes this is new and uncomfortable territory for us to figure this out…do any of you have any examples to share?* I’m open to your feedback. All of you is welcome here. I am on your side. Any questions?
10:55 – 11:00	Slide 12	**DESIGNING YOUR OWN ALLIANCE for the BEING = ACTIVITY Part 1** (3 min) Journal on the following: How do you want to BE with your learner? Consider what you know about CTS What would you include in your Designed Alliance?
11:00 – 11:15	Slide 13	**DESIGNING YOUR OWN ALLIANCE for the BEING = ACTIVITY Part 2** (3 min each / 10 min total) Share your Designed Alliance thoughts and discuss. **LARGE GROUP Debrief**
11:15 – 11:25	Slide 14	**Designing the Alliance for the “DOING” with Your Learner** Define PFAs - Simple ways: What do you want to make sure we work on in the coaching? What outcomes are important to you? If it was a year from now, and we’re having coffee, and you are satisfied with the coaching, what is different in your life? How will you know… …that we are headed in the right direction? …that we are “there?” …again, what’s different? **DEMO and example** Group - notice what you notice about how we become clearer on what the learner wants. Coach: *It’s my intention that this relationship serves you well, so I’d like to spend a few minutes exploring what you’d like to bring to your sessions or to work on together. What’s one thing that you’d like to do here?* Learner: *One thing that’s at the top of my mind is that I want to work on my communication skills. I feel like I get abrasive sometimes when I’m in the clinic, and we’re just super slammed–especially with my colleagues. I want to improve my soft skills with them*. Coach: *What does “soft skill” mean to you?* Learner: *I just want to be more responsive and less reactive - less abrasive!* Coach: *You’ve said “abrasive” a couple of times. What’s that about?* Learner: *Yah, I’ve been recently told that I can be abrasive - that and I am too short and impatient and my frustration impacts the people around me*. Coach: *Yeah, it can be hard to self-manage when you’re stressed. Okay, if it was six months from now and you’ve made some progress (i.e., you are more responsive and less reactive, less abrasive), what’s that look like or sound like?* Learner: *Well, hopefully nobody tells me I’m abrasive again! And I just feel like I’m more part of the team and more balanced*. Coach: *Great, you feel more balanced, more part of the team. And what are you* *doing* *differently?* Learner: *Hmmmmm… I’m more aware when I start to get fast and frustrated*. Coach: *Great, you see it coming, you are more aware. What else are you doing differently?* Learner: *Maybe when I notice myself speeding up, I take a breath and reset myself somehow… IDK, that’s what I’d like to work on, something along those lines. Something to cue me to slow down and soften up. I’d love to know how you handle it when you just want to bark at people*. C*oach: Okay, yes, I can share that too. And if we make good progress here:* *You’ll be more aware of the habit or dynamic* *You’ll have a tool or a practice to help deal with the intense moments* *You’ll ultimately be less likely to react abrasively* *Your relationships will feel better*. *It could go on, eg*, *I’m just curious - what else would help relationships feel better? What’s important to you about all of this?* **Big Group Deconstruct:** What did you notice helped us define more clearly what the learner hopes to get out of the coaching relationship? What they want: Define the key word/s they use: “soft skills” Where they are now: Explore: “abrasive” “Metrics” for knowing they’ve been successful: What’s different when it’s going better? What are they doing differently? Adding some reflection and acknowledgment of the challenge and/or of their humanness goes a long way! Questions?
11:25 – 11:27	Slide 15	Summary So, now you have the nuts and bolts of creating trust and safety down. Let’s summarize and discuss how this connects to where we’re going next. We’ve discussed Mindset which is so important for creating trust and safety: Honoring the person - creative/resourceful/whole, unconditional positive regard. Today, we went over the Designed Alliance, the structure that supports trust and safety. In next classes, we will take a deeper dive into skills of Effective Communication.
11:27 – 11:30	Slide 16	Application and Close Out Between this session and next: Practice elements of Designed Alliance with a learner or for a meeting, just somewhere in life.

## Appendix G1 Session 4: Deep Dive into the Skills of Effective Communication – Facilitator Guide

**Learning Objectives:**

By the end of the session, you will be able to:

1. Define effective communication (in a coaching approach)
2. Listen “beyond the words,” and explain why it matters to do so
3. Articulate the importance of direct communication, identify how it shows up in a coaching relationship, and use it appropriately in your own interactions with coachees

**Table:**

Time	Slide	Notes/Presenter Talking Points
8:00–8:05	Slide 1	(3 mins) Introduction
8:05–8:06	Slide 2	**Learning Objectives - Effective Communication Listening and talking**. **How are you listening and how are you talking?** Listening: Making distinctions between levels of listening Direct communication
8:06–8:10	Slide 3	**Recap from last time** Doing: Designed Alliance (engagement) as a structure to support trust and safety Being: Trust and safety--what it is, etc. How did that go (or not)? Lingering questions? *From the homework:* Work on trust and safety (element you chose). Practice elements of Designed Alliance with coachee or for a meeting, just somewhere in life.
8:10–8:16	No slide	**Today** is about effective communication–another key skill in coaching! A key aspect of effective communication is making room for the other person. At a very basic level, it’s about: Listening and talking. How are you listening and how are you talking? We introduced a bit about each of these when we talked a bit about “just listening” in Session #1 and when we covered powerful questions in Session #2. Now, we’re going to do a deeper dive into those. **Activity: Listening Actively** Let’s start today with Listening Actively and jump into an activity. The stem: something that someone is really struggling with. Instructions: Two volunteers will pretend we are in a coaching relationship. The coach will ask the learner what they would like coaching on today. The learner will share a short story about something going on with them for two minutes. Just listen as you normally would in any conversation. That’s all! **Debrief** Individual reflection from audience members: What did you notice your brain doing/thinking as a general listener? Put your scientist hat on, what are you observing in yourself? Jot it down. (20–30 seconds) Be honest! It might be that you are distracted, wondering about lunch, thinking about a patient, thinking about how the issue relates to you, should I pick up milk on the way home? :) Group Debrief: Let’s hear from a few - what was going on for you as a listener? No right answer and be honest!
8:16–8:19	Slide 4 Slide 5	**Teaching: 3 levels of listening** There are 3 levels of listening we teach in coaching. **Level One: Internal**. You were probably mostly all in what we call Level One listening, and this is where we normally hang out. We listen to the words of the other person, but our attention is on what it means to us personally. For example, while listening to a learner who is worried about time management, we may think about our own struggles and get distracted by those thoughts or begin by offering our tips before we truly understand what time management means to our learner and where it gets difficult for them or what’s important to them. It’s a lot about you. **Listening through your filters**. I’m focused on my own thoughts, opinions, feelings. On what’s wrong and how I’d fix it, the advice I’d like to give. On what I know or think I know and how what they’re saying relates to me and my situation. This is normal! We all do this because we are all humans! And we need to sometimes be at Level one! **Teach Level Two: Focused listening - focused completely on the OTHER person**. Focused entirely on the speaker. I’ve let go of a lot of my internal dialogue about me. I’m noticing the words and how the words are spoken. Very focused on WHAT they are saying only, trying to understand the words (and **not** focused a whole lot on any emotions, facial expressions, body language, etc. )
8:19–8:21	No slide/ take slide down for a minute	**Activity: Level Two Listening (2 min)** *Volunteer learner to repeat the story but don’t say it exactly the same. Keep it genuine and real*. Listen again to the same set up/story. This time listen at level TWO, entirely focused on what the person is saying. Notice your internal dialogue, and set aside these distractions. After: Notice what’s different and jot it down.
8:21–8:25	Slide 6	**Teach Level Three: Global/Like a radio field or satellite dish**. At level three, we keep setting down our distractions and we are listening to the words AND we start to notice the nonverbals: pacing, energy changes, signs of emotion, nonverbals, what’s not being said, etc. There can be a sense of receiving vs. trying really hard to hear it all. Some liken it to being a satellite dish - open, receiving. Noticing the unspoken and EVERYTHING ELSE. Some call this whole body listening with mind, heart, gut. **Activity: Level Three Listening** Listen again to the same set up/story. This time listen at level THREE. Continue to notice your internal dialogue, and set aside these distractions, AND become a satellite dish, receiving more than super-focused. **What do you notice? What’s different? What are the nonverbals? Jot it down**.
8:25–8:30	Slide 7	**Debrief - Listening Actively** What did you notice is different about listening at the different levels? What’s the impact of listening at different levels? Shine a light on: We listen with more focus, more deeply at Level 2 We will notice more nuance and emotion at Level 3, and you might notice you have more curiosity and feel more connection there. *How might you use this in your own coaching relationships?* Bottomline: We often listen at Level 1, which is okay–sometimes we need to just be gathering information and trying to make sense of the situation–but when working with a learner, we may miss a lot of valuable information/intelligence and opportunity to tap into the learner’s own insight. When we listen beyond the words, we have more data to understand the situation and our learner. Different questions arise from the different ways we listen. When it’s not a transactional problem and solution, we need to listen more deeply and be more curious about what’s making this a problem for this smart person and what’s truly important to them. We want you to have these distinctions so you can choose which level is most useful to this moment with this learner. Next, we turn to talking about effective communication in terms of direct communication and its relationship to coaching/mentoring.
8:30–8:40	No slide Slide 8 Slide 9 Slide 10	**Effective Communication: Direct Communication** CHAT: What comes to mind when you hear “direct communication?” Shout it out. Frank, clarity, forthright, can be harsh, lacking warmth, not relational. In coaching, direct communication means being both **clear and succinct** in service to your learner. It is about thinking before you speak, being concise and precise in your language. It is about NOT A LOT of extra verbiage–just enough to get your question out and/or your point across. Why is direct communication so important in a coach approach? Because the less succinct or clear you are, the more YOU talk: The more leading you become. You begin making meaning for the learner, your interpretation becomes part of the story. It takes you out of a coaching-approach and into the realm of teaching or telling or consulting. The more you talk, the less room there is for your learner’s thoughts and insights to emerge. Maybe give a quick and overly silly example here: Sharon, what lights you up? VERSUS Sharon, what lights you up, you know, as a physician…because a lot of the research on wellbeing shows that if you can figure out what excites you at work, you can try and do more of that and that will help you feel more fulfilled and less burned out…but everyone feels differently…so, I mean, what do you like most about work, or what do you wish you had more of at work to feel less pressured and more at peace? Can you hear the difference between the two? Two main ways direct communication shows up in coaching/mentoring: It’s clear and succinct in your questions. it’s clear and succinct in your reflections. **Remember, powerful questions** are SHORT Q’s (5–7 words) that usually start with What or How. ○ Not a lot of preamble or post-amble ○ Not a lot of explaining your question or re-wording your question - ask one POWERFUL question and then STOP and create the space for your learner to reflect and answer. ○ Examples are things like: What else? How does that impact you? What do you really want? **We are also succinct in our reflections**. What do we mean by reflections here? When providing a reflection, we help our learners see themselves more clearly **and** we make sure we are understanding them. We are reflecting *the essence* of what’s being said/what we think we are hearing in service to the learner. Not a summary, but what seems most important. Essence of the words Essence of the energy Essence of the emotion When you reflect the essence, you shift out of pure Mentor mode (i.e., telling/advising) and into coach approach mode. Why? To check your understanding AND more importantly, to “hold up the mirror” for the learner so they can see themselves and what they want more clearly. We do this because we don’t always recognize what we’ve said or how we’ve said it, and it can be powerful to hear our own words or energy reflected back to us. It’s also helpful for coaches to be sure that they are tracking correctly with the learner (but this is less of the emphasis here). Example: Learner, what would you like coaching on today? Speaker gives a concise reflection on the essence. Follow up example where the speaker gives a rambling reflection on the essence. **Summary:** Direct communication (asking and reflecting in this way) is a muscle you have to build, and it may be challenging if, as the coach, you are used to “telling or teaching.” Your default may want to be to provide an answer or go right to a solution, when the more powerful, appropriate, and coachlike response may be to ask a question or make a reflective observation.
8:40–9:10	Slide 11	**Putting It All Together** (30min) Coach in pairs (12 min each). Keep in mind what we’ve learned today around effective communication: powerful questions, levels of listening, offering reflections, and holding silence. Topic (if they don’t have one) - pick a mild to moderate challenge that you are experiencing in your work or personal life right now.
9:10–9:20		**Debrief** How did it go? What are you learning? What are you still wondering about?
9:20–9:30	Slide 12	**Wrap up** We have two sessions left! What are you still wondering about? Put in chat. **Practice succinct talking**. **Practice listening more deeply - 2 levels of listening** anywhere in life!

## Appendix H1 Session 5: Deep Dive into Evoking Awareness – Facilitator Guide

**Learning Objectives** :

By the end of the session, you will be able to:

1. Define the importance of values in the process of evoking new awareness
2. Work with your learners to define their most important values, their True North
3. Manage “what gets in the way” of living fully by those values

**Table:**

Time	Slide	Notes/Presenter Talking Points
10–10:05	Slide 1	(3 mins) Get present in the moment.
10:05–10:09	Slide 2	**Recap from last time** **Effective Communication -** Levels of Listening and Direct Communication How did the homework go? Practice succinct talking Practice listening more deeply
10:09–10:10	Slide 3	**Learning Objectives** Evoking new awareness is what you are aiming for in each session and meeting because new insights lead to action and transformation. Strategies for evoking awareness: True North is what you are evoking and listening for when talking with your learners–the insights and new awarenesses that are emerging and that can have a transformational impact on growth and change. Ways of working with the learner to find their True North (values) Noticing and managing “what gets in the way” of living fully by our values
10:10–10:20	Slide 4	**Evoking Awareness** Evoking awareness is all about opening new perspectives and generating new insights that, when acted upon, can bring new results and powerful change in one’s life or work. How do we do this? In simplified terms, Evoking Awareness is about the interplay of two main pieces: Helping your learners locate and align with their True North Helping them identify and get curious about what gets in the way of what’s important to them It’s all about helping them evoke new awareness, new insight, in these two areas. **Locating the True North:** What is True North? How do you locate yours? It’s what’s important to them, what they value right now. And it’s their vision and desires for the future. It’s what aligns with their strengths and passions. When we live in alignment with our values, we live a life that is fulfilled. Today we’ll focus on values as part of the True North we’re interested in. Let’s start with an activity. **Activity - True North via Values** You may or may not do value clarification with your learners, but it can be very helpful, so we’ll give you a tool or two today. Let’s start with this: Values List Activity Write down your top five values (1 min) - can include whatever comes to mind. Give learners the values list. Take a look through this list. Is there anything on this list that you want to add or replace a value with? (1–2 min) [Appendix H3. Session 5: Individual Core Values List] *Maybe discuss values briefly here; what are they?* Future Self-Visualization Activity (8–9 min) Another way to look at values is to do a short visualization. Again, you may or may not do this with learners, but we wanted to give you an experience of it for yourself. You may find it useful for yourself, and then you can decide re: coaching. As with any visualization, there is no right way or wrong way to experience this. Some folks see lots, some sense things but don’t see them, others have nothing at all appear, and that is FINE. We are not looking for a particular experience, just giving the opportunity to play in the sandbox of your mind, potentially accessing info we don’t normally tap into in our day to day. Invite you to have an open mind to this experiment. *Short visualization example: Five years from now, things are going well, you are feeling more satisfied, enlivened, etc. What is different? What are you experiencing more of?* **Say at the end of the visual:** Take a few notes about what you saw or sensed or noticed in the future. Given that, are there any values floating up that you want to add to your list? **Group Debrief (2 mins)** What do you make of this? How might visualizing be helpful?
10:23-ish	Slide 5 Slide 6	**Connect the Dots with Coaching** Take a minute and reflect on the following questions (they are all asking the same thing in a different way): How do your values connect to being a coach? Why do you coach? What values are you honoring by being a coach? Let’s hear from a couple of people. How does it feel to acknowledge this? What does it feel like to have these new insights or be reminded of what’s most important to you? It usually feels good! **Acknowledging and living in alignment with our values is an antidote to so much of the burnout, stress, feeling of powerlessness, etc**. So, we gave you these methods, but how might you actually apply it in a coaching situation? **How to work with values (in a coaching session): The Coach Approach** All of the things we’re about to mention are traditionally done at one of our first significant meetings with a coachee but could really be done at any point along the way. Values come up A LOT in coaching! **Listen for and ask about:** what the learner wants, desires, values, envisions for the future, passions, energy/resonance (animation, tone, pitch, pacing, etc.). **Or complete a value activity** - the values list and/or short visual to take them out of all the mental business and stress of the moment and into possibility. You can do a simpler form of **stepping into a preferred future** with learners, like we did earlier. Key points: Getting clear on values is really KEY because they guide so much decision-making in our lives. Honoring/dishonoring creates resonance/dissonance. Again and again, we return to our values–to what’s most important–to help guide us toward new insights and different ways of considering and doing things in our lives, ways that are more resonant and fulfilling and hopeful.
10:30–10:38	Slide 7	**What Gets in the Way** of living in their True North or what they are trying to do and/or what we want? What causes us to betray our values and/or ignore our True North? **External systems** and demands–for sure. It can be hard to see how to get out from under the paperwork, requests, the schedule, on-going demands. Your learners MIGHT have some control or influence here, and more often, our control is with our **internal systems**. We sometimes refer to these internal systems and demands as our inner critic, habit nature, saboteur voice, etc. **Today, as we learn about what gets in the way, we’ll focus on inner critics**. We all have an inner critic that says things like “you’re not good enough at this, you should just stop, or you don’t work hard enough like everyone else,” etc! Whenever we are learning something new or making a change, the inner critic gets louder because it wants us to stay safe--which means staying the same. In learning a coach approach, you are learning something new, maybe for the first time you are coaching, so what’s the inner critic saying to you? What’s it trying to tell you? Take a moment to write down what your critic says to you. Also, does your critic have a name? If so, what is it? If you are willing, share your thoughts. Answers might be things like: I’m not really experienced enough to coach I am not trained as a coach and don’t know how to do it; So, we all have them and your learners will have them, too. Shining a light on them immediately helps to take away some of their power, so you can get back to the business of living by your values/True North more fully. How do you shine the light on the inner critic? How do you work with learners when a learner’s inner critic is showing up and getting in the way? Shine the light = curious, light, judgment-free **Name it and partner**. Make what’s invisible visible AND partner (don’t just assume you know but actually name and ask about it): “It sounds like… what do you notice?” (Listen for Coulda, Oughta, Woulda, Shoulda). **How is it serving you?** What would happen if you didn’t (fill in blank, for example, do it all perfectly)? What does your inner critic want FOR you? *This is where compassion often shows up, and it is a game changer. As this all softens, you can access more fully the True North/what you really want*. **How do you want it to be?** What do you prefer? What does that look like? How can you practice that?
10:38–10:43	Slide 7 (again) Remind them of the UWCPC handout as a reference for later.	**ACTIVITY Journaling** - **Exploring Your Inner Critic** (5 min) Think about the inner critic voices you identified earlier today. If you didn’t identify one earlier, take a moment to do that now by writing down a typical phrase you hear your inner critic say a lot. Give the Inner Critic a name–don’t overthink it! *Walk them through these questions as they journal*. Name it. Explore it: What does it say to you? When does it show up? What is the impact on you? How is it serving? What’s it doing for you? What do you prefer? How would you prefer to do what it’s trying to do for you? What would you tell yourself? What might you do differently? What would be the impact? Become aware of it. Name it. Explore how to be with it. Shining the light of awareness on the learners helps them think and get quieter.
10:43–11:00	Slide 8	**DEMO: The Inner Critic** They were asked to “bring a current situation where your inner critic might be involved.” **Coach** 12 minutes **Debrief** 5 minutes What do you notice, what stands out for you? How is a coaching session different from what you might have experienced in a more traditional mentoring session? We all have an inner critic and giving space to look at it and what is more true or aspirational is very helpful! The inner critic tries to protect and help, and so giving it a little love or compassion also helps it loosen its grip. And adding VALUES adds power.
11:00–11:15	Slide 7 again	**ONE person Coach for 12 minutes each. Show them slide 7 again if they want it/take a pic of it**. Get into pairs and coach around your inner critic. Decide who is coach first, who is client first. Client share about your inner critic. Client: consider what questions or reflections were most useful and be ready to debrief. Coach: Use the questions from slide 7 AND move into your curiosity, levels of listening, your own questions. Be sure to explore “What do you prefer?” before finishing.
11:15–11:25		Debrief What questions or reflections were most useful?
11:25–11:30	Slide 9	**Homework and Wrap Up**

## Appendix H2 Session 5: Your Inner Critic – Handout

**Notice and Name**. Be the Observer. Get to Know your Inner Critic.

- When does it show up?
- How does it show up? Explore with Thoughts, Feelings, Action, Results: TFAR:
  ○ What does it sound like? (Thoughts or narrative)
  ○ What does it feel like in your body? (Feeling: sensation/emotion)
  ○ What action or inaction does it inspire? (Action)
  ○ What is the impact for you and others? (Results)
- If it were personified, what would it look like? (human, critter, other?)
- What would you name your inner critic?

**Serving:** How is it trying to serve you?

- What is it taking care of?
- What does it want for you?
- Extend compassion or kindness or understanding?

**Practice what you Prefer:**

- How do you prefer to take care of what the inner critic is trying to do for you?
- How do you prefer to talk to yourself? What’s more honoring?
- How do you prefer to be with yourself?
- If you “try that on,” what does it feel like?
- How can you practice this?

**For further exploration if you like:**

- What’s the early warning sign that the inner critic is on the premises? The sooner you notice, the easier it will be to acknowledge it and shift from it.
- How do you want to be with it when you notice? For example, what would you say to it? You could say one or more of these: *I see you. There you are. Thanks for sharing. I see you and know you want to help but let’s do it differently. Oh, Perfection Pat is here!*
- After acknowledging your inner critic, you can shift to what you prefer - or take a breath or look out the window to reset your attention to where you want it.

## Appendix H3 Session 5: Individual Core Values List

**Table:**

Accountability	Curiosity	Honesty	Patience	Service
Achievement	Dignity	Hope	Peace	Simplicity
Adventure	Equality	Humility	Perseverance	Spirituality
Authenticity	Faith	Humor	Personal fulfillment	Teamwork
Balance	Family	Independence	Power	Time
Belonging	Financial stability	Integrity	Purpose	Tradition
Career	Forgiveness	Joy	Recognition	Travel
Commitment	Friendship	Justice	Reliability	Truth
Community	Generosity	Kindness	Respect	Understanding
Connection	Grace	Knowledge	Responsibility	Uniqueness
Courage	Gratitude	Leadership	Self-respect	Vulnerability
Creativity	Health	Love		
Additional Values:				

Abbreviated version adapted from Brene Brown content on https://brenebrown.com/resources/dare-to-lead-list-of-values/

## Appendix I1 Session 6: Deep Dive into Facilitating Learning and Growth – Facilitator Guide

**Learning Objectives:**

By the end of the session, you will be able to:

1. Integrate insight into action
2. Highlight the importance of Presence
3. Wrap Up

**Table:**

Time	Slide	Notes/Presenter Talking Points
8:00–8:05	Slide 1	3 mins **Being present**
8:05–8:10	Slide 2	**Recap of last time and of homework** Brief: evoking awareness, values and the inner critic – RECAP: Evoking new awareness is what you are aiming for in each session and meeting because new insights lead to action and transformation. Strategies for evoking awareness: True North is what you are evoking and listening for when talking with your learner–the insights and new awarenesses that are emerging and that can have a transformational impact on growth and change). Ways of working with learner to find the True North (values) Noticing and managing “what gets in the way” of living fully by our values What gets in the way is often the gremlin voice… Did anyone do any practice or have any notice around values and the inner critic?
8:10–8:11	Slide 3	**Learning Objectives** Recap of all five sessions - everything we’ve learned is leading to taking new actions for new results. Focus: Integrating insight into action Highlight the importance of presence Wrap up - coaching
8:11–8:15	Slides 4 – 8	**RECAP ALL 5 SESSIONS** **Session #1- the overview Session #2 - the basics** **Session #3 - co creating relationships Session #4 - effective communication Session #5 - evoking awareness** Recap of all five sessions - everything we’ve learned is leading to taking new actions for new results.
8:15–8:22	Slide 9	**From Insight to Action - Lecturette (2 min) and Role Play (5 min)** Coaching is all about deepening the learning and forwarding the action. Insights emerge, and we integrate those insights into action. Action steps help us to “lock in” the learning. That’s fundamental to what makes coaching different from other “learning situations” in one’s life. New awareness feels powerful but those insights have a very short shelf life and will disappear into the ethers unless we put them into action. Toward the end of every coaching session, you must ask: What are you learning about your situation? What are you learning about yourself? THEN ask→ How can you take this forward? That’s how you move from insight into action. Again: deepen the learning and forward the action. **Set Up– ROLE PLAY** Pay attention to what insights are happening and what the coach and learner do together to move insight into action. What are you noticing? **ROLE PLAY:** Coach: What do you want coaching on? Learner: I’m feeling overwhelmed and am having difficulty prioritizing where to focus my efforts for improvement. It feels like I can’t do anything well since starting residency. Coach: So, I hear you say you feel like you can’t do anything well since starting residency. I’m curious - what ARE you doing well? Learner: Huh….hang on….well, I’m showing up for my patients. I’m getting here on time. I’m caring. I know I’m doing my best in the ER. I’m getting most of my charts done on time. I guess I’m doing some things well. Coach: It sure sounds like it. It sounds like you are doing **a lot** well. Where’s the rub? Learner: I don’t acknowledge what I do well. I focus on anything I’m not getting an A+ in. Coach: Aha! Where might it be okay to get a B or B-even? Learner: Nowhere! Ugh, but that just doesn’t work, you know? It’s just not realistic…it never really has been, but I always hold myself to this standard. PAUSE I guess I could allow myself some grace and allow myself to get a B-in some other areas…but I really do want an A with my patients! But I know that if I want to do my best with my patients and get the A in that, some other things are going to have to just fall off a bit, or I’m going to have to be okay with some “B” level work, or I’m going to drive myself nuts and burnout! Coach: What happens if you burn out? Learner: I leave medicine and have no patients to treat! Game over. [With Emphasis]: Ooh that’s sobering. Coach: What just shifted there? Learner: I’m seeing a larger perspective. It’s really not at all about my A’s, my unrealistic expectations of myself, it’s about doing what I need to do to stay healthy and to stay IN. Coach: So, let’s pause here. What are you learning about yourself and your situation as we talk through this? Learner: It’s that I’m doing many things well but I can’t aim for all A+’s in this moment of my life and maybe not ever! It’s just not doing me or others any good. It doesn’t serve, and in fact, it hinders or hurts me. Coach: Okay, so how do you take that insight into life? Learner: Ooh, where the rubber meets the road! Something needs to change. I need to change the way I’m looking at this. I’m going to give myself a break on some administrative things, I’m going to acknowledge all that I am doing, I’m going to say “thanks but no thanks” to some requests or opportunities. Coach: How does it feel to say that? Learner: Good! It may be hard to pull off at times but it’s needed. Coach: Great, what’s the first specific thing you’ll do? Learner: I was asked to join a committee on X, and I’m honestly not that interested but wanted to help. But, having had this conversation, I’m going to gracefully decline. Coach: Anything else? Learner: I think this is a good place to start. Coach: Who or what can support you in this? Learner: I’m hoping you can…and I can also meet with my friends. We talk about this a lot, and I think they are big fans of mine, and I know they will be supportive in my quest to be a B student in some areas. Coach: Yes! It sounds like you have good friends here! What might get in the way for you? Learner: If I start to see myself slipping back into my old A+ compulsion, I will send a text to my friends ASAP and let them know that I’m losing my mind and need their help to get back on track! I’ll also take a deep breath and remind myself that I am only one person who can only do so much.
8:22–8:25	Slide 10	**Actions Debrief - What did you notice? What helped take insight into action?** Notice that actions: Often come right from the learner’s new awareness which makes them customized/different for everyone (and more likely to “stick” and work). Varied. No one “right” action. Doable. Resonant-you want to do it; you might even be looking forward to it. Experiments - what works and is adjustable. Sometimes uncomfortable!!! Outside of comfort zone = Growth. Facilitating growth happens via attaching action to insights. Actions may be things like: specific actions, experiments, non-actions, journaling, a way of being, follow-up research, just letting it marinate, etc.
8:25–8:28	Slide 11	**Review the Elements of Facilitating Client Growth - 4 B’s Handout** Bottomline: the learning - the new awareness/insight Bridge to Action: What’s next for you from here? (the action) Barriers: What might get in the way and how to be with that? Bolsters: What do you need to support that?
8:28–8:35	Slide 12	**Coaching in Pairs Activity - Instruction/Set Up** Reflect: What new insights do you have from this series, and what do you want to do to integrate these insights into practice (in coaching and other situations)? Choose one insight you want to put into action. (1 min) Coach using the 4B’s.
8:35–9:05	Slide 13	**Pairs Coaching** (10 min each, 20 min total) Topic: Go back to explore your insight you chose earlier and coach on integration or next steps or anything else - a place you feel stuck or want to open to creativity or insight. Remind them (see handout): Topic Trust and safety Curiosity reigns – ask powerful questions Listening actively - three levels Presence Evoking awareness - What’s in the way? Acknowledging the inner critic and what motivates values/ True North Facilitating client growth
9:05–9:13	Slide 14 for the quotation Slide 15	Presence and unconditional positive regard. *“I’ve learned that people will forget what you said, people will forget what you did, but people will never forget how you made them feel.”* -Maya Angelou And how a person feels seems to impact everything, from how well they perform tasks to how long they stay in a particular job. As you are working with your learner, we invite you to remember that you are uniquely positioned to impact how they feel about their experience as a resident. You are seeing and hearing them and being witness to this part of their professional journey in a way that no one else does. This way of being with your learner has an impact–don’t underestimate it! In coaching language, we call this “relational presence.” This includes how present you are in the moment and how you are holding the person in front of you. We’ve mentioned holding learners as creative, resourceful, and whole. We also practice seeing our learners through the lens of unconditional positive regard. We will explore what we mean by unconditional positive regard and relational presence through a video. This video is called “look beyond borders.” It’s a 5 min video from Amnesty International. I want you to settle in and notice what you notice. Video Link: https://www.amnesty.org/en/latest/news/2016/05/look-refugees-in-the-eye/
9:13–9:20		Take a moment What did you notice in the video? How does this relational presence or unconditional positive regard relate to the coaching relationship? Bottomline: Being held with unconditional positive regard increases engagement and positive outcomes. And it’s simply a gift. Where else do we experience this?
9:20–9:25		Brief Survey-email link to the participants in class and have them complete it right then and there.
9:25–9:30	Slide 15	**Final Debrief and Questions:** Overall, what’s your biggest takeaway from this entire series? What lingering questions do you have?

## Appendix I2 Session 6: Facilitating Learning and Growth – Handout

### Insight to Action

What are you learning about your situation?

What are you learning about yourself?

How can you take this forward?

### Facilitating Client Growth - The 4 B’s

- Bottomline: The learning, the new awareness/insight
- Bridge to Action: What’s next for you from here? (the action)
- Barriers: What might get in the way and how to be with that?
- Bolsters: What do you need to support that?

### Coaching Steps: Quick Reference

- Topic
- Trust and safety
- Curiosity reigns – ask Powerful Questions
- Listening actively - three levels
- Presence
- Evoking awareness - What’s in the way? Acknowledging the inner critic and what motivates values/ True North
- Facilitating Client Growth

## Appendix J Faculty Pre-Survey

1. I feel ______ helping individual residents through personal struggles.
  - Very comfortable
  - Comfortable
  - Neither comfortable nor uncomfortable
  - Uncomfortable
  - Very uncomfortable
2. I feel ________ guiding individual residents who are struggling with clinical skills.
  - Very comfortable
  - Comfortable
  - Neither comfortable nor uncomfortable
  - Uncomfortable
  - Very uncomfortable
3. I feel ________ working with individual residents who are struggling with professionalism concerns.
  - Very comfortable
  - Comfortable
  - Neither comfortable nor uncomfortable
  - Uncomfortable
  - Very uncomfortable
4. I feel ________ motivating individual residents who are disengaged.
  - Very comfortable
  - Comfortable
  - Neither comfortable nor uncomfortable
  - Uncomfortable
  - Very uncomfortable
5. I feel ________ further challenging individual residents who are already excelling in residency.
  - Very comfortable
  - Comfortable
  - Neither comfortable nor uncomfortable
  - Uncomfortable
  - Very uncomfortable

Comments:

## Appendix K Asynchronous / Phase 1 Survey

1. Approximately how much of the assigned reading in the *Coaching in Medical Education* book were you able to complete?
  - 100%
  - 75%
  - 50%
  - 25%
  - 0%
2. How effective was the book *Coaching in Medical Education* in helping you learn foundational coaching principles?
  - Extremely effective
  - Somewhat effective
  - Neither effective nor ineffective
  - Somewhat ineffective
  - Extremely ineffective
3. Approximately how many of the curated articles were you able to read?
  - 100%
  - 75%
  - 50%
  - 25%
  - 0%
4. How effective were the curated articles in helping you learn foundational coaching principles?
  - Extremely effective
  - Somewhat effective
  - Neither effective nor ineffective
  - Somewhat ineffective
  - Extremely ineffective
5. In what ways was the asynchronous curriculum helpful?
6. In what ways could the asynchronous curriculum be improved?

Comments:

## Appendix L Didactic Sessions / Phase 2 Survey

1. I feel ______ helping individual residents through personal struggles.
  - Very comfortable
  - Comfortable
  - Neither comfortable nor uncomfortable
  - Uncomfortable
  - Very uncomfortable
2. I feel ________ guiding individual residents who are struggling with clinical skills.
  - Very comfortable
  - Comfortable
  - Neither comfortable nor uncomfortable
  - Uncomfortable
  - Very uncomfortable
3. I feel ________ working with individual residents who are struggling with professionalism concerns.
  - Very comfortable
  - Comfortable
  - Neither comfortable nor uncomfortable
  - Uncomfortable
  - Very uncomfortable
4. I feel ________ motivating individual residents who are disengaged.
  - Very comfortable
  - Comfortable
  - Neither comfortable nor uncomfortable
  - Uncomfortable
  - Very uncomfortable
5. I feel ________ further challenging individual residents who are already excelling in residency.
  - Very comfortable
  - Comfortable
  - Neither comfortable nor uncomfortable
  - Uncomfortable
  - Very uncomfortable
6. What is the most useful thing that you’ve learned during this course?
7. What do you still feel challenged by?
8. What are your suggestions for improving our training?
9. What **format** would you prefer future training sessions be held in?
10. How **frequent** should future training sessions be?
11. My experience with resident coaching is ____________.
  - Exceeding expectations
  - Meeting expectations
  - Below expectations
12. Being a resident coach is ____________ my professional satisfaction.
  - Increasing
  - Neutral to
  - Decreasing
13. Resident coaching is taking ____________ than I was anticipating.
  - More time
  - The right amount of time
  - Less time
14. On a scale from 1 to 5, how prepared are you to use a coach approach?
15. On a scale from 1 to 5, was this training worth your time?
16. On a scale from 1 to 5, to what degree will this impact your coaching approach?
17. What unexpected results, if any, did you get from this program?
18. What would you tell a colleague about this program?
19. On a scale of 1 to 5, how confident do you feel with facilitating coaching conversations with residents?

Comments:

## Appendix M Faculty Coaching / Phase 3 Survey

1. My experience with my physician coach ____________.
  - Exceeded expectations
  - Met expectations
  - Was below expectations
2. How did this experience influence your understanding of what a successful coaching relationship looks/feels like?
3. What did you gain from this professional coaching experience (personally or professionally)?
4. What are ways in which this experience could have been more helpful?
5. How did this experience affect your own approach to coaching with residents (and other trainees)?
6. What are some suggestions to optimize this experience in the future?
  How helpful was this experience in providing you with:
7. New insights on how to understand residents’ (and others’) behaviors and motivations
  - Very helpful
  - Helpful
  - Neither helpful nor unhelpful
  - Unhelpful
  - Very unhelpful
8. The ability to discuss uncomfortable topics (areas in need of improvement) with residents constructively:
  - Very helpful
  - Helpful
  - Neither helpful nor unhelpful
  - Unhelpful
  - Very unhelpful
9. Confidence in my ability to coach and support residents:
  - Very helpful
  - Helpful
  - Neither helpful nor unhelpful
  - Unhelpful
  - Very unhelpful
10. New ways to enhance my relationships with my resident coachees:
  - Very helpful
  - Helpful
  - Neither helpful nor unhelpful
  - Unhelpful
  - Very unhelpful
11. Ability to help residents establish, and work towards, key goals:
  - Very helpful
  - Helpful
  - Neither helpful nor unhelpful
  - Unhelpful
  - Very unhelpful

Comments:
