# Supplementary material for: A Longitudinal, Practical Curriculum for Faculty Development as New Coaches in Graduate Medical Education
Source: J Educ Teach Emerg Med. 2025 Jul 31;10(3):C1–C92. doi: 10.21980/J88M08 (PMC12320991; doi:10.21980/J88M08)
Supplement: Supplementary file 1 [file 10-3-C1-SuppD3.pptx]

## Slide 1
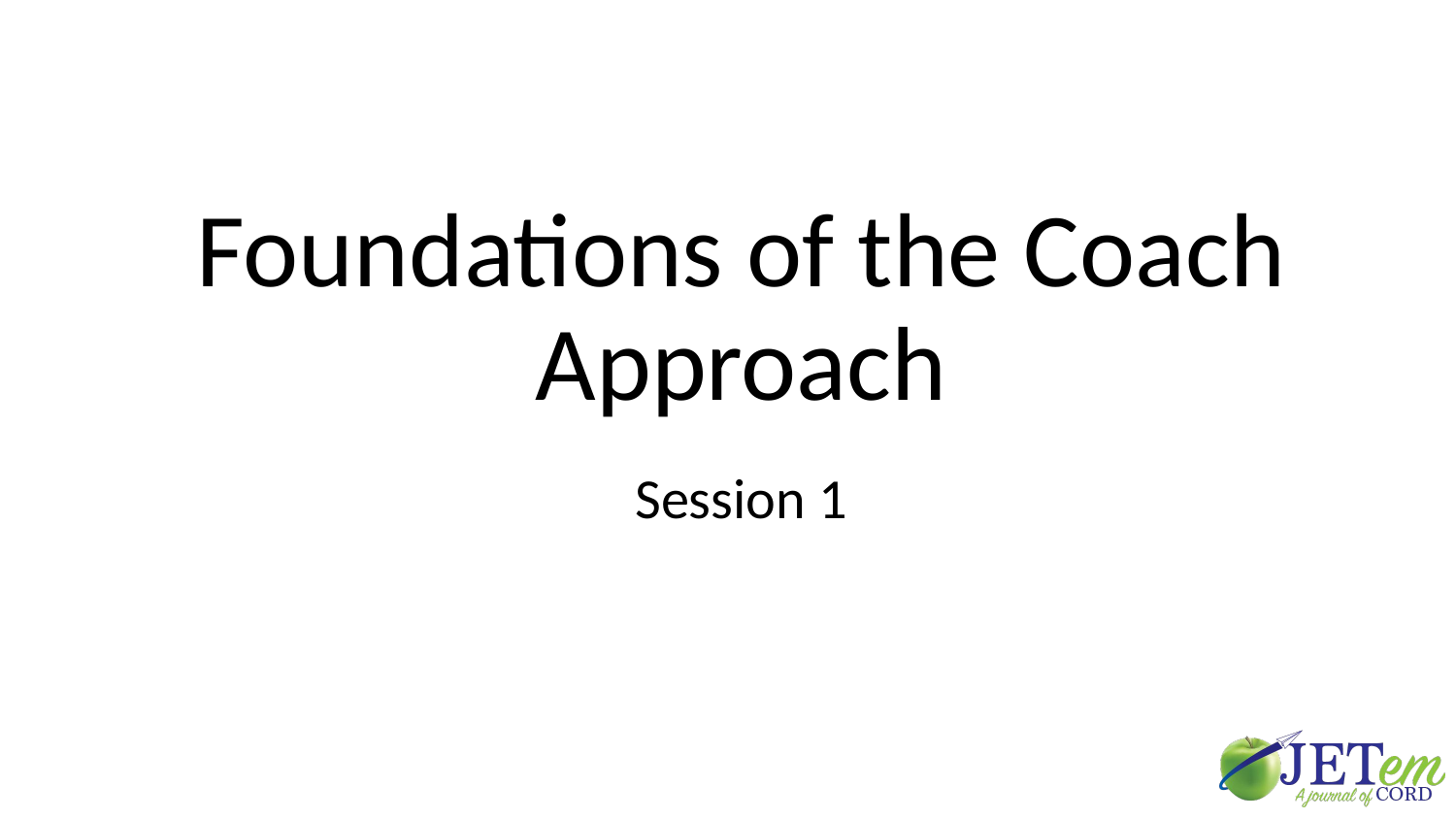

# Foundations of the Coach Approach
Session 1

## Slide 2
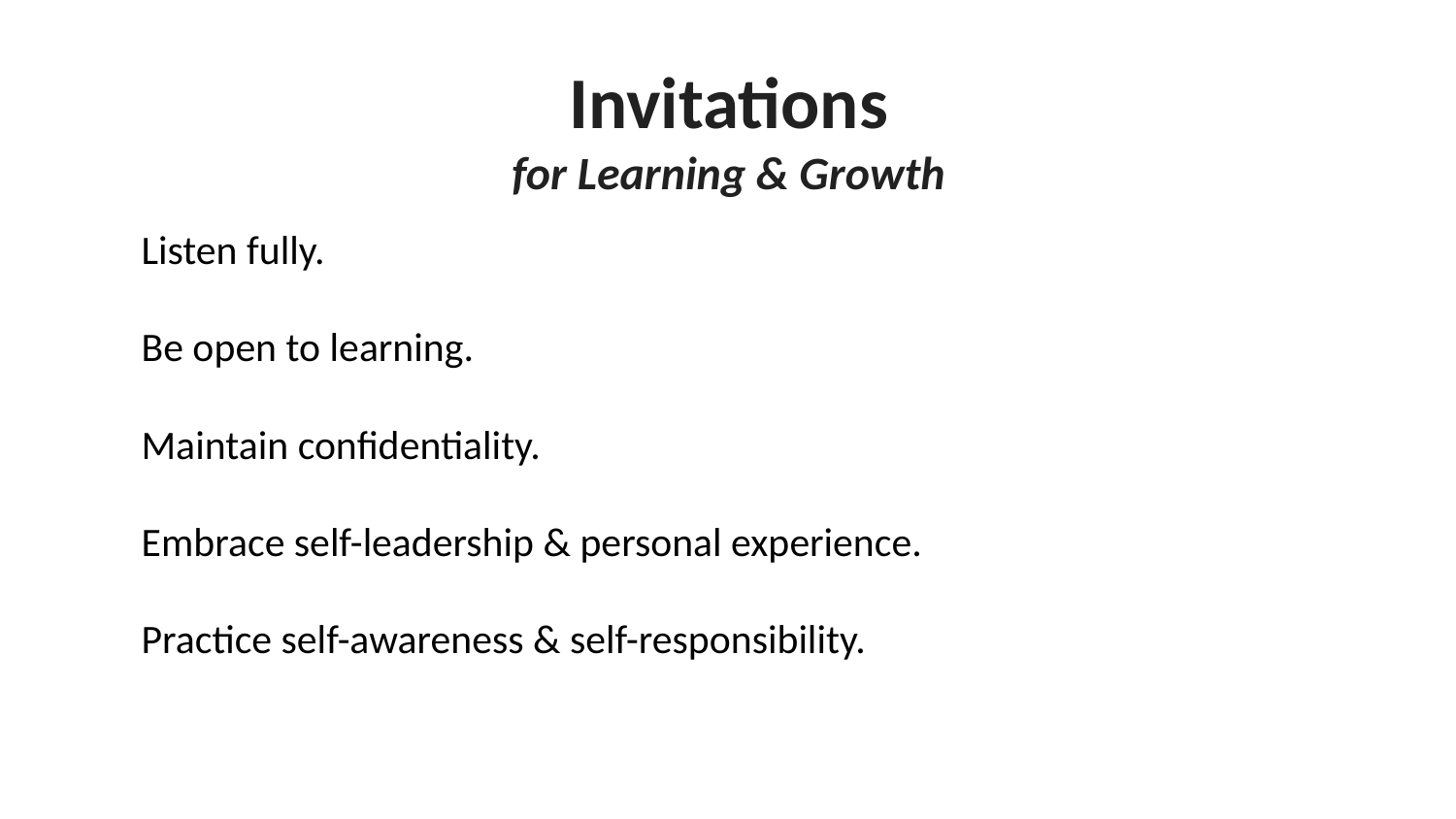

# Invitations
for Learning & Growth
Listen fully.
Be open to learning.
Maintain confidentiality.
Embrace self-leadership & personal experience.
Practice self-awareness & self-responsibility.

## Slide 3
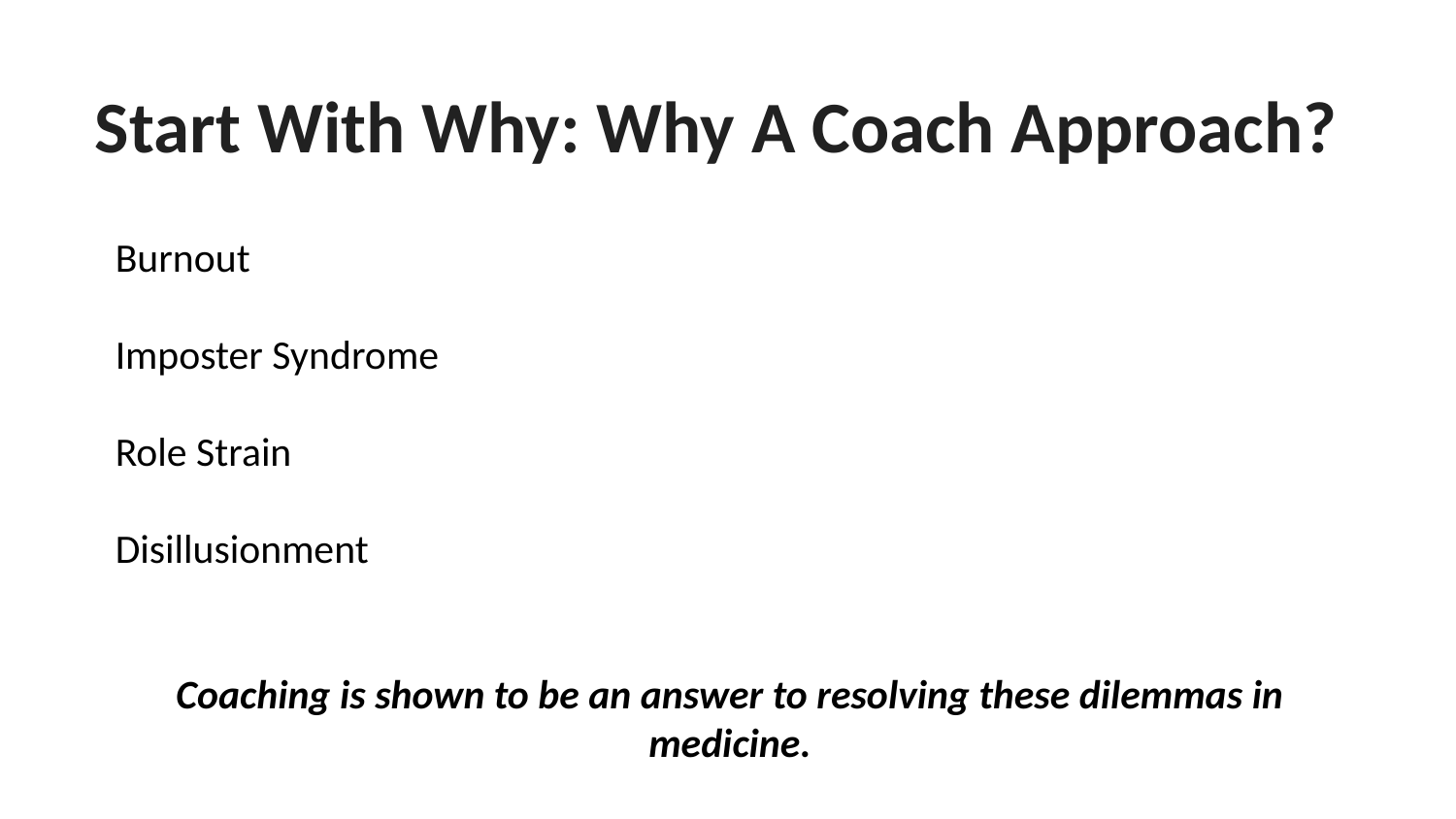

# Start With Why: Why A Coach Approach?
Burnout
Imposter Syndrome
Role Strain
Disillusionment
Coaching is shown to be an answer to resolving these dilemmas in medicine.

## Slide 4
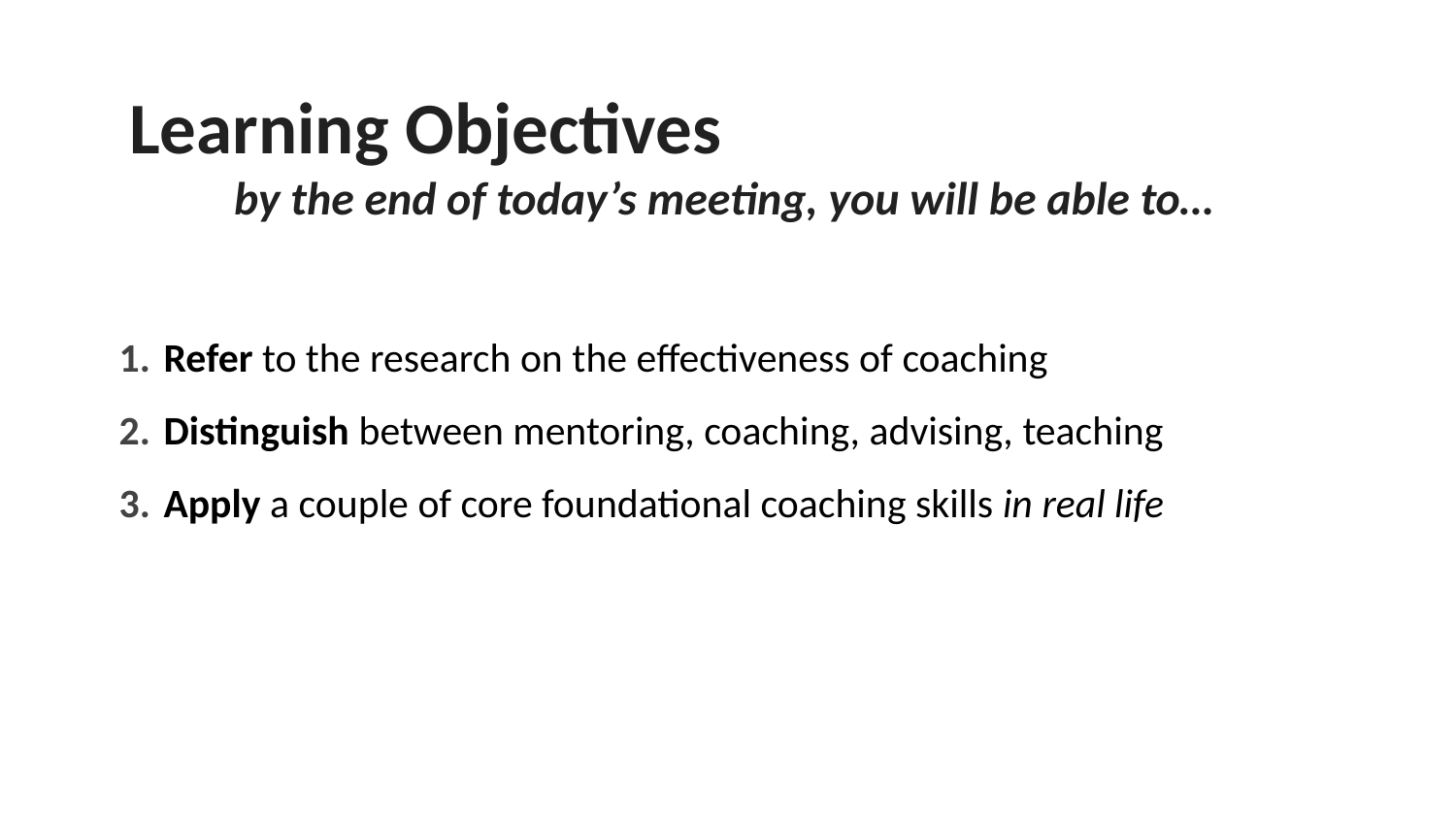

# Learning Objectives
by the end of today’s meeting, you will be able to…
Refer to the research on the effectiveness of coaching
Distinguish between mentoring, coaching, advising, teaching
Apply a couple of core foundational coaching skills in real life

## Slide 5
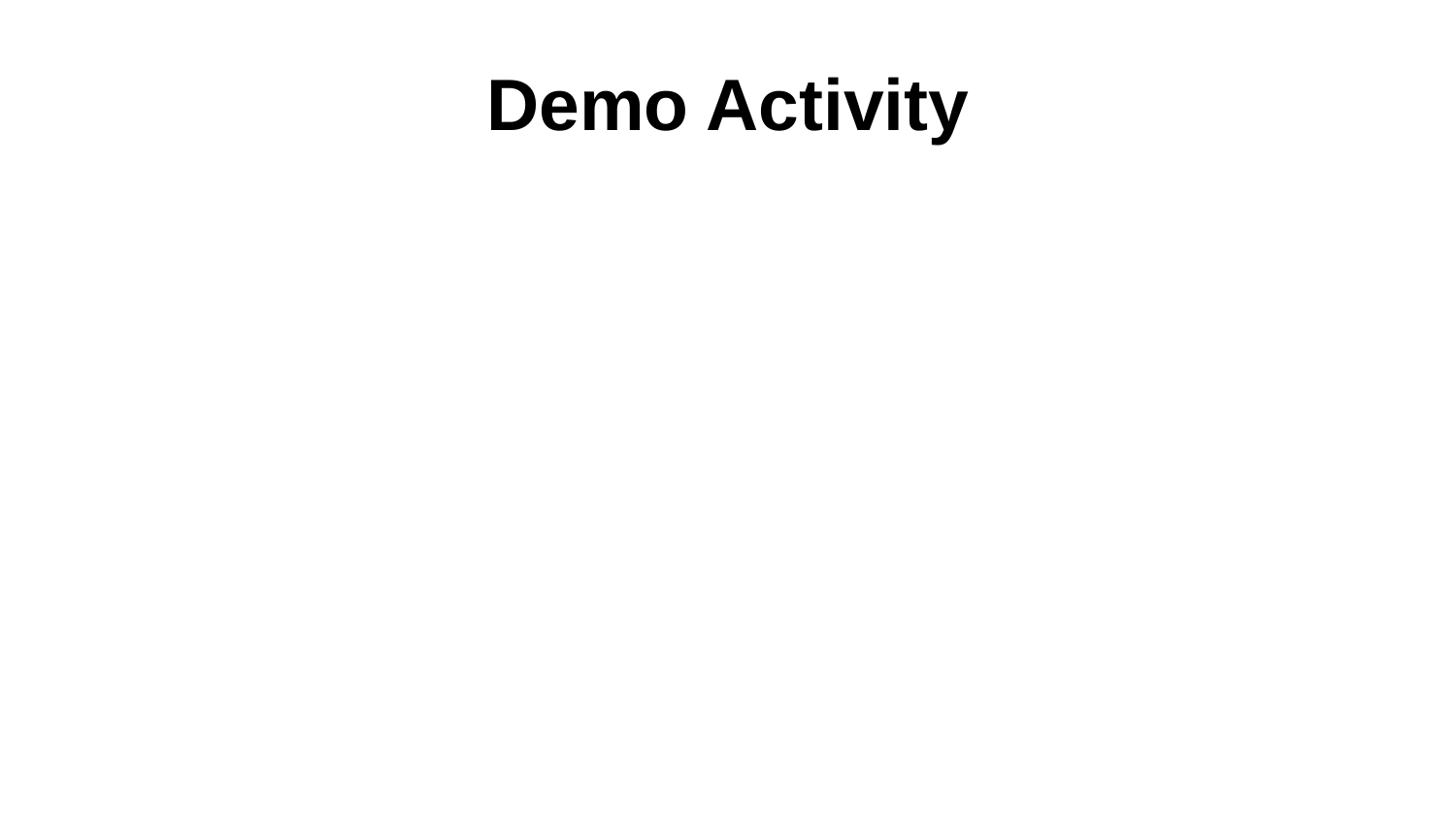

# Demo Activity

## Slide 6
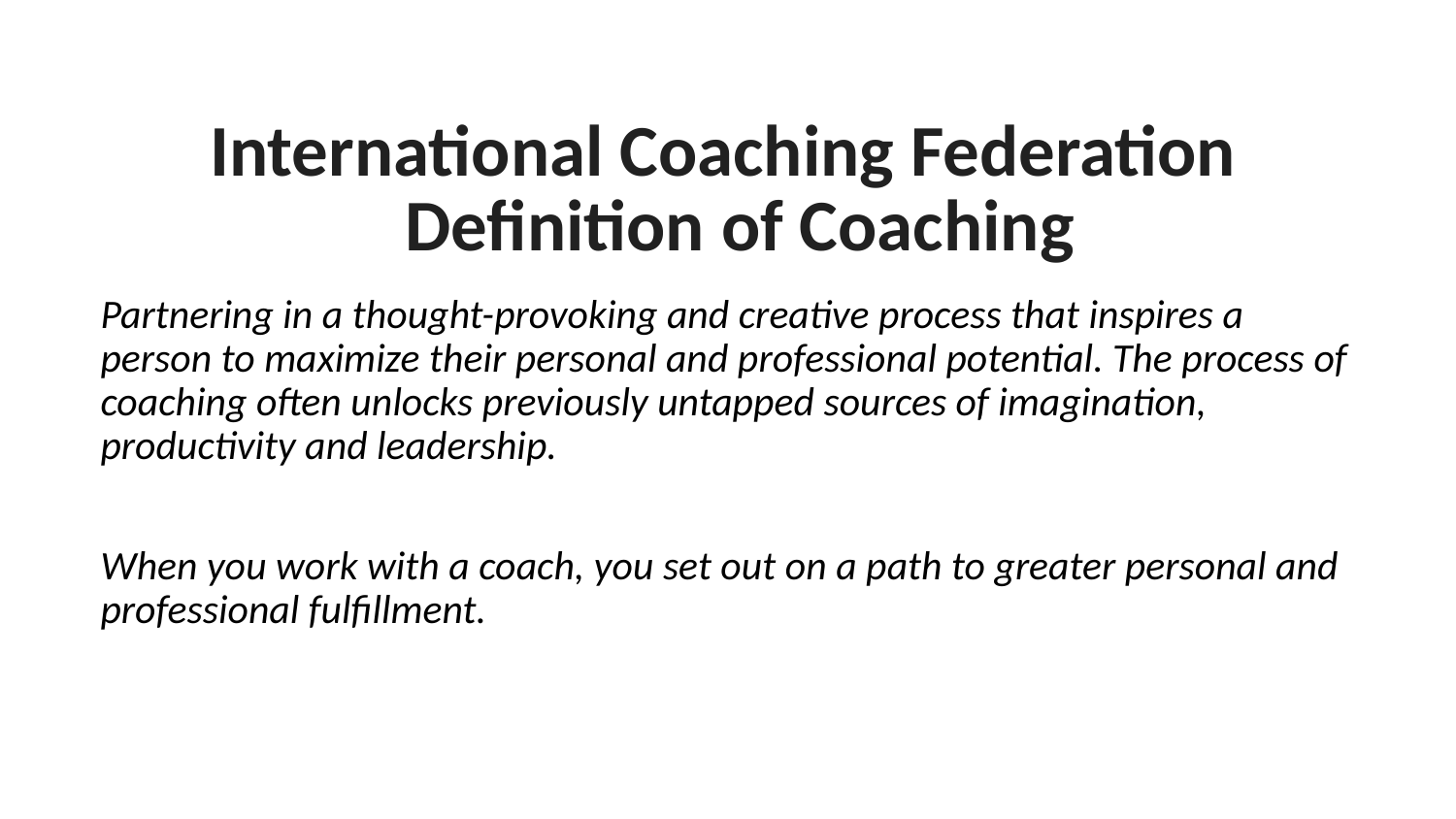

# International Coaching Federation
 Definition of Coaching
Partnering in a thought-provoking and creative process that inspires a person to maximize their personal and professional potential. The process of coaching often unlocks previously untapped sources of imagination, productivity and leadership.
When you work with a coach, you set out on a path to greater personal and professional fulfillment.

## Slide 7
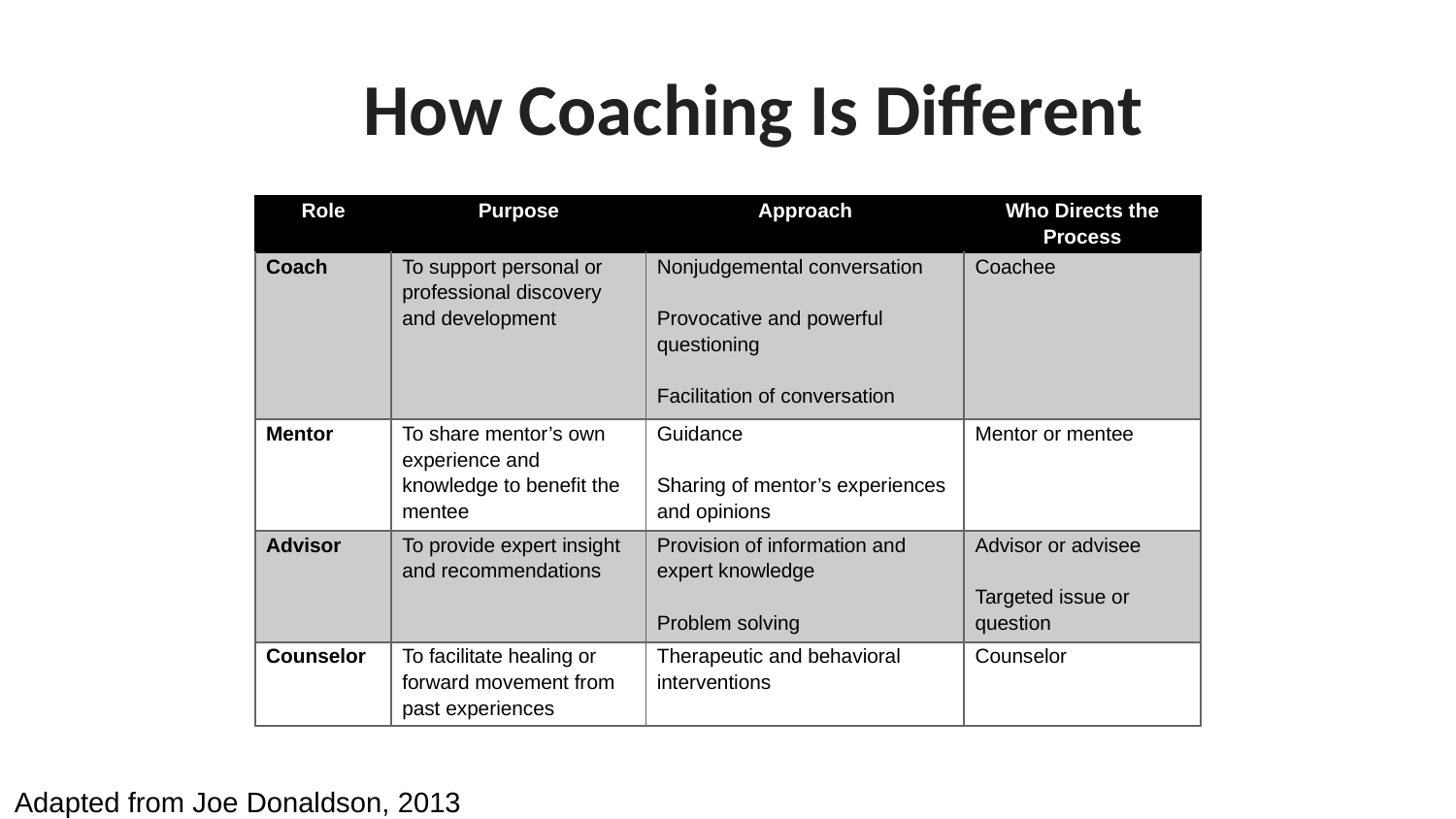

# How Coaching Is Different
| Role | Purpose | Approach | Who Directs the Process |
| --- | --- | --- | --- |
| Coach | To support personal or professional discovery and development | Nonjudgemental conversation   Provocative and powerful questioning   Facilitation of conversation | Coachee |
| Mentor | To share mentor’s own experience and knowledge to benefit the mentee | Guidance   Sharing of mentor’s experiences and opinions | Mentor or mentee |
| Advisor | To provide expert insight and recommendations | Provision of information and expert knowledge   Problem solving | Advisor or advisee   Targeted issue or question |
| Counselor | To facilitate healing or forward movement from past experiences | Therapeutic and behavioral interventions | Counselor |
Adapted from Joe Donaldson, 2013

## Slide 8
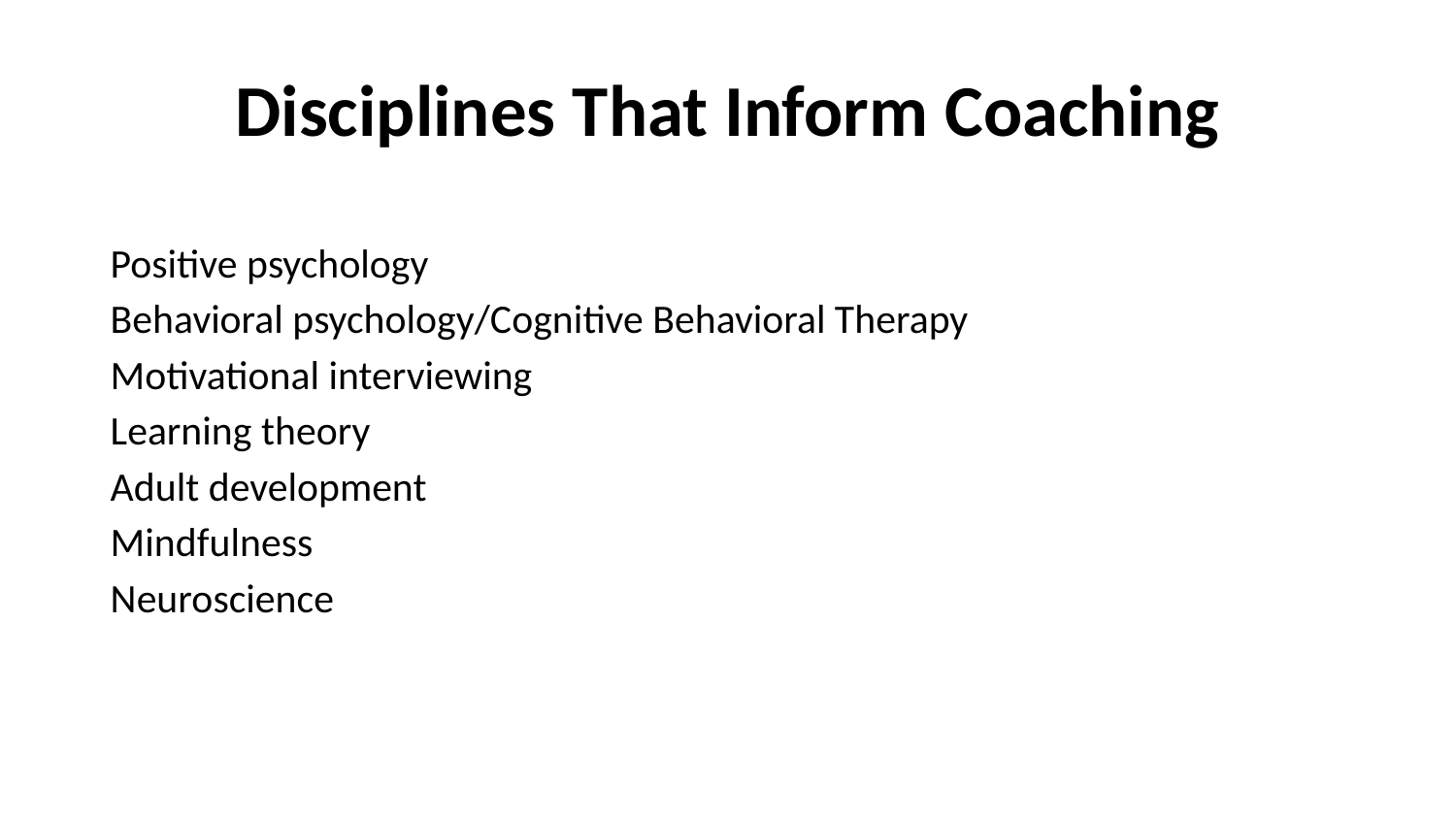

# Disciplines That Inform Coaching
Positive psychology
Behavioral psychology/Cognitive Behavioral Therapy
Motivational interviewing
Learning theory
Adult development
Mindfulness
Neuroscience

## Slide 9
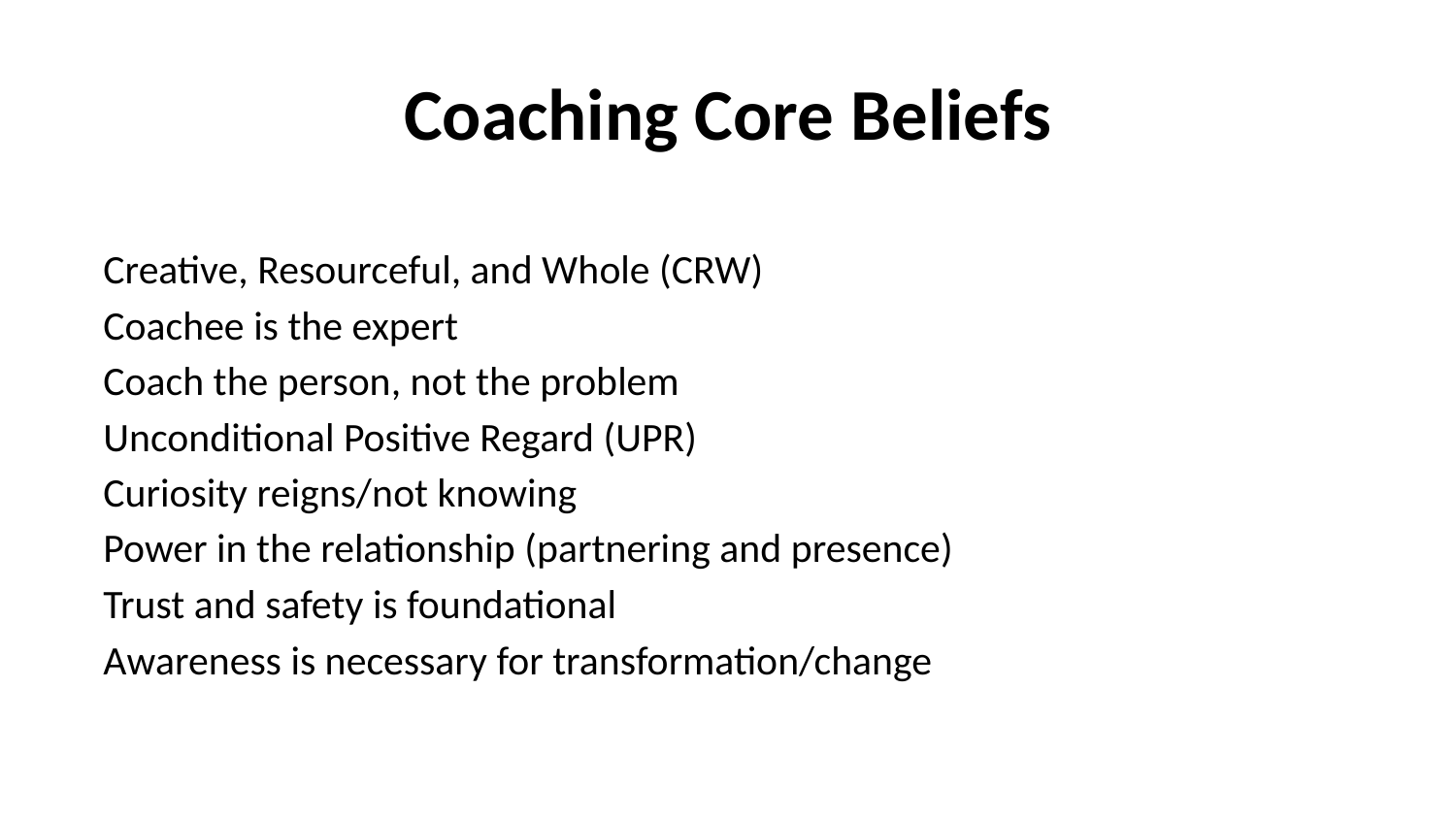

# Coaching Core Beliefs
Creative, Resourceful, and Whole (CRW)
Coachee is the expert
Coach the person, not the problem
Unconditional Positive Regard (UPR)
Curiosity reigns/not knowing
Power in the relationship (partnering and presence)
Trust and safety is foundational
Awareness is necessary for transformation/change

## Slide 10
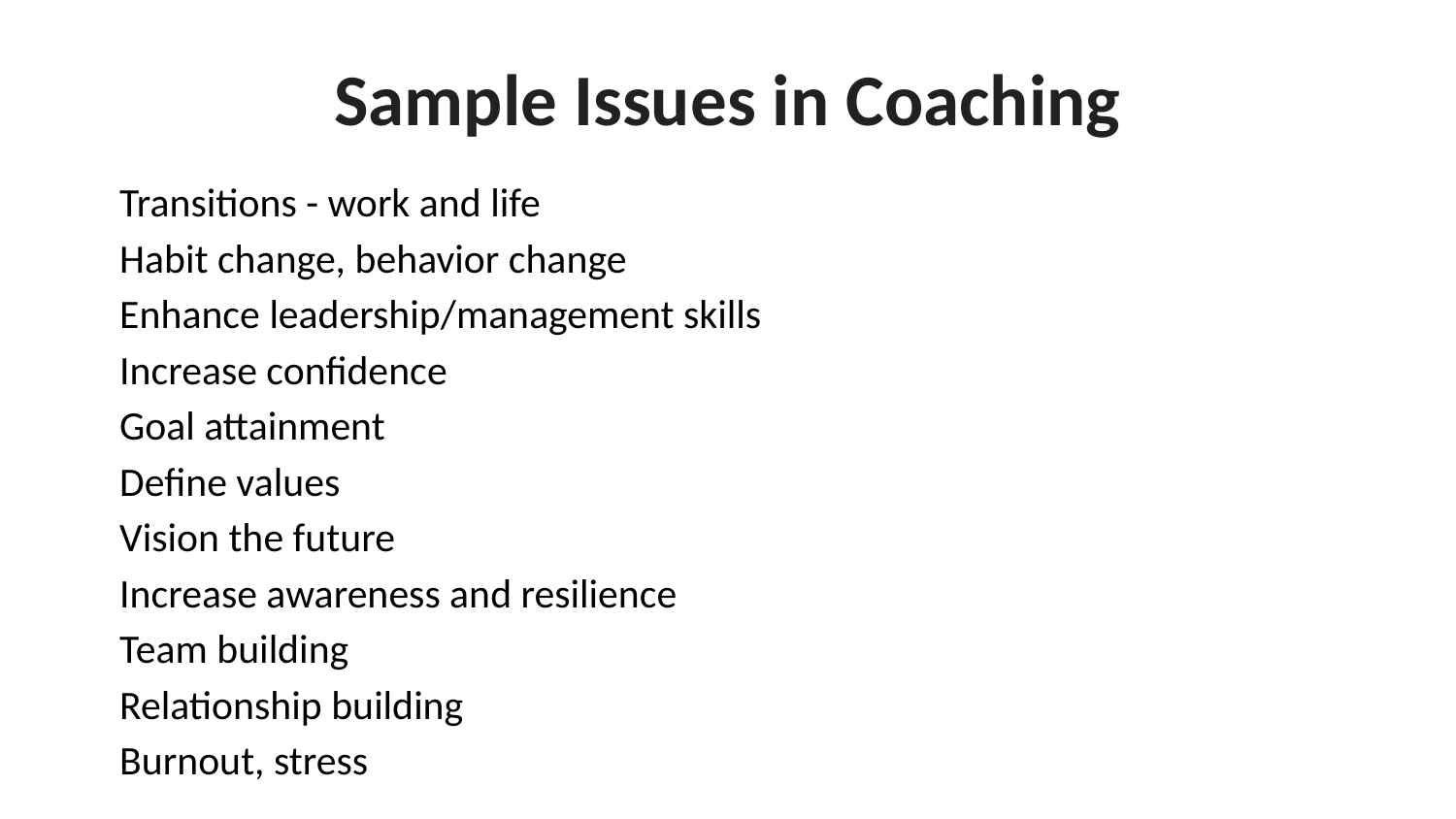

# Sample Issues in Coaching
Transitions - work and life
Habit change, behavior change
Enhance leadership/management skills
Increase confidence
Goal attainment
Define values
Vision the future
Increase awareness and resilience
Team building
Relationship building
Burnout, stress

## Slide 11
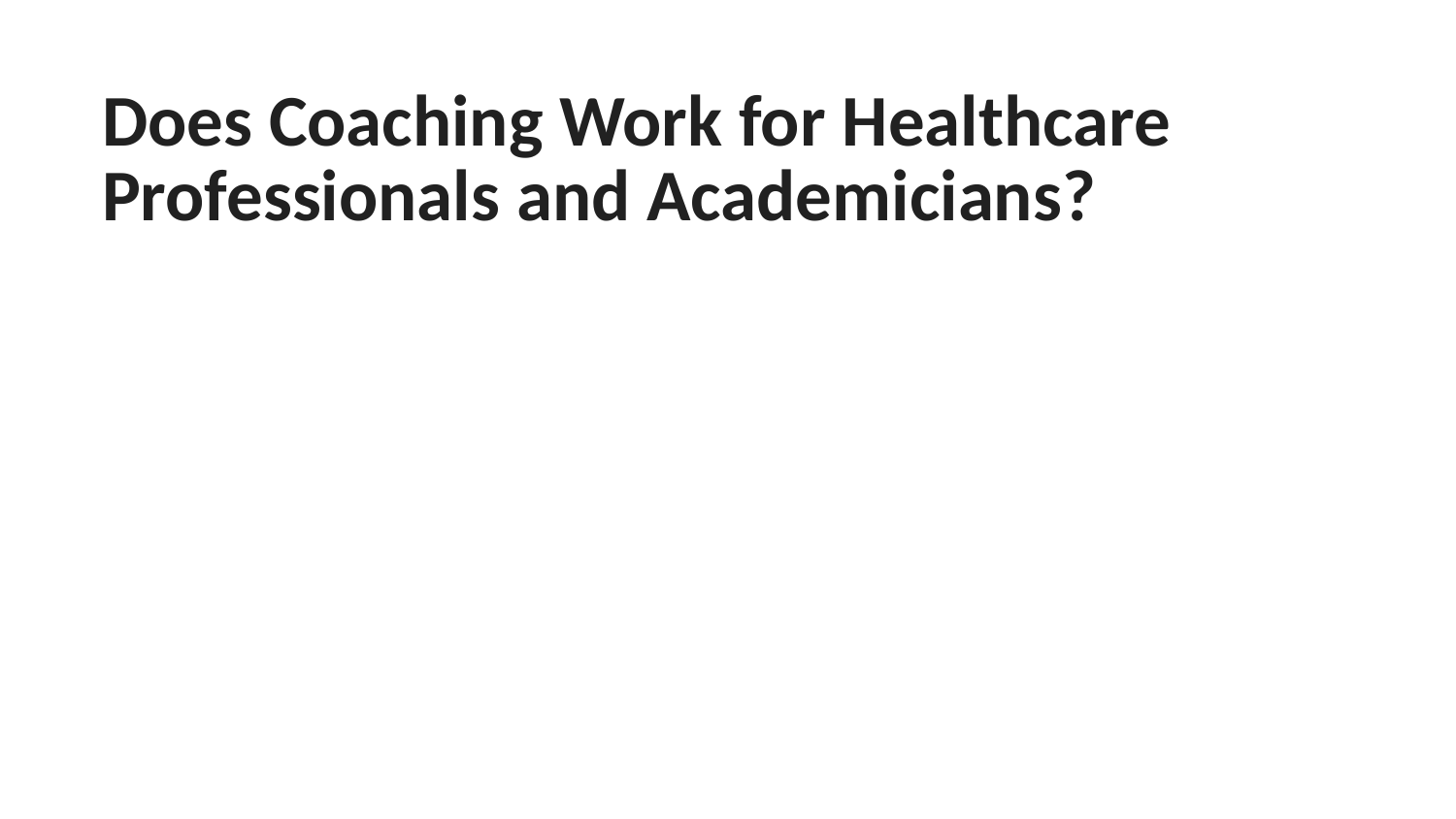

# Does Coaching Work for Healthcare Professionals and Academicians?

## Slide 12
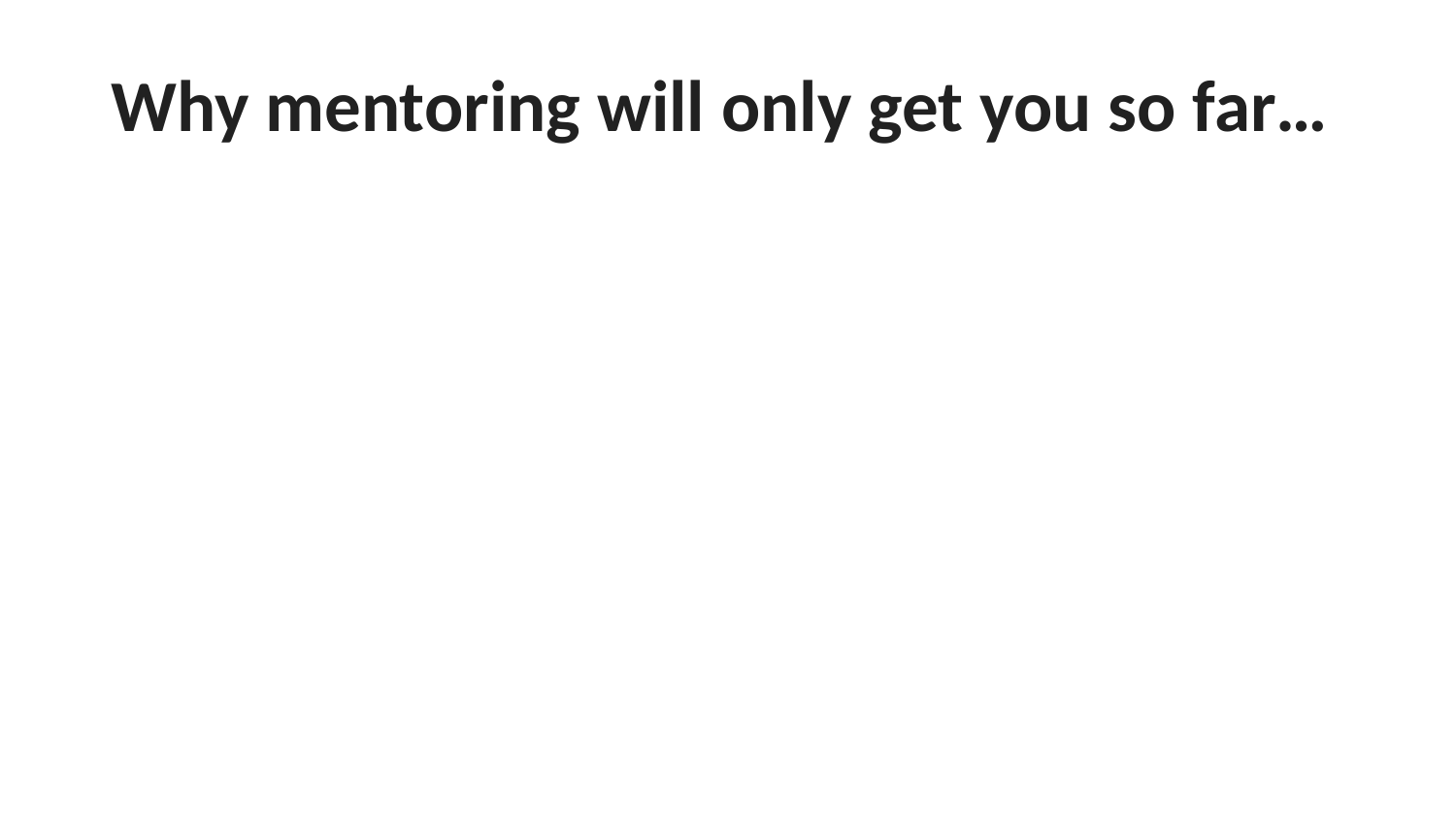

# Why mentoring will only get you so far…

## Slide 13
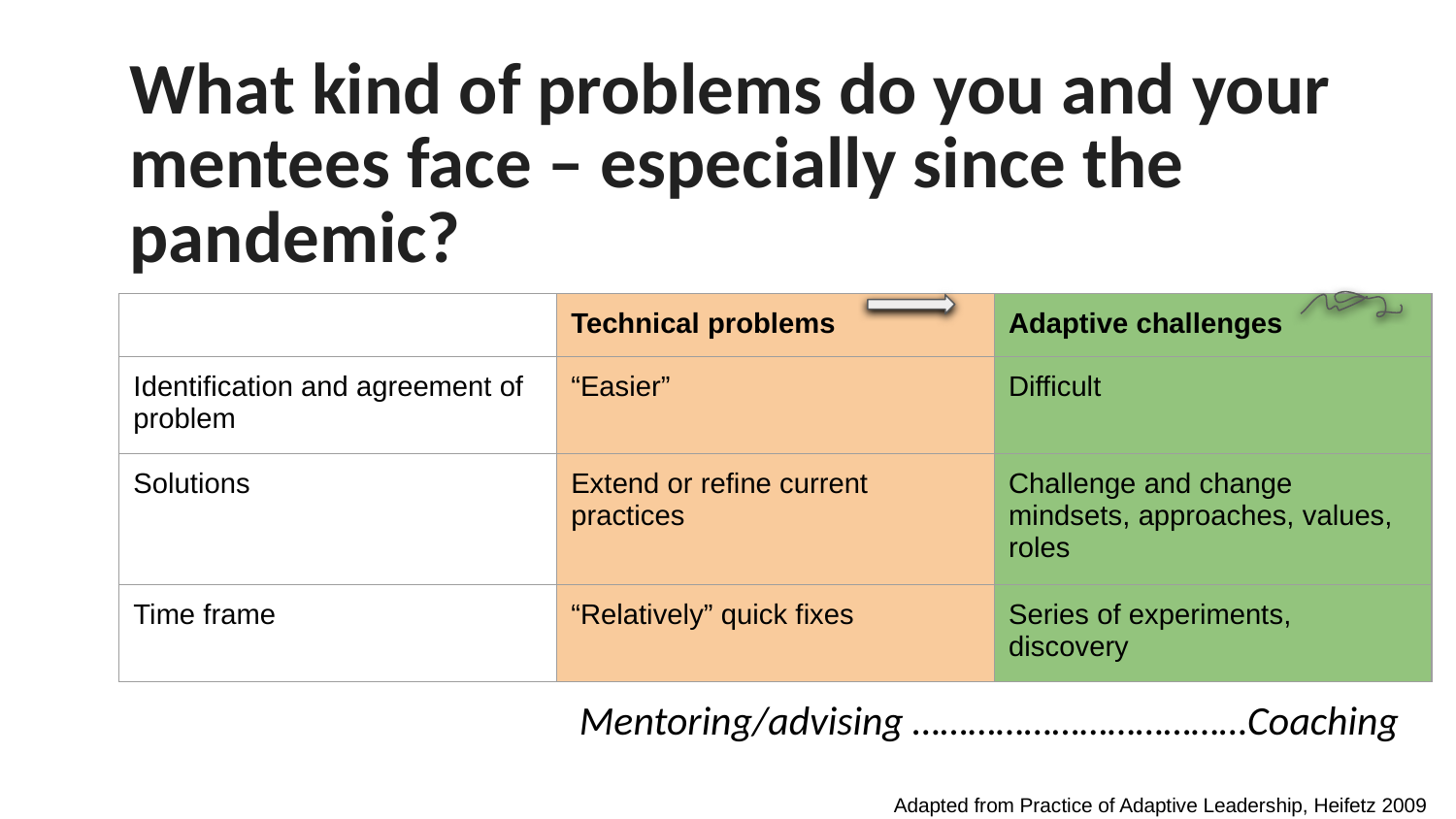

# What kind of problems do you and your mentees face – especially since the pandemic?
| | Technical problems | Adaptive challenges |
| --- | --- | --- |
| Identification and agreement of problem | “Easier” | Difficult |
| Solutions | Extend or refine current practices | Challenge and change mindsets, approaches, values, roles |
| Time frame | “Relatively” quick fixes | Series of experiments, discovery |
Mentoring/advising ………………………………Coaching
Adapted from Practice of Adaptive Leadership, Heifetz 2009

## Slide 14
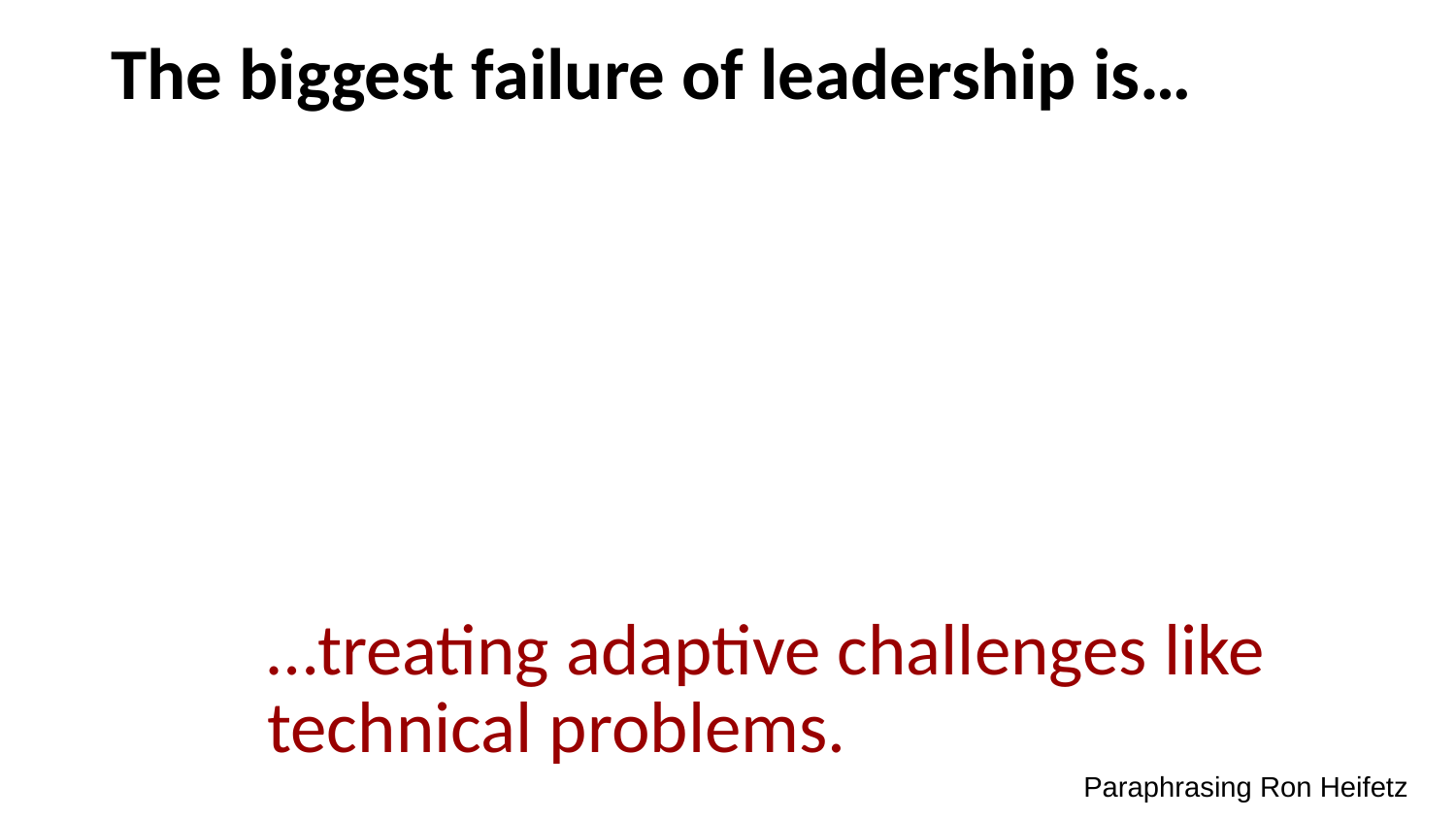

# The biggest failure of leadership is…
…treating adaptive challenges like technical problems.
Paraphrasing Ron Heifetz

## Slide 15
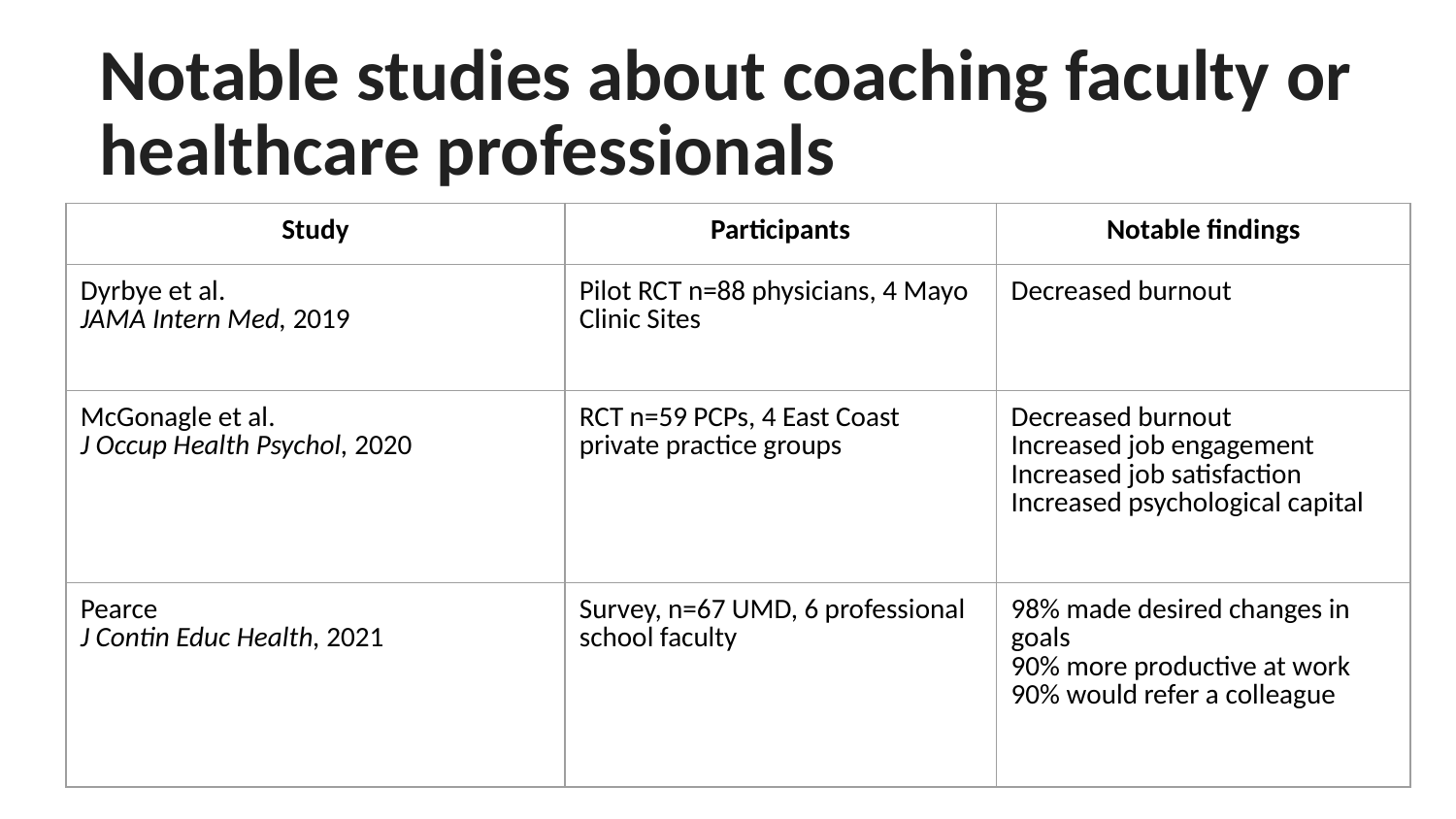

# Notable studies about coaching faculty or healthcare professionals
| Study | Participants | Notable findings |
| --- | --- | --- |
| Dyrbye et al. JAMA Intern Med, 2019 | Pilot RCT n=88 physicians, 4 Mayo Clinic Sites | Decreased burnout |
| McGonagle et al. J Occup Health Psychol, 2020 | RCT n=59 PCPs, 4 East Coast private practice groups | Decreased burnout Increased job engagement Increased job satisfaction Increased psychological capital |
| Pearce J Contin Educ Health, 2021 | Survey, n=67 UMD, 6 professional school faculty | 98% made desired changes in goals 90% more productive at work 90% would refer a colleague |

## Slide 16
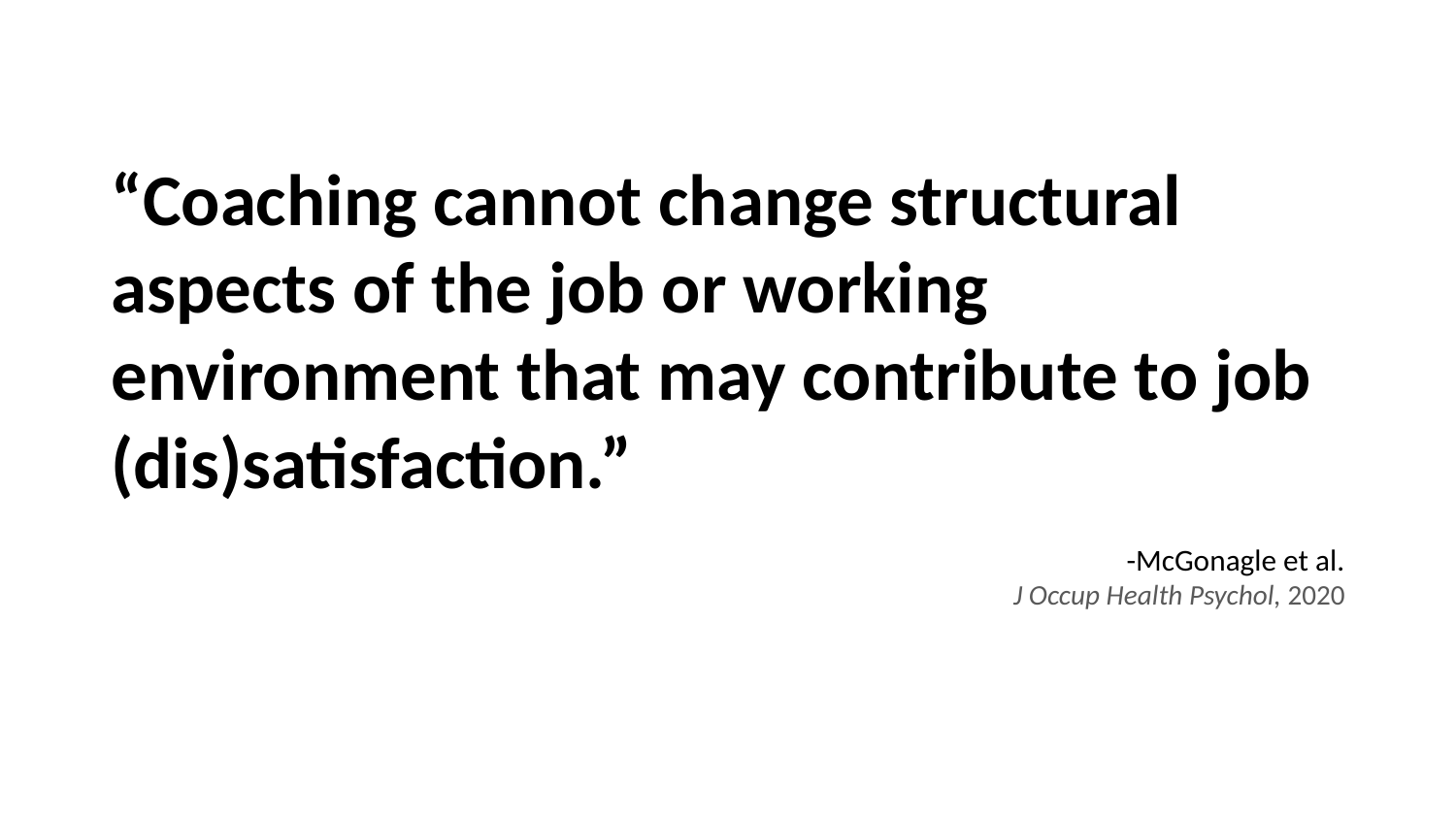

“Coaching cannot change structural aspects of the job or working environment that may contribute to job (dis)satisfaction.”
-McGonagle et al.
J Occup Health Psychol, 2020

## Slide 17
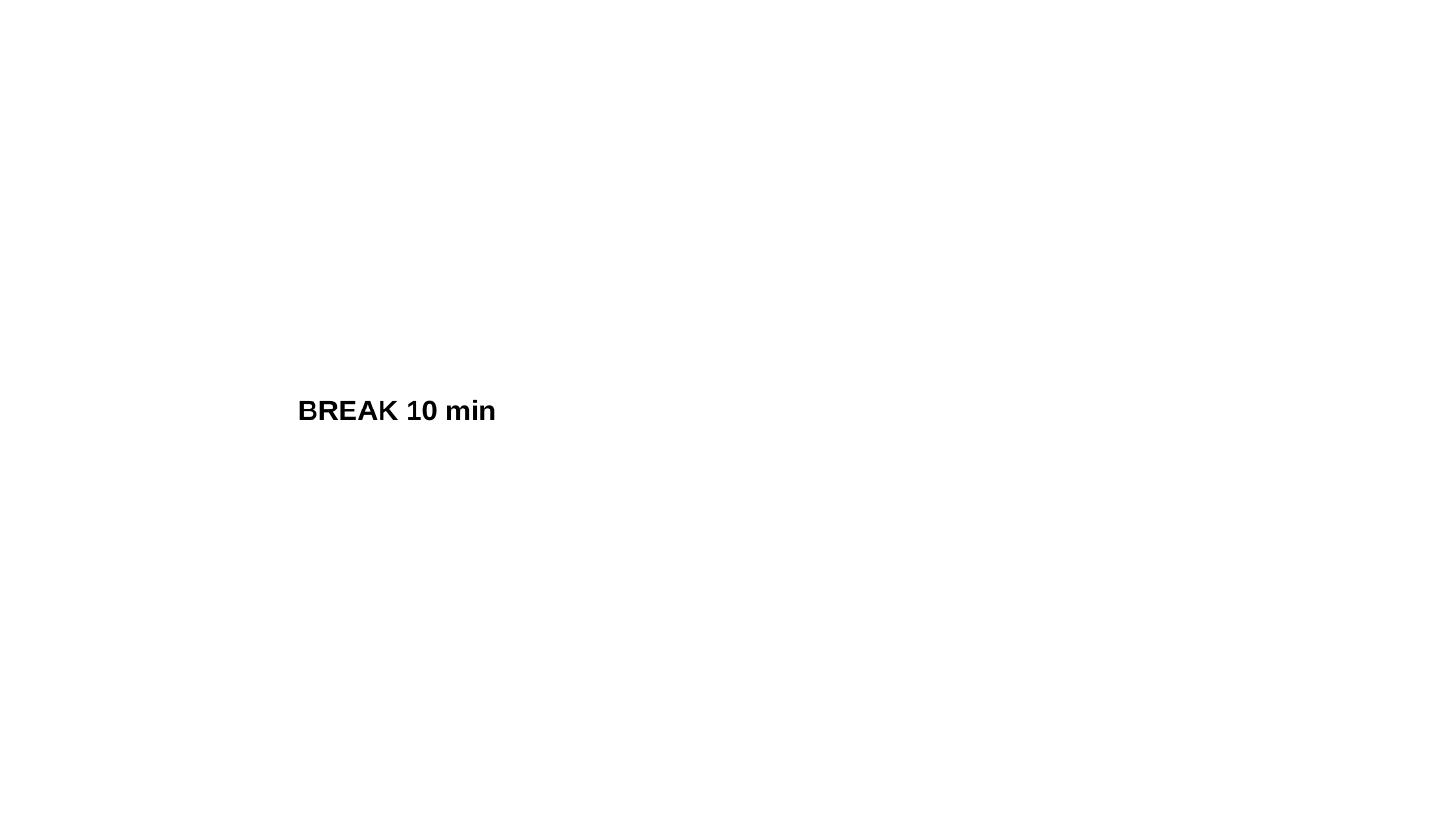

BREAK 10 min

## Slide 18
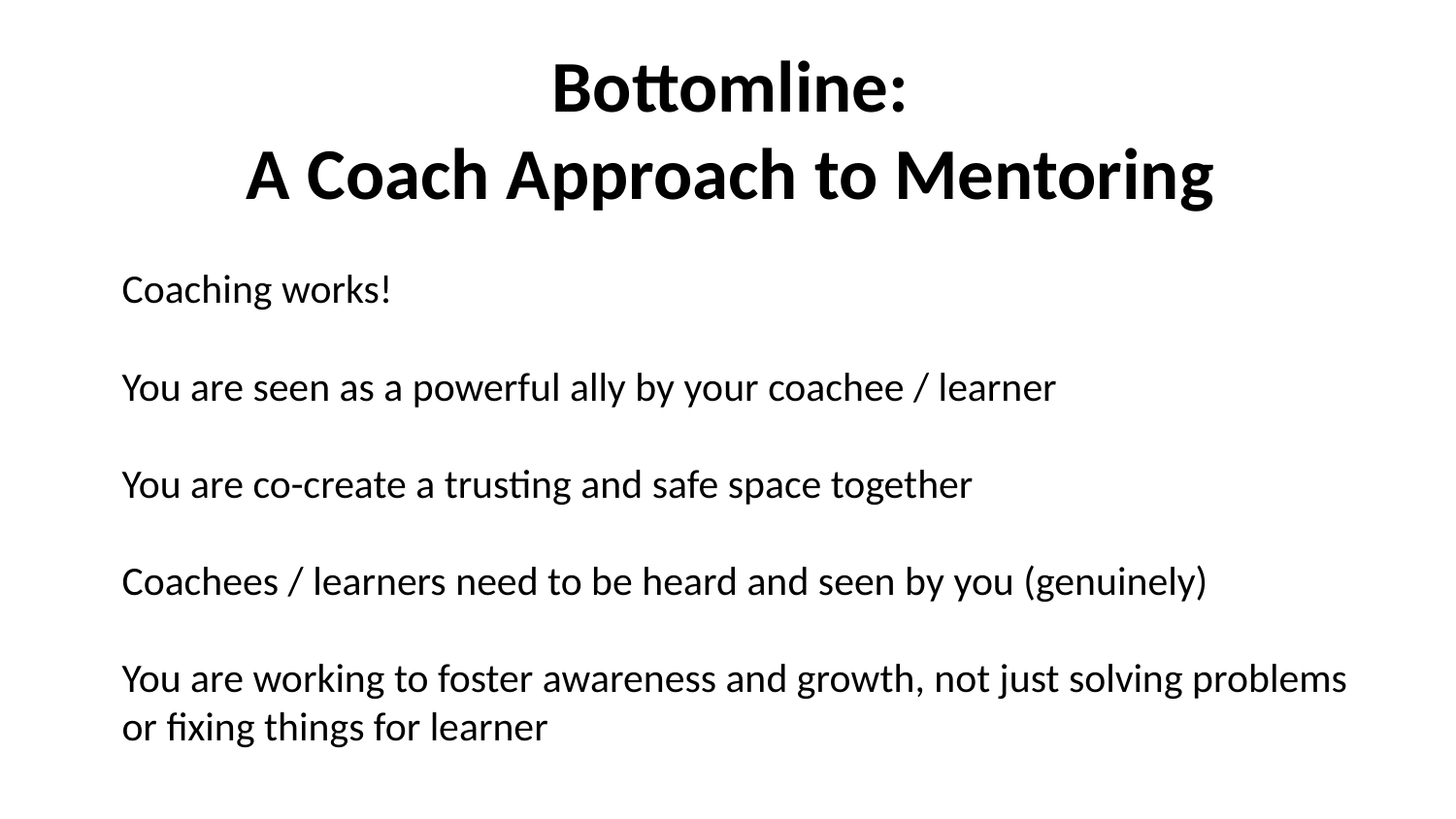

# Bottomline:
A Coach Approach to Mentoring
Coaching works!
You are seen as a powerful ally by your coachee / learner
You are co-create a trusting and safe space together
Coachees / learners need to be heard and seen by you (genuinely)
You are working to foster awareness and growth, not just solving problems or fixing things for learner

## Slide 19
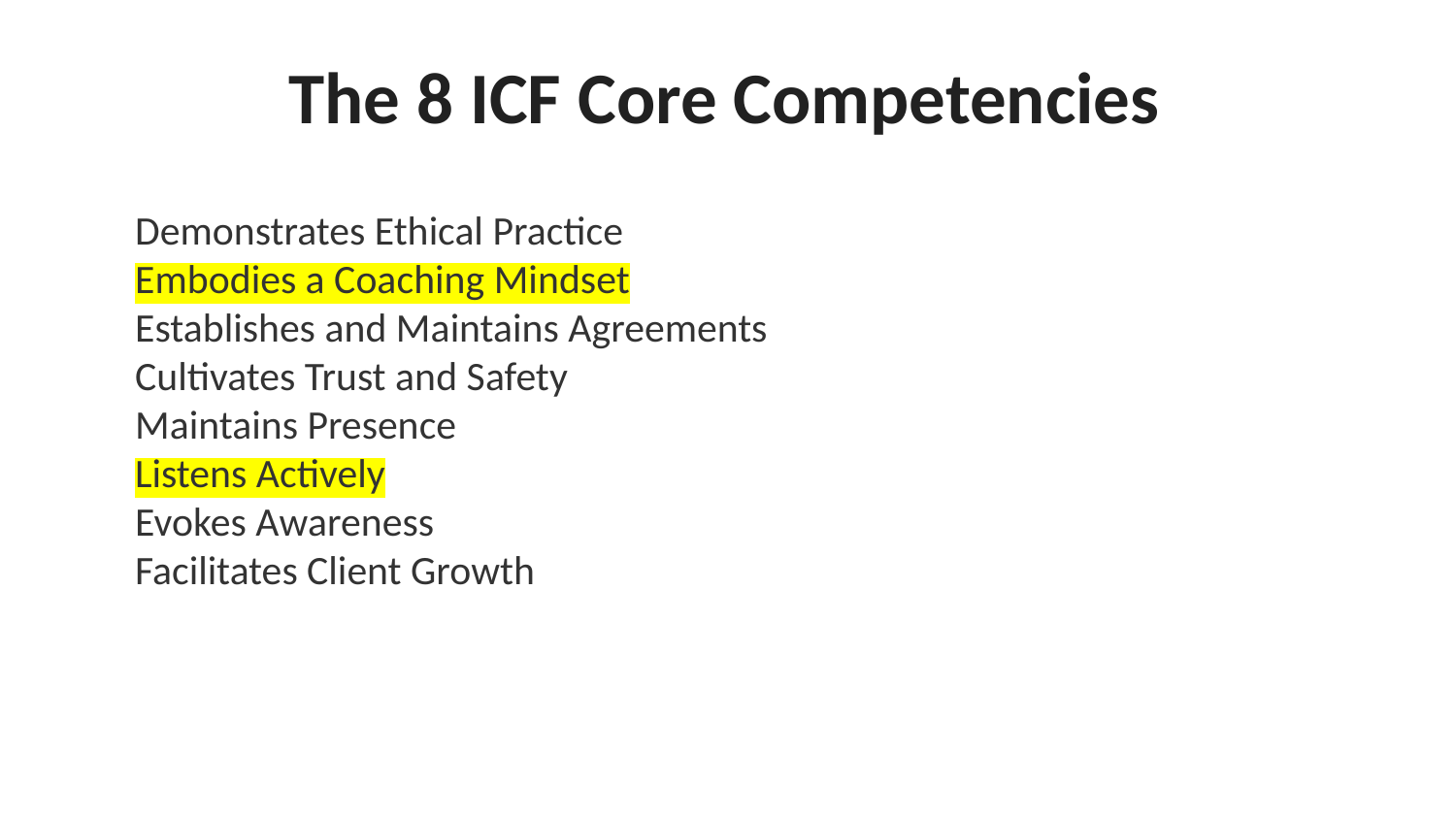

# The 8 ICF Core Competencies
Demonstrates Ethical Practice
Embodies a Coaching Mindset
Establishes and Maintains Agreements
Cultivates Trust and Safety
Maintains Presence
Listens Actively
Evokes Awareness
Facilitates Client Growth

## Slide 20
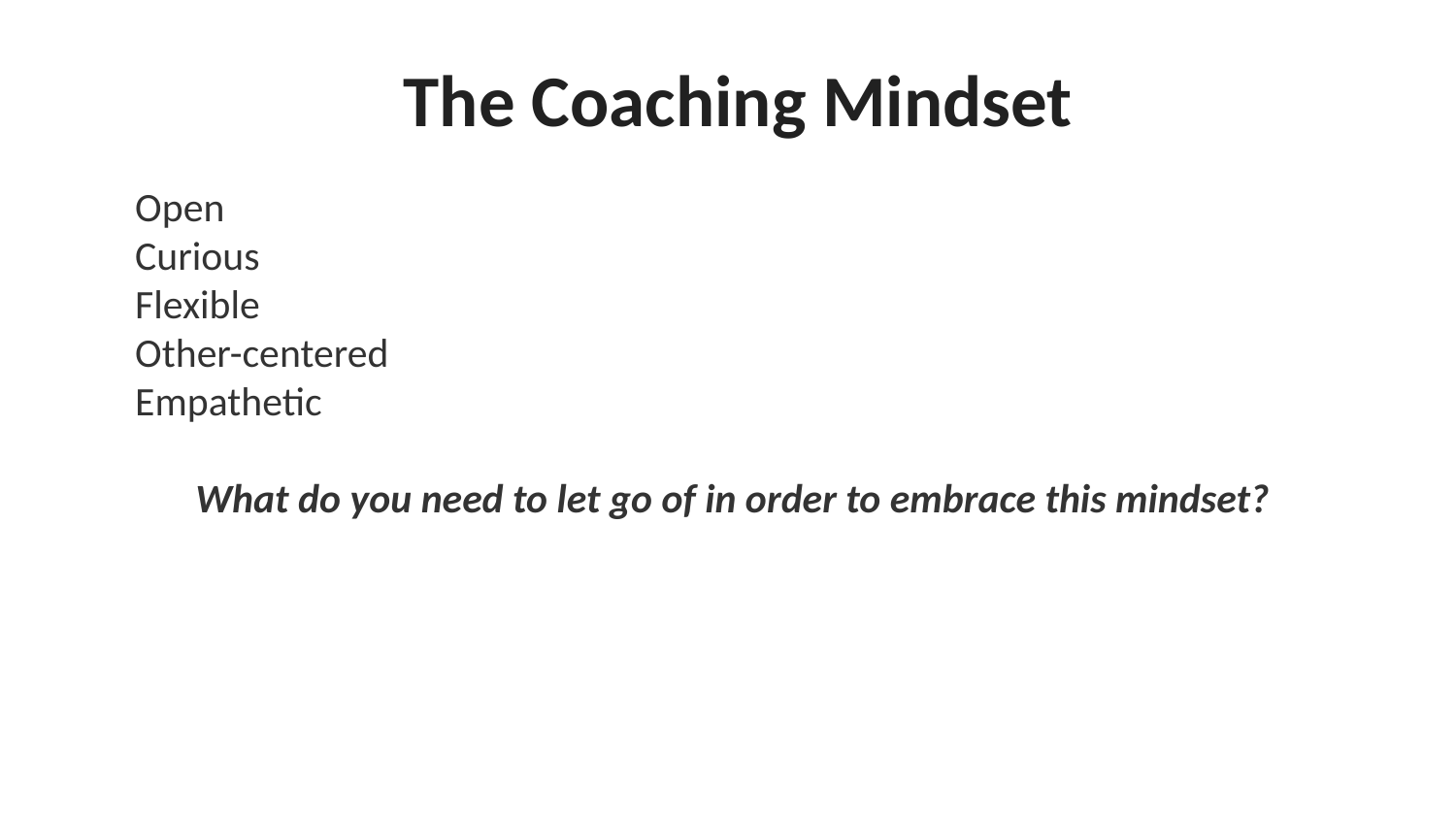

# The Coaching Mindset
Open
Curious
Flexible
Other-centered
Empathetic
What do you need to let go of in order to embrace this mindset?

## Slide 21
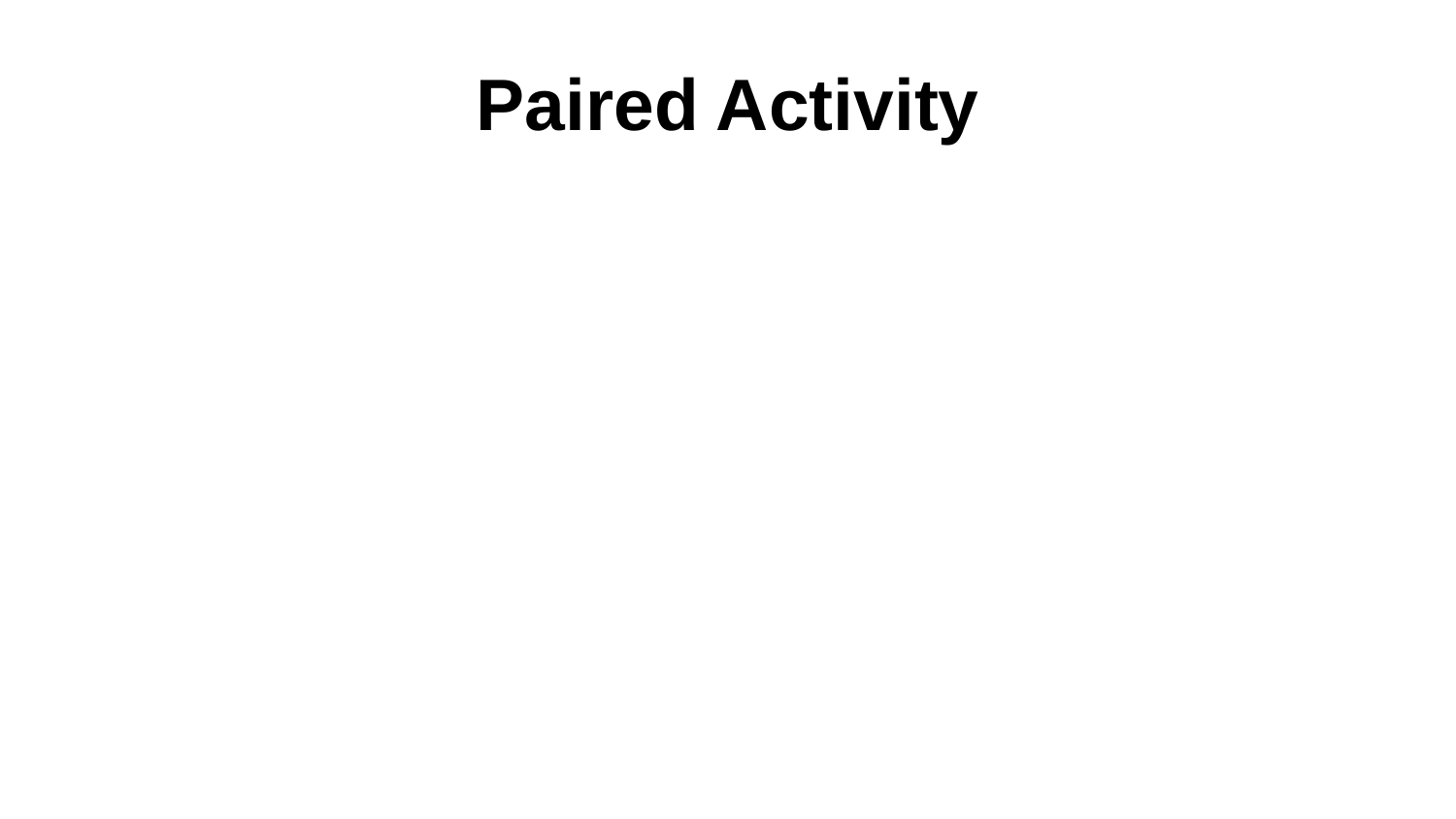

# Paired Activity

## Slide 22
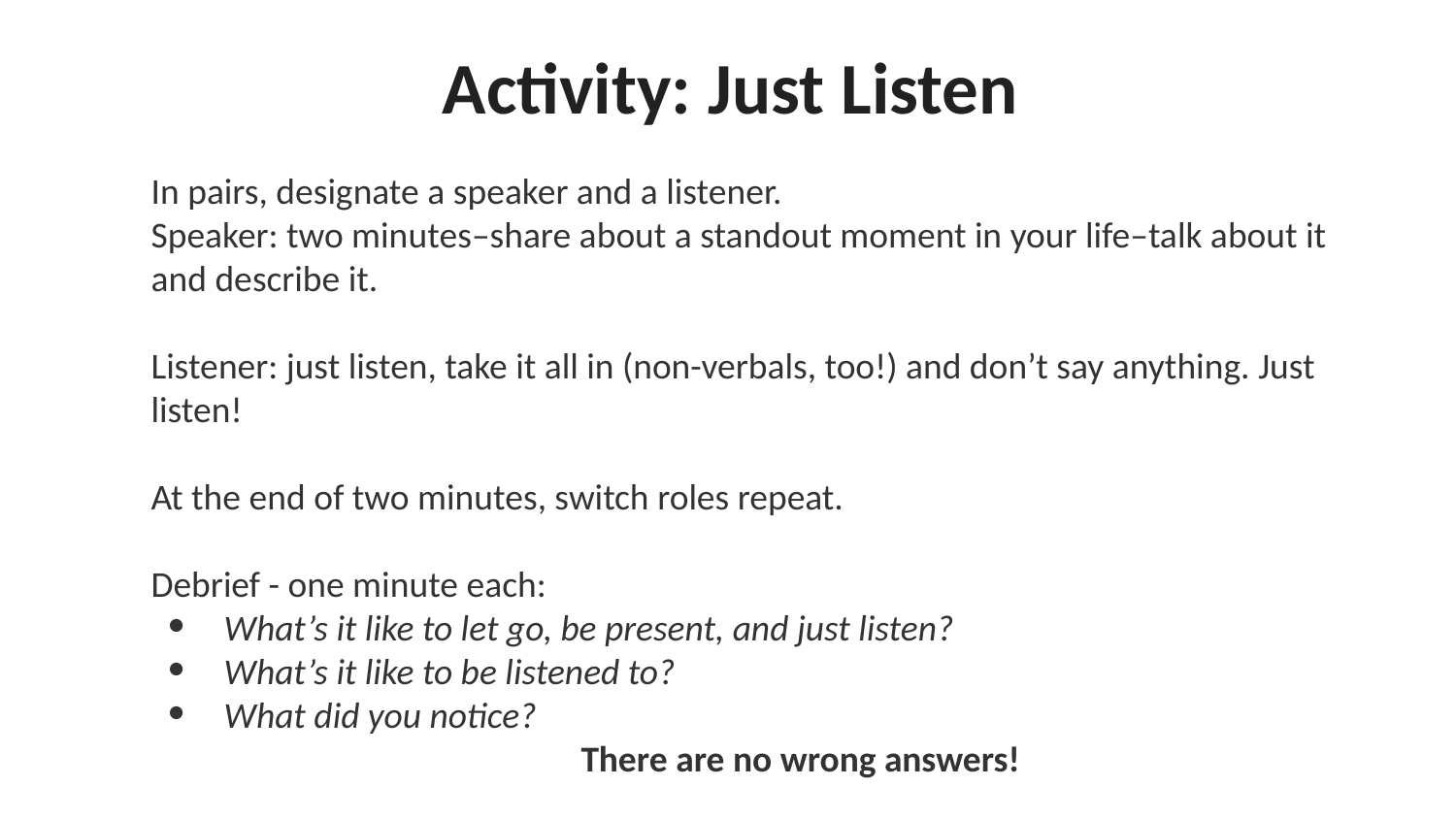

# Activity: Just Listen
In pairs, designate a speaker and a listener.
Speaker: two minutes–share about a standout moment in your life–talk about it and describe it.
Listener: just listen, take it all in (non-verbals, too!) and don’t say anything. Just listen!
At the end of two minutes, switch roles repeat.
Debrief - one minute each:
What’s it like to let go, be present, and just listen?
What’s it like to be listened to?
What did you notice?
There are no wrong answers!

## Slide 23
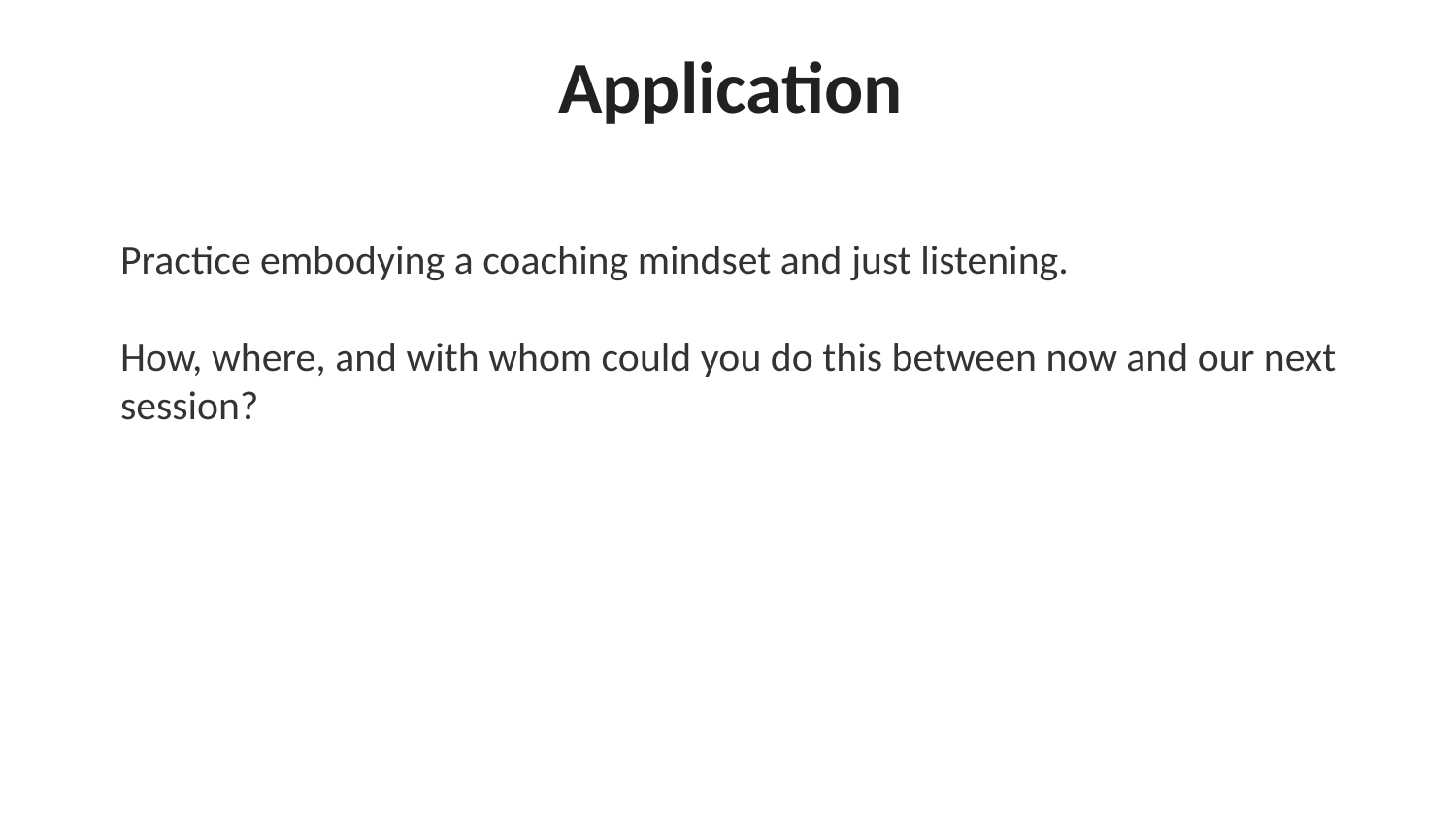

# Application
Practice embodying a coaching mindset and just listening.
How, where, and with whom could you do this between now and our next session?
