# Supplementary material for: A Longitudinal, Practical Curriculum for Faculty Development as New Coaches in Graduate Medical Education
Source: J Educ Teach Emerg Med. 2025 Jul 31;10(3):C1–C92. doi: 10.21980/J88M08 (PMC12320991; doi:10.21980/J88M08)
Supplement: Supplementary file 2 [file 10-3-C1-SuppE3.pptx]

## Slide 1
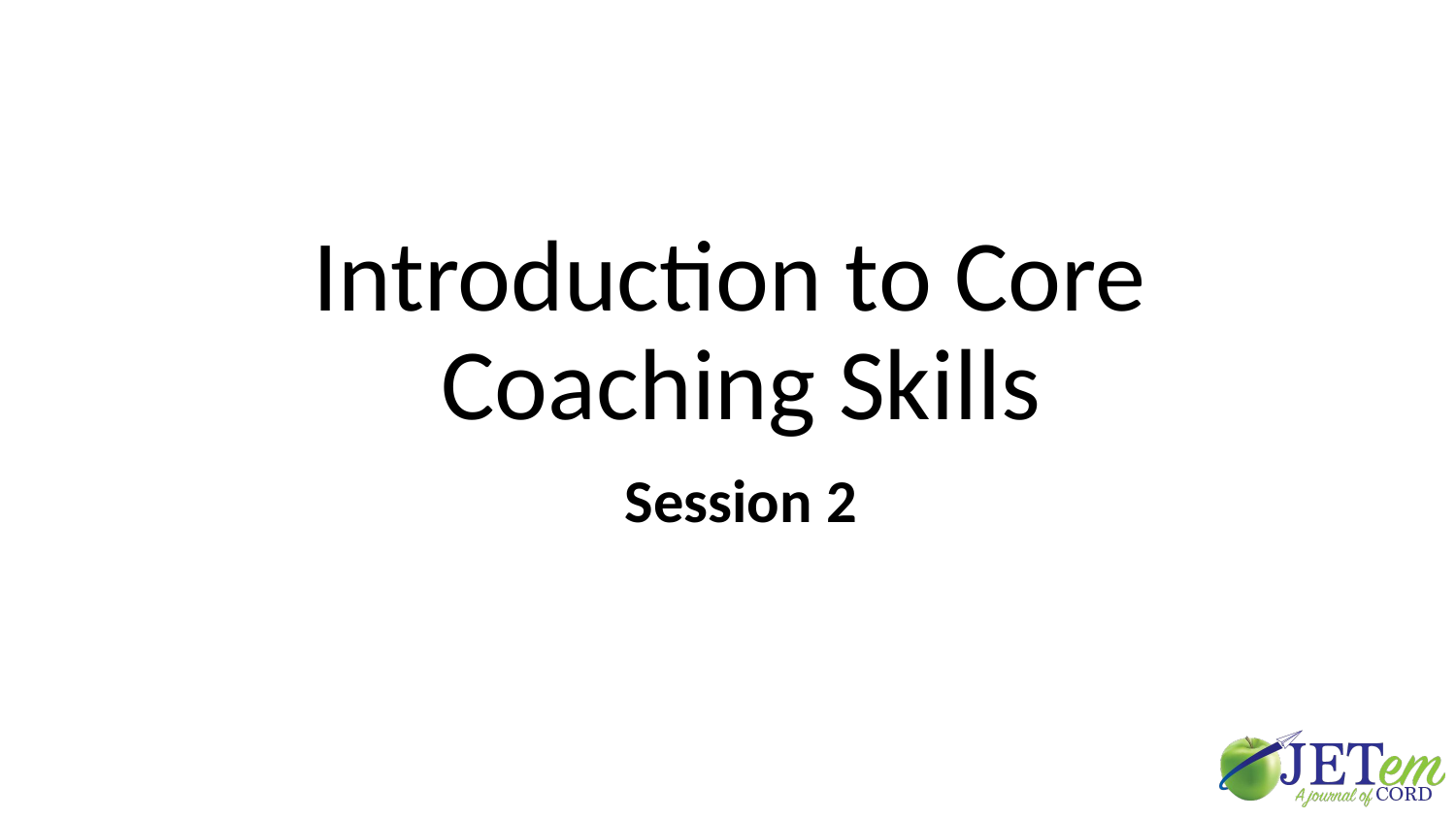

# Introduction to Core
Coaching Skills
Session 2

## Slide 2
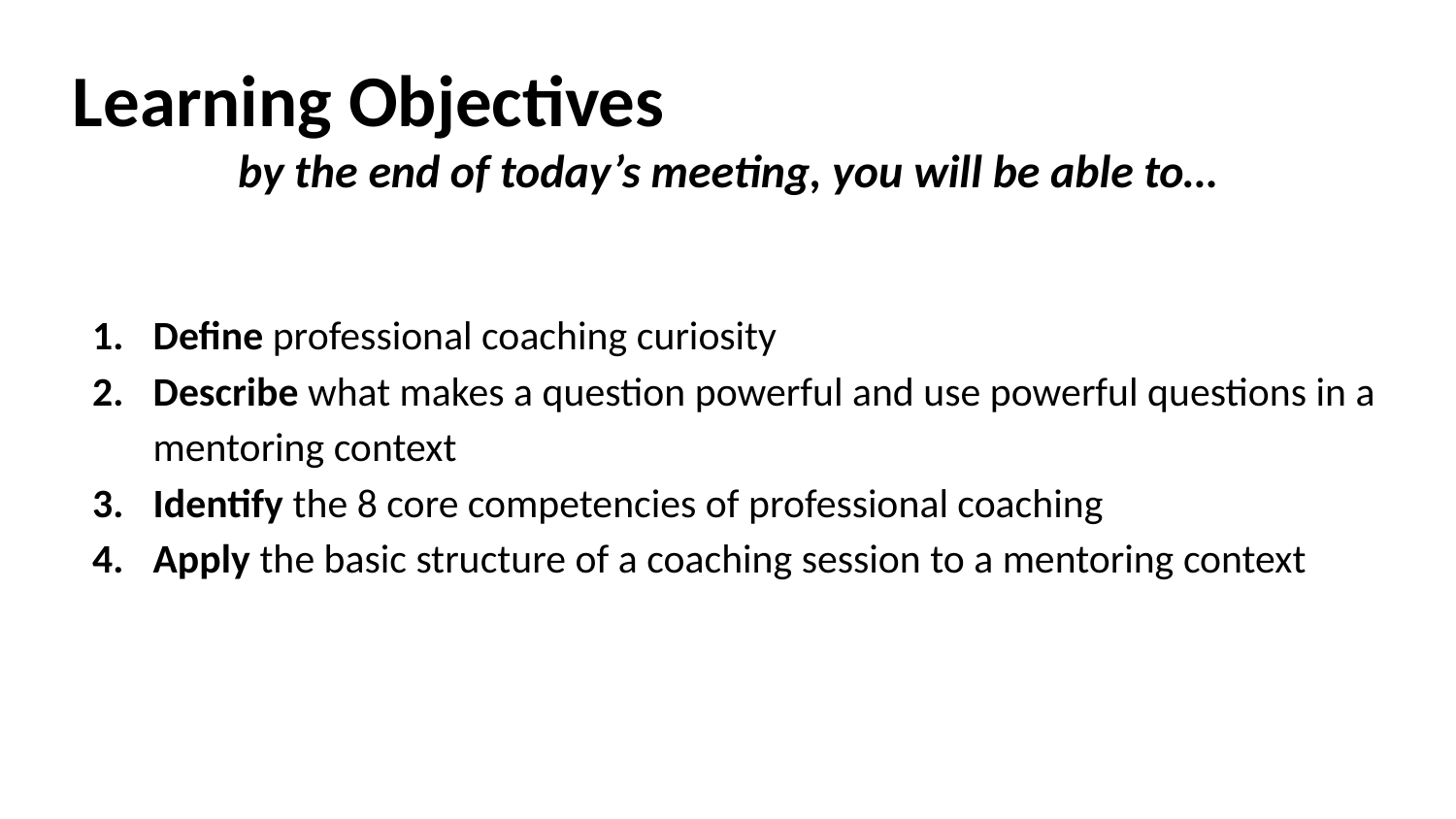

# Learning Objectives
by the end of today’s meeting, you will be able to…
Define professional coaching curiosity
Describe what makes a question powerful and use powerful questions in a mentoring context
Identify the 8 core competencies of professional coaching
Apply the basic structure of a coaching session to a mentoring context

## Slide 3
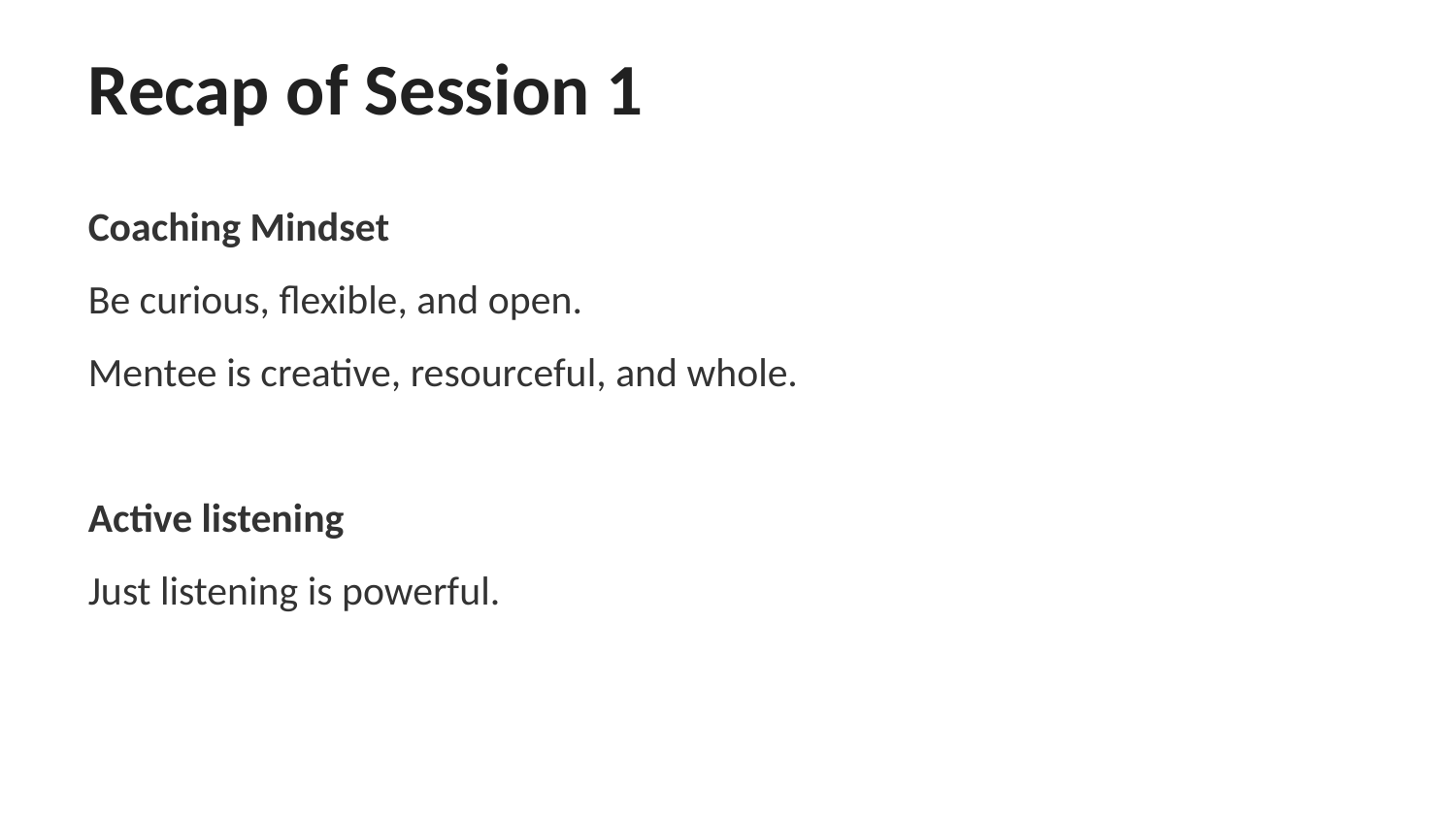

# Recap of Session 1
Coaching Mindset
Be curious, flexible, and open.
Mentee is creative, resourceful, and whole.
Active listening
Just listening is powerful.

## Slide 4
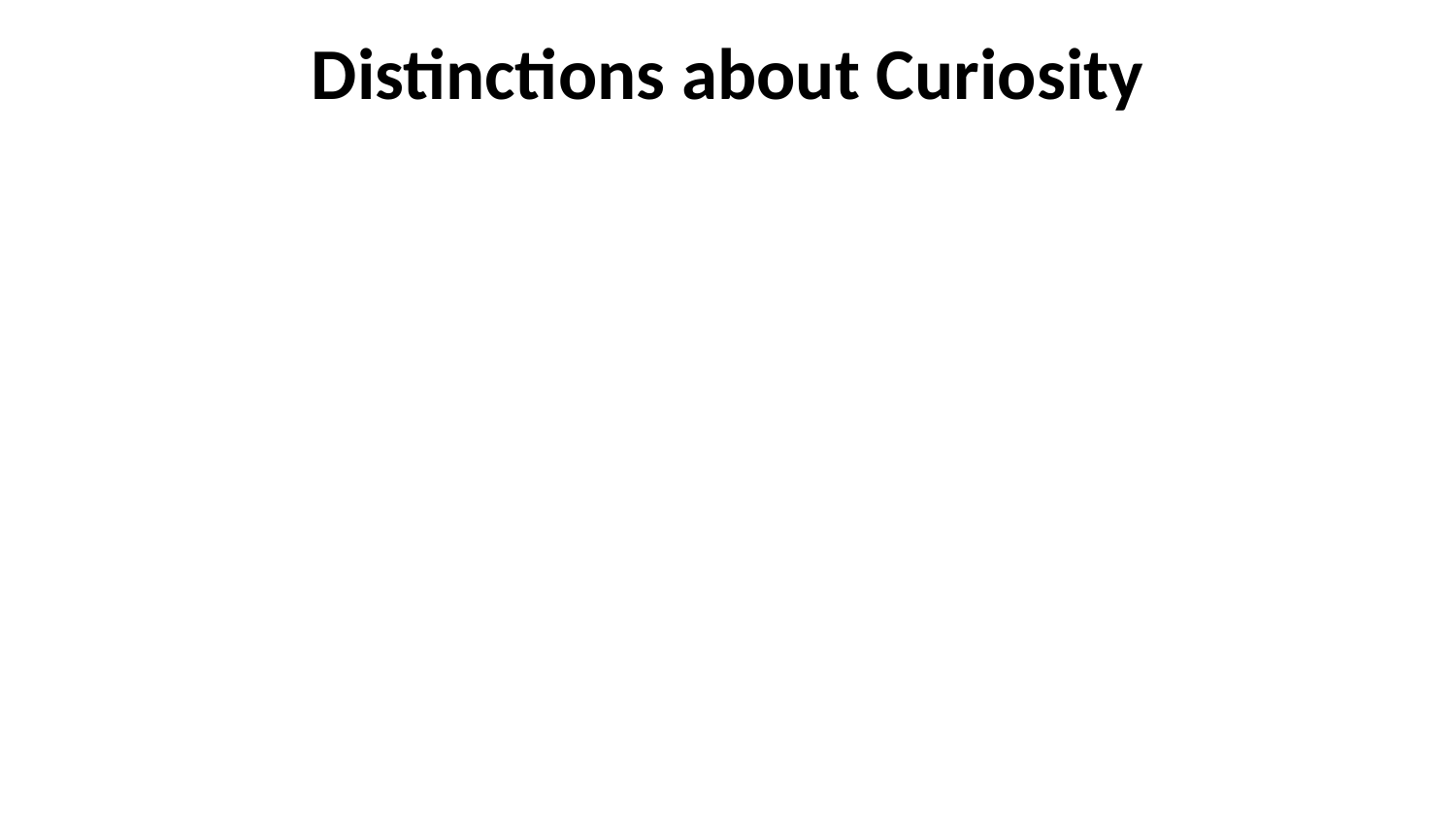

# Distinctions about Curiosity

## Slide 5
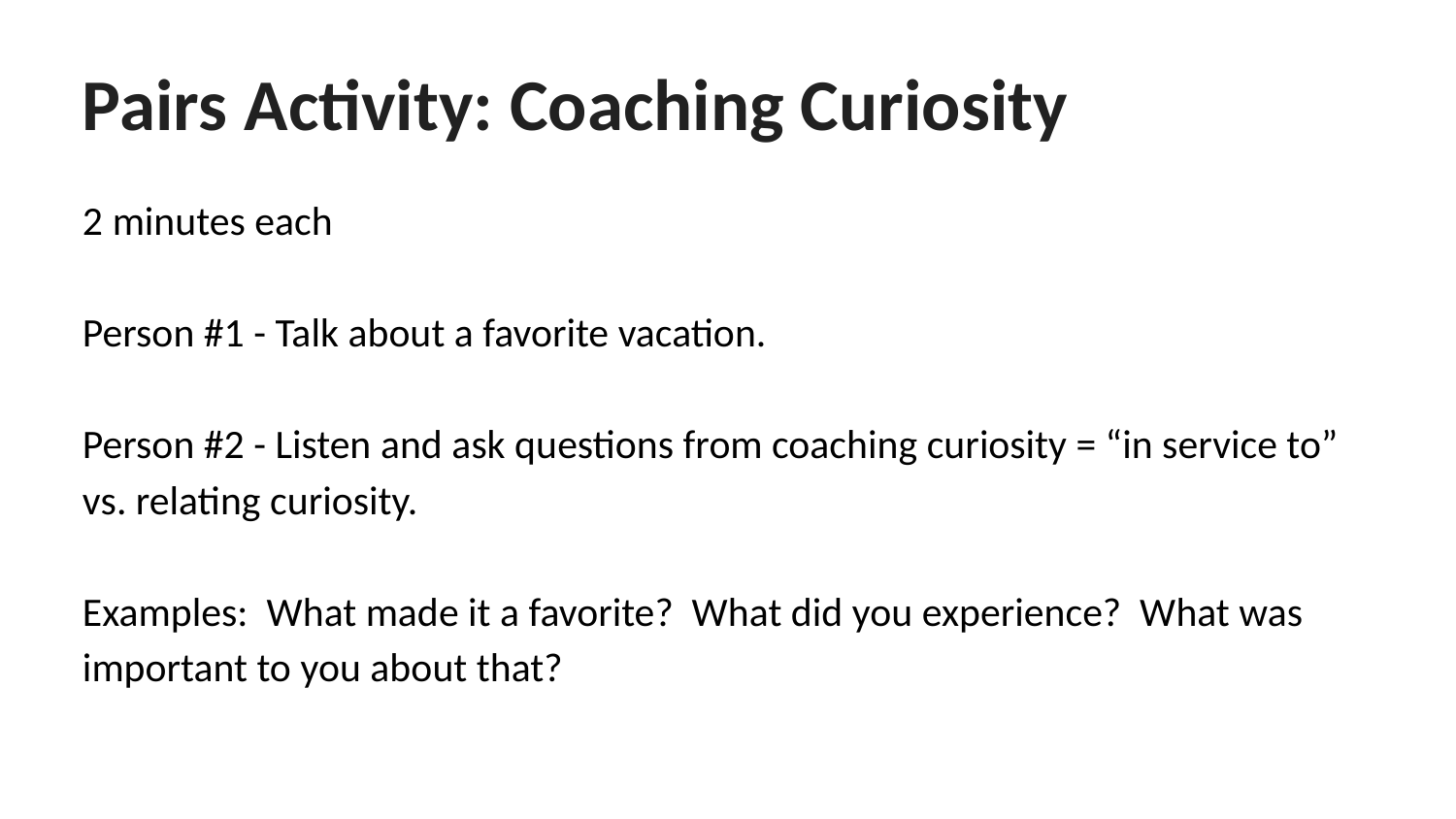

# Pairs Activity: Coaching Curiosity
2 minutes each
Person #1 - Talk about a favorite vacation.
Person #2 - Listen and ask questions from coaching curiosity = “in service to” vs. relating curiosity.
Examples: What made it a favorite? What did you experience? What was important to you about that?

## Slide 6
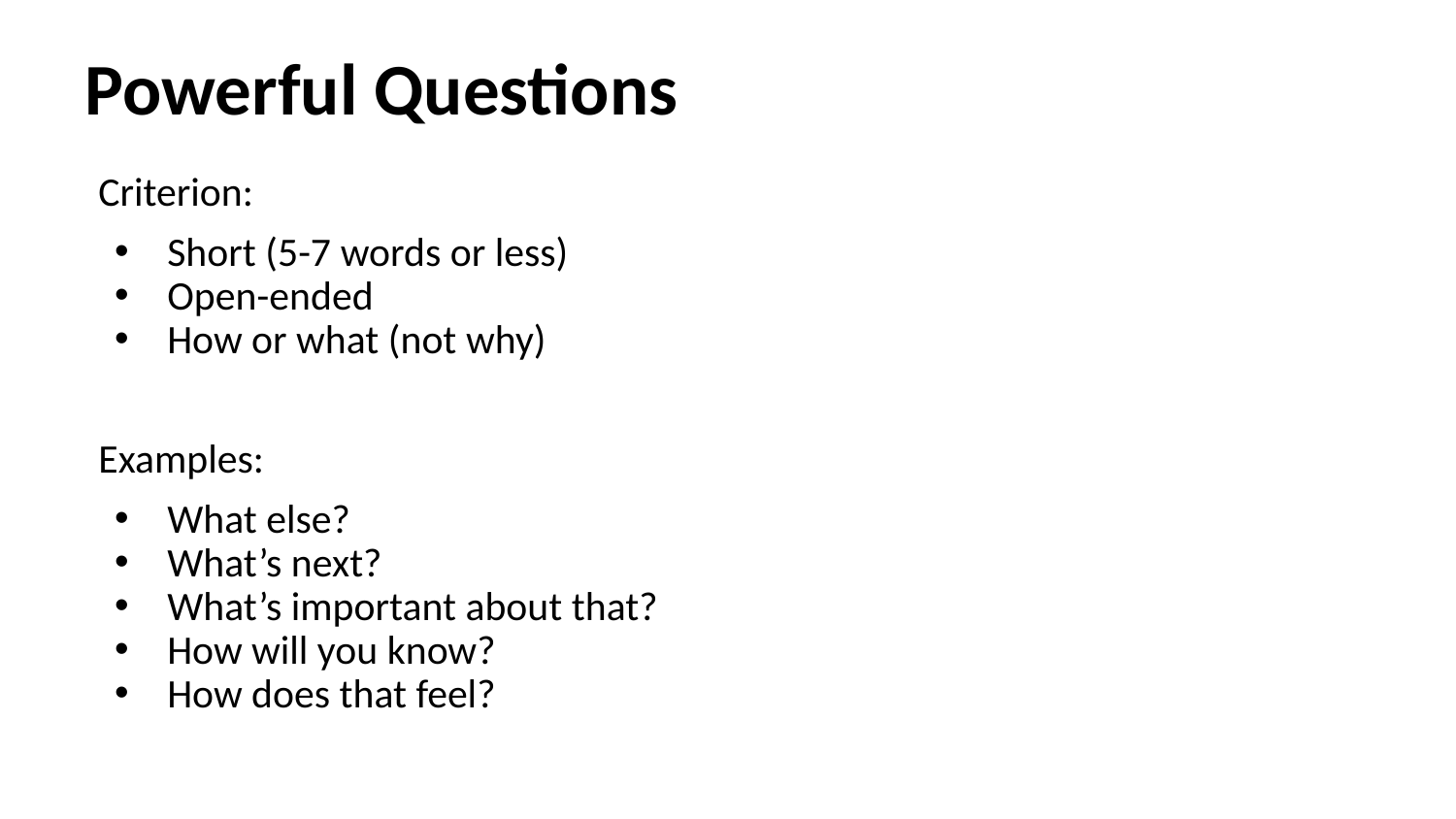

# Powerful Questions
Criterion:
Short (5-7 words or less)
Open-ended
How or what (not why)
Examples:
What else?
What’s next?
What’s important about that?
How will you know?
How does that feel?

## Slide 7
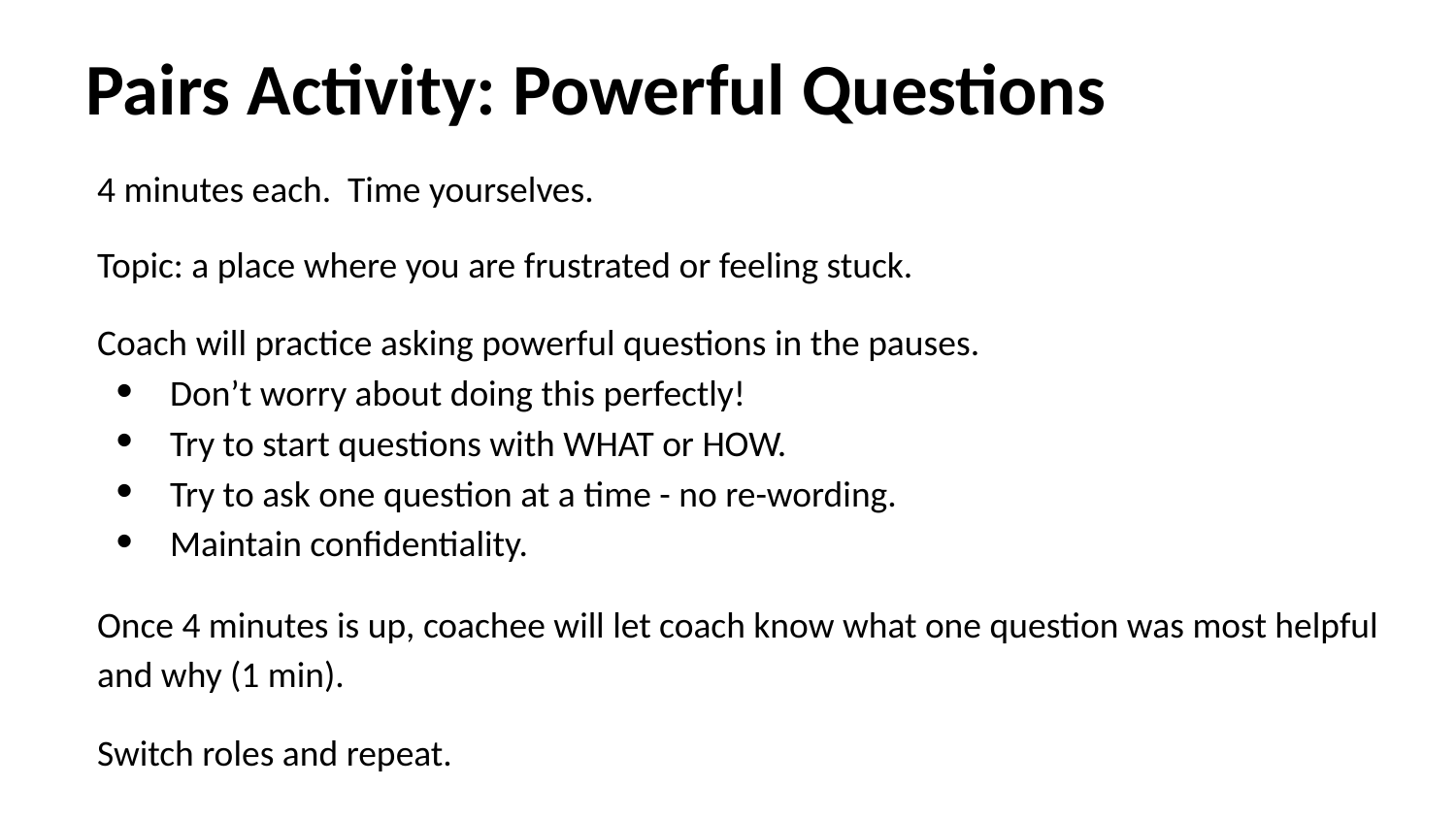

# Pairs Activity: Powerful Questions
4 minutes each. Time yourselves.
Topic: a place where you are frustrated or feeling stuck.
Coach will practice asking powerful questions in the pauses.
Don’t worry about doing this perfectly!
Try to start questions with WHAT or HOW.
Try to ask one question at a time - no re-wording.
Maintain confidentiality.
Once 4 minutes is up, coachee will let coach know what one question was most helpful and why (1 min).
Switch roles and repeat.

## Slide 8
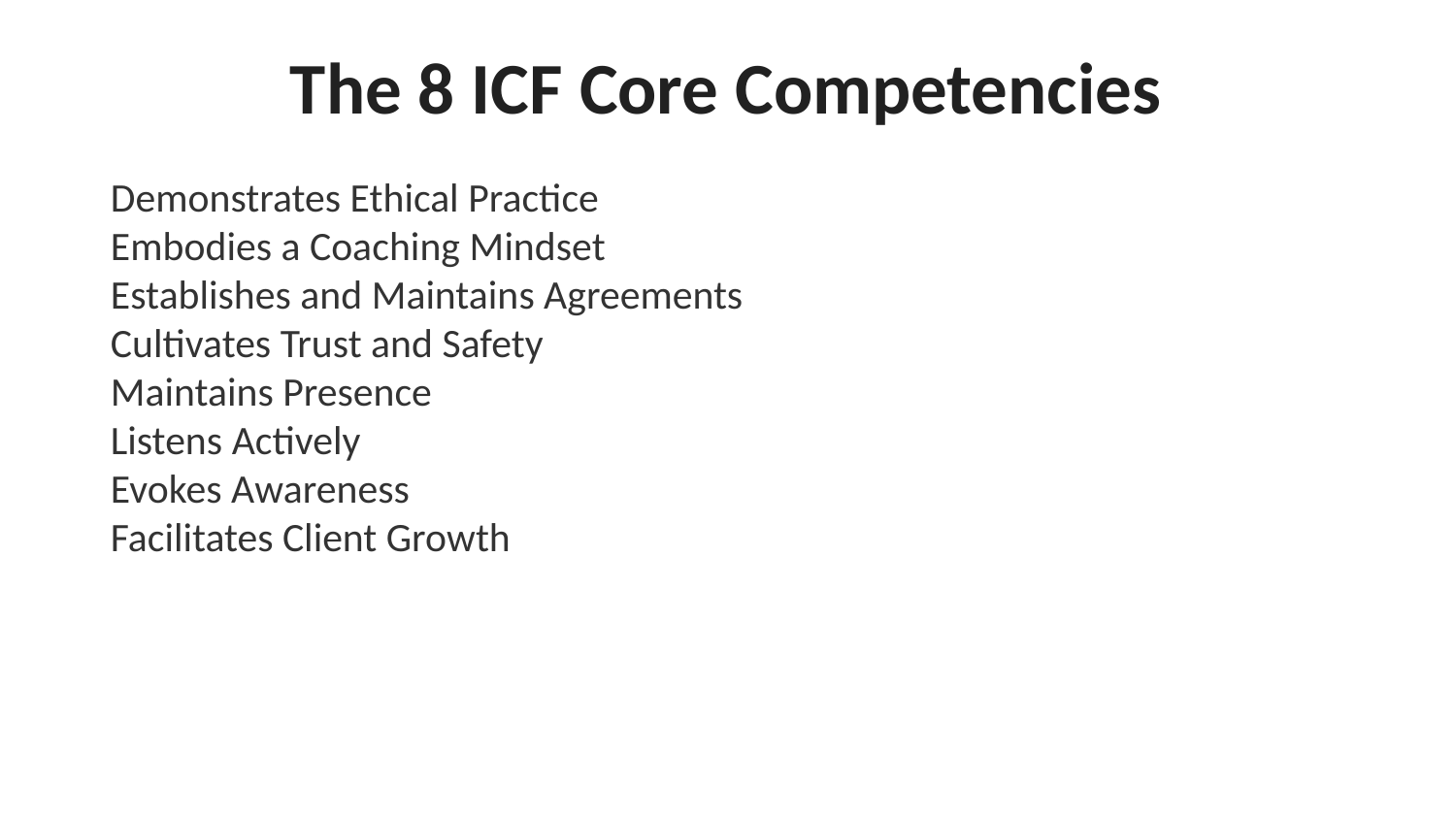

# The 8 ICF Core Competencies
Demonstrates Ethical Practice
Embodies a Coaching Mindset
Establishes and Maintains Agreements
Cultivates Trust and Safety
Maintains Presence
Listens Actively
Evokes Awareness
Facilitates Client Growth

## Slide 9
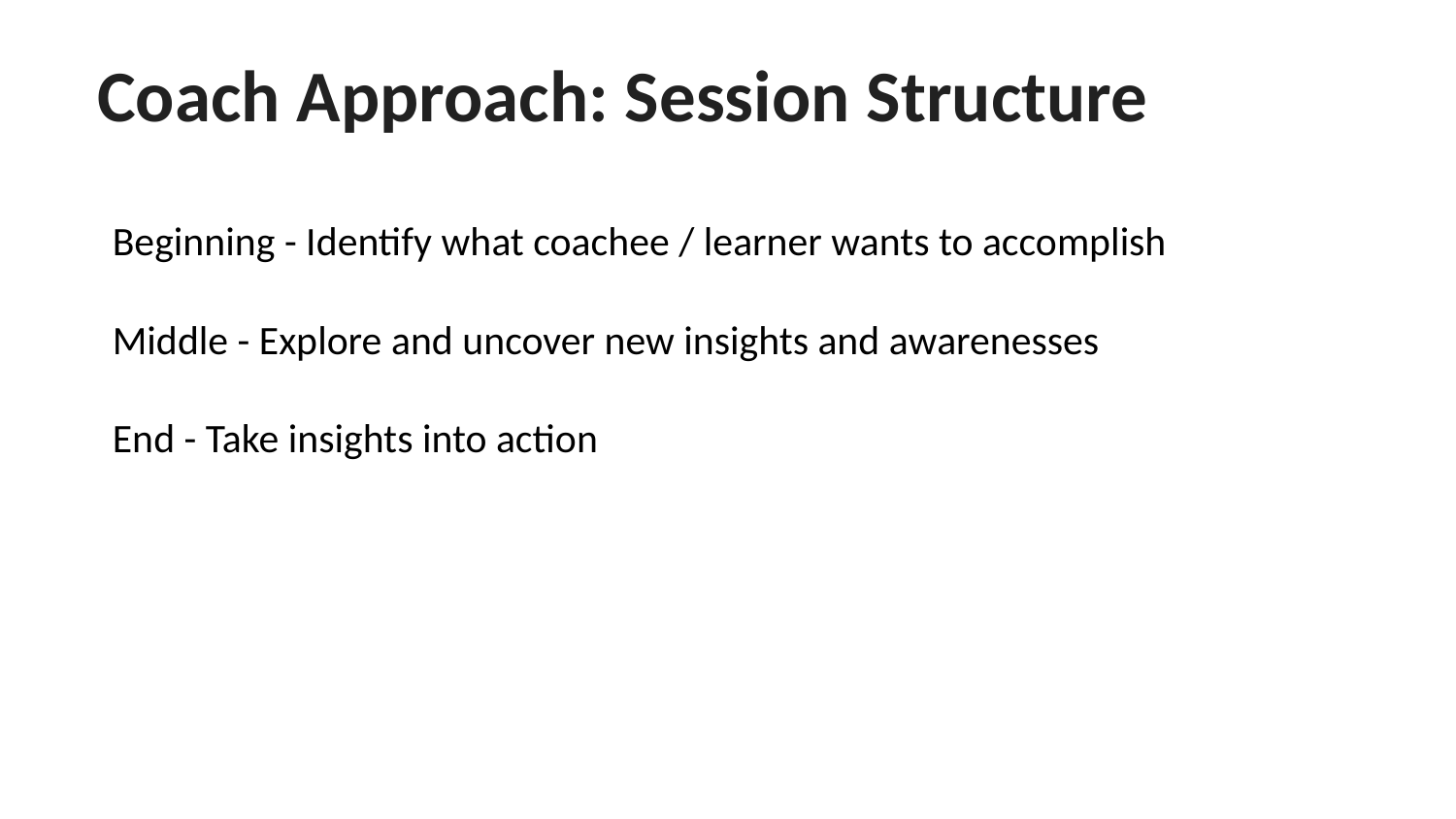

# Coach Approach: Session Structure
Beginning - Identify what coachee / learner wants to accomplish
Middle - Explore and uncover new insights and awarenesses
End - Take insights into action

## Slide 10
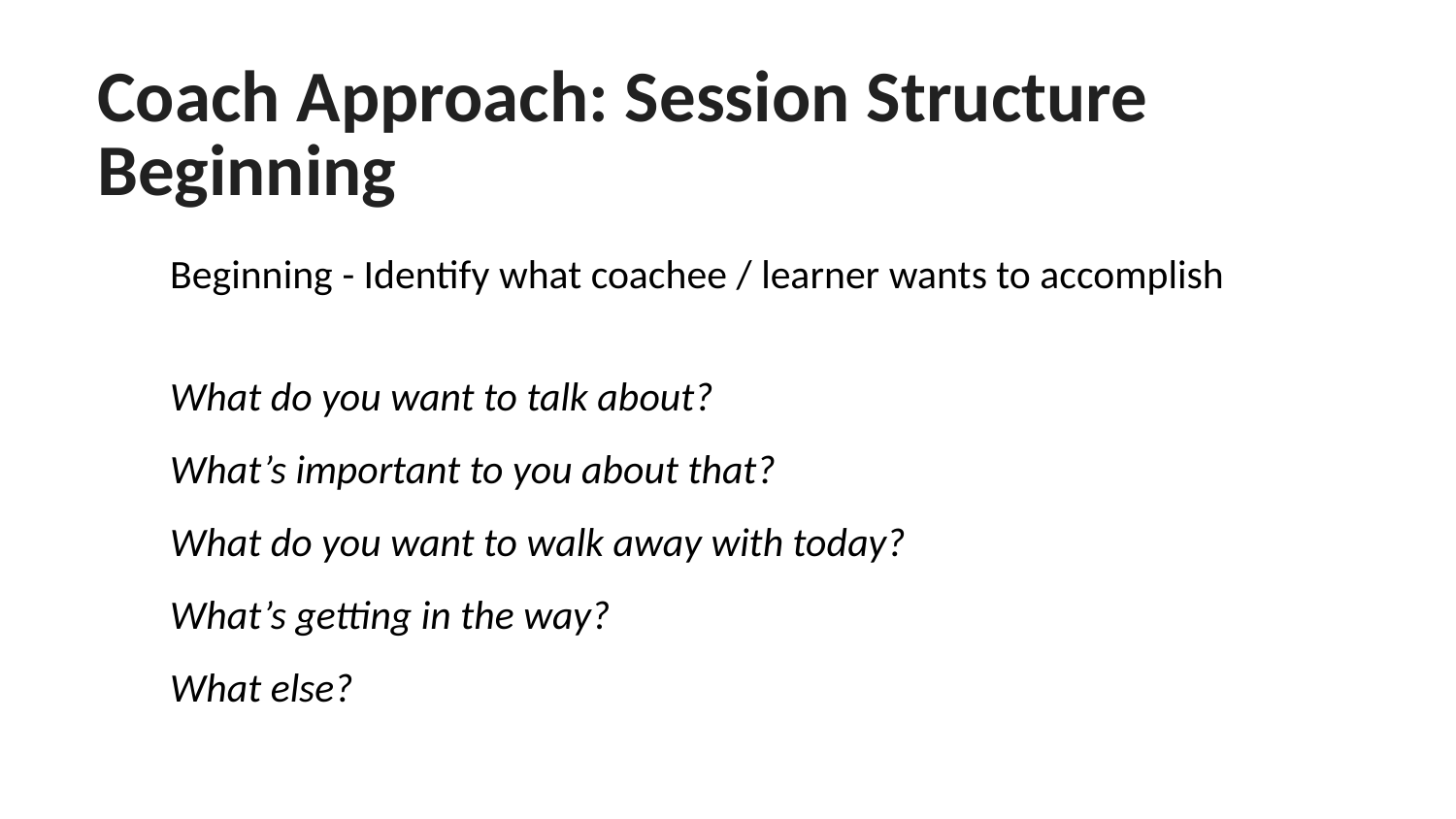

# Coach Approach: Session Structure Beginning
Beginning - Identify what coachee / learner wants to accomplish
What do you want to talk about?
What’s important to you about that?
What do you want to walk away with today?
What’s getting in the way?
What else?

## Slide 11
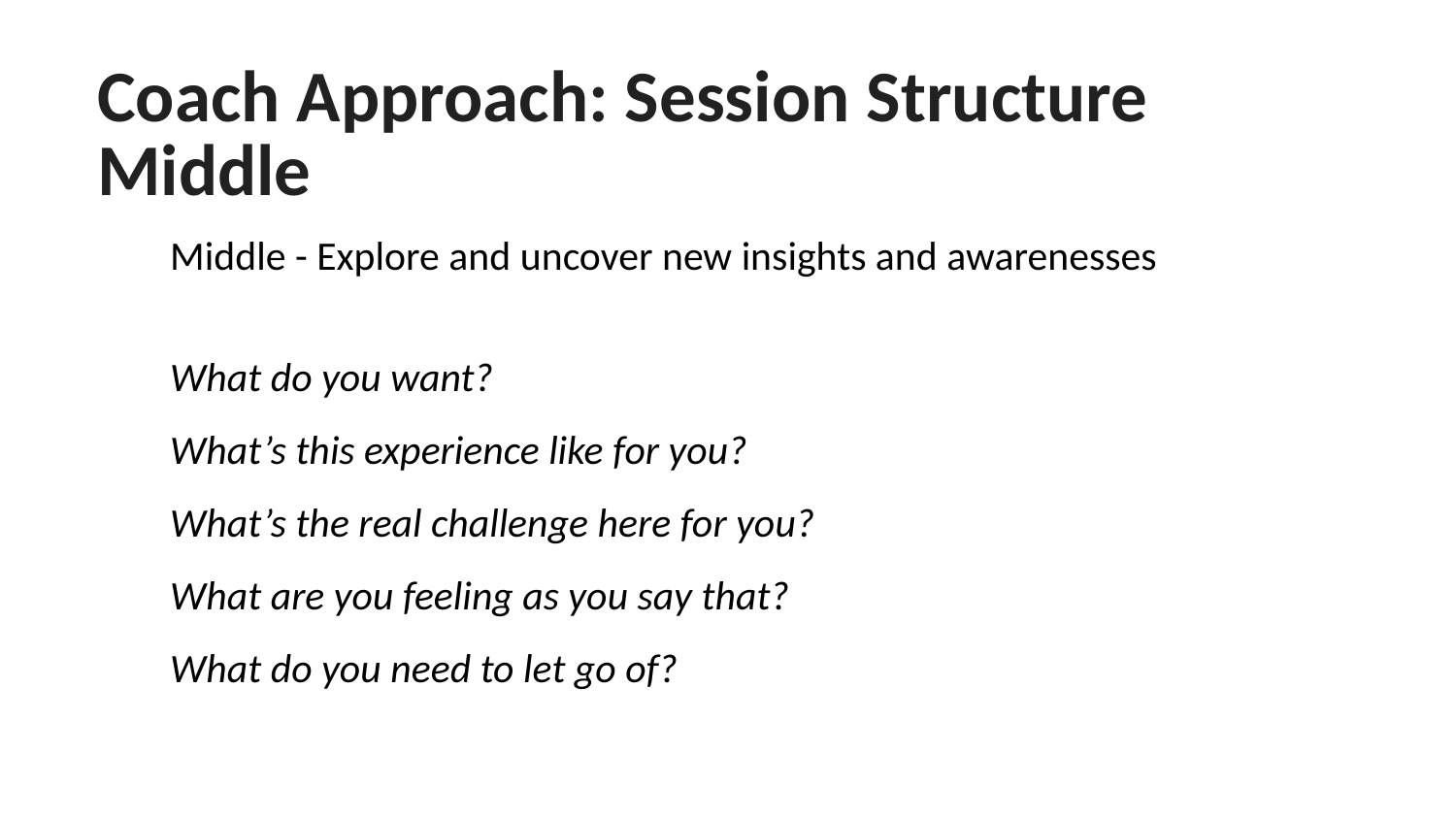

# Coach Approach: Session Structure Middle
Middle - Explore and uncover new insights and awarenesses
What do you want?
What’s this experience like for you?
What’s the real challenge here for you?
What are you feeling as you say that?
What do you need to let go of?

## Slide 12
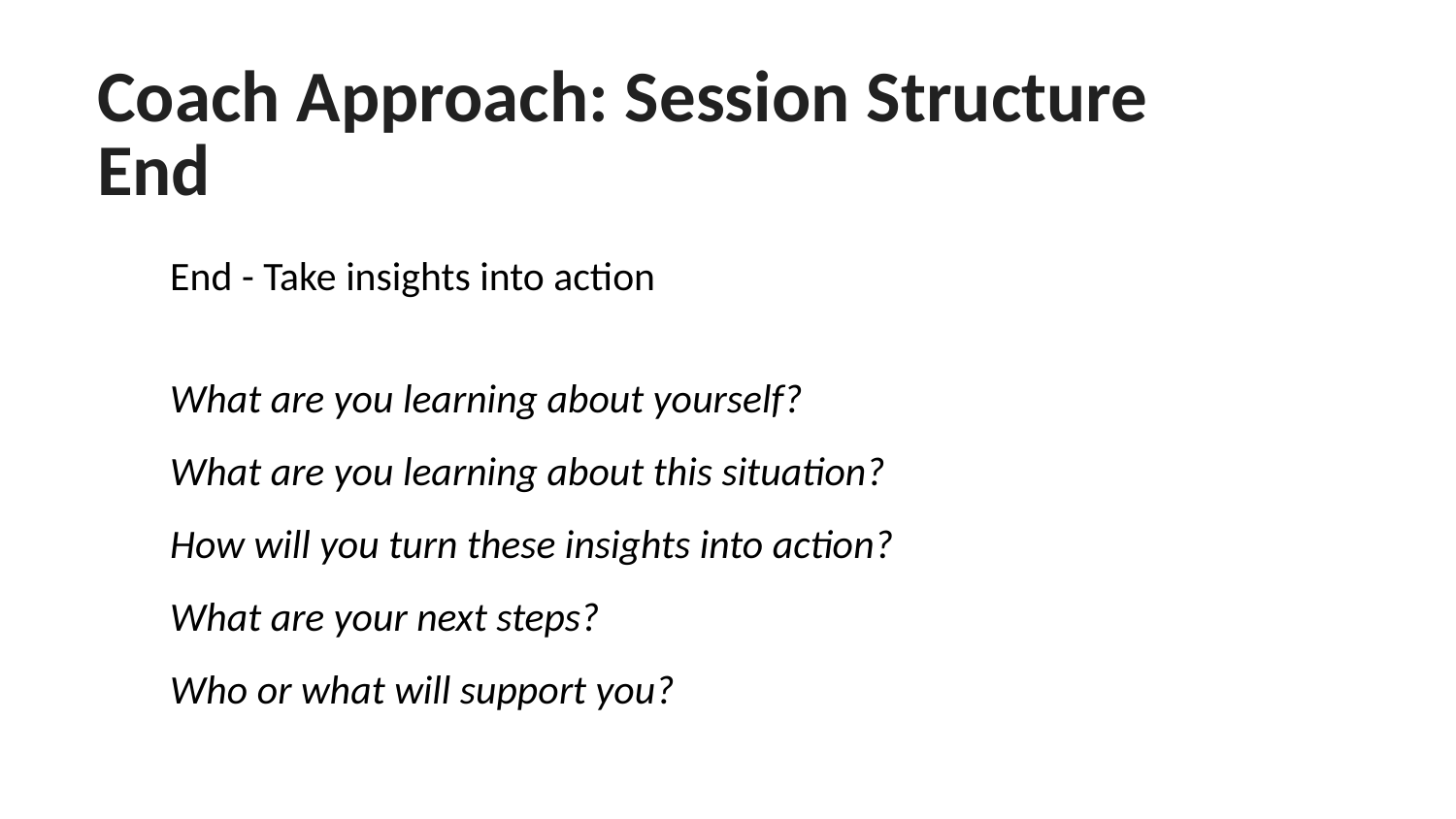

# Coach Approach: Session Structure End
End - Take insights into action
What are you learning about yourself?
What are you learning about this situation?
How will you turn these insights into action?
What are your next steps?
Who or what will support you?

## Slide 13
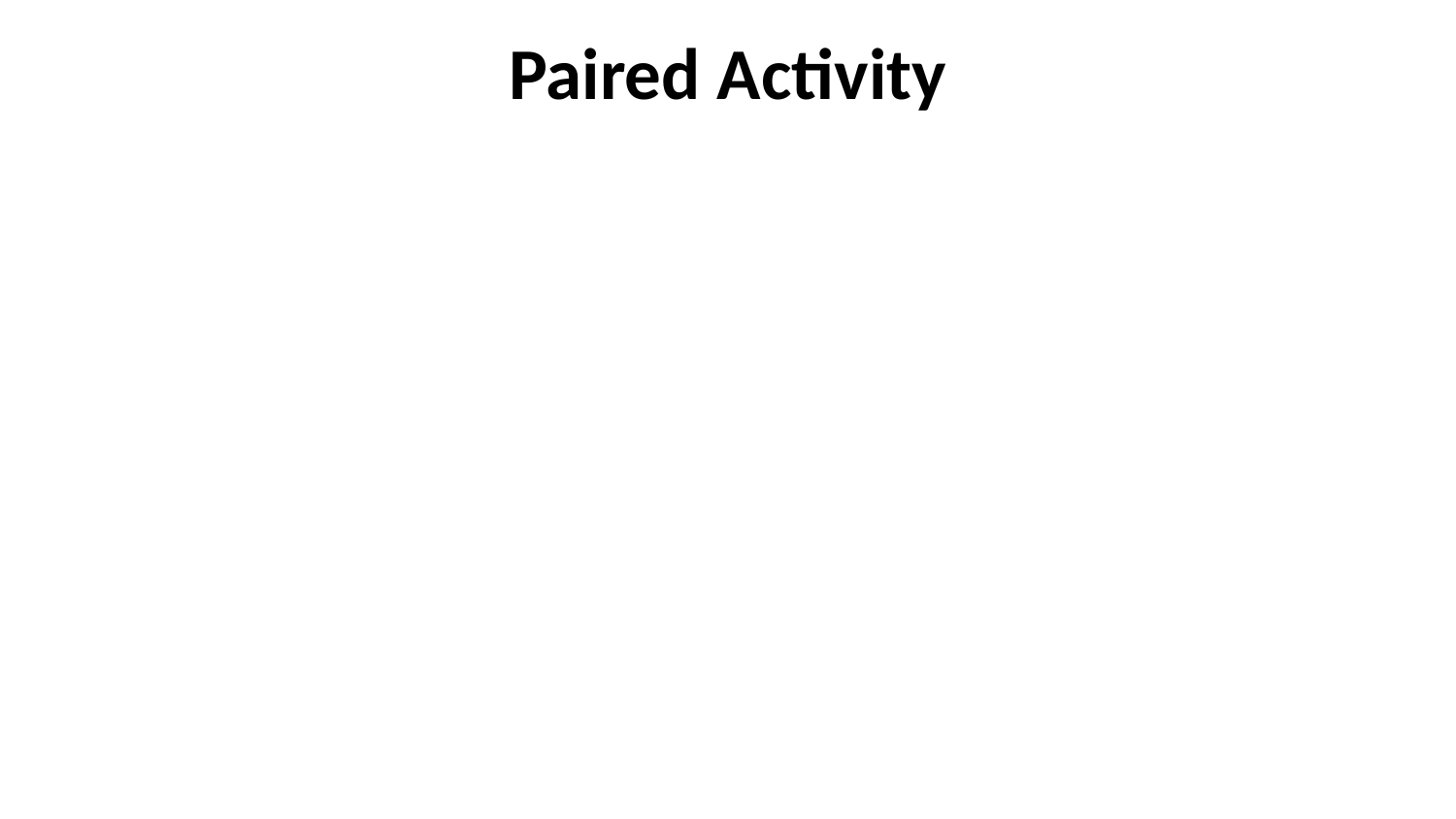

# Paired Activity

## Slide 14
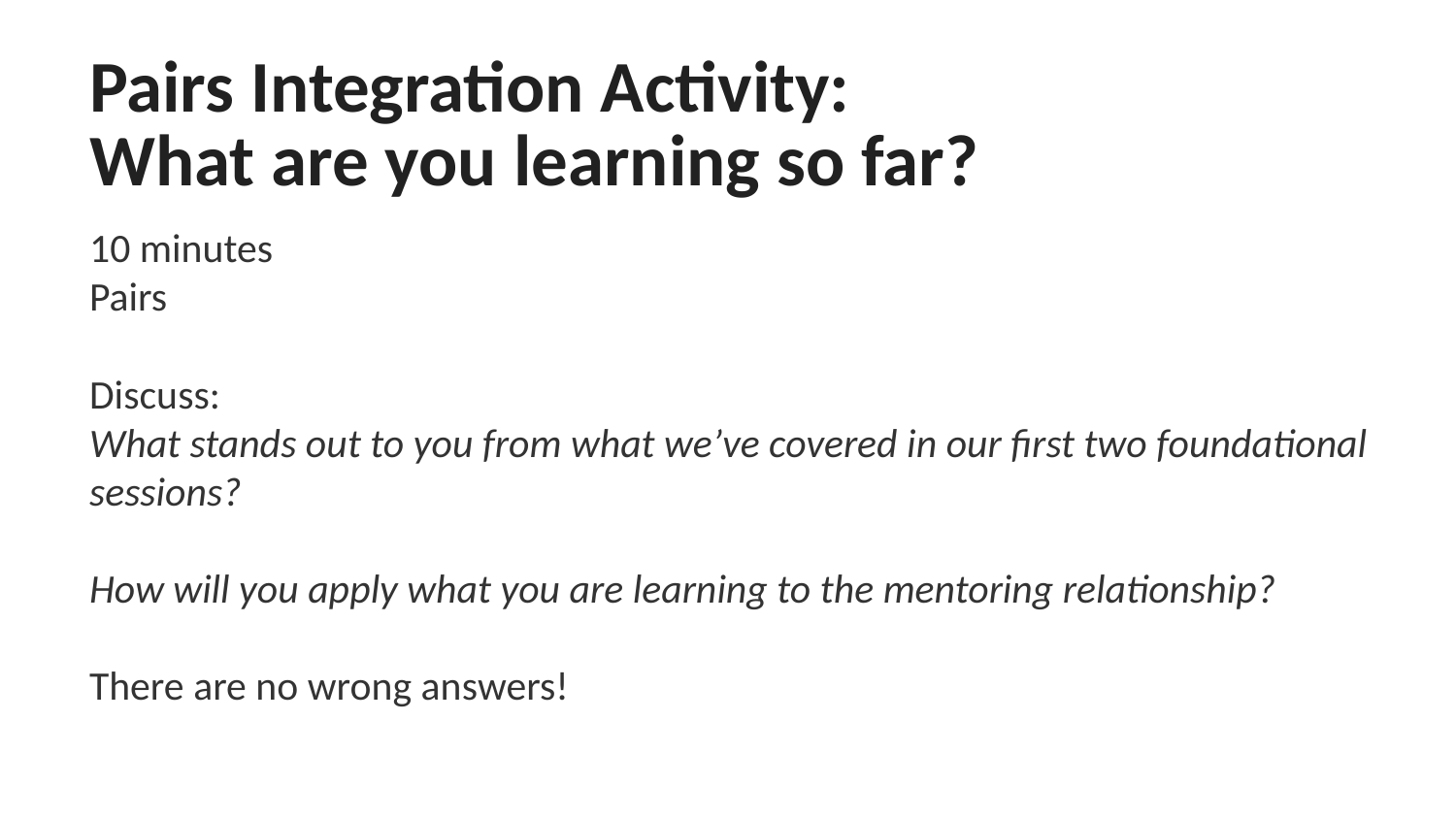

# Pairs Integration Activity:
What are you learning so far?
10 minutes
Pairs
Discuss:
What stands out to you from what we’ve covered in our first two foundational sessions?
How will you apply what you are learning to the mentoring relationship?
There are no wrong answers!

## Slide 15
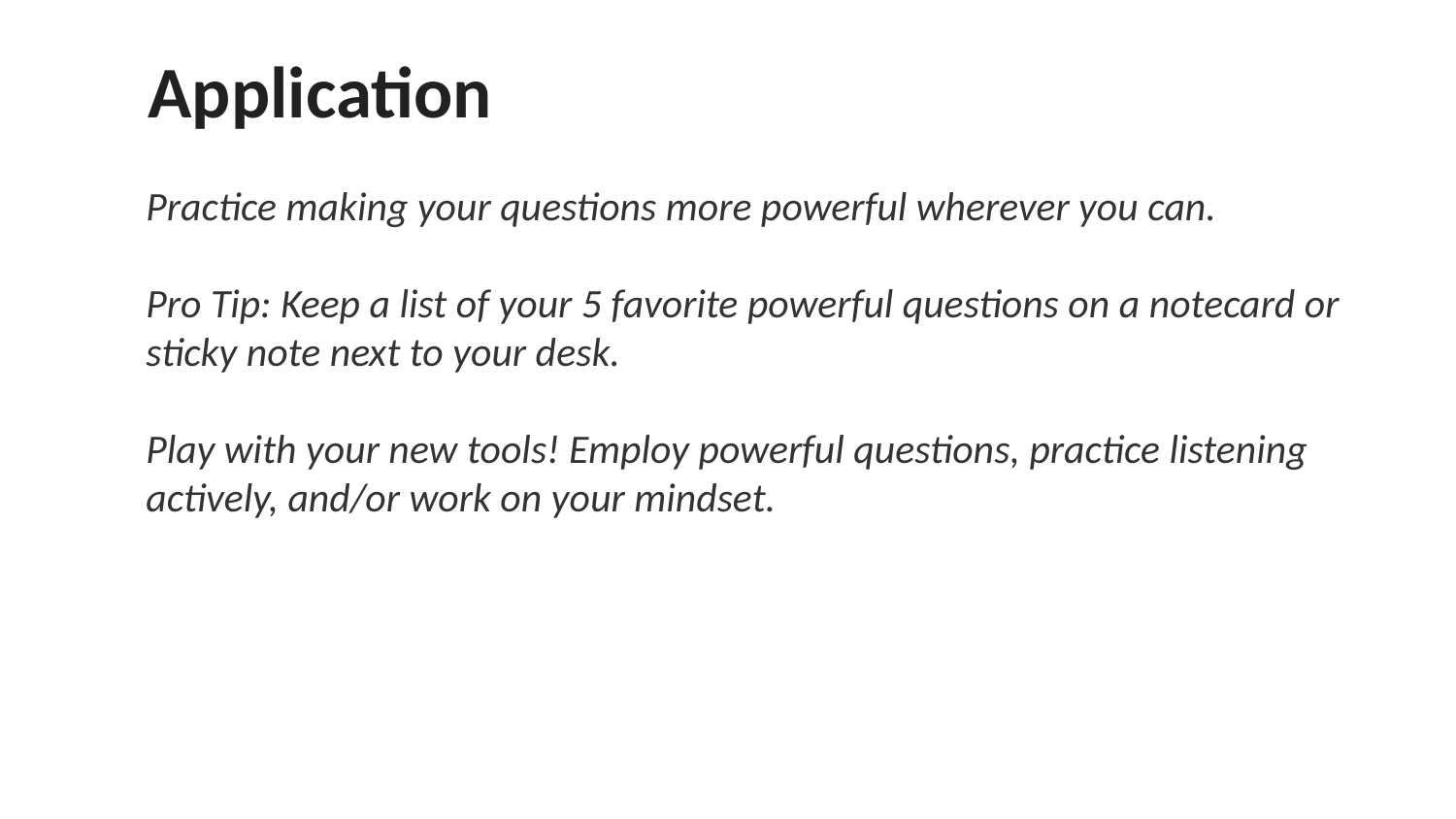

# Application
Practice making your questions more powerful wherever you can.
Pro Tip: Keep a list of your 5 favorite powerful questions on a notecard or sticky note next to your desk.
Play with your new tools! Employ powerful questions, practice listening actively, and/or work on your mindset.
