# Supplementary material for: A Longitudinal, Practical Curriculum for Faculty Development as New Coaches in Graduate Medical Education
Source: J Educ Teach Emerg Med. 2025 Jul 31;10(3):C1–C92. doi: 10.21980/J88M08 (PMC12320991; doi:10.21980/J88M08)
Supplement: Supplementary file 3 [file 10-3-C1-SuppF2.pptx]

## Slide 1
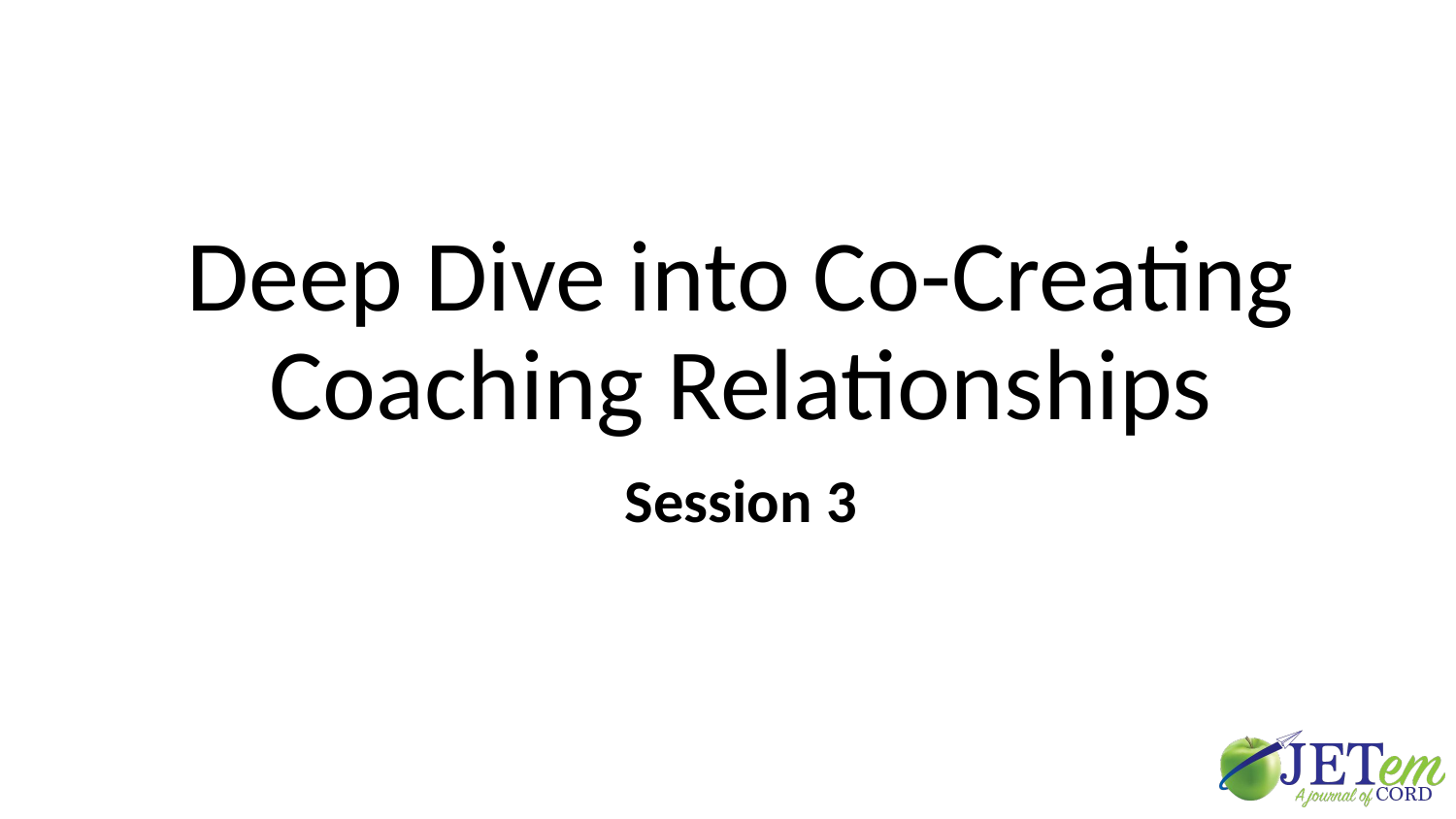

Deep Dive into Co-Creating Coaching Relationships
Session 3

## Slide 2
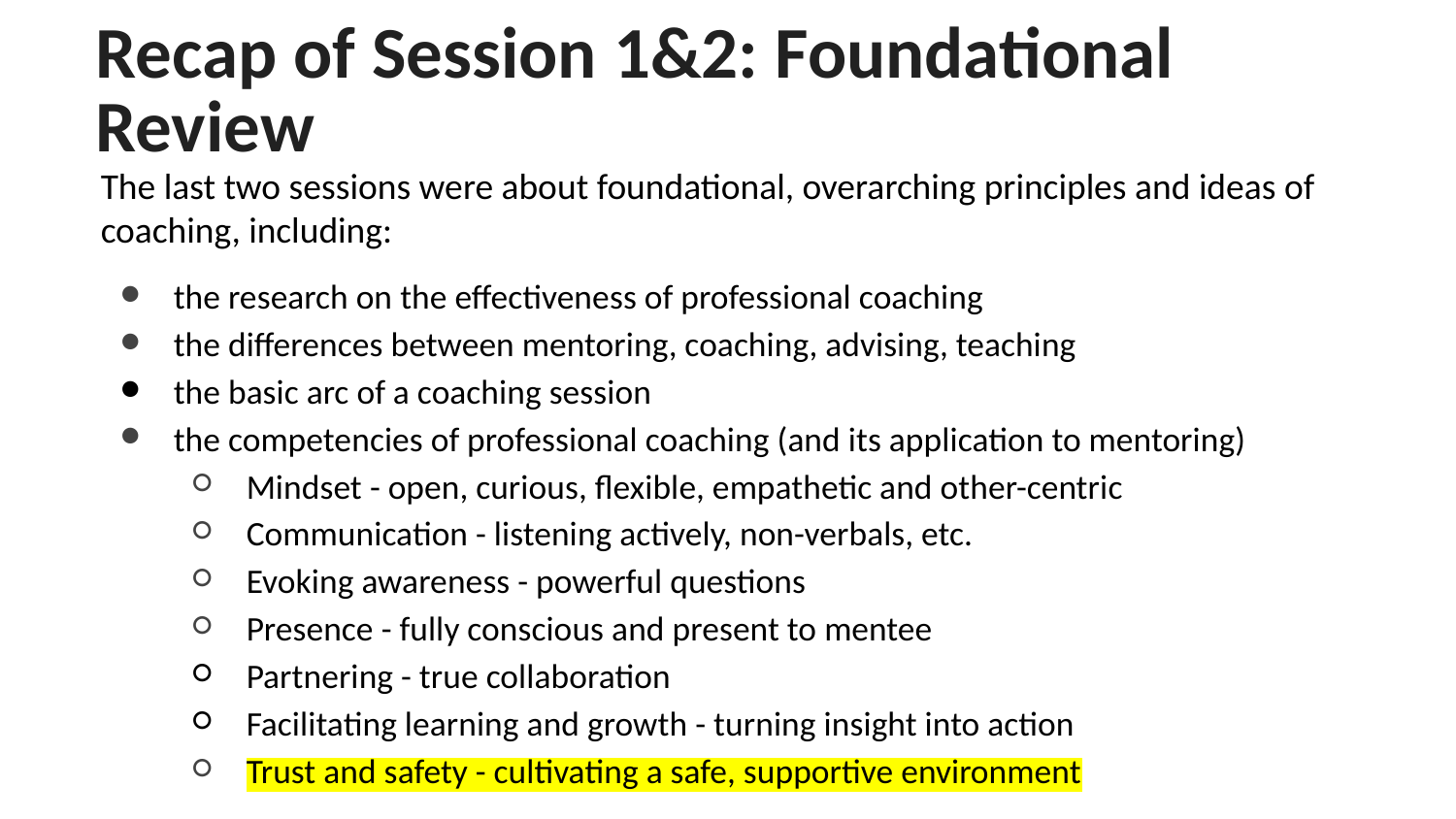

# Recap of Session 1&2: Foundational Review
The last two sessions were about foundational, overarching principles and ideas of coaching, including:
the research on the effectiveness of professional coaching
the differences between mentoring, coaching, advising, teaching
the basic arc of a coaching session
the competencies of professional coaching (and its application to mentoring)
Mindset - open, curious, flexible, empathetic and other-centric
Communication - listening actively, non-verbals, etc.
Evoking awareness - powerful questions
Presence - fully conscious and present to mentee
Partnering - true collaboration
Facilitating learning and growth - turning insight into action
Trust and safety - cultivating a safe, supportive environment

## Slide 3
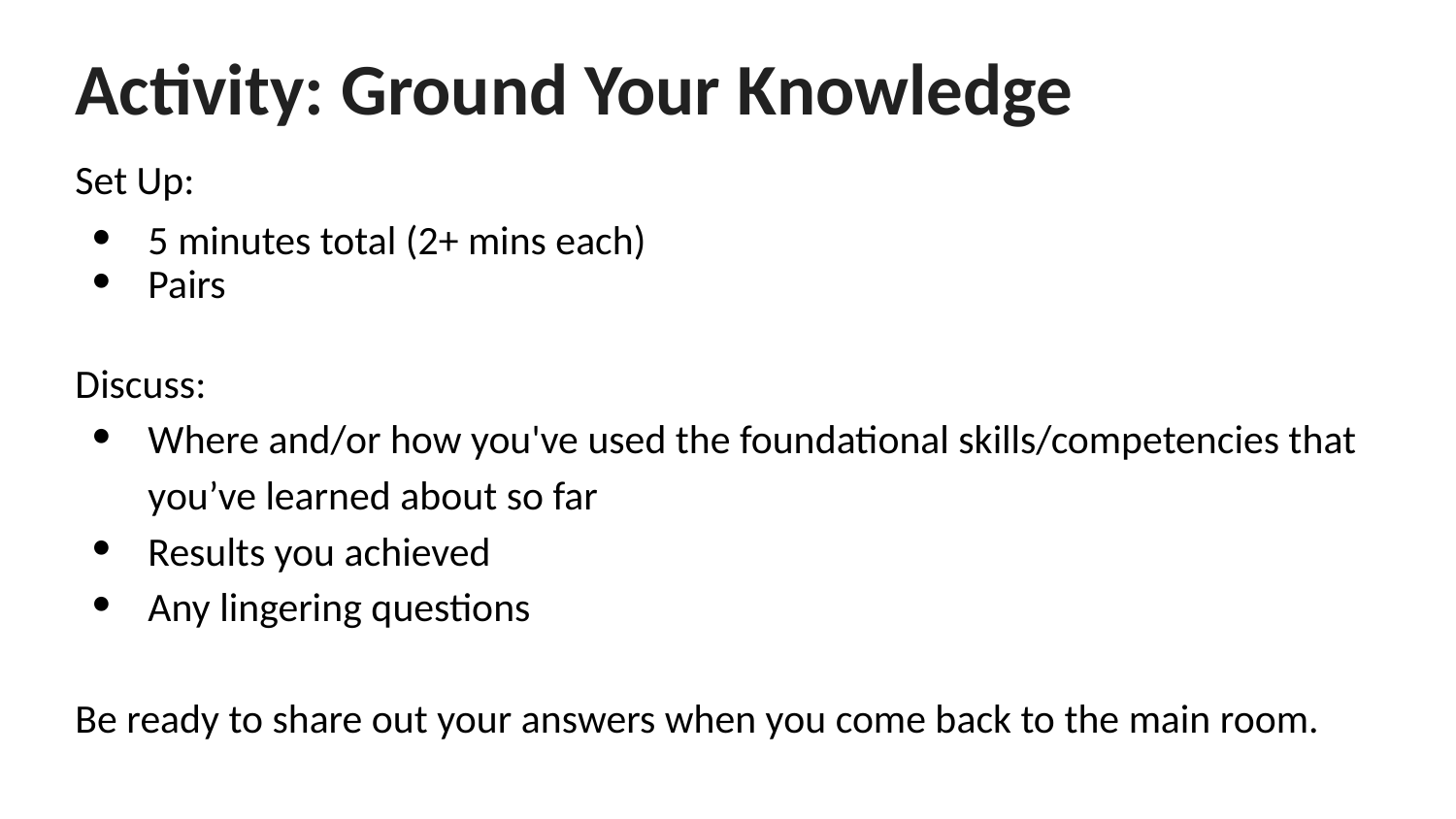

# Activity: Ground Your Knowledge
Set Up:
5 minutes total (2+ mins each)
Pairs
Discuss:
Where and/or how you've used the foundational skills/competencies that you’ve learned about so far
Results you achieved
Any lingering questions
Be ready to share out your answers when you come back to the main room.

## Slide 4
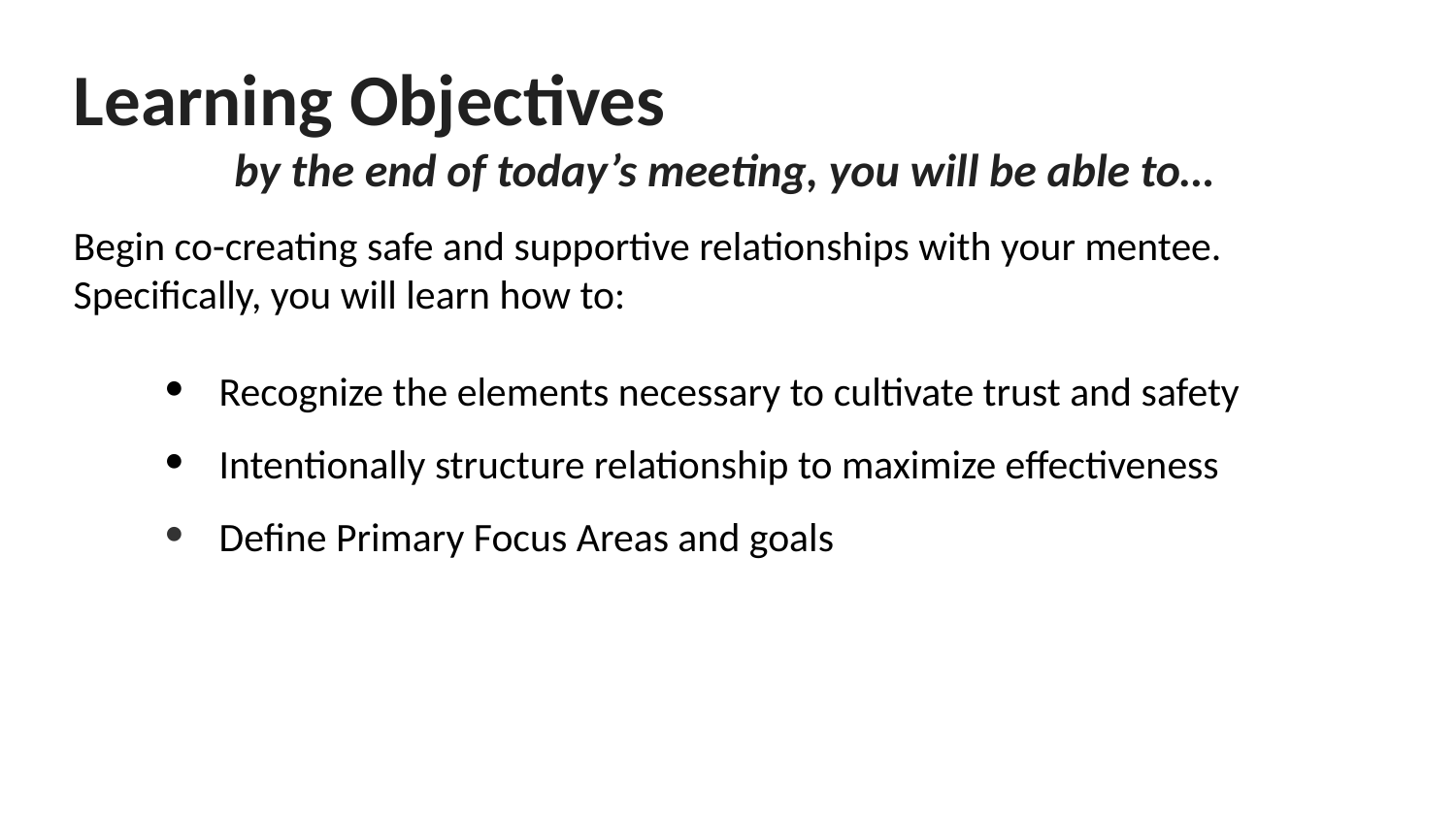

# Learning Objectives
by the end of today’s meeting, you will be able to…
Begin co-creating safe and supportive relationships with your mentee. Specifically, you will learn how to:
Recognize the elements necessary to cultivate trust and safety
Intentionally structure relationship to maximize effectiveness
Define Primary Focus Areas and goals

## Slide 5
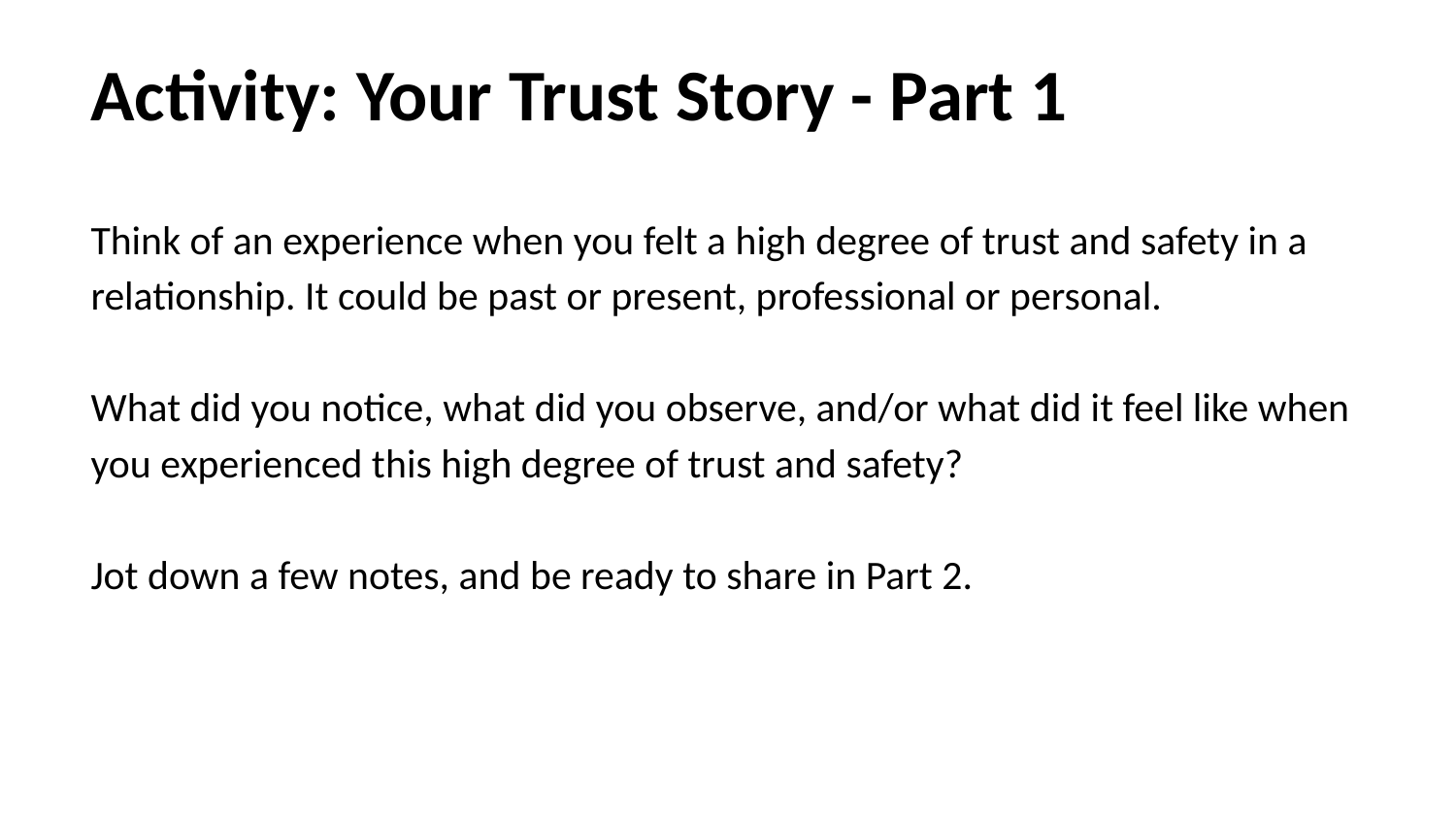

# Activity: Your Trust Story - Part 1
Think of an experience when you felt a high degree of trust and safety in a relationship. It could be past or present, professional or personal.
What did you notice, what did you observe, and/or what did it feel like when you experienced this high degree of trust and safety?
Jot down a few notes, and be ready to share in Part 2.

## Slide 6
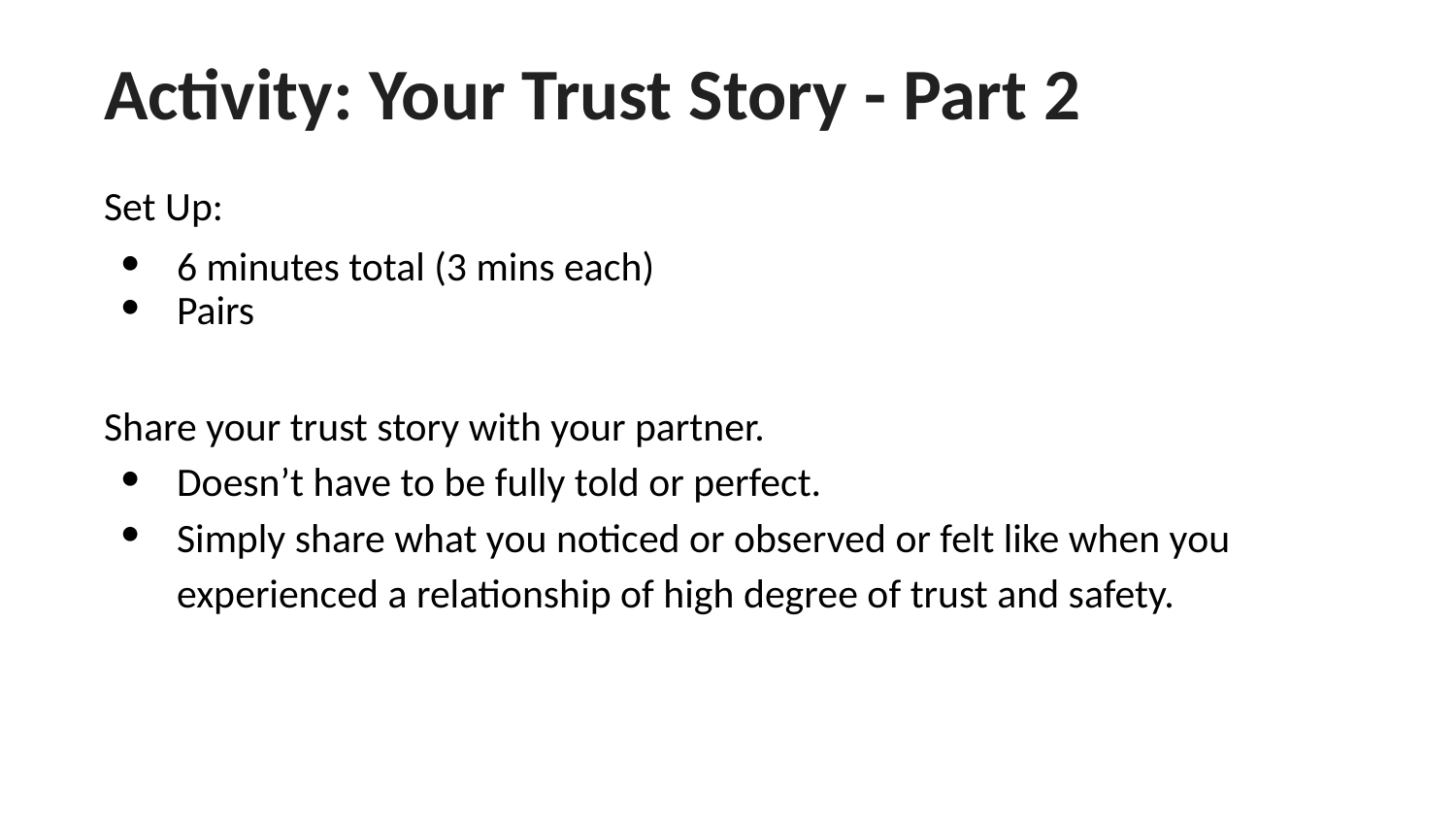

# Activity: Your Trust Story - Part 2
Set Up:
6 minutes total (3 mins each)
Pairs
Share your trust story with your partner.
Doesn’t have to be fully told or perfect.
Simply share what you noticed or observed or felt like when you experienced a relationship of high degree of trust and safety.

## Slide 7
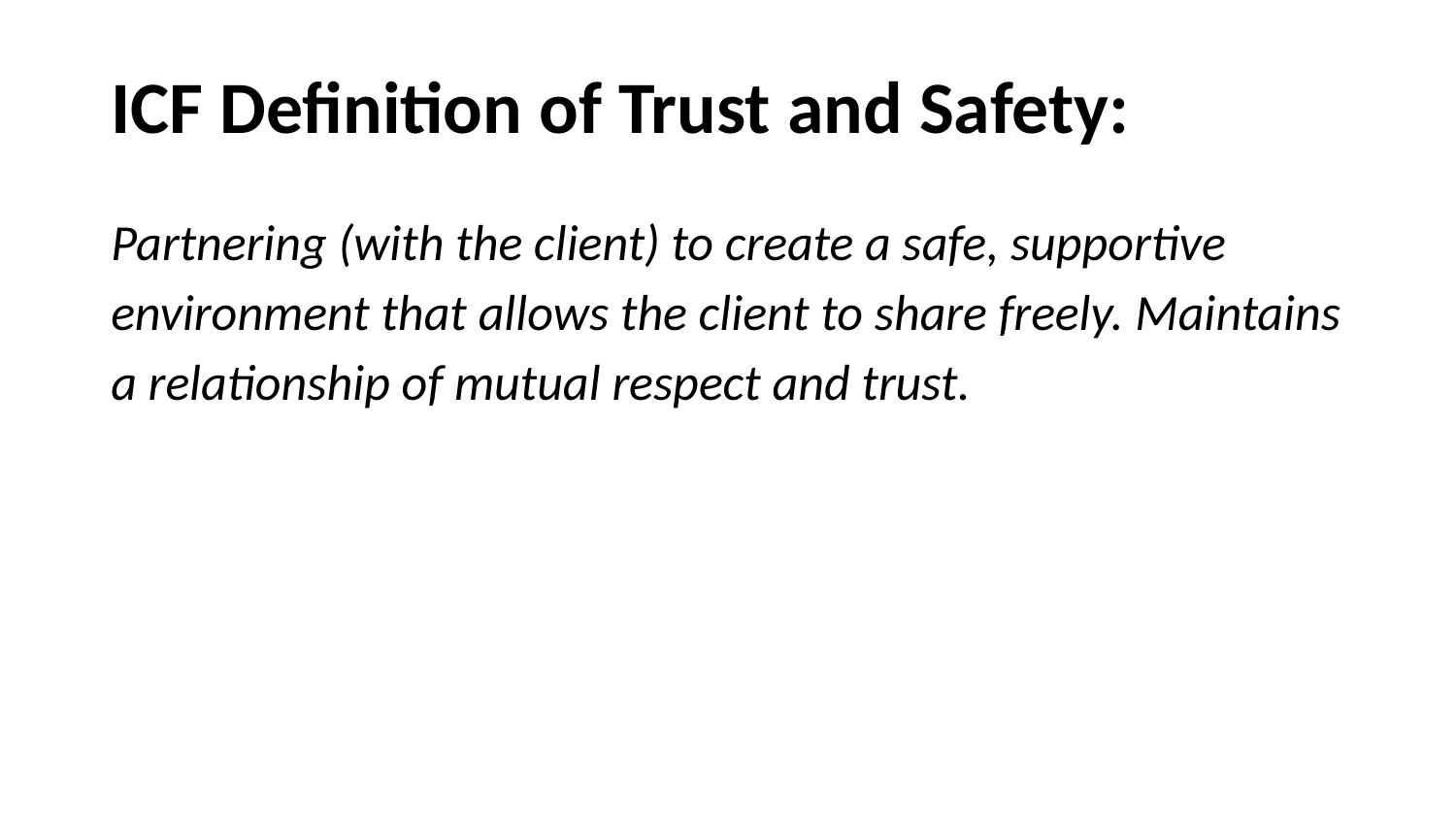

# ICF Definition of Trust and Safety:
Partnering (with the client) to create a safe, supportive environment that allows the client to share freely. Maintains a relationship of mutual respect and trust.

## Slide 8
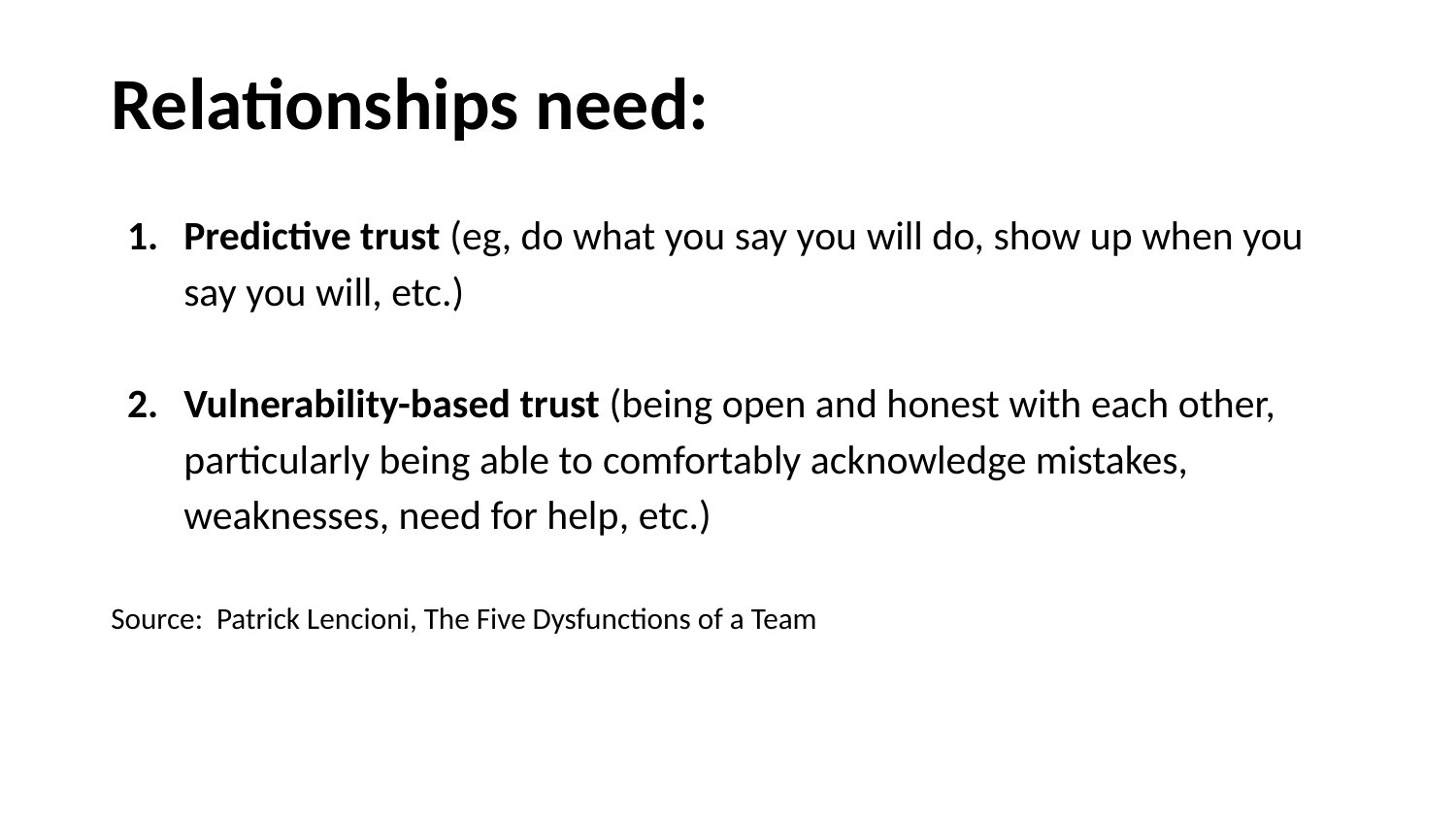

# Relationships need:
Predictive trust (eg, do what you say you will do, show up when you say you will, etc.)
Vulnerability-based trust (being open and honest with each other, particularly being able to comfortably acknowledge mistakes, weaknesses, need for help, etc.)
Source: Patrick Lencioni, The Five Dysfunctions of a Team

## Slide 9
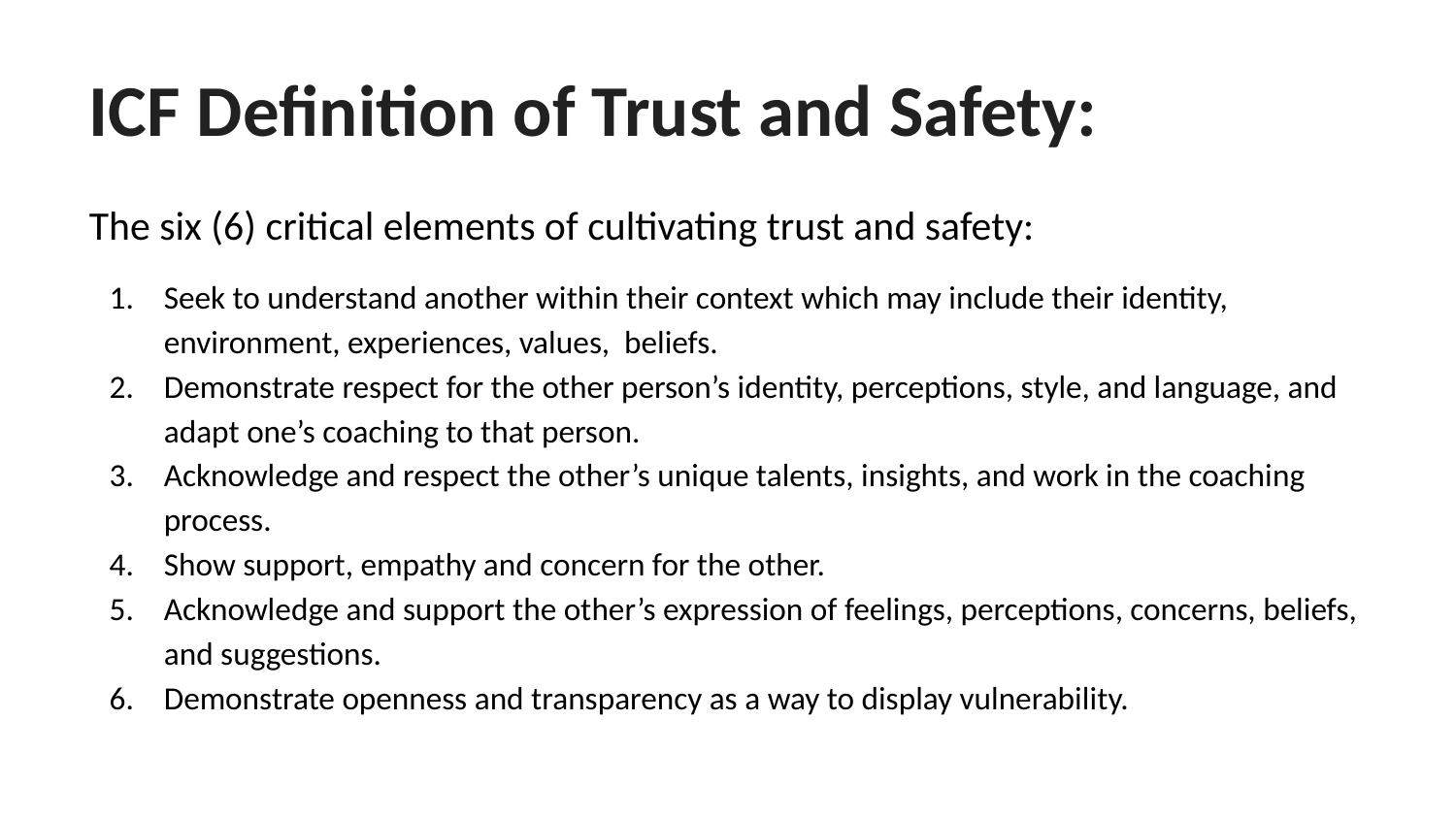

# ICF Definition of Trust and Safety:
The six (6) critical elements of cultivating trust and safety:
Seek to understand another within their context which may include their identity, environment, experiences, values, beliefs.
Demonstrate respect for the other person’s identity, perceptions, style, and language, and adapt one’s coaching to that person.
Acknowledge and respect the other’s unique talents, insights, and work in the coaching process.
Show support, empathy and concern for the other.
Acknowledge and support the other’s expression of feelings, perceptions, concerns, beliefs, and suggestions.
Demonstrate openness and transparency as a way to display vulnerability.

## Slide 10
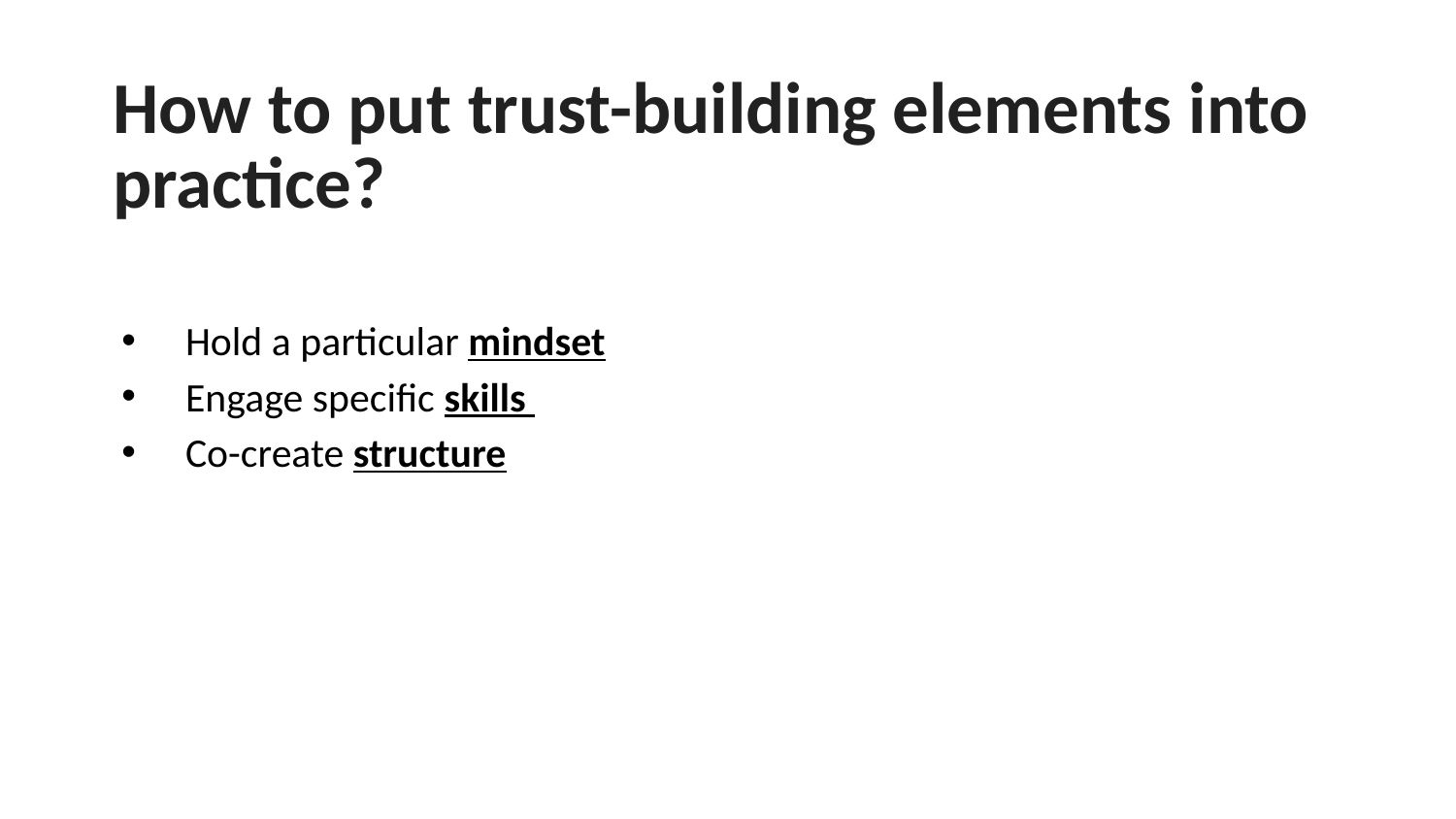

# How to put trust-building elements into practice?
Hold a particular mindset
Engage specific skills
Co-create structure

## Slide 11
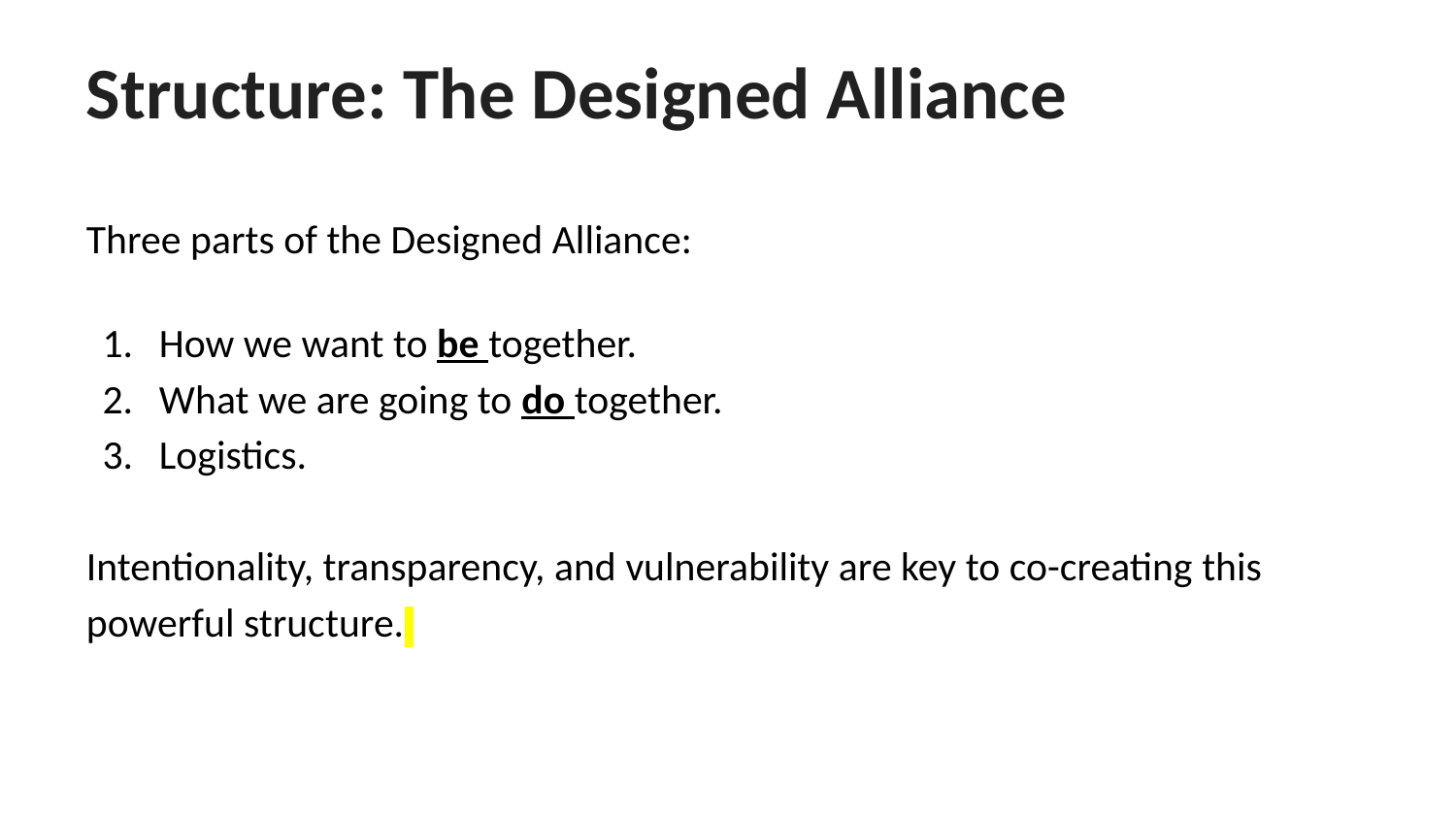

# Structure: The Designed Alliance
Three parts of the Designed Alliance:
How we want to be together.
What we are going to do together.
Logistics.
Intentionality, transparency, and vulnerability are key to co-creating this powerful structure.

## Slide 12
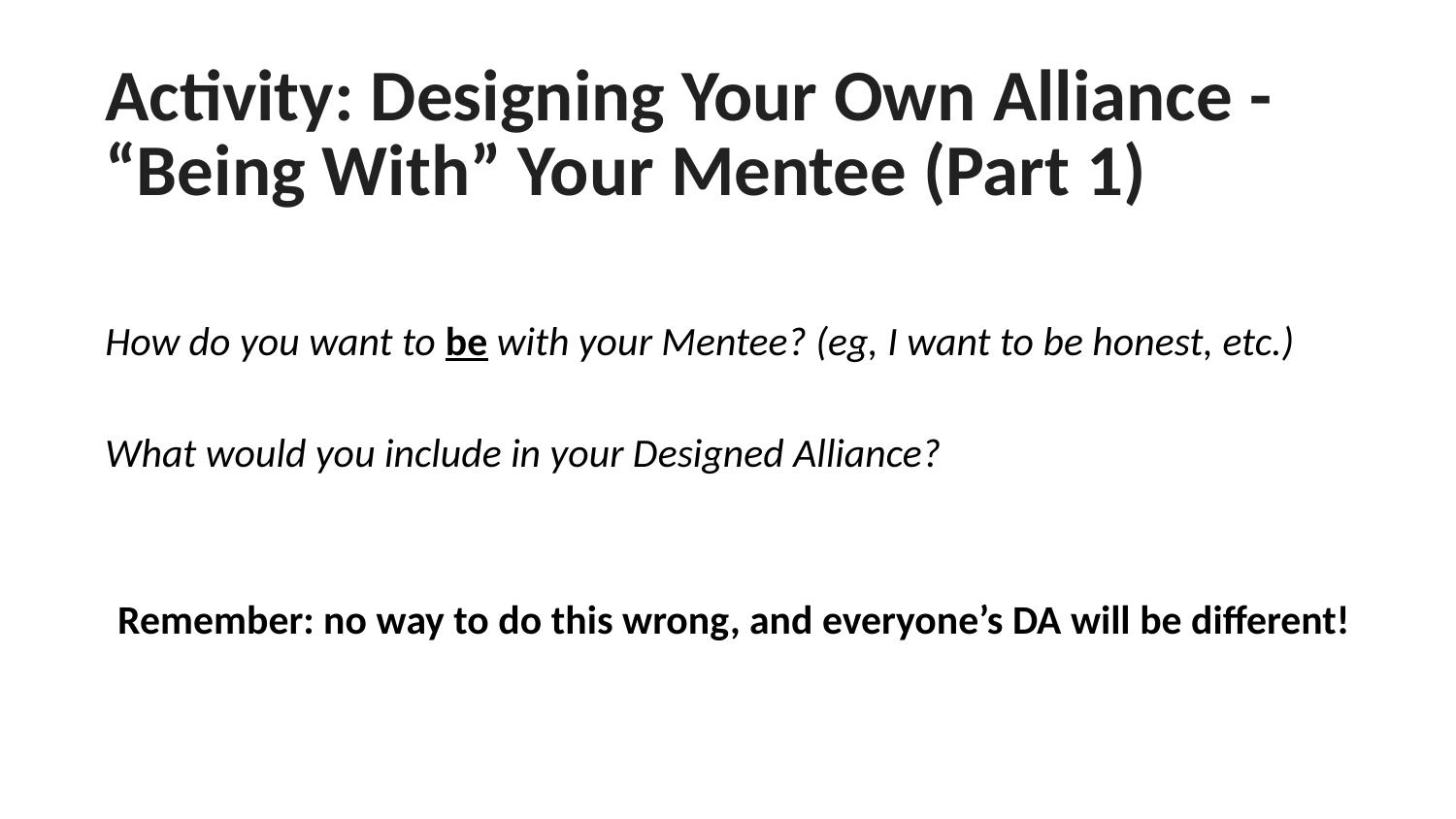

# Activity: Designing Your Own Alliance - “Being With” Your Mentee (Part 1)
How do you want to be with your Mentee? (eg, I want to be honest, etc.)
What would you include in your Designed Alliance?
Remember: no way to do this wrong, and everyone’s DA will be different!

## Slide 13
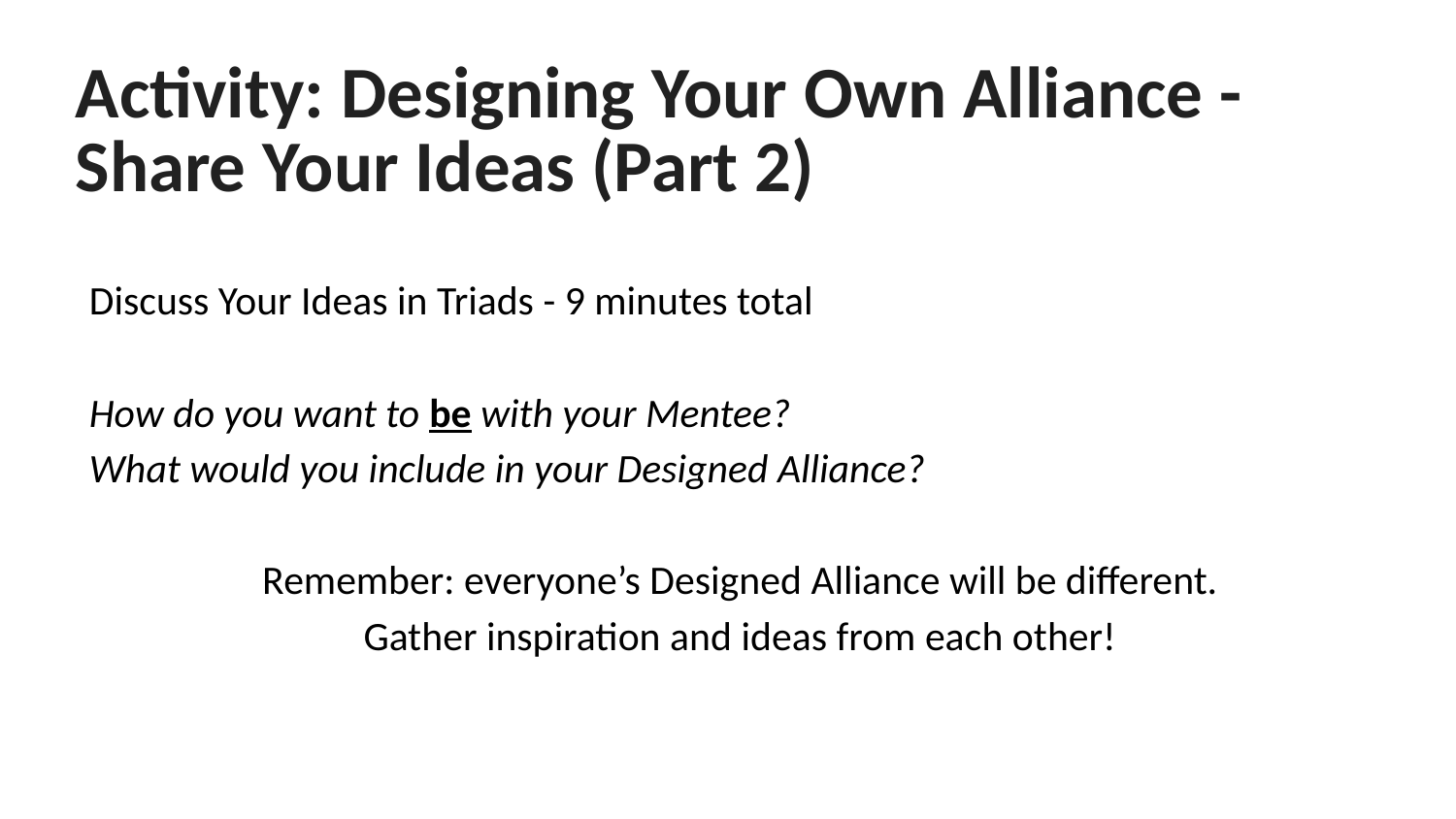

# Activity: Designing Your Own Alliance - Share Your Ideas (Part 2)
Discuss Your Ideas in Triads - 9 minutes total
How do you want to be with your Mentee?
What would you include in your Designed Alliance?
Remember: everyone’s Designed Alliance will be different.
Gather inspiration and ideas from each other!

## Slide 14
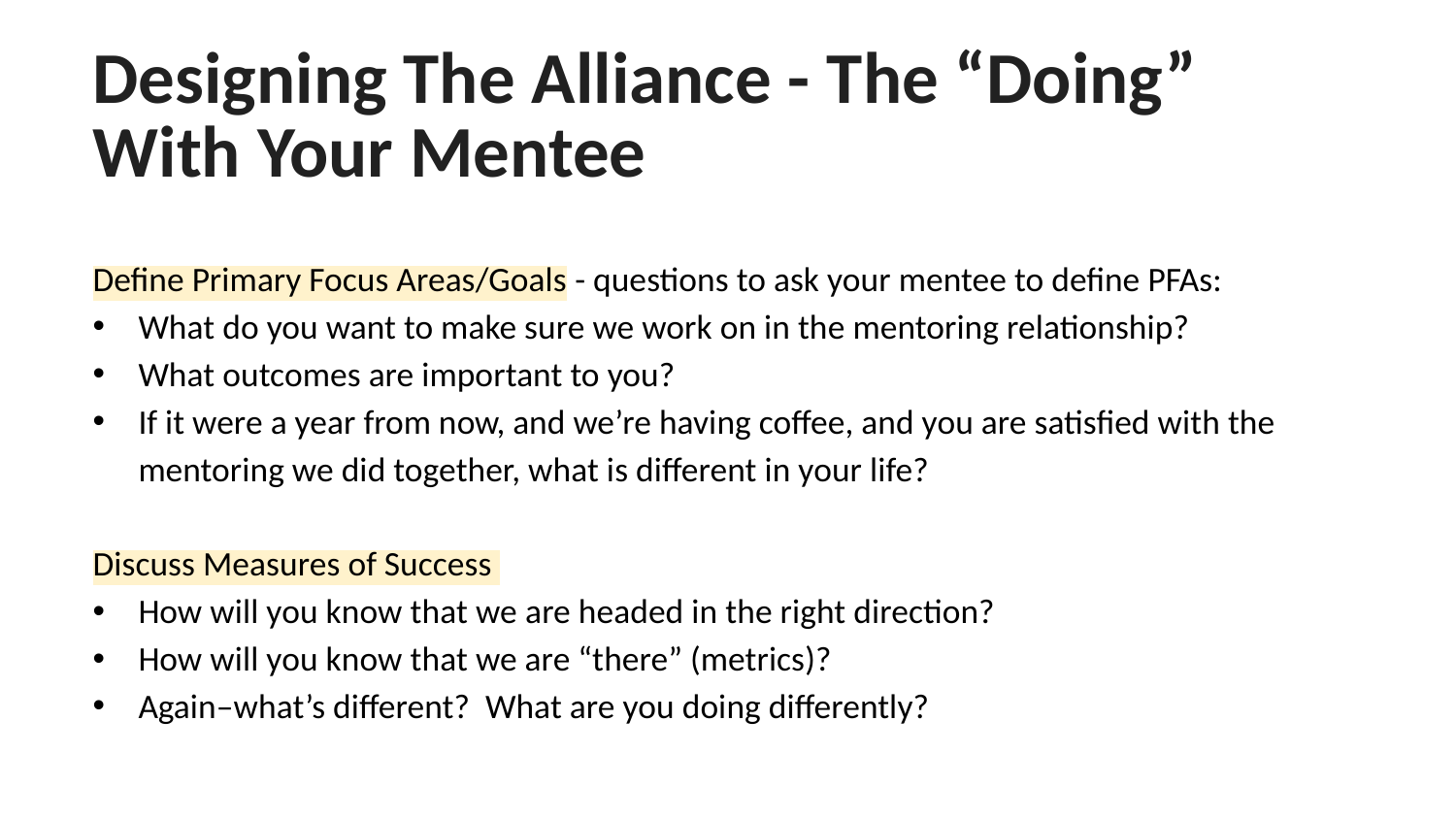

# Designing The Alliance - The “Doing” With Your Mentee
Define Primary Focus Areas/Goals - questions to ask your mentee to define PFAs:
What do you want to make sure we work on in the mentoring relationship?
What outcomes are important to you?
If it were a year from now, and we’re having coffee, and you are satisfied with the mentoring we did together, what is different in your life?
Discuss Measures of Success
How will you know that we are headed in the right direction?
How will you know that we are “there” (metrics)?
Again–what’s different? What are you doing differently?

## Slide 15
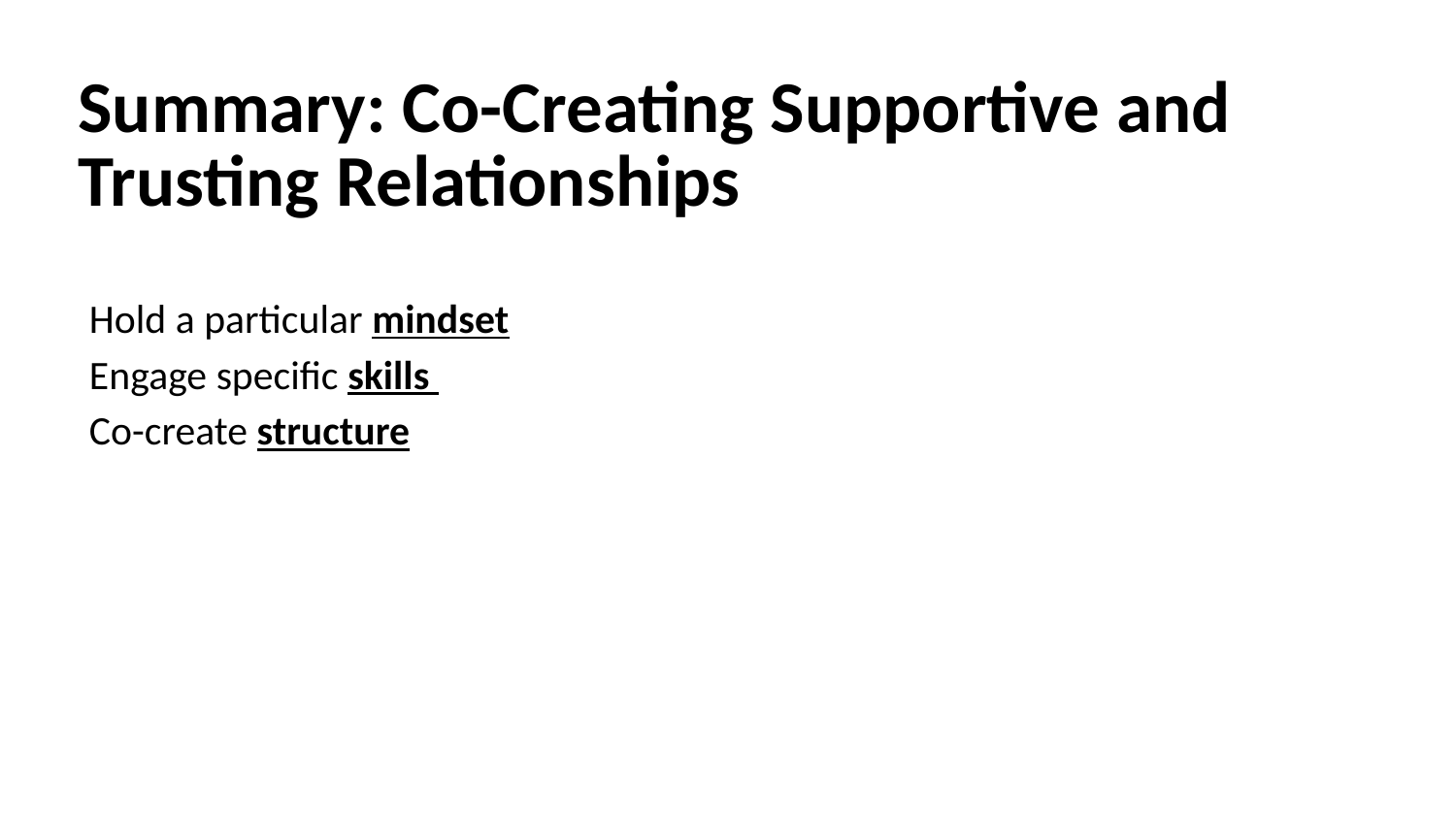

# Summary: Co-Creating Supportive and Trusting Relationships
Hold a particular mindset
Engage specific skills
Co-create structure

## Slide 16
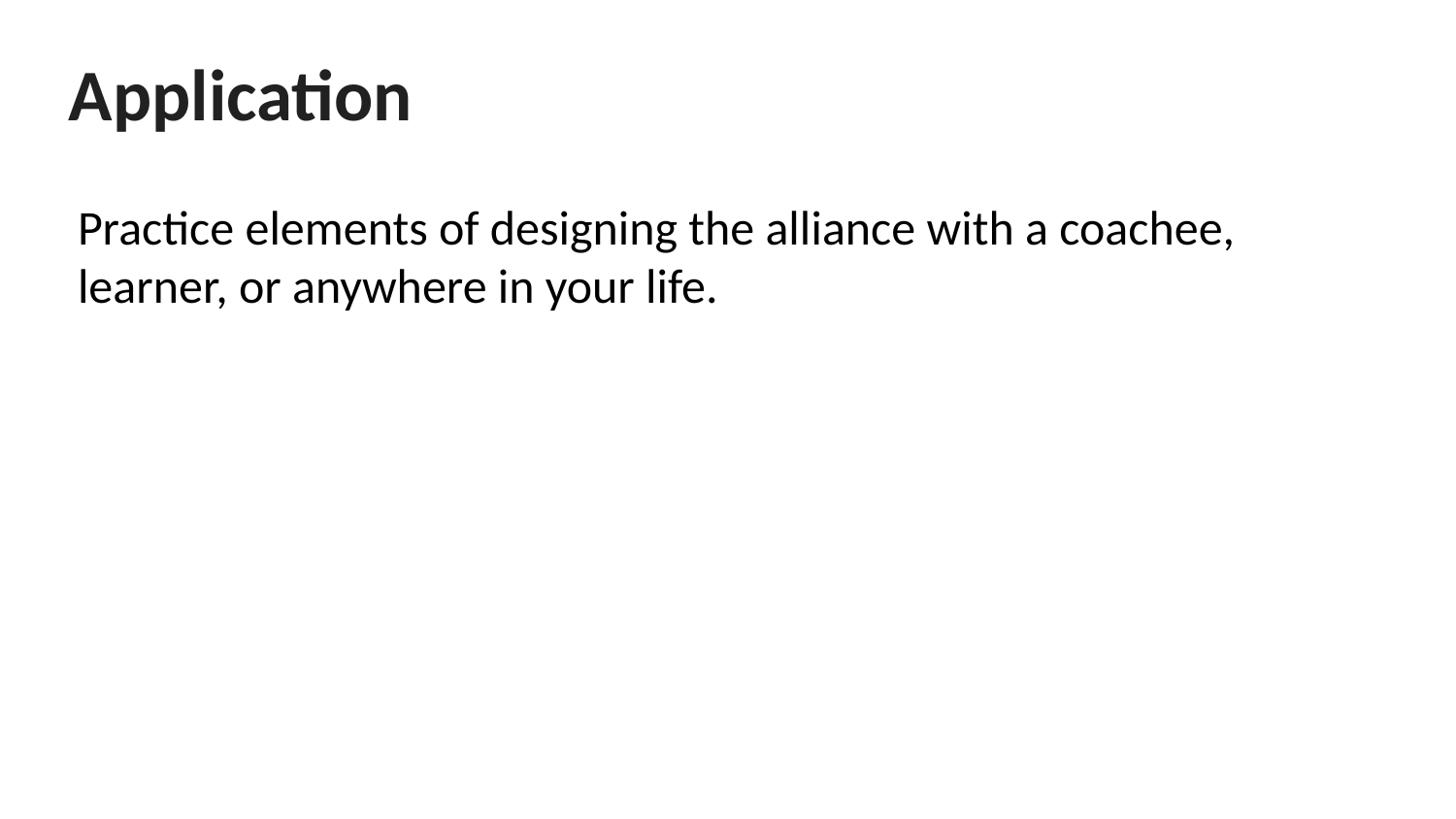

# Application
Practice elements of designing the alliance with a coachee, learner, or anywhere in your life.
