# Supplementary material for: A Longitudinal, Practical Curriculum for Faculty Development as New Coaches in Graduate Medical Education
Source: J Educ Teach Emerg Med. 2025 Jul 31;10(3):C1–C92. doi: 10.21980/J88M08 (PMC12320991; doi:10.21980/J88M08)
Supplement: Supplementary file 4 [file 10-3-C1-SuppG2.pptx]

## Slide 1
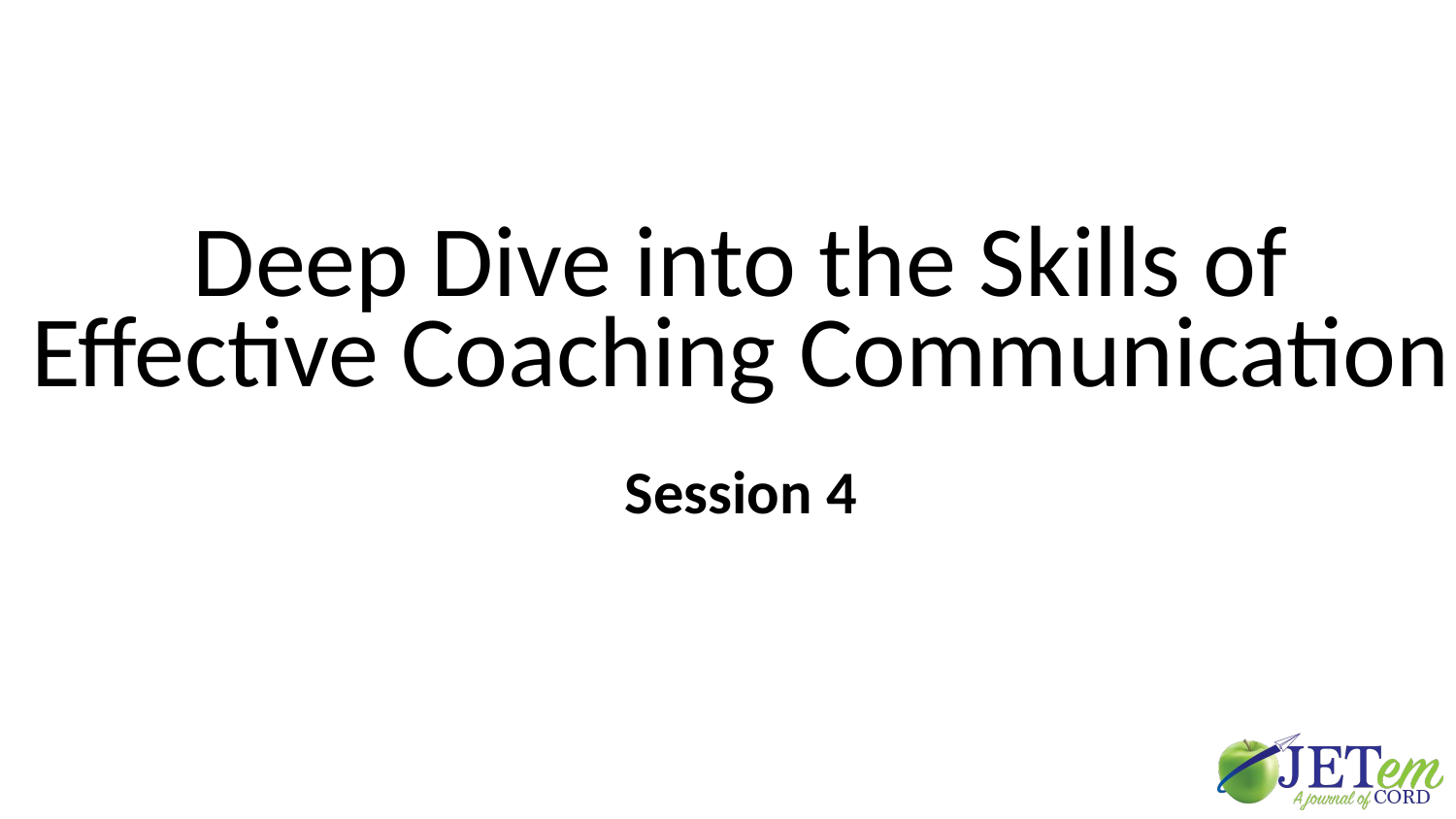

Deep Dive into the Skills of Effective Coaching Communication
Session 4

## Slide 2
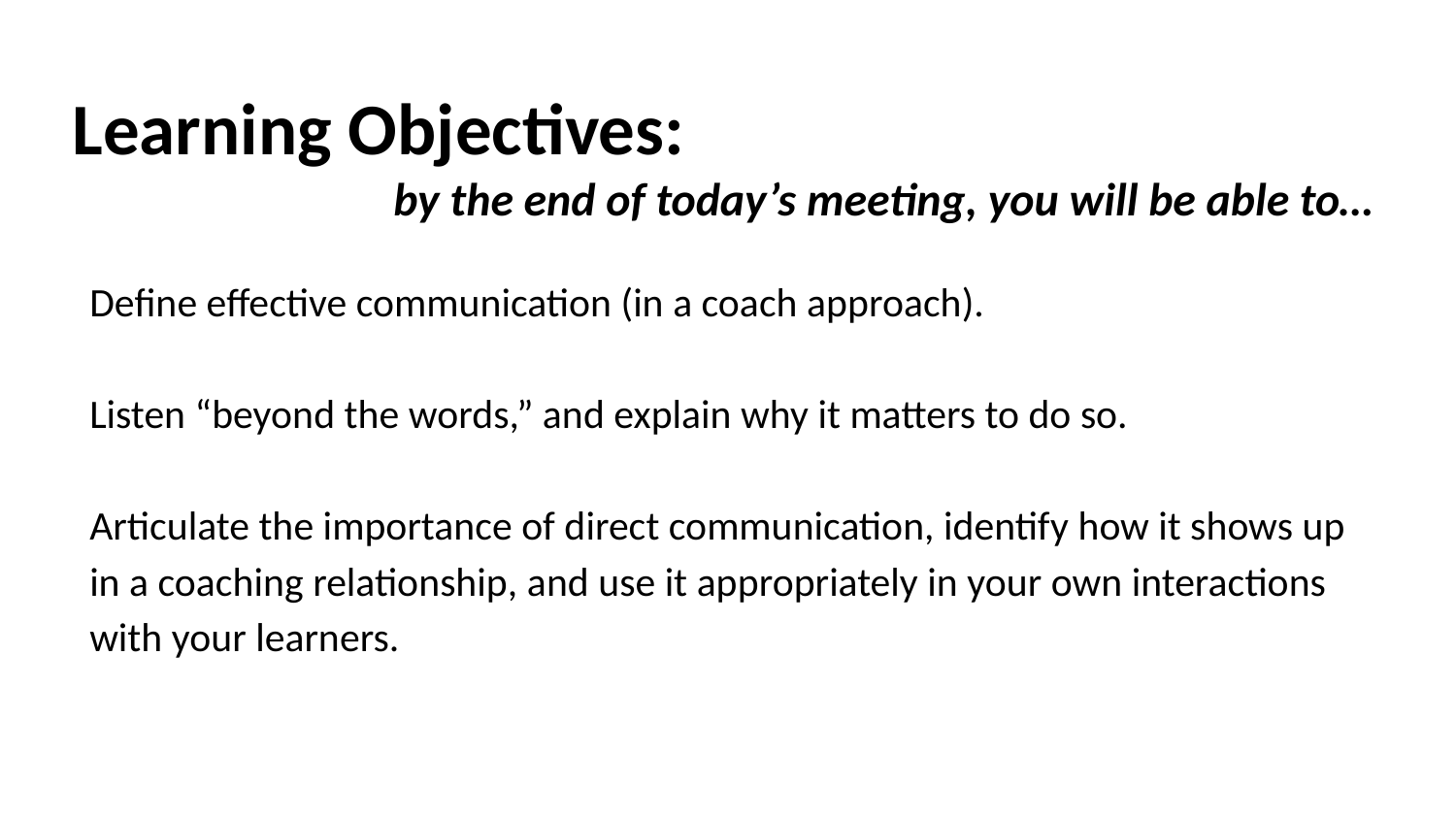

# Learning Objectives:
by the end of today’s meeting, you will be able to…
Define effective communication (in a coach approach).
Listen “beyond the words,” and explain why it matters to do so.
Articulate the importance of direct communication, identify how it shows up in a coaching relationship, and use it appropriately in your own interactions with your learners.

## Slide 3
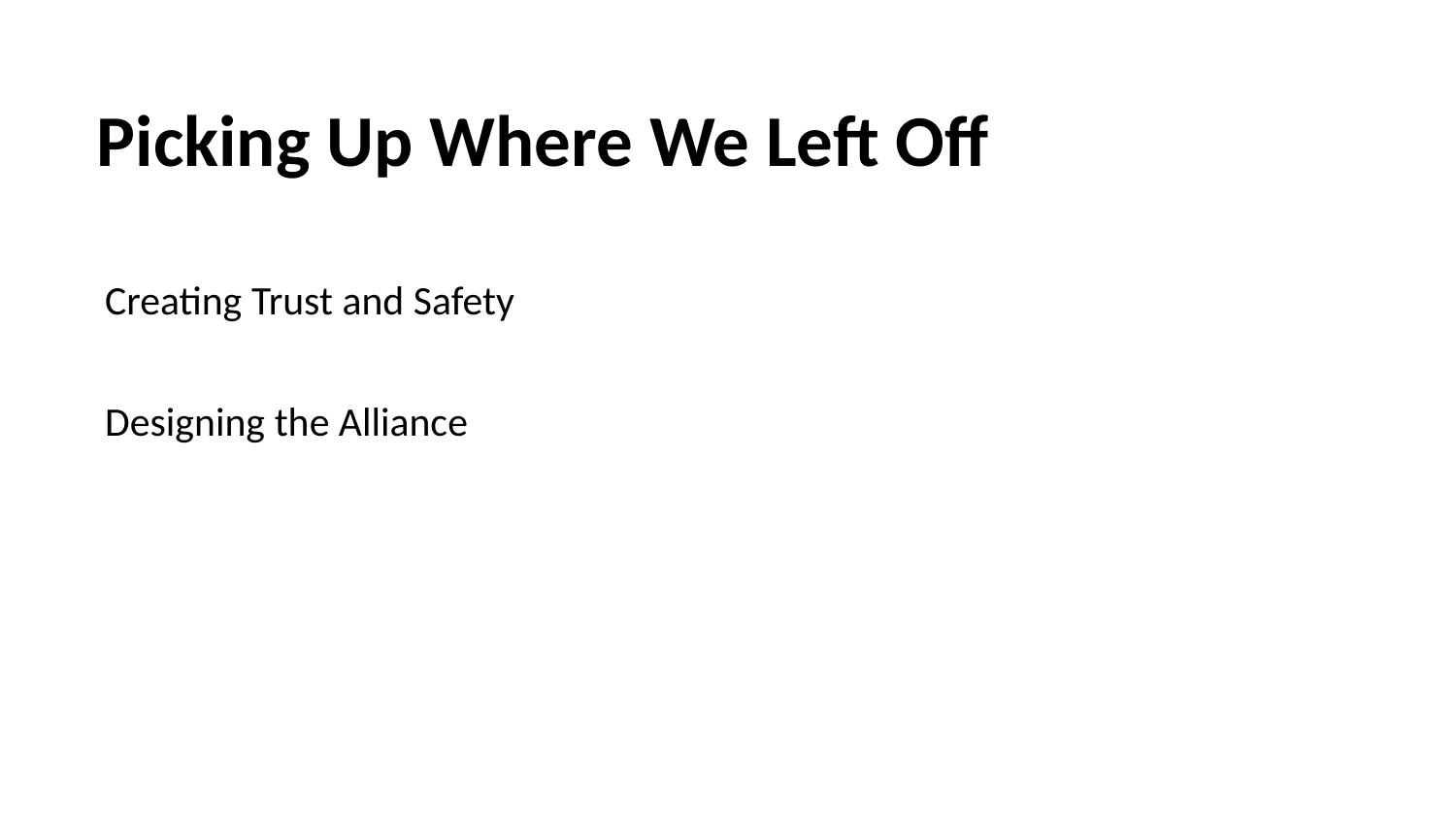

# Picking Up Where We Left Off
Creating Trust and Safety
Designing the Alliance

## Slide 4
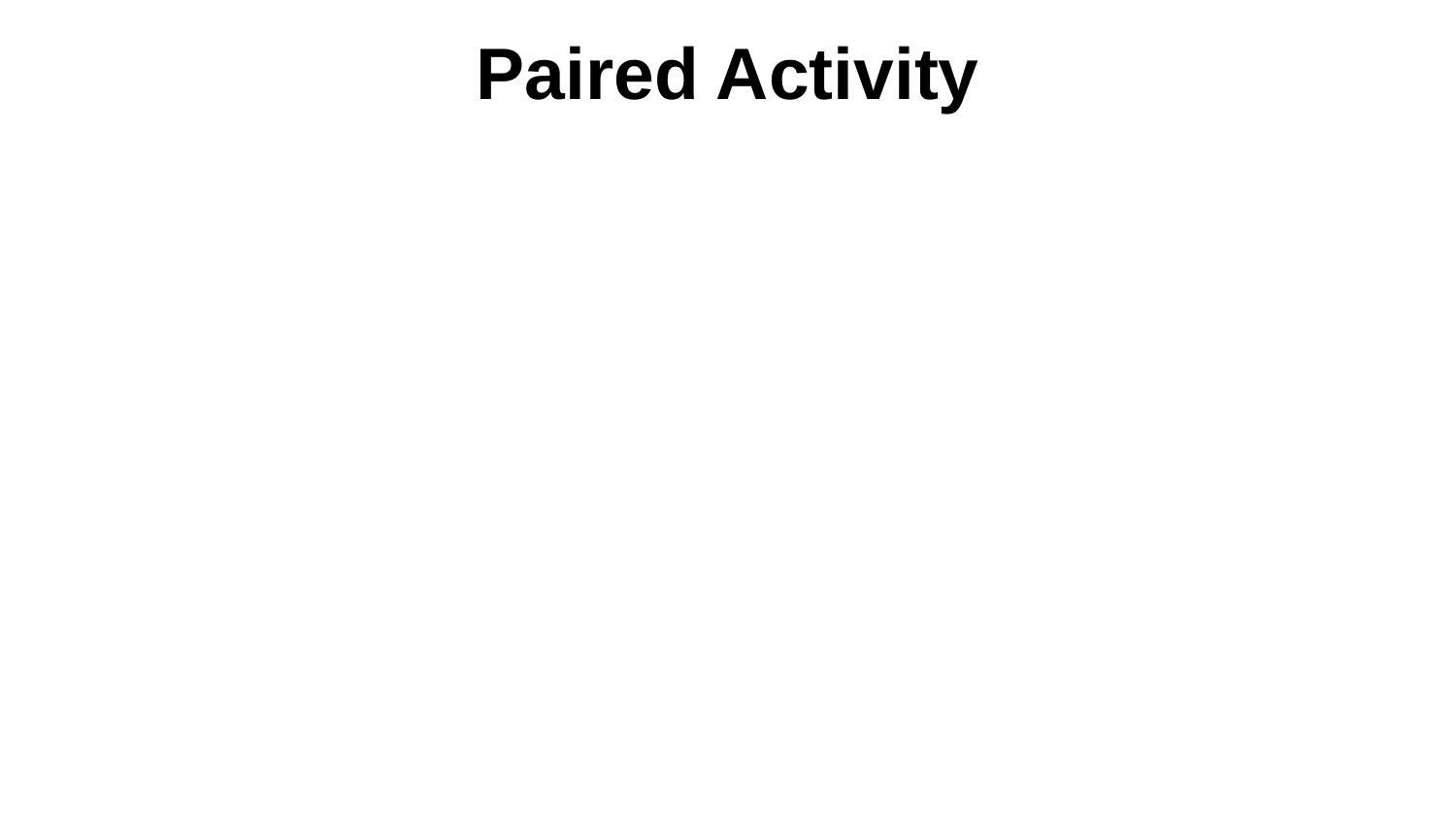

# Paired Activity

## Slide 5
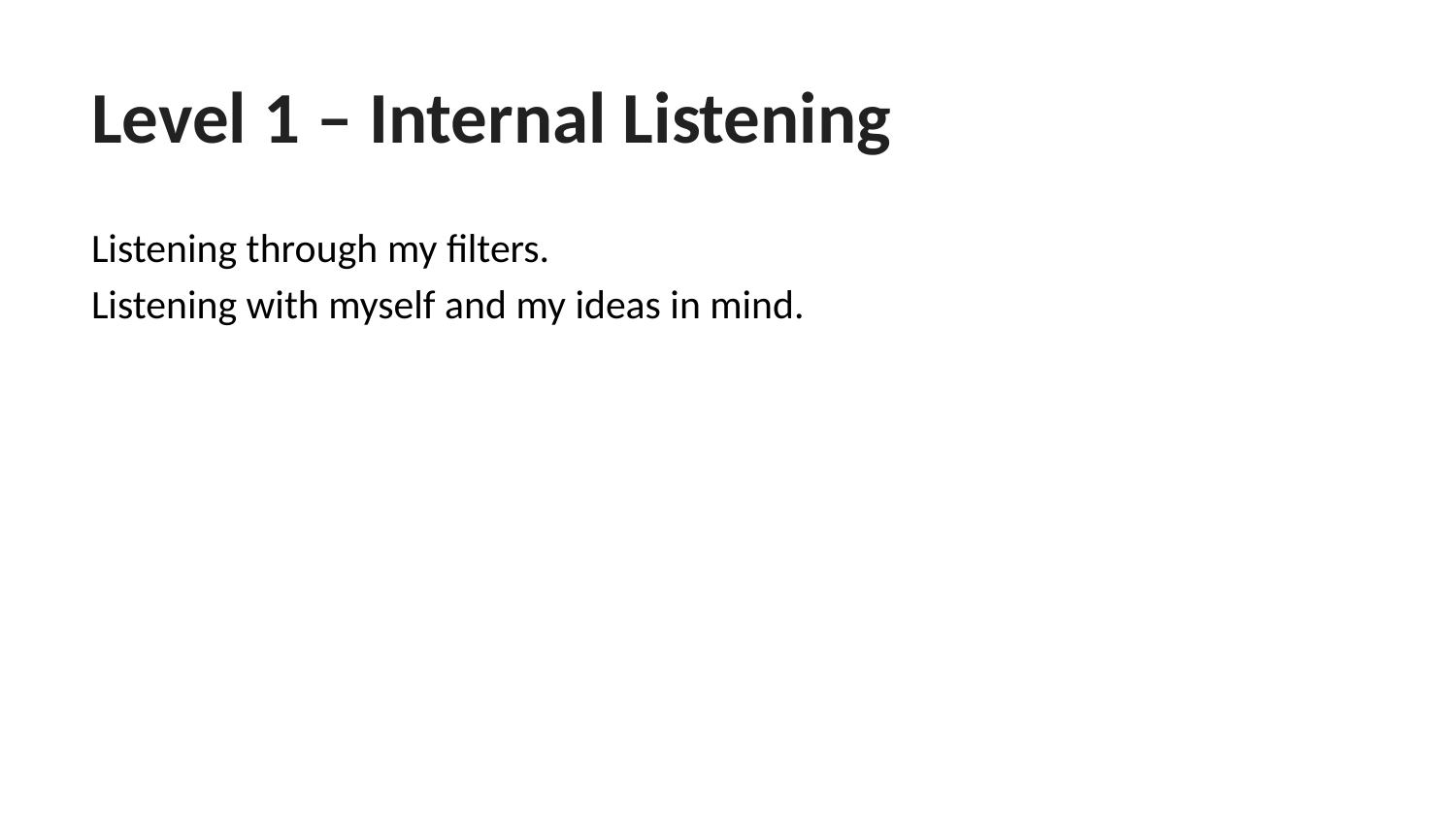

# Level 1 – Internal Listening
Listening through my filters.
Listening with myself and my ideas in mind.

## Slide 6
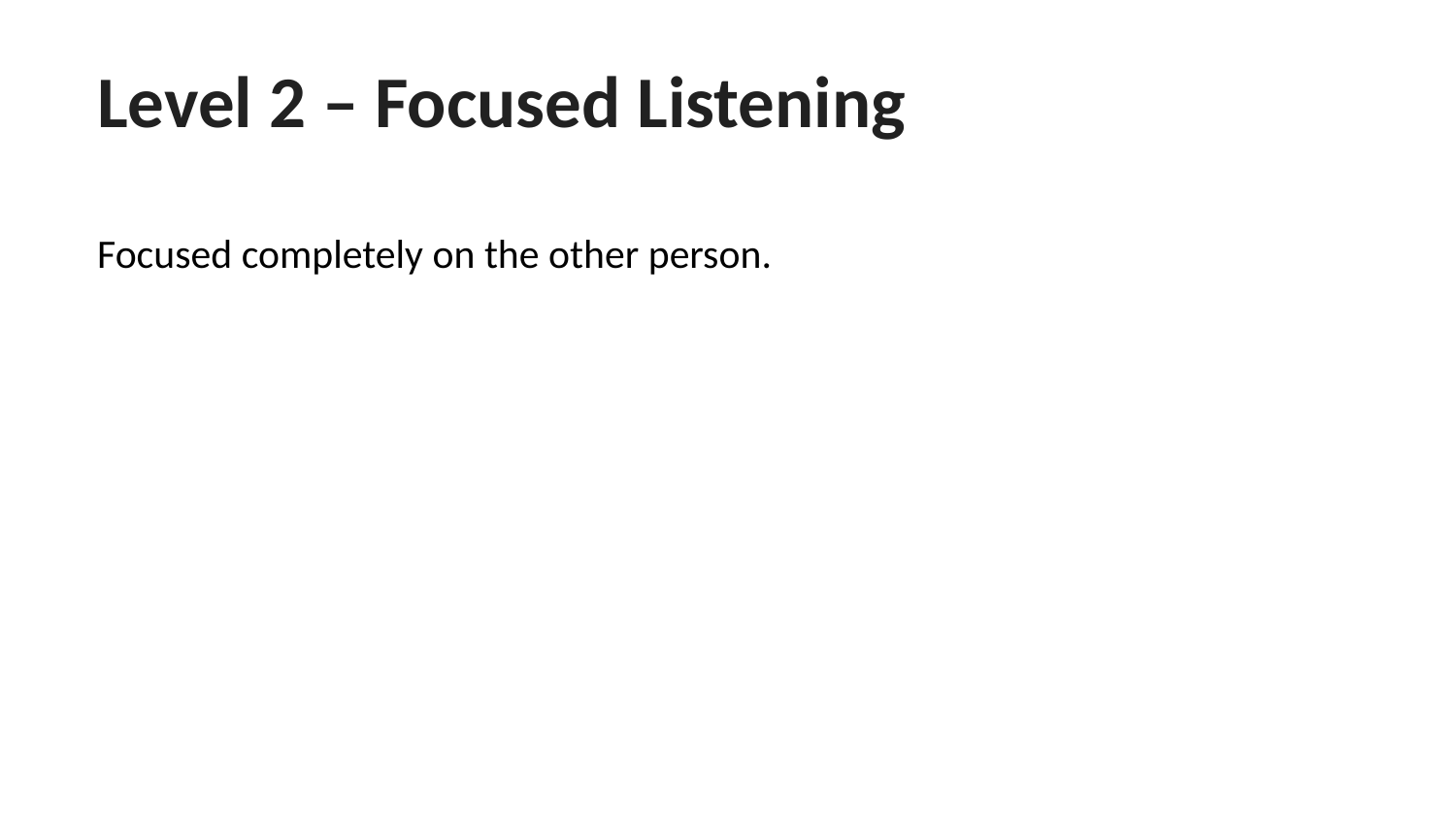

# Level 2 – Focused Listening
Focused completely on the other person.

## Slide 7
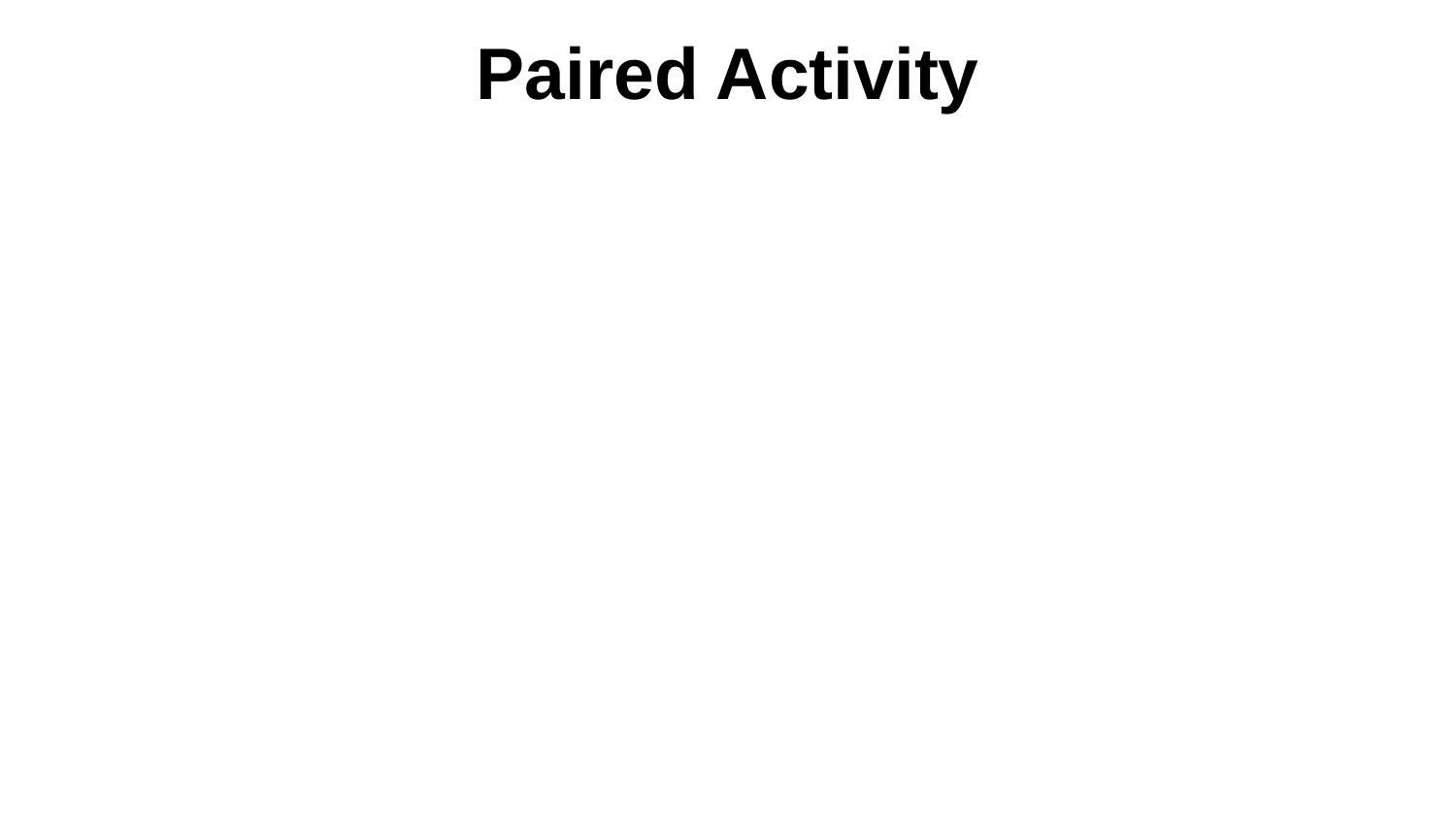

# Paired Activity

## Slide 8
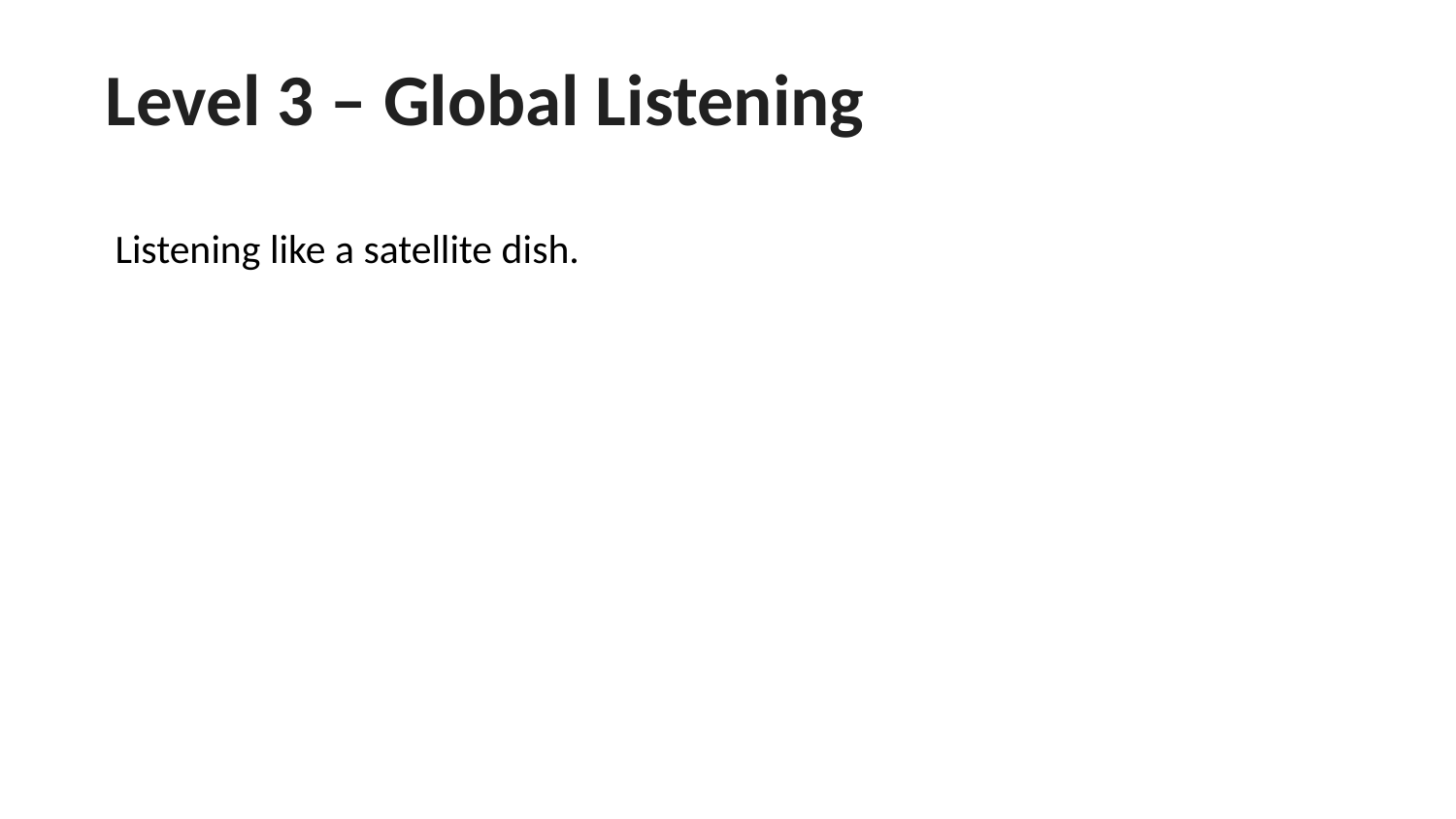

# Level 3 – Global Listening
Listening like a satellite dish.

## Slide 9
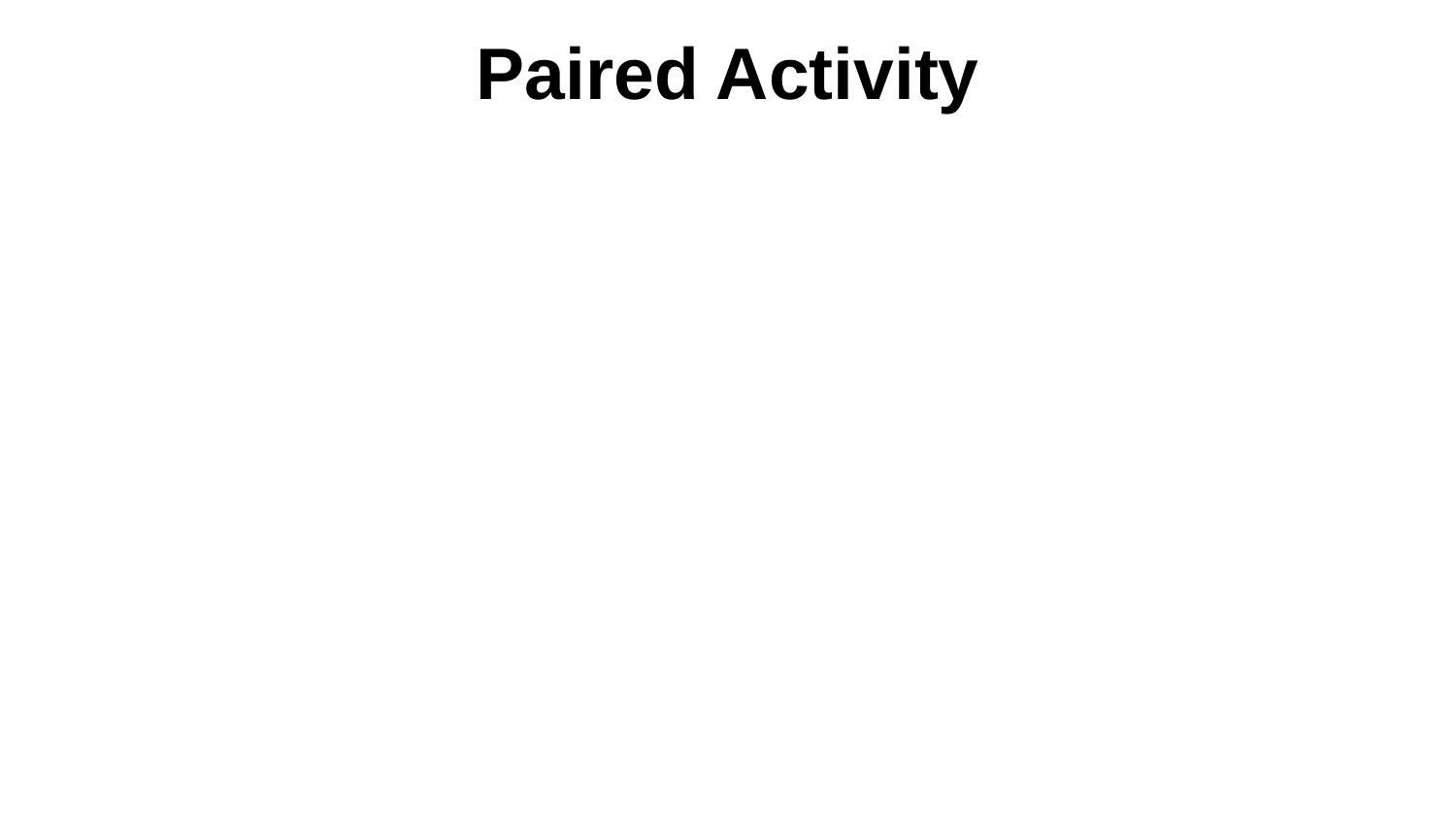

# Paired Activity

## Slide 10
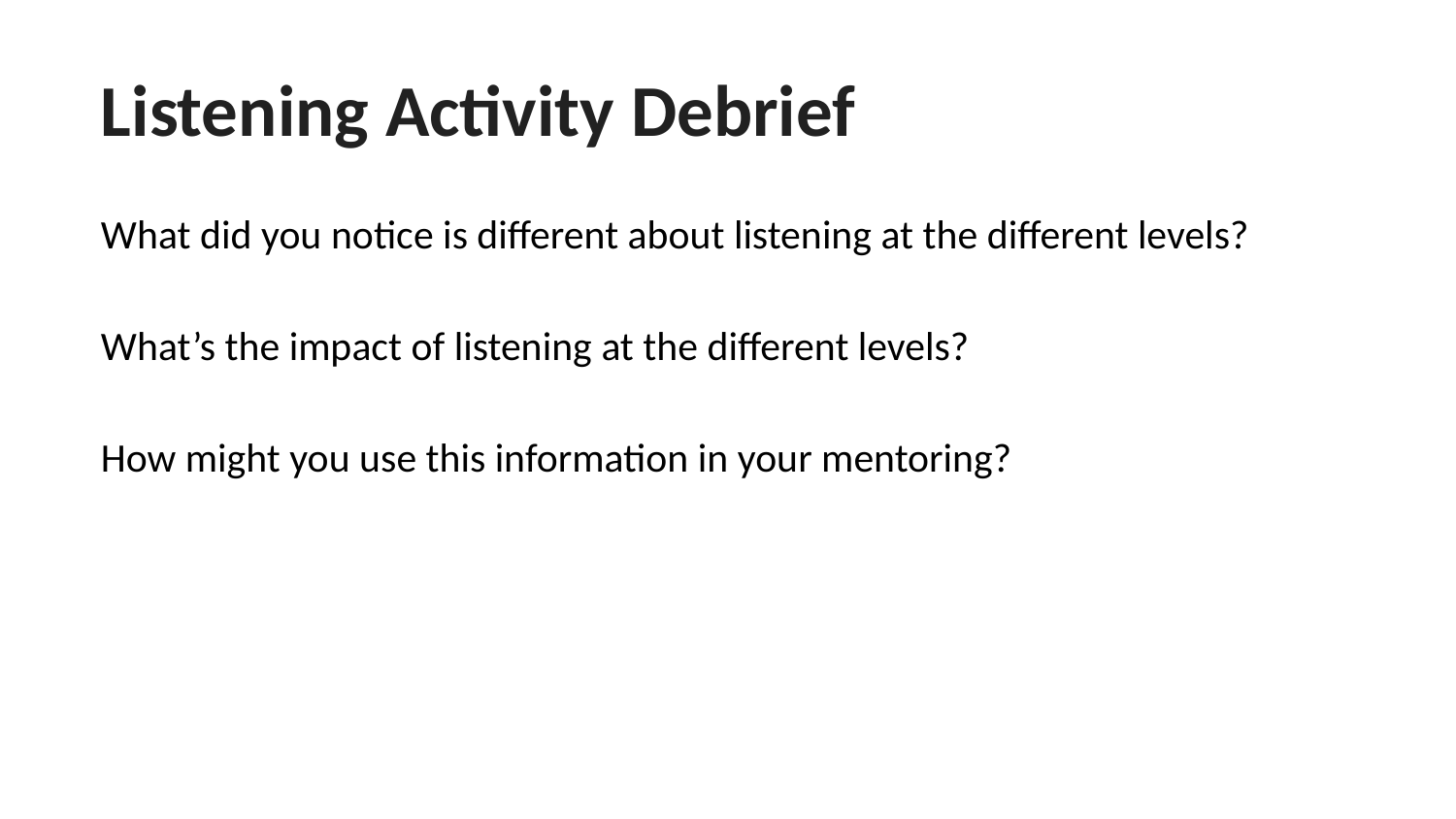

# Listening Activity Debrief
What did you notice is different about listening at the different levels?
What’s the impact of listening at the different levels?
How might you use this information in your mentoring?

## Slide 11
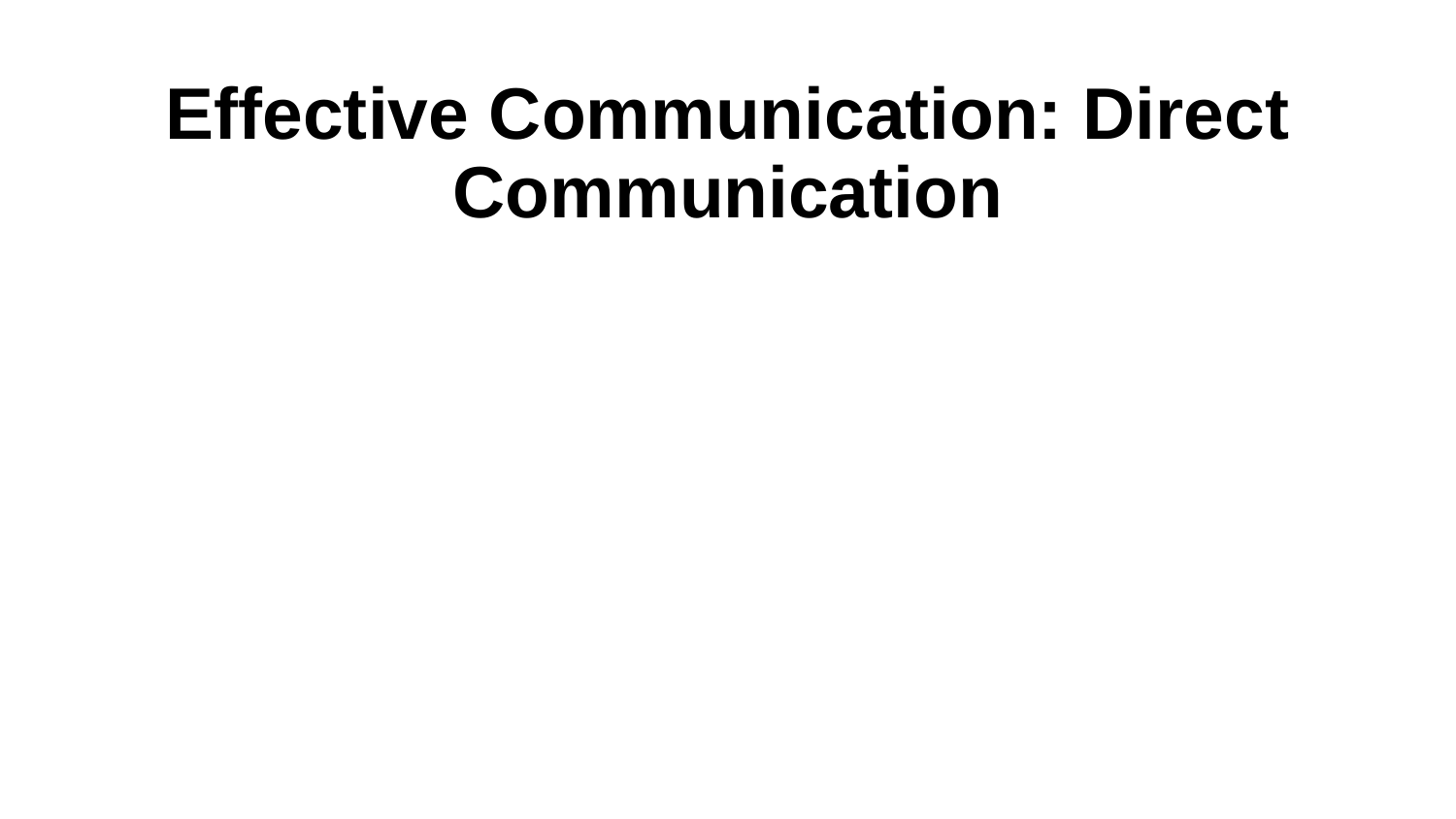

# Effective Communication: Direct Communication

## Slide 12
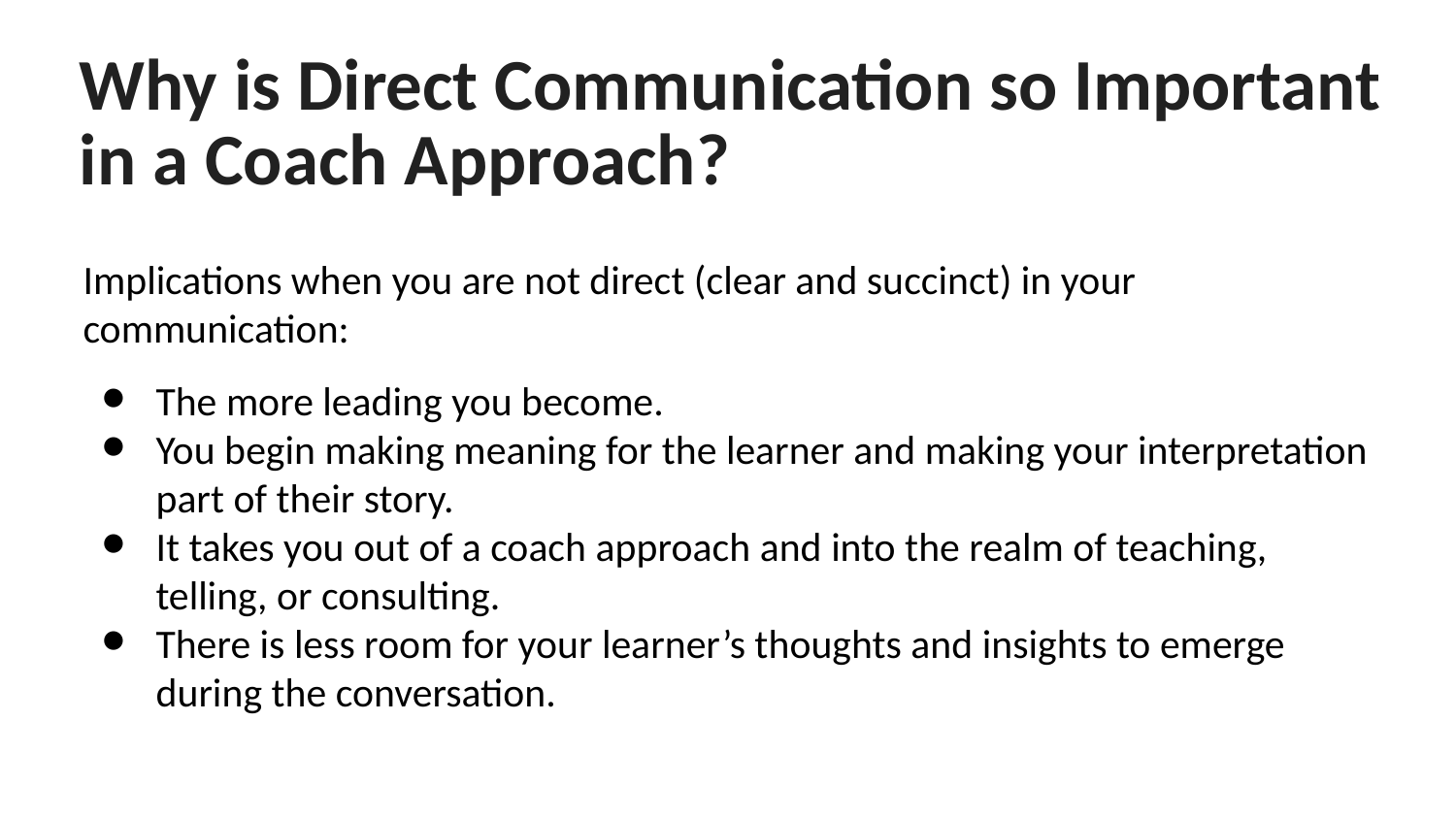

# Why is Direct Communication so Important in a Coach Approach?
Implications when you are not direct (clear and succinct) in your communication:
The more leading you become.
You begin making meaning for the learner and making your interpretation part of their story.
It takes you out of a coach approach and into the realm of teaching, telling, or consulting.
There is less room for your learner’s thoughts and insights to emerge during the conversation.

## Slide 13
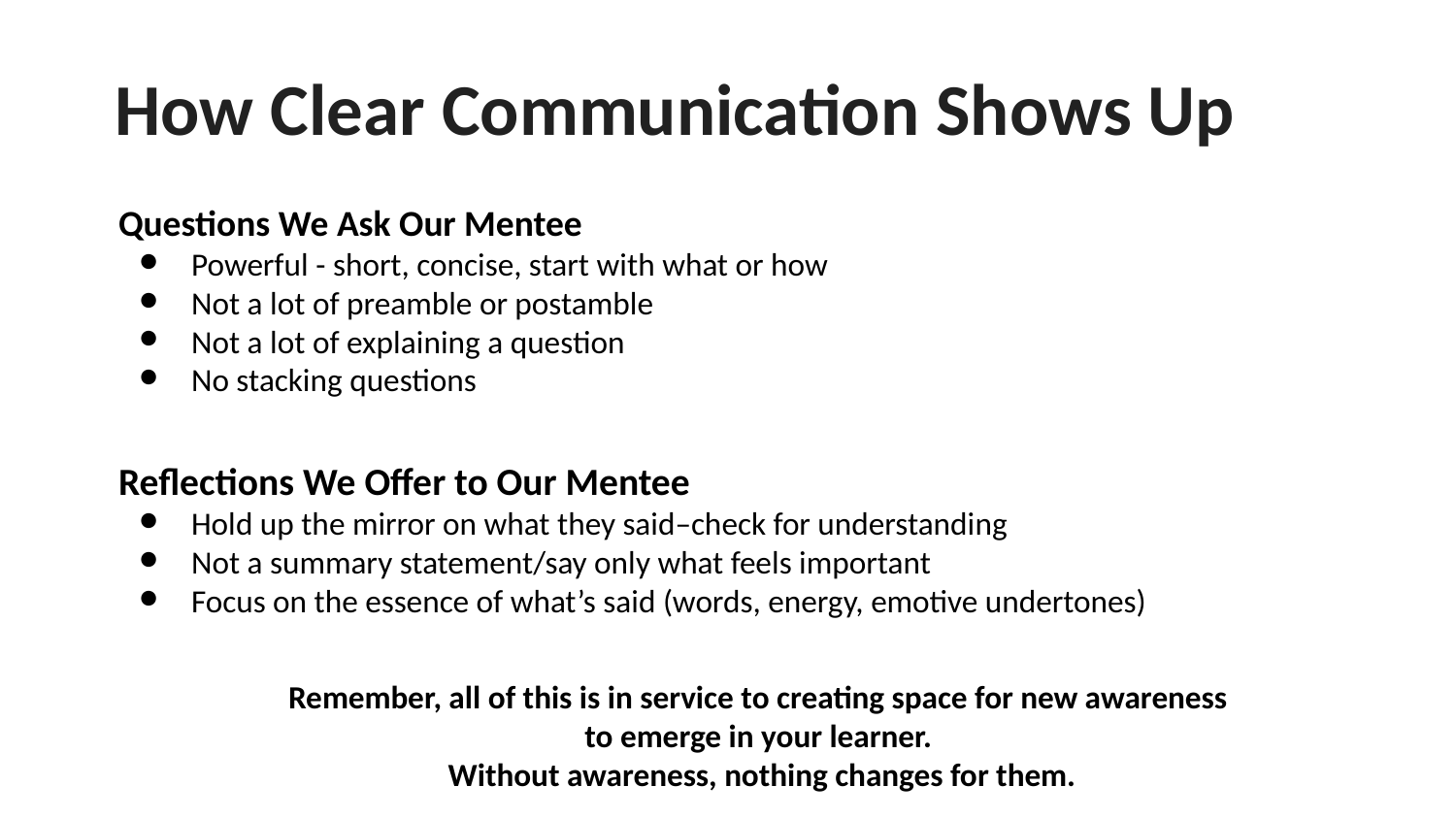

# How Clear Communication Shows Up
Questions We Ask Our Mentee
Powerful - short, concise, start with what or how
Not a lot of preamble or postamble
Not a lot of explaining a question
No stacking questions
Reflections We Offer to Our Mentee
Hold up the mirror on what they said–check for understanding
Not a summary statement/say only what feels important
Focus on the essence of what’s said (words, energy, emotive undertones)
Remember, all of this is in service to creating space for new awareness
to emerge in your learner.
Without awareness, nothing changes for them.

## Slide 14
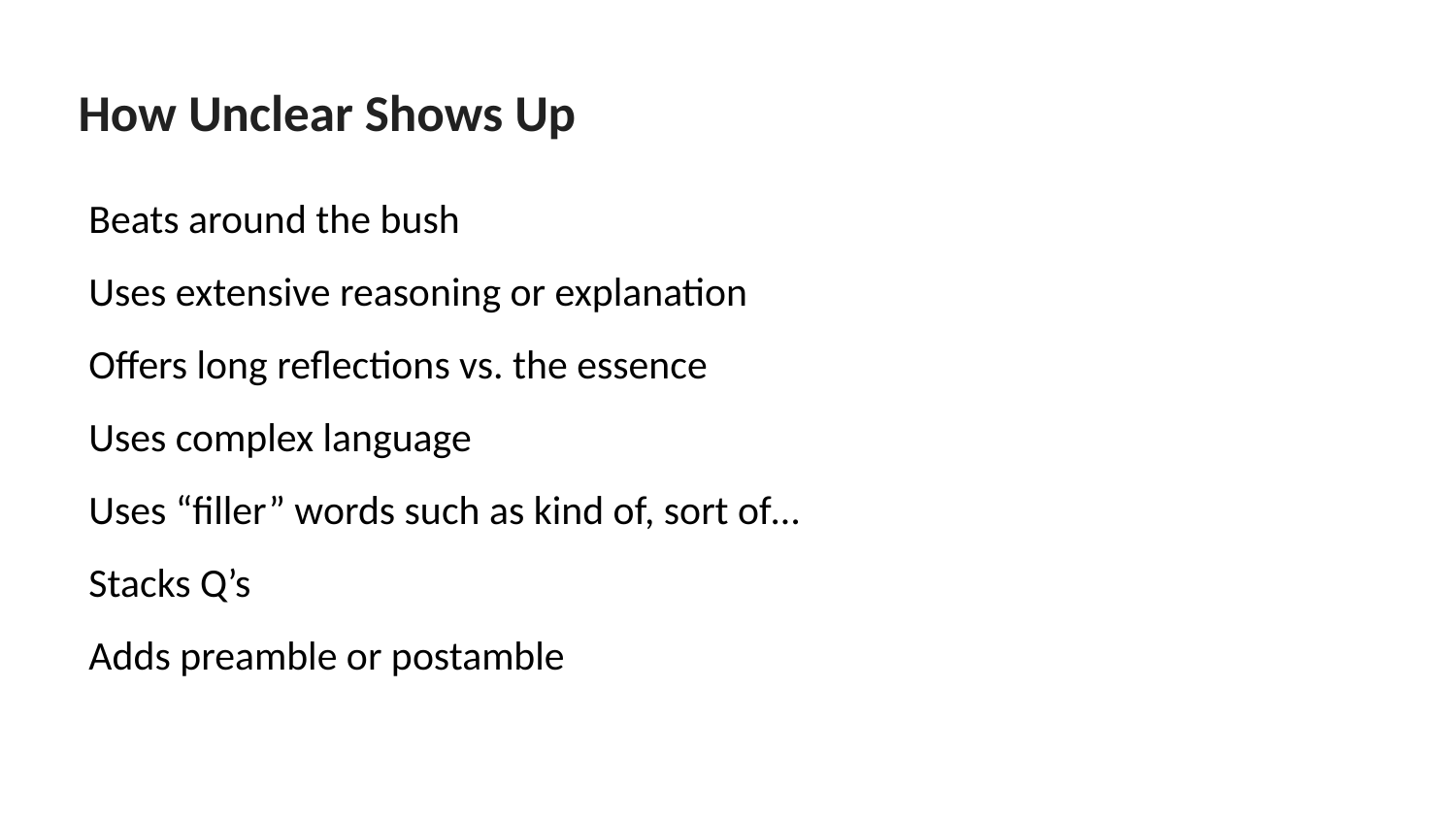

# How Unclear Shows Up
Beats around the bush
Uses extensive reasoning or explanation
Offers long reflections vs. the essence
Uses complex language
Uses “filler” words such as kind of, sort of…
Stacks Q’s
Adds preamble or postamble

## Slide 15
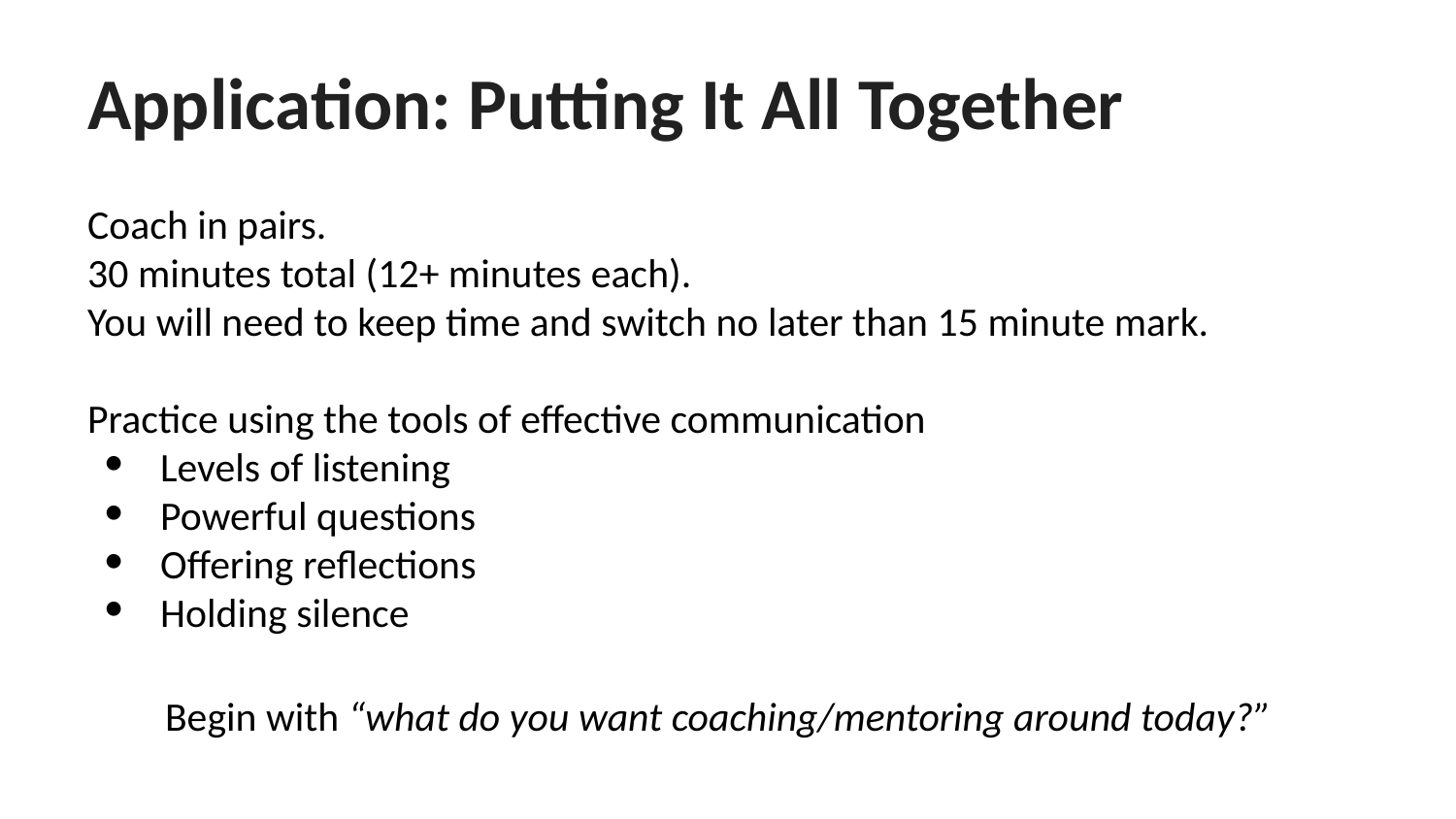

# Application: Putting It All Together
Coach in pairs.
30 minutes total (12+ minutes each).
You will need to keep time and switch no later than 15 minute mark.
Practice using the tools of effective communication
Levels of listening
Powerful questions
Offering reflections
Holding silence
Begin with “what do you want coaching/mentoring around today?”

## Slide 16
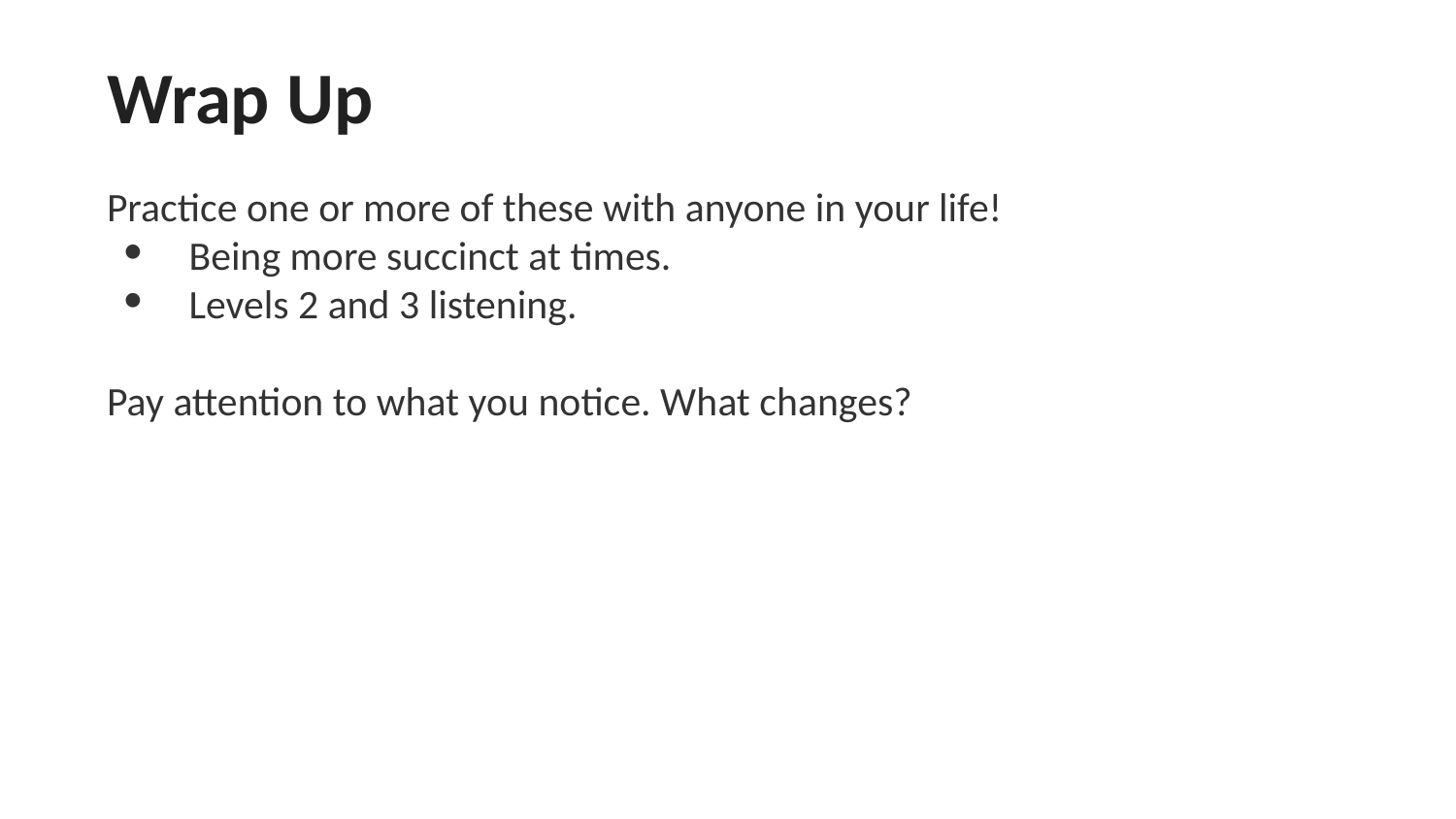

# Wrap Up
Practice one or more of these with anyone in your life!
 Being more succinct at times.
 Levels 2 and 3 listening.
Pay attention to what you notice. What changes?
