# Supplementary material for: A Longitudinal, Practical Curriculum for Faculty Development as New Coaches in Graduate Medical Education
Source: J Educ Teach Emerg Med. 2025 Jul 31;10(3):C1–C92. doi: 10.21980/J88M08 (PMC12320991; doi:10.21980/J88M08)
Supplement: Supplementary file 5 [file 10-3-C1-SuppH4.pptx]

## Slide 1
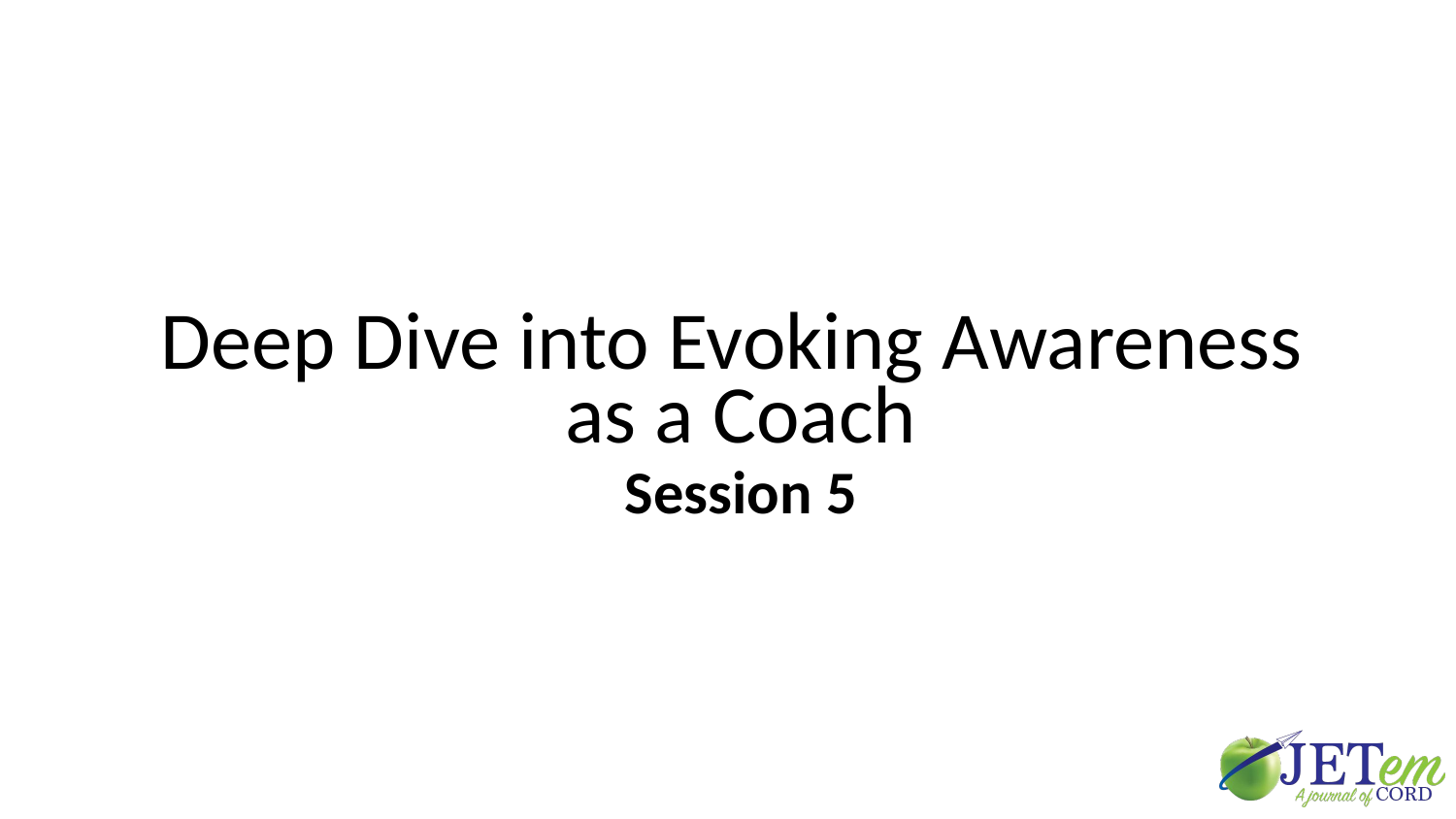

Deep Dive into Evoking Awareness
as a Coach
Session 5

## Slide 2
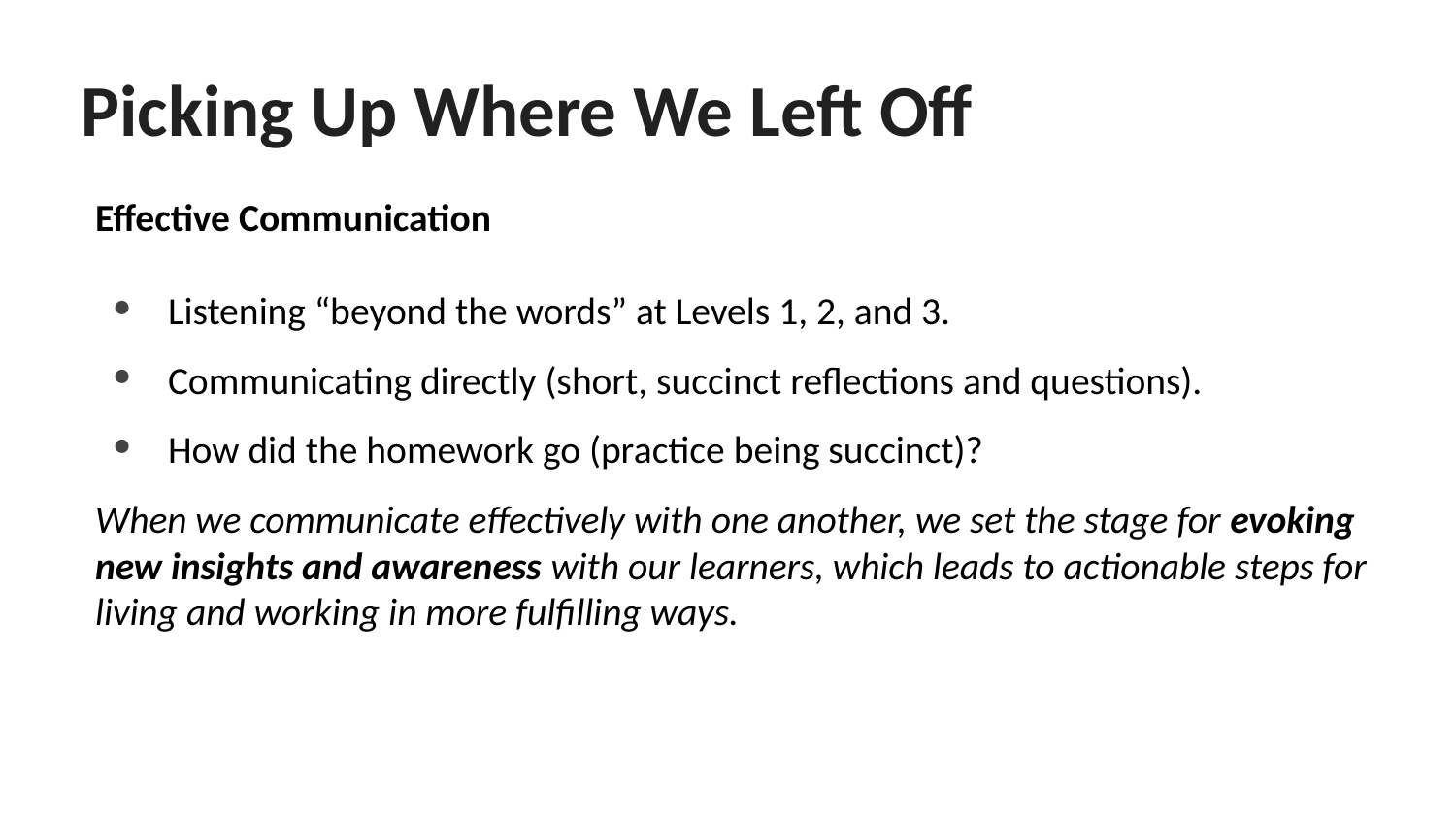

# Picking Up Where We Left Off
Effective Communication
Listening “beyond the words” at Levels 1, 2, and 3.
Communicating directly (short, succinct reflections and questions).
How did the homework go (practice being succinct)?
When we communicate effectively with one another, we set the stage for evoking new insights and awareness with our learners, which leads to actionable steps for living and working in more fulfilling ways.

## Slide 3
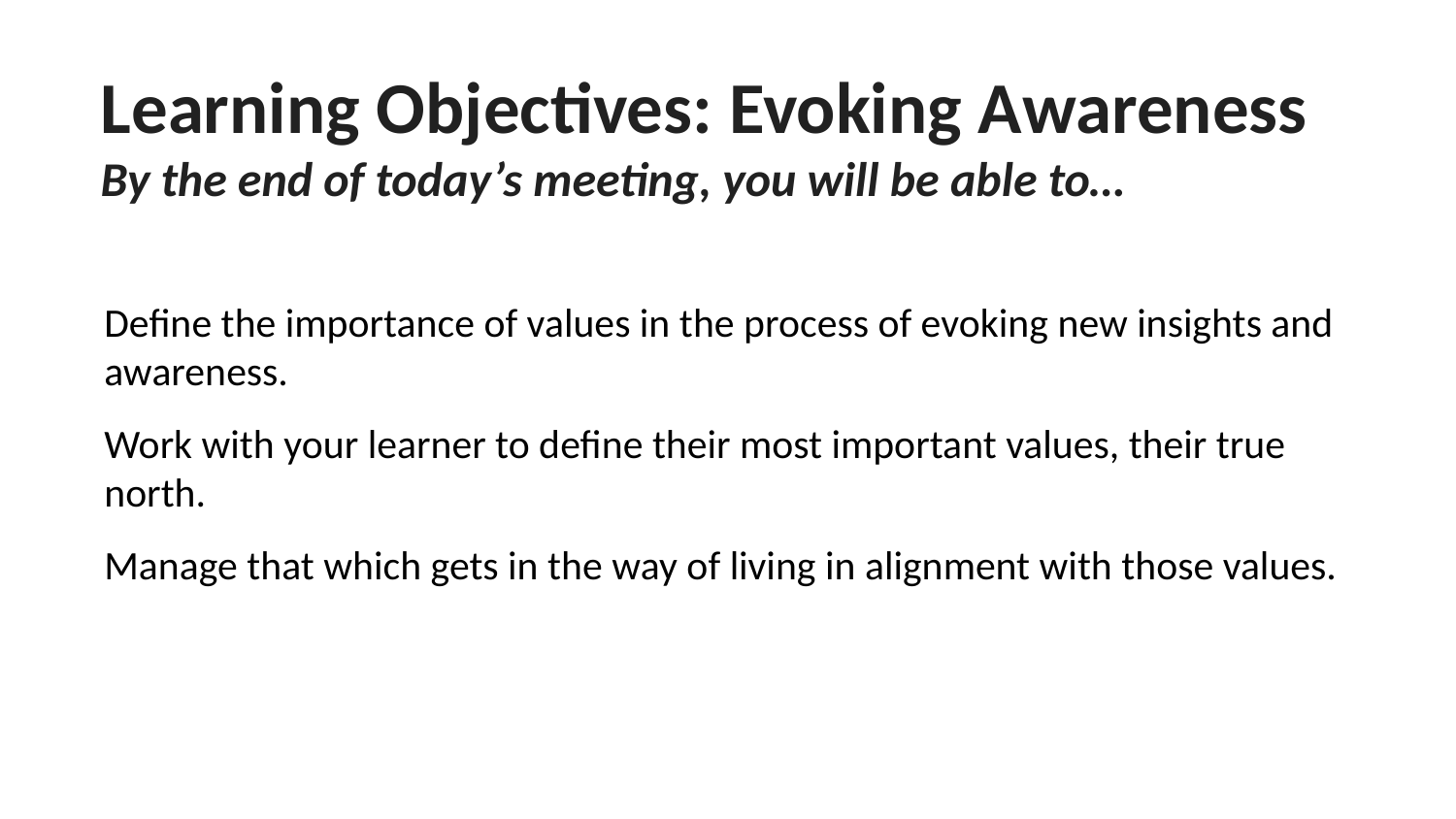

# Learning Objectives: Evoking Awareness
By the end of today’s meeting, you will be able to…
Define the importance of values in the process of evoking new insights and awareness.
Work with your learner to define their most important values, their true north.
Manage that which gets in the way of living in alignment with those values.

## Slide 4
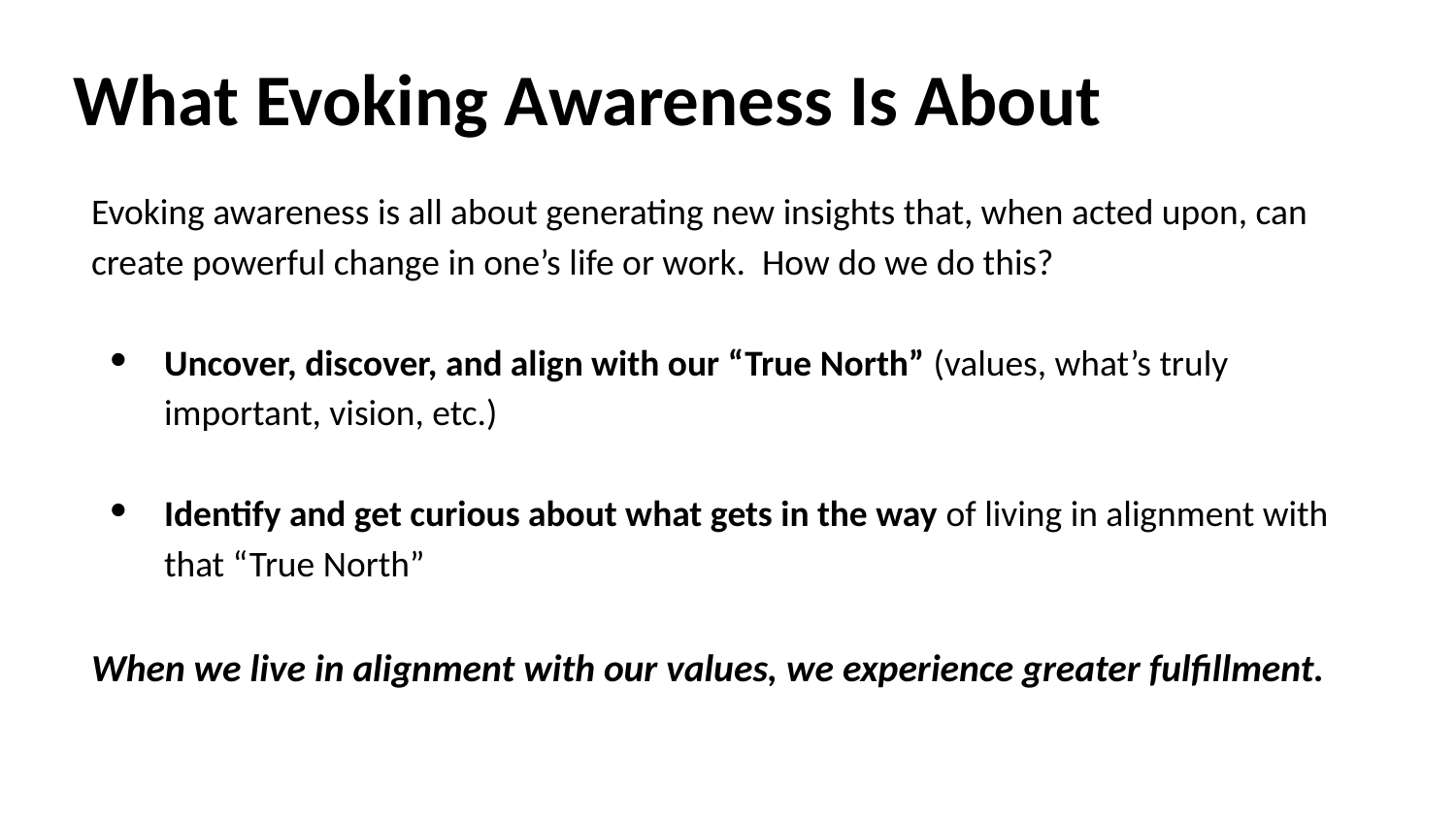

# What Evoking Awareness Is About
Evoking awareness is all about generating new insights that, when acted upon, can create powerful change in one’s life or work. How do we do this?
Uncover, discover, and align with our “True North” (values, what’s truly important, vision, etc.)
Identify and get curious about what gets in the way of living in alignment with that “True North”
When we live in alignment with our values, we experience greater fulfillment.

## Slide 5
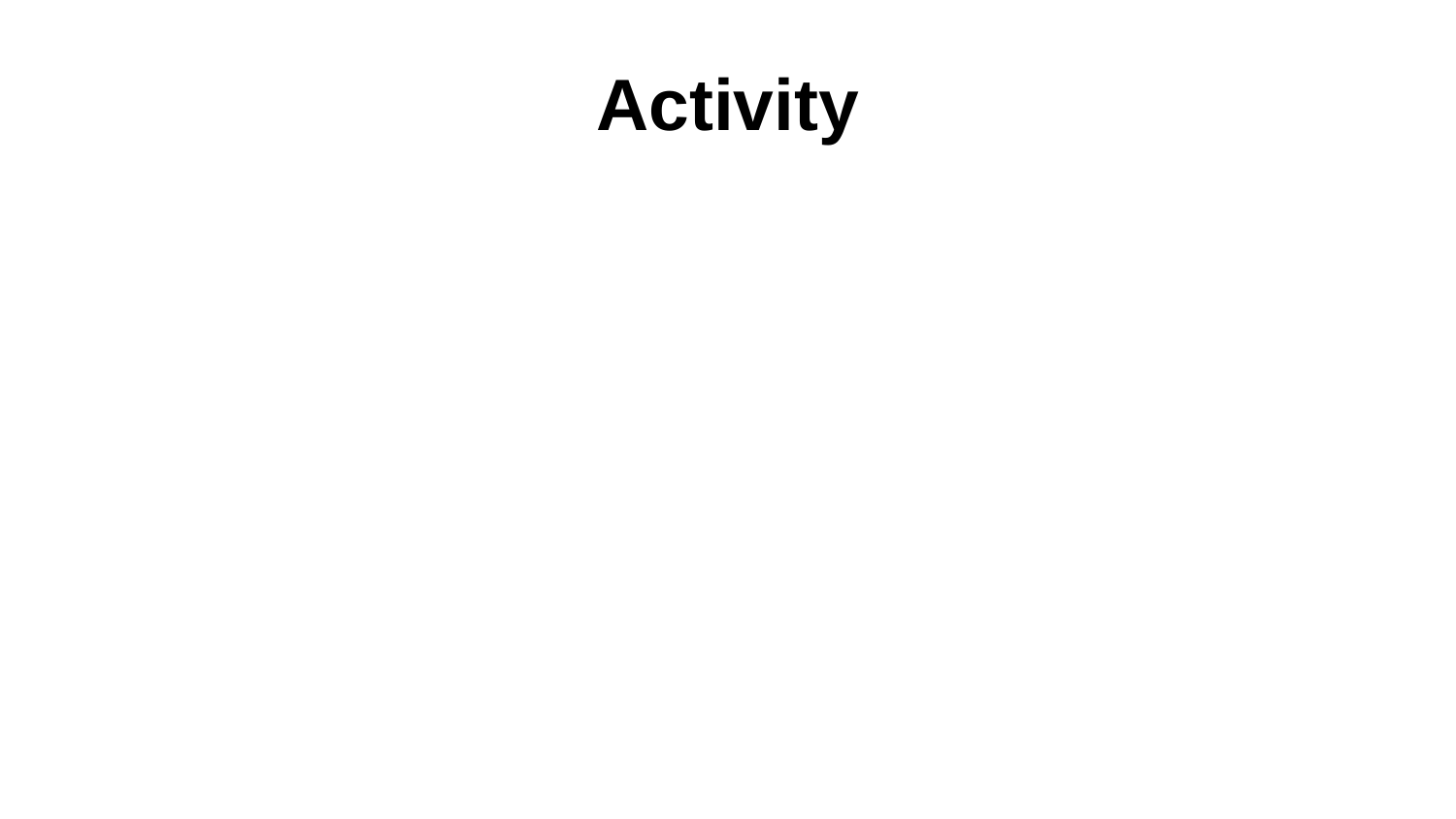

# Activity

## Slide 6
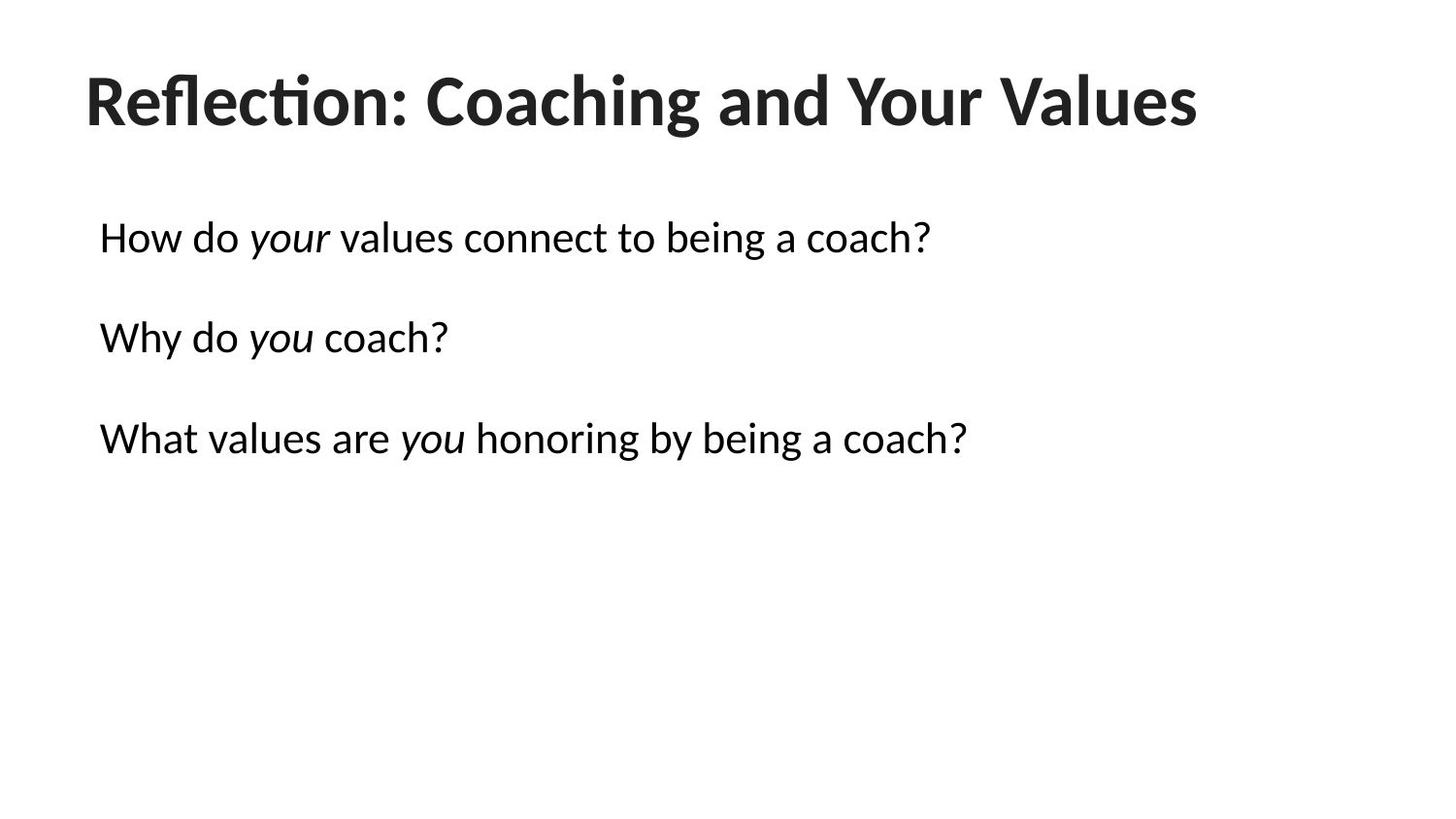

# Reflection: Coaching and Your Values
How do your values connect to being a coach?
Why do you coach?
What values are you honoring by being a coach?

## Slide 7
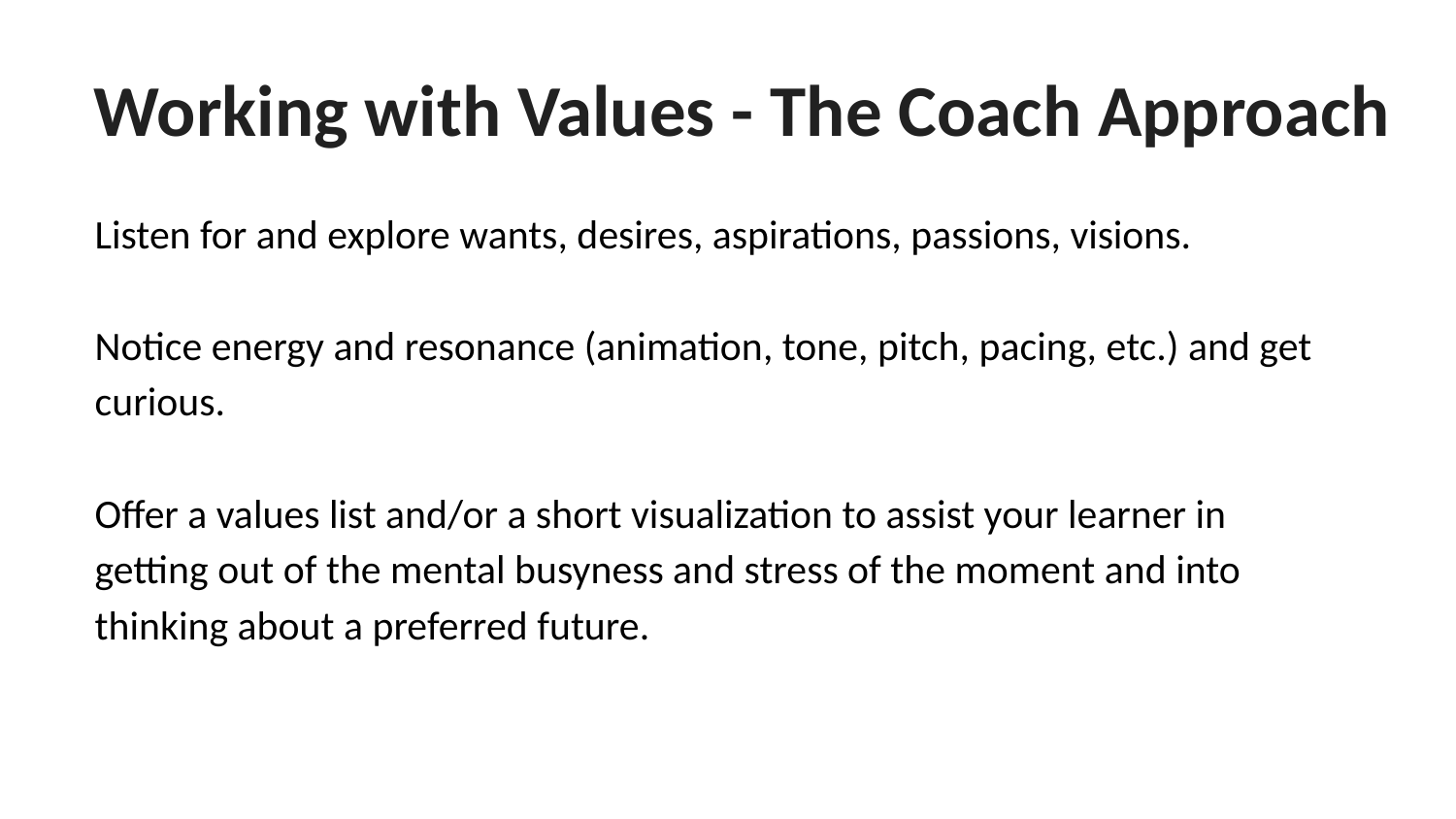

# Working with Values - The Coach Approach
Listen for and explore wants, desires, aspirations, passions, visions.
Notice energy and resonance (animation, tone, pitch, pacing, etc.) and get curious.
Offer a values list and/or a short visualization to assist your learner in getting out of the mental busyness and stress of the moment and into thinking about a preferred future.

## Slide 8
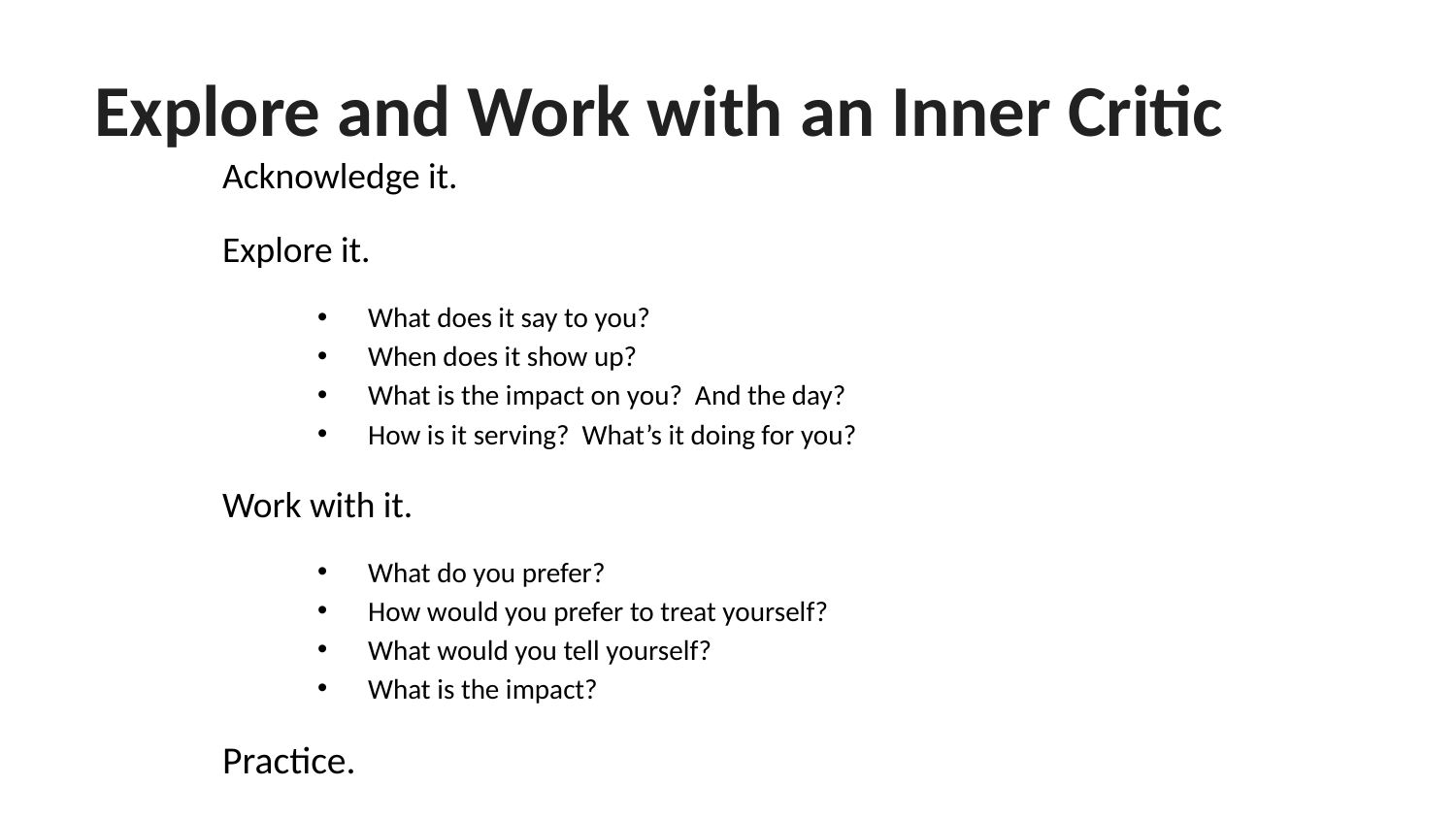

# Explore and Work with an Inner Critic
Acknowledge it.
Explore it.
What does it say to you?
When does it show up?
What is the impact on you? And the day?
How is it serving? What’s it doing for you?
Work with it.
What do you prefer?
How would you prefer to treat yourself?
What would you tell yourself?
What is the impact?
Practice.

## Slide 9
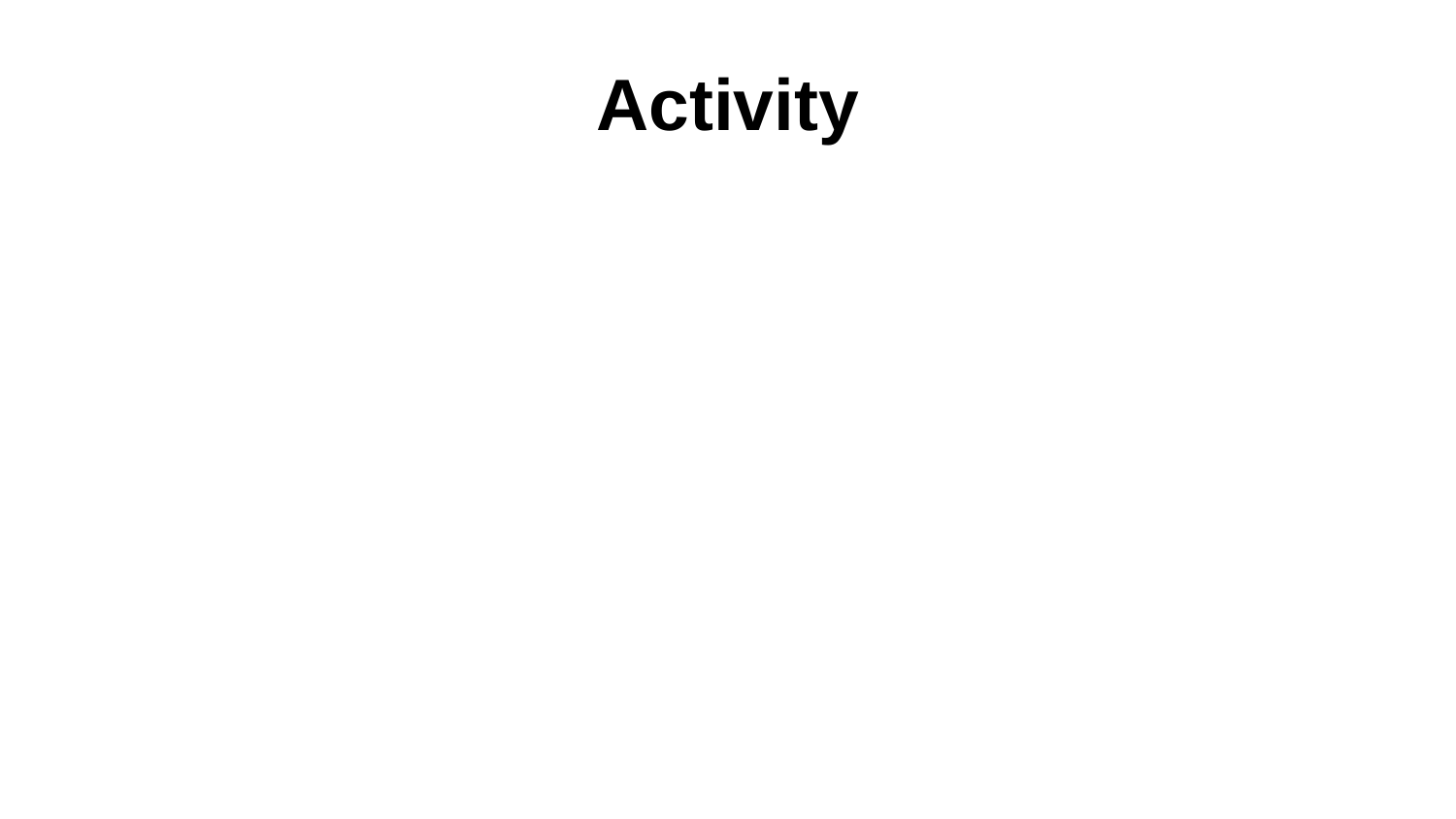

# Activity

## Slide 10
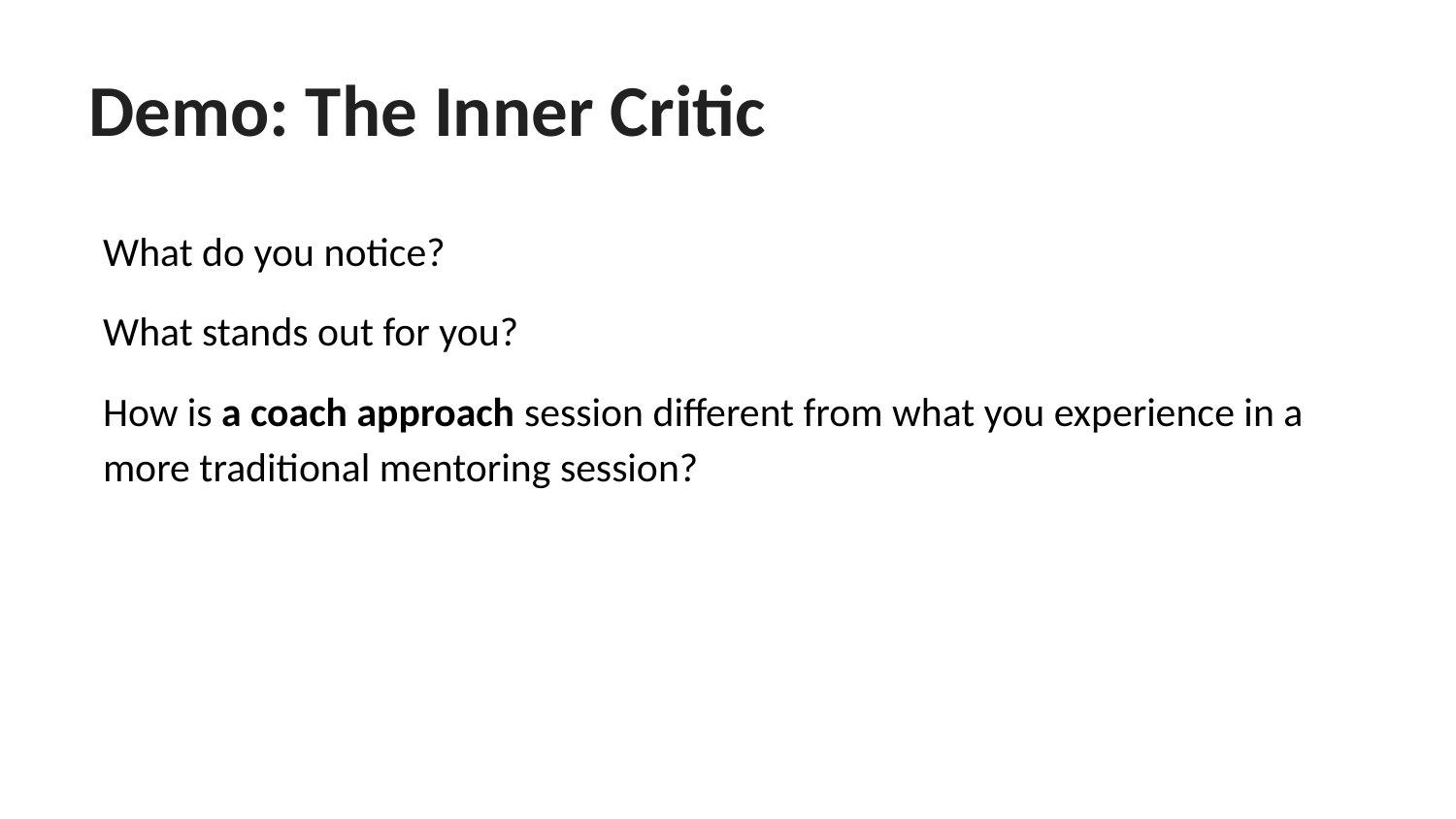

# Demo: The Inner Critic
What do you notice?
What stands out for you?
How is a coach approach session different from what you experience in a more traditional mentoring session?

## Slide 11
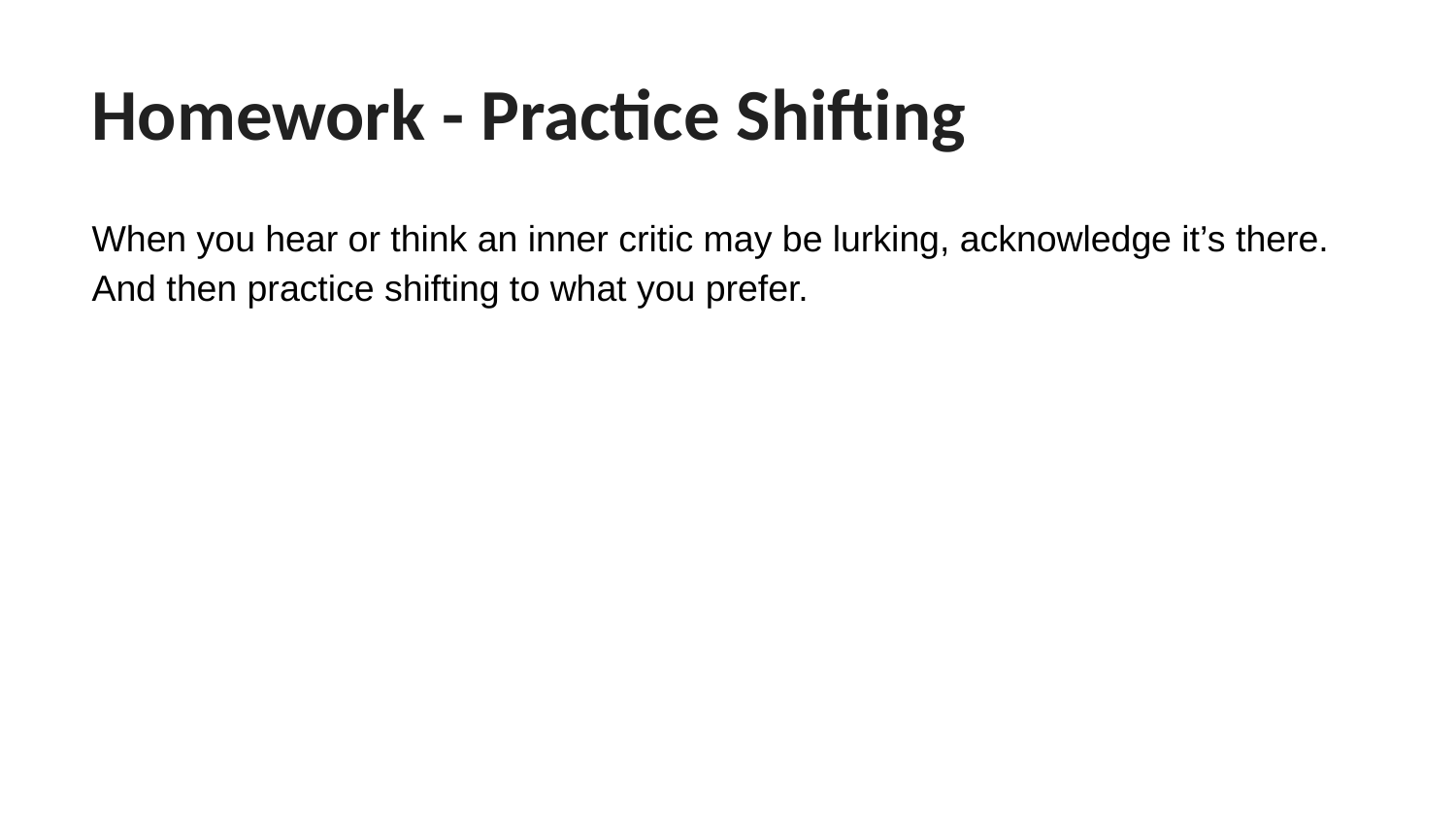

# Homework - Practice Shifting
When you hear or think an inner critic may be lurking, acknowledge it’s there. And then practice shifting to what you prefer.
