# Supplementary material for: A Longitudinal, Practical Curriculum for Faculty Development as New Coaches in Graduate Medical Education
Source: J Educ Teach Emerg Med. 2025 Jul 31;10(3):C1–C92. doi: 10.21980/J88M08 (PMC12320991; doi:10.21980/J88M08)
Supplement: Supplementary file 6 [file 10-3-C1-SuppI3.pptx]

## Slide 1
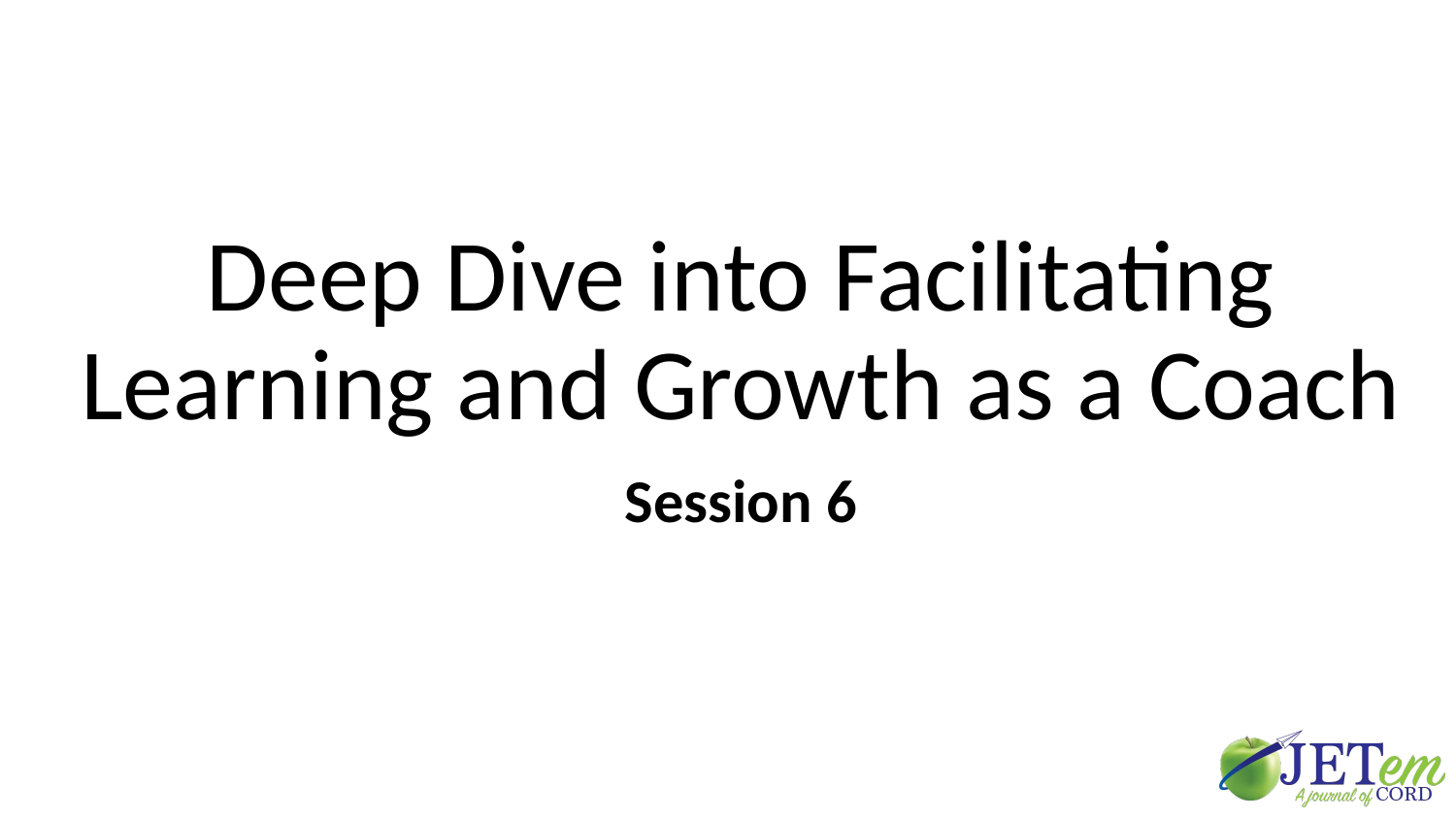

Deep Dive into Facilitating Learning and Growth as a Coach
Session 6

## Slide 2
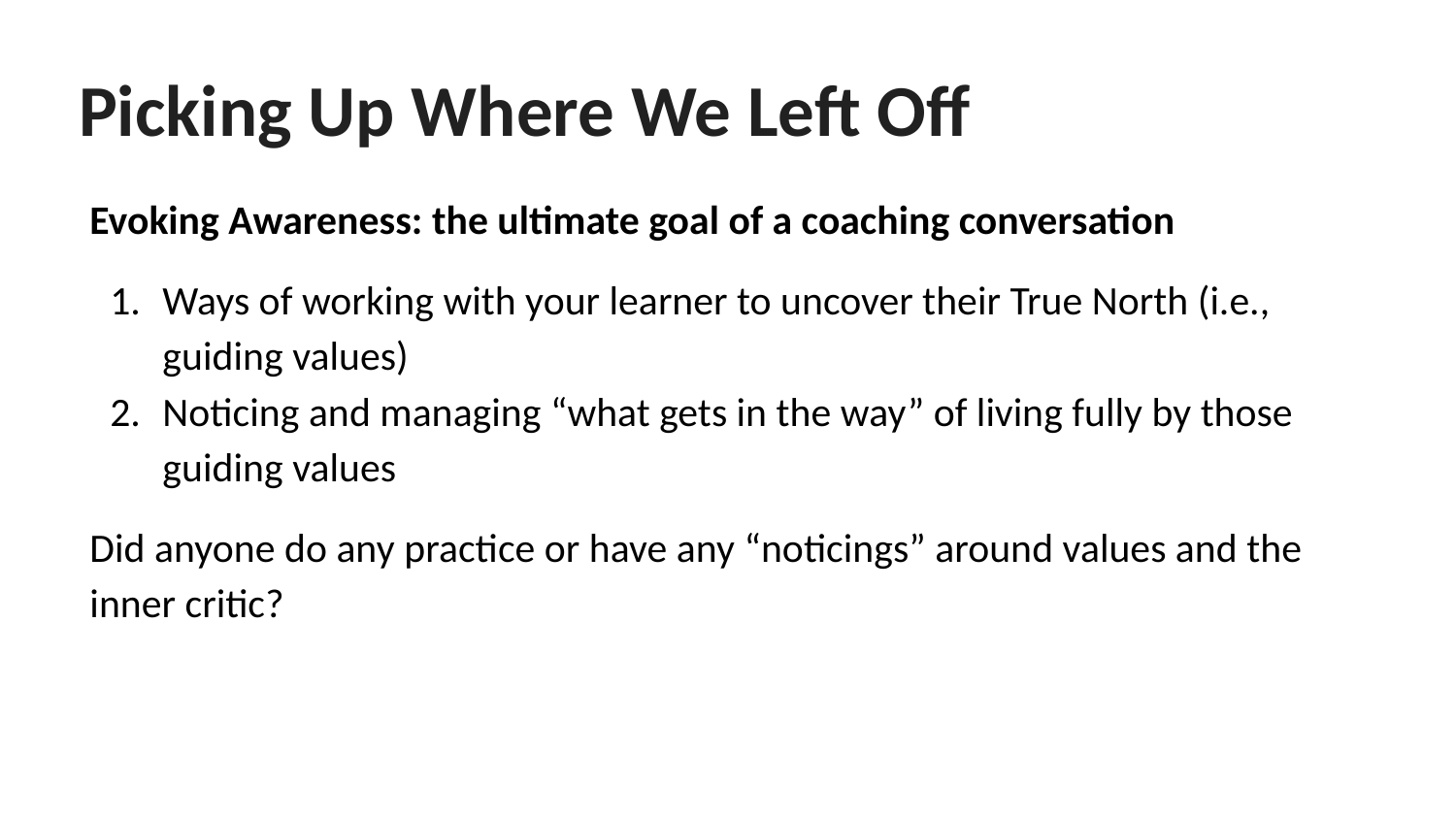

# Picking Up Where We Left Off
Evoking Awareness: the ultimate goal of a coaching conversation
Ways of working with your learner to uncover their True North (i.e., guiding values)
Noticing and managing “what gets in the way” of living fully by those guiding values
Did anyone do any practice or have any “noticings” around values and the inner critic?

## Slide 3
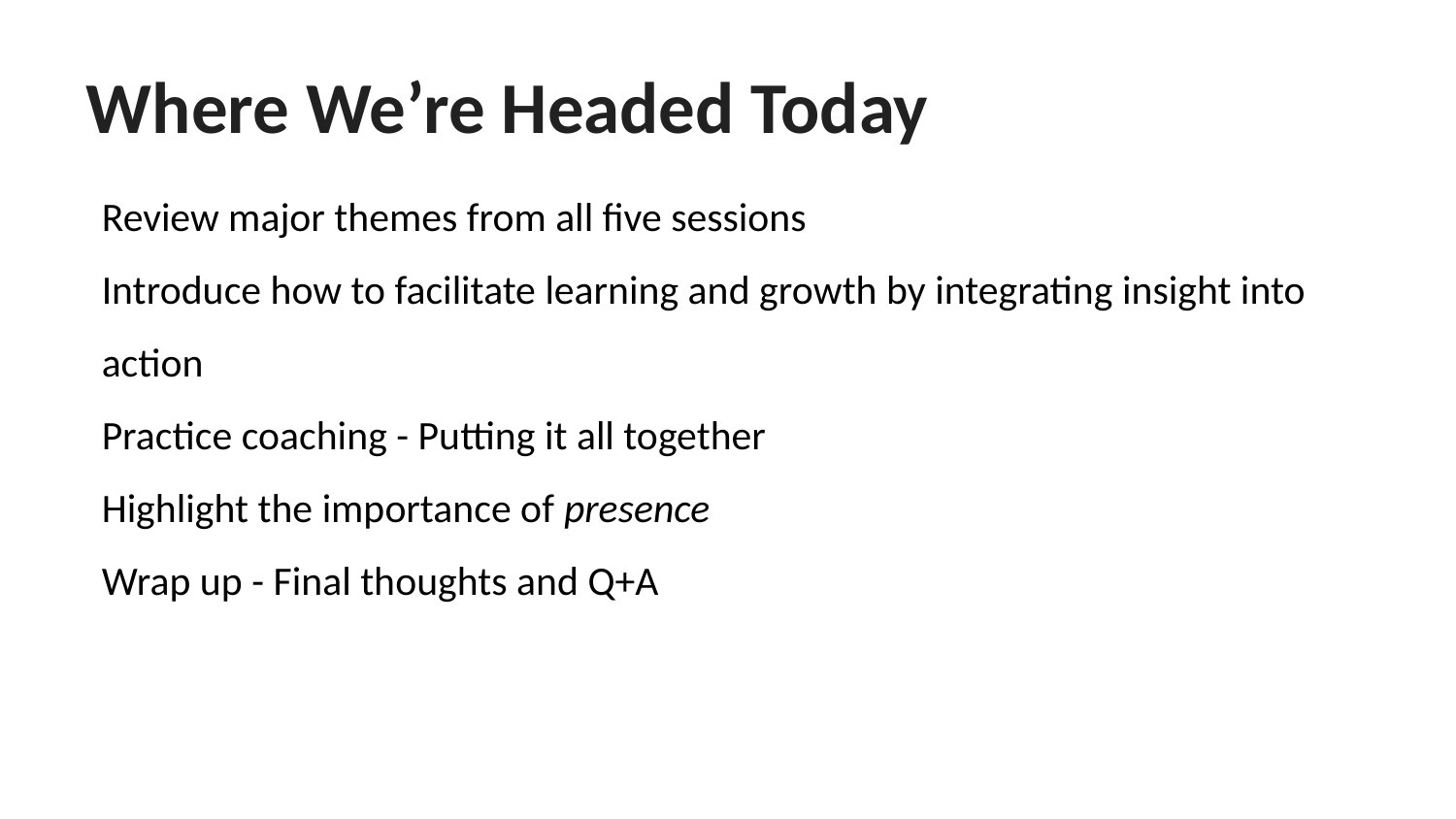

# Where We’re Headed Today
Review major themes from all five sessions
Introduce how to facilitate learning and growth by integrating insight into action
Practice coaching - Putting it all together
Highlight the importance of presence
Wrap up - Final thoughts and Q+A

## Slide 4
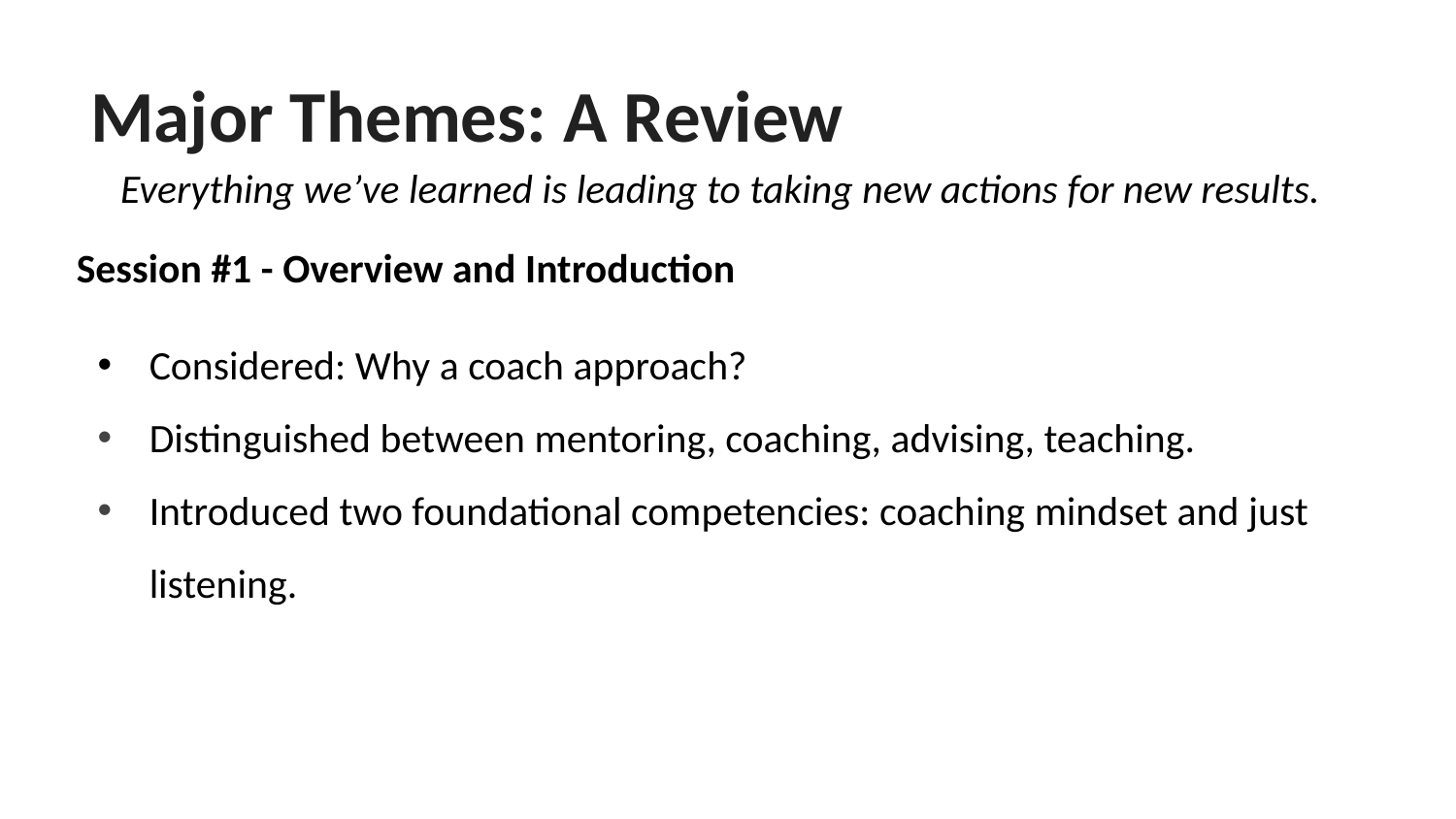

# Major Themes: A Review
Everything we’ve learned is leading to taking new actions for new results.
Session #1 - Overview and Introduction
Considered: Why a coach approach?
Distinguished between mentoring, coaching, advising, teaching.
Introduced two foundational competencies: coaching mindset and just listening.

## Slide 5
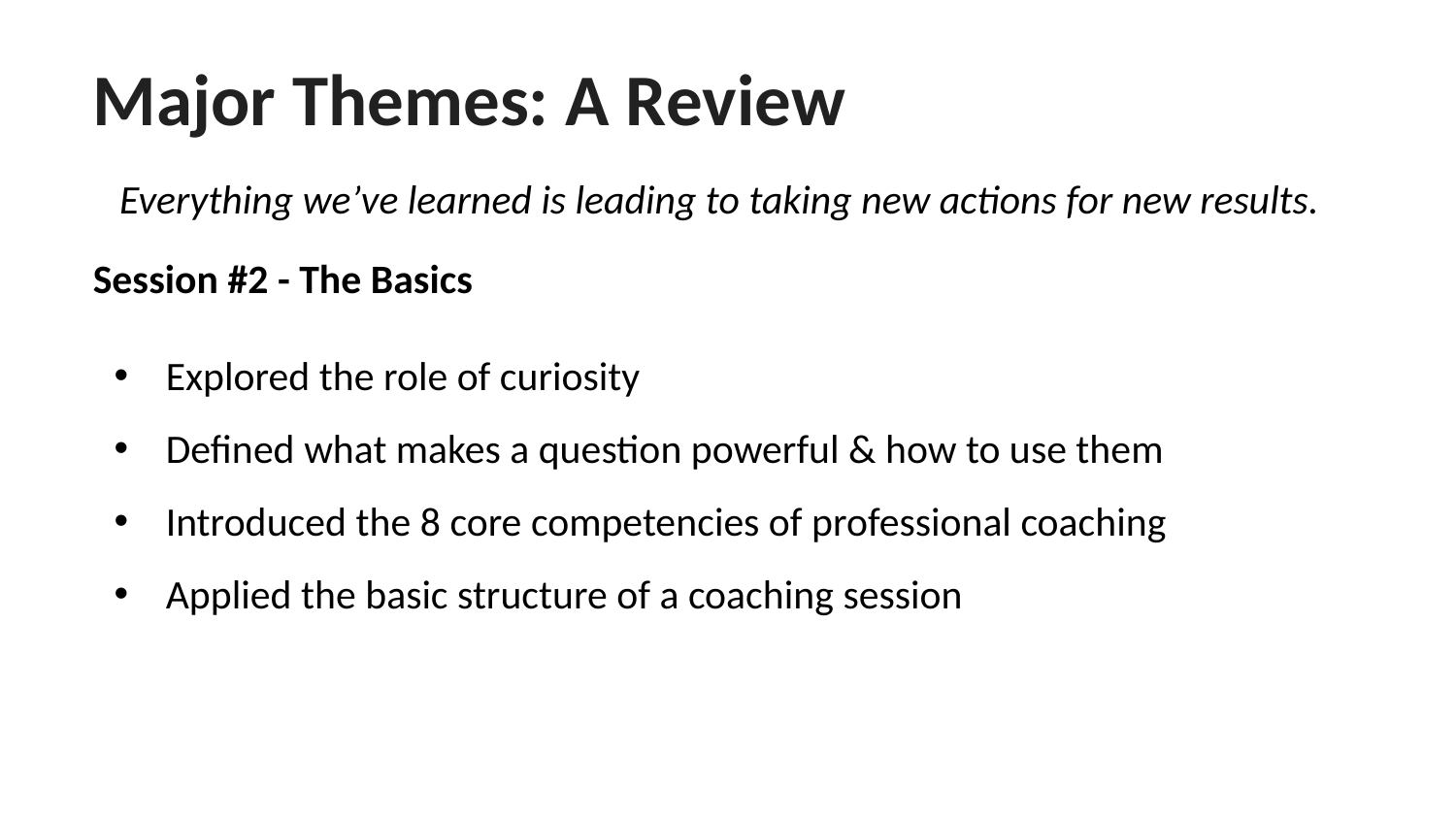

# Major Themes: A Review
Everything we’ve learned is leading to taking new actions for new results.
Session #2 - The Basics
Explored the role of curiosity
Defined what makes a question powerful & how to use them
Introduced the 8 core competencies of professional coaching
Applied the basic structure of a coaching session

## Slide 6
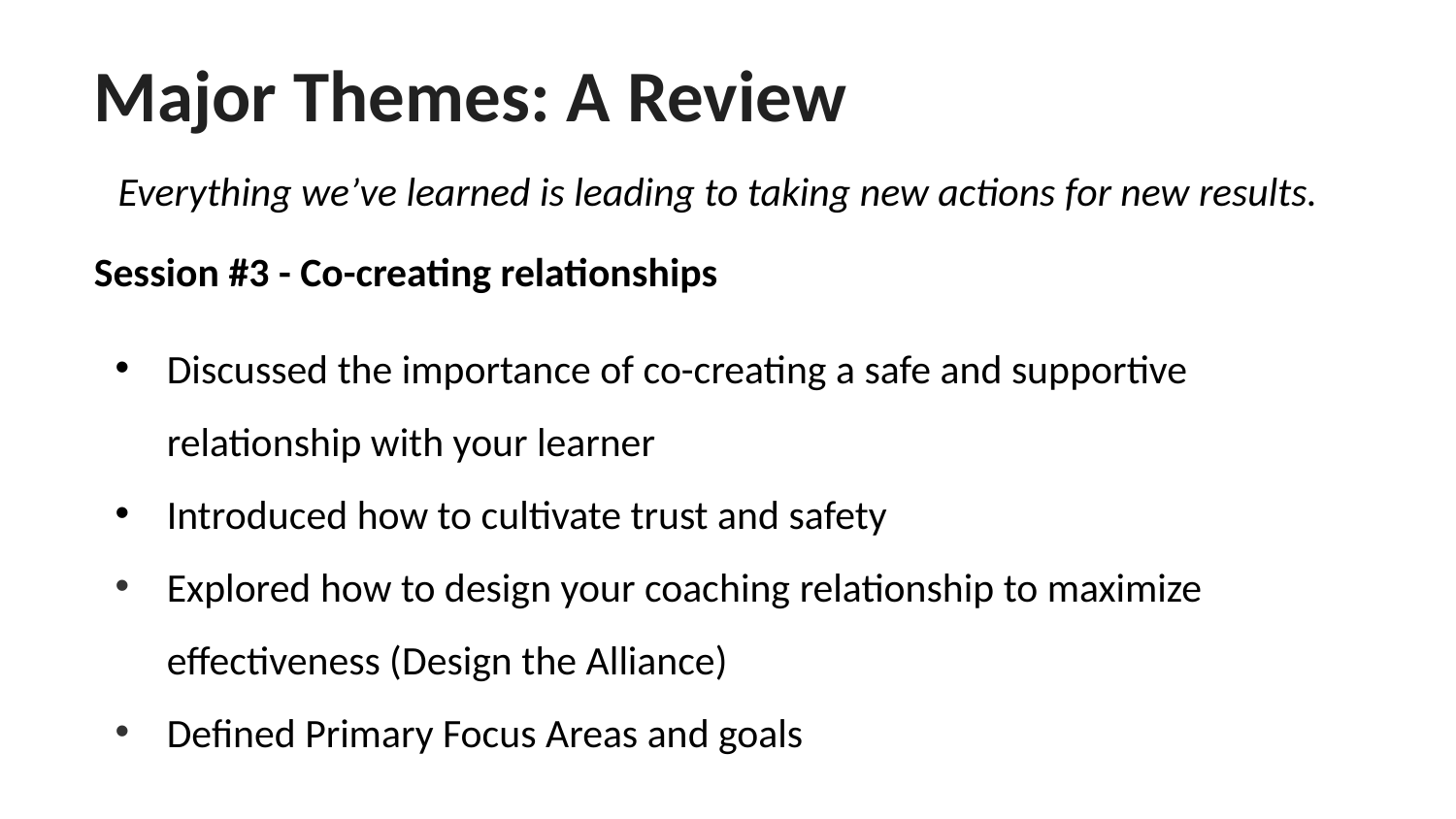

# Major Themes: A Review
 Everything we’ve learned is leading to taking new actions for new results.
Session #3 - Co-creating relationships
Discussed the importance of co-creating a safe and supportive relationship with your learner
Introduced how to cultivate trust and safety
Explored how to design your coaching relationship to maximize effectiveness (Design the Alliance)
Defined Primary Focus Areas and goals

## Slide 7
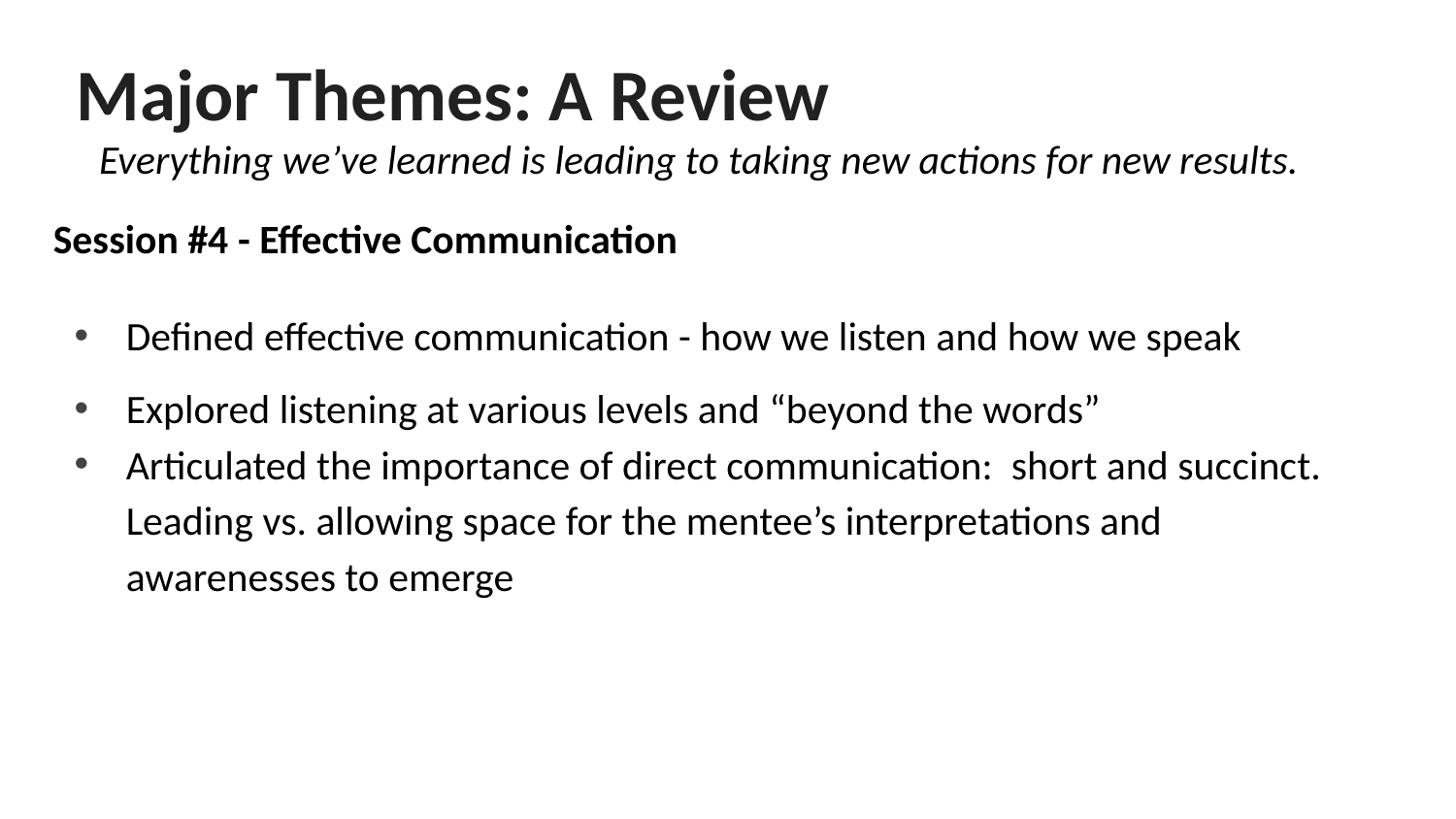

# Major Themes: A Review
Everything we’ve learned is leading to taking new actions for new results.
Session #4 - Effective Communication
Defined effective communication - how we listen and how we speak
Explored listening at various levels and “beyond the words”
Articulated the importance of direct communication: short and succinct. Leading vs. allowing space for the mentee’s interpretations and awarenesses to emerge

## Slide 8
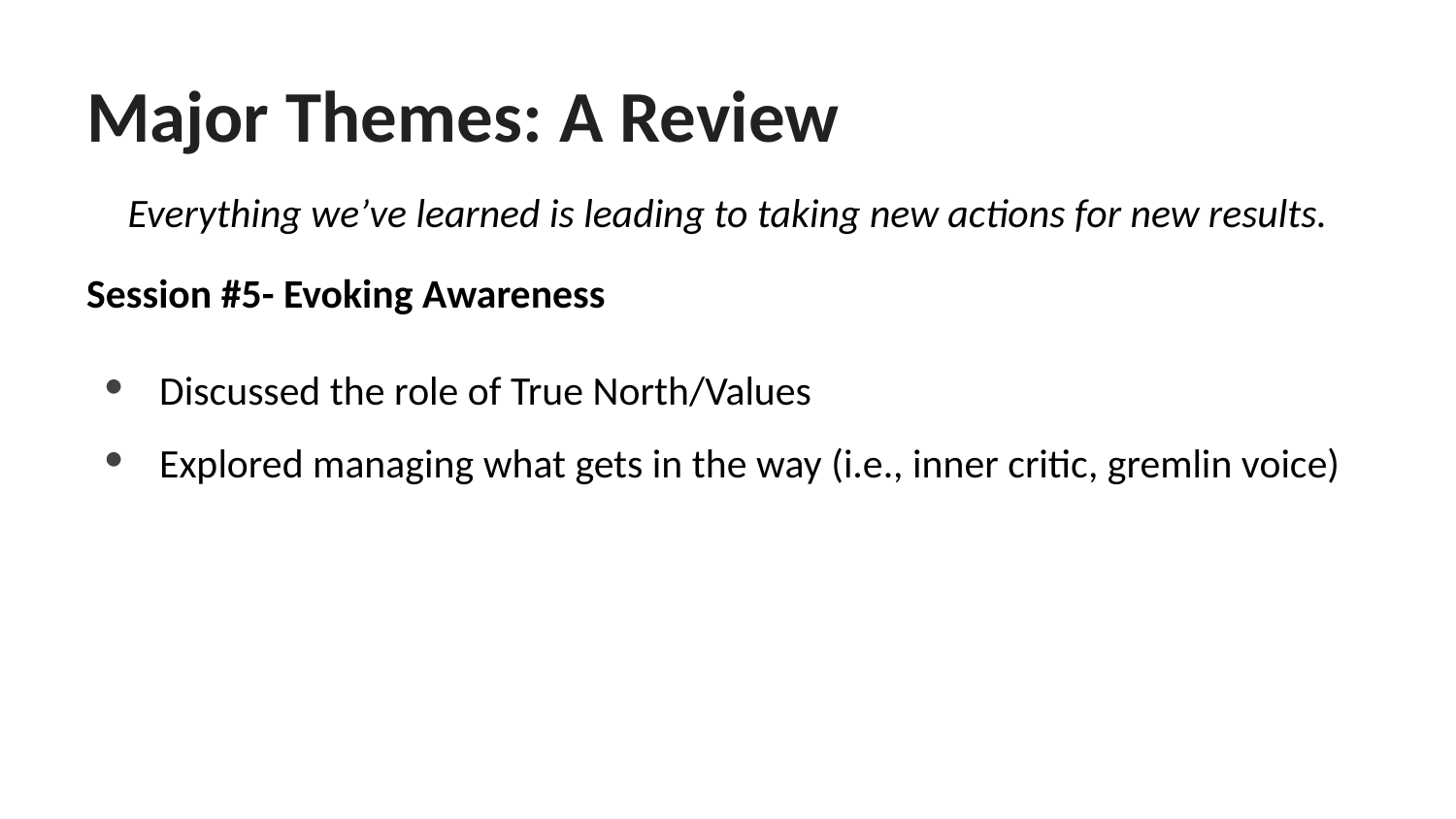

# Major Themes: A Review
Everything we’ve learned is leading to taking new actions for new results.
Session #5- Evoking Awareness
Discussed the role of True North/Values
Explored managing what gets in the way (i.e., inner critic, gremlin voice)

## Slide 9
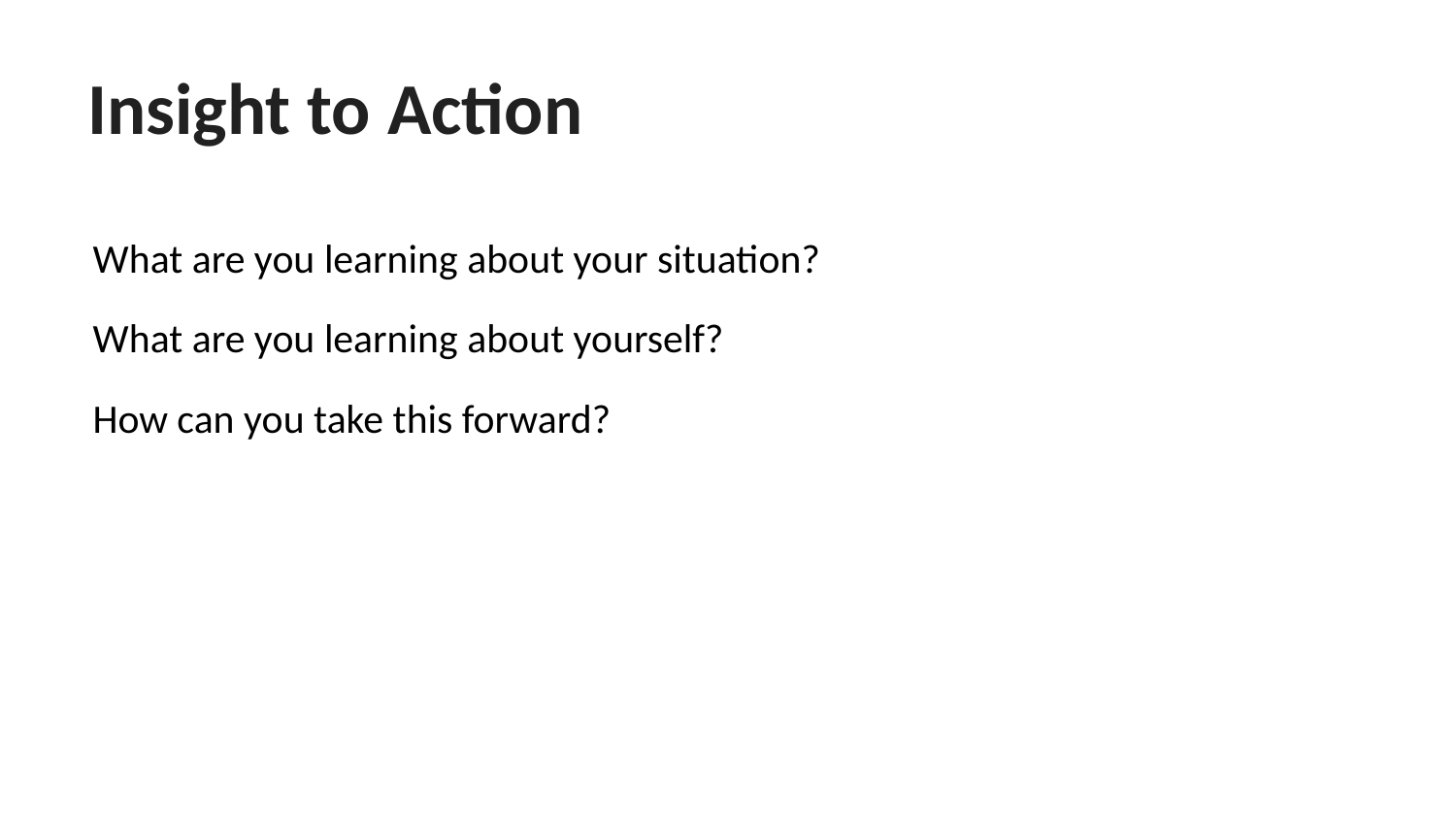

# Insight to Action
What are you learning about your situation?
What are you learning about yourself?
How can you take this forward?

## Slide 10
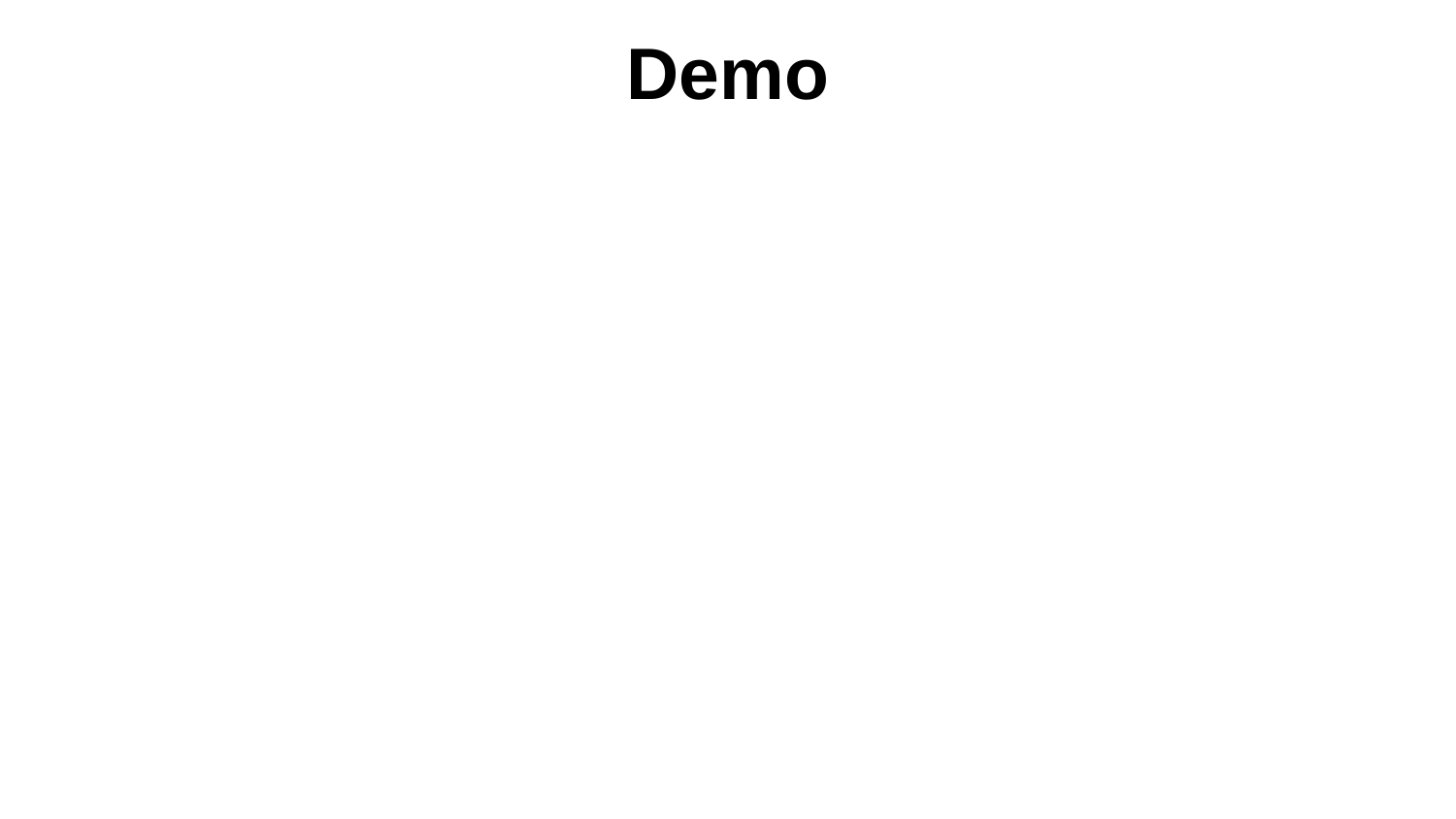

# Demo

## Slide 11
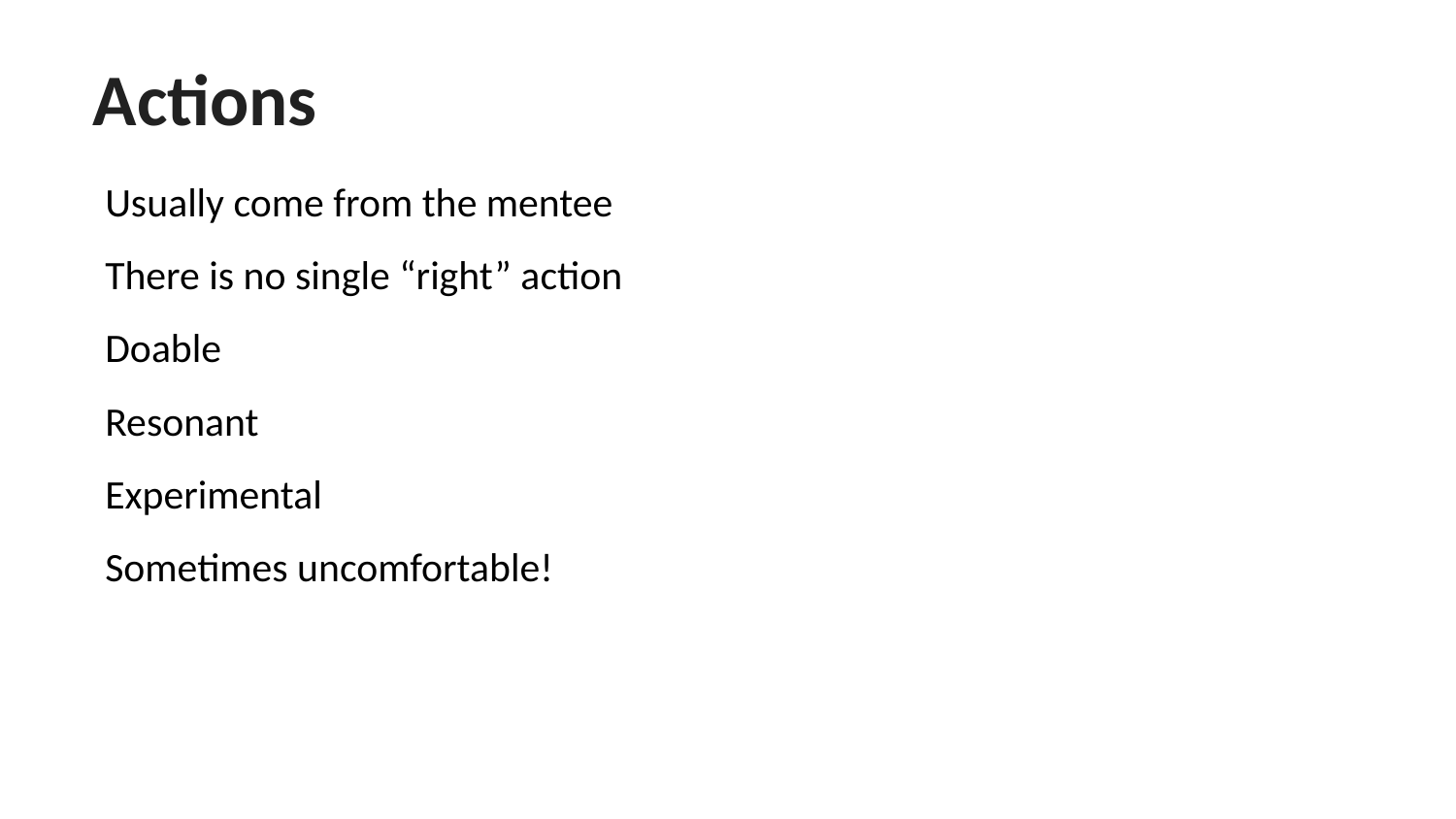

# Actions
Usually come from the mentee
There is no single “right” action
Doable
Resonant
Experimental
Sometimes uncomfortable!

## Slide 12
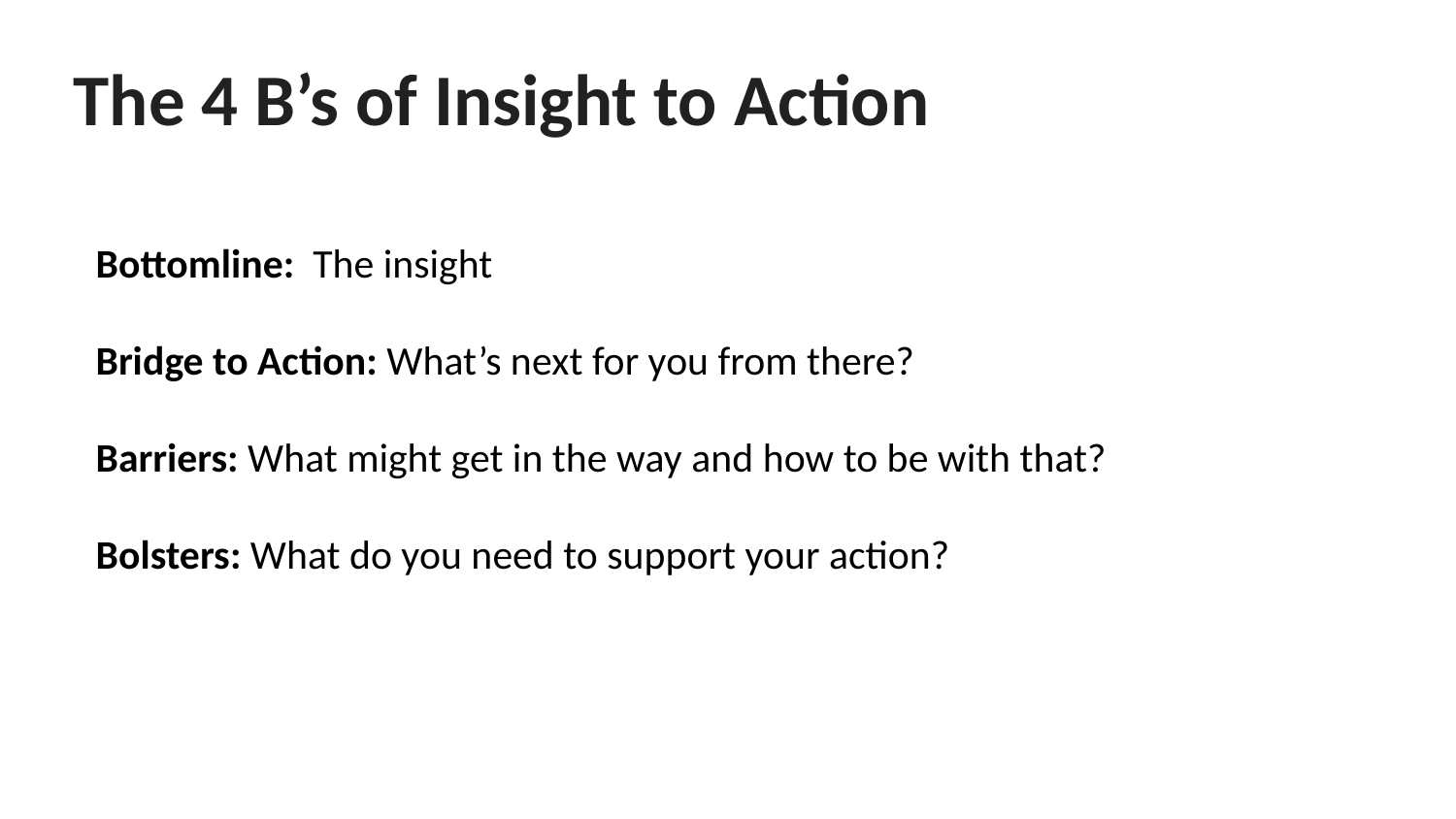

# The 4 B’s of Insight to Action
Bottomline: The insight
Bridge to Action: What’s next for you from there?
Barriers: What might get in the way and how to be with that?
Bolsters: What do you need to support your action?

## Slide 13
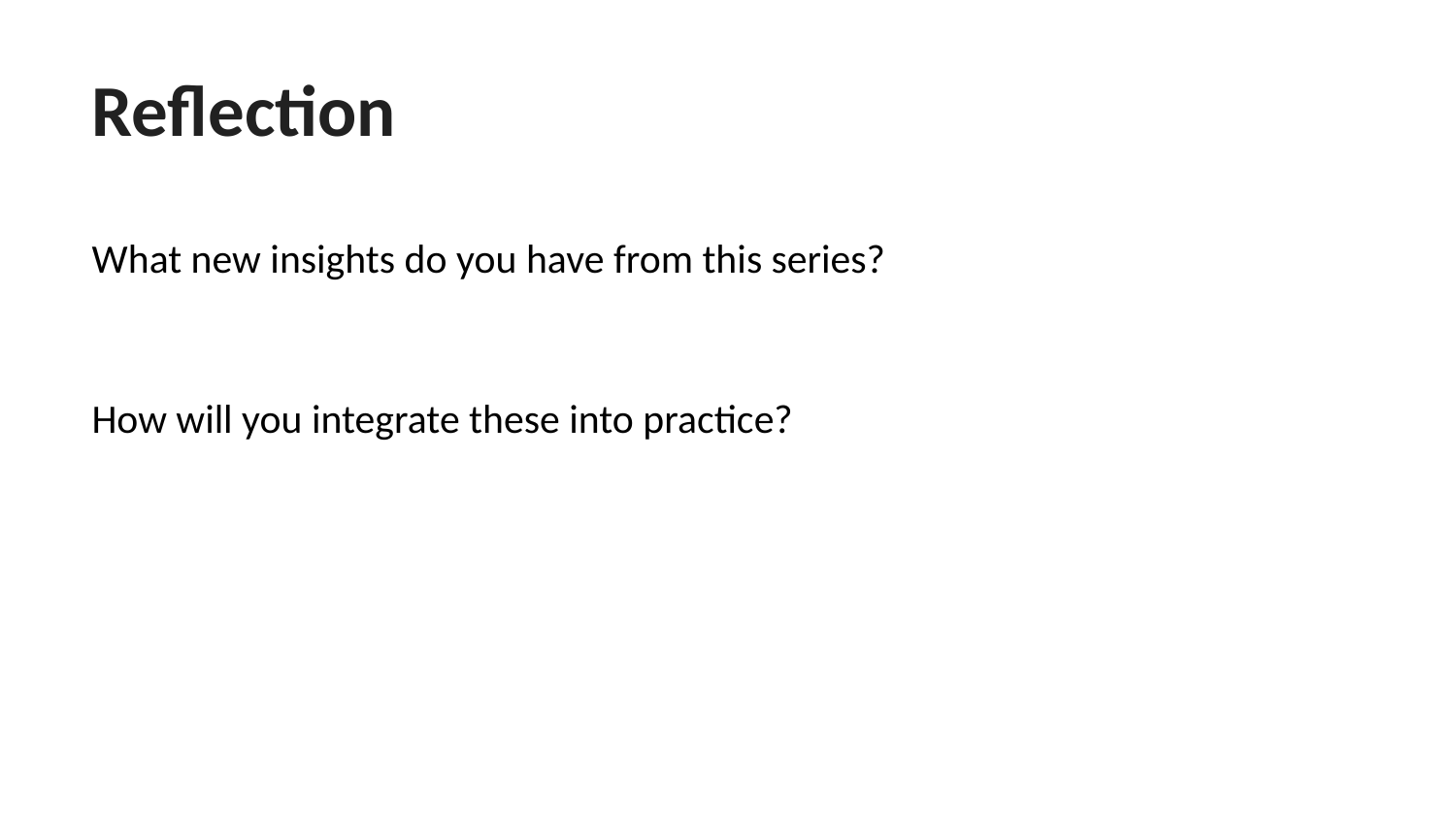

# Reflection
What new insights do you have from this series?
How will you integrate these into practice?

## Slide 14
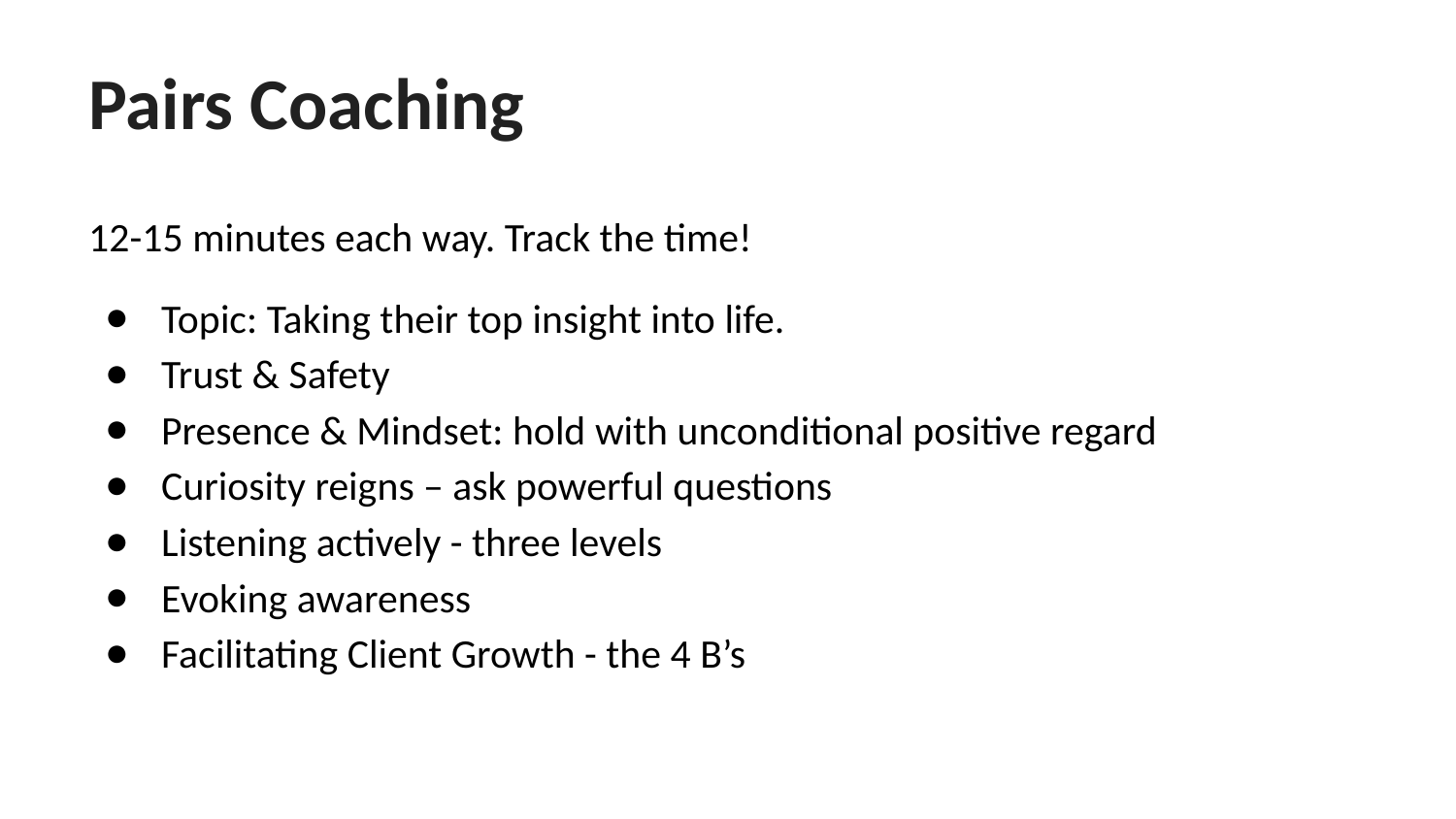

# Pairs Coaching
12-15 minutes each way. Track the time!
Topic: Taking their top insight into life.
Trust & Safety
Presence & Mindset: hold with unconditional positive regard
Curiosity reigns – ask powerful questions
Listening actively - three levels
Evoking awareness
Facilitating Client Growth - the 4 B’s

## Slide 15
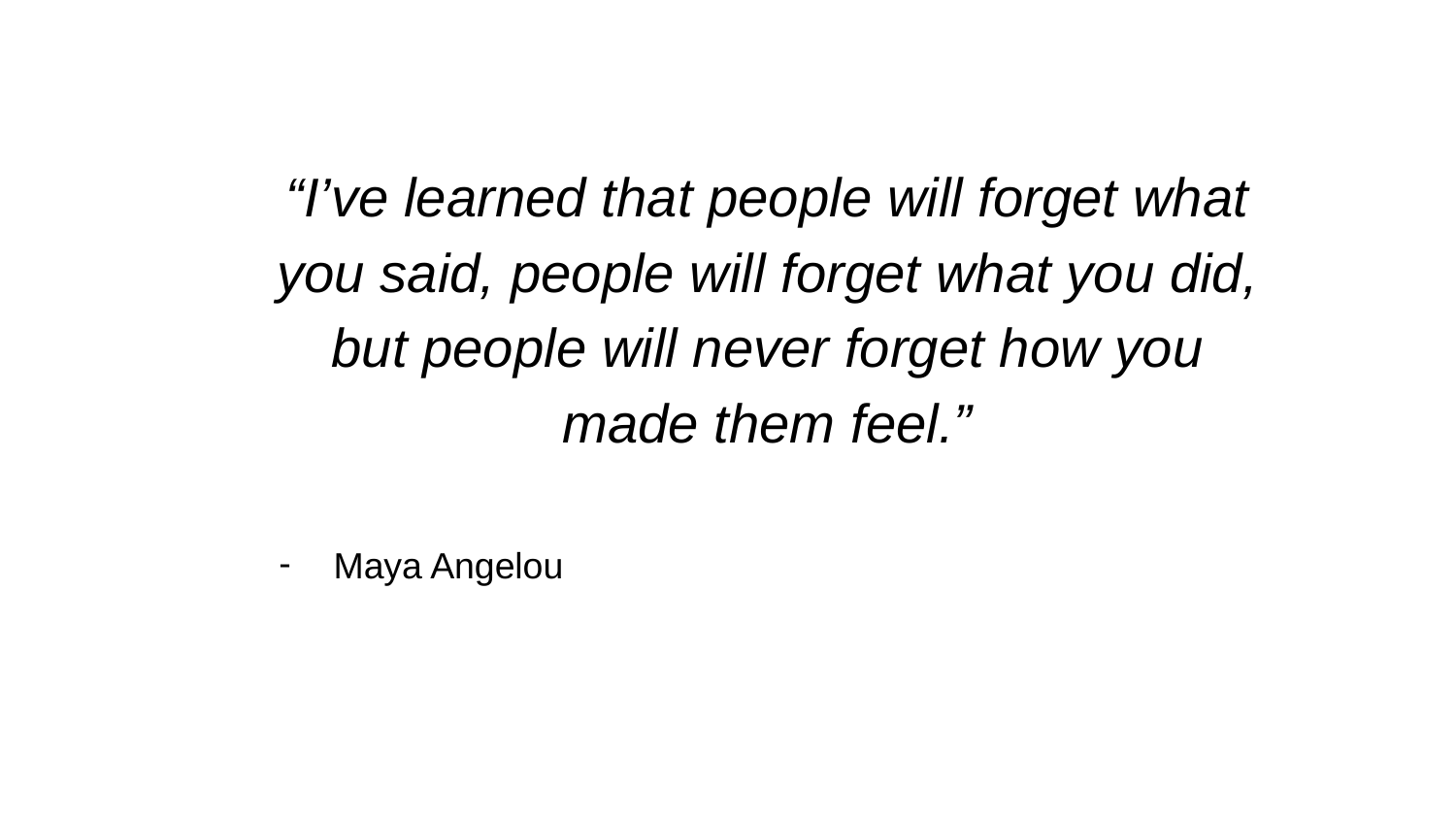

“I’ve learned that people will forget what you said, people will forget what you did, but people will never forget how you made them feel.”
Maya Angelou

## Slide 16
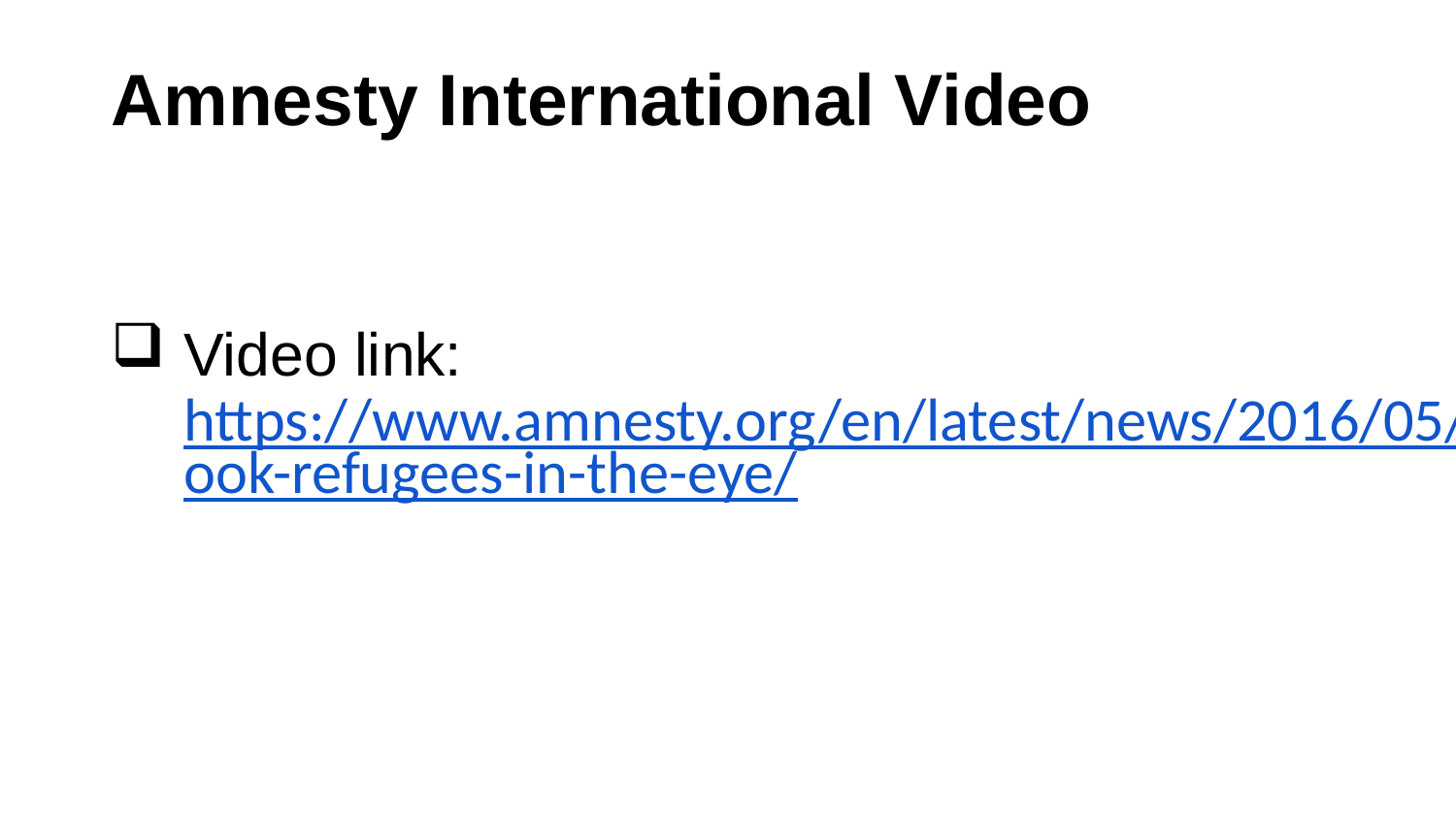

# Amnesty International Video
Video link: https://www.amnesty.org/en/latest/news/2016/05/look-refugees-in-the-eye/
